# Substrate selectivity of an isolated enoyl reductase catalytic domain from an iterative highly reducing fungal polyketide synthase reveals key components of programming†
†Electronic supplementary information (ESI) available: Details of all experimental and characterisation data. See DOI: 10.1039/c6sc03496a



**DOI:** 10.1039/c6sc03496a

**Published:** 2016-09-26

**Authors:** Douglas M. Roberts, Christoph Bartel, Alan Scott, David Ivison, Thomas J. Simpson, Russell J. Cox

**Affiliations:** a School of Chemistry , University of Bristol , Cantock's Close , Bristol BS8 1TS , UK; b Institute for Organic Chemistry , BMWZ , Leibniz Universität Hannover , Schneiderberg 1b , 30167 , Hannover , Germany . Email: russell.cox@oci.uni-hannover.de

## Abstract

A *cis*-acting enoyl reductase (ER) catalytic domain was isolated from a fungal highly reducing iterative polyketide synthase (HR-iPKS) for the first time and studied *in vitro*. The ER from the squalestatin tetraketide synthase forms a discrete dimeric protein in solution. The ER shows broad substrate selectivity, reducing enoyl species including both natural and unnatural substrates. Pantetheine-bound substrate thiolesters reacted much faster than the corresponding SNAC thiolesters. The unnatural substrates included *Z*-olefins, 2-ethyl olefins and pentaketides. Methylation of the substrate modifies the activity of the ER such that the 2,4-dimethyl oct-2-enoyl substrate fits into the active site but cannot be reduced. A new NMR-based assay was developed for the direct observation of the stereochemical preferences at the 4′ position of the NADPH cofactor and the C-2 and C-3 positions of the substrates. The assay reveals that the fungal iPKS ER-catalysed reaction is stereochemically identical to that of the vertebrate FAS (vFAS) at the cofactor 4′ position and the substrate 3-position, but the high stereoselectivity displayed by intact SQTKS is lost such that reprotonation at the 2-position is unselective by the isolated ER. A 3D model of ER was consistent with these observations and showed that the ER may sequester its final substrate to prevent further chain extension. The results support a developing model for programming by HR-iPKS in which competition for substrates between restrictive and permissive catalytic domains chaperones the growing polyketide to completion, while allowing for errors and evolution.

## Abstract 2

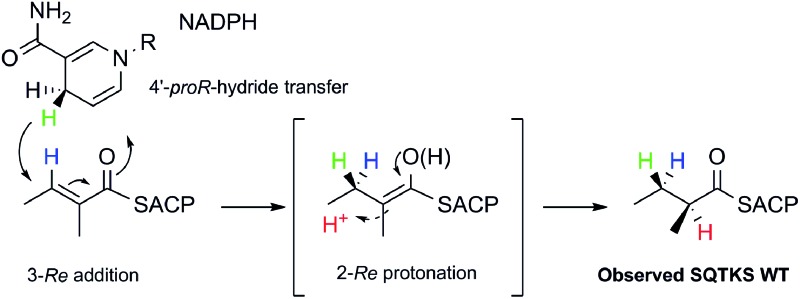
The complete stereochemical course and substrate selectivity of the enoyl reductase domain from the fungal polyketide synthase squalestatin tetraketide synthase (SQTKS) have been determined.

Fungal polyketides are highly diverse secondary metabolites which are created by iterative type I polyketide synthases (iPKS).^1,2^ These enzymes differ from the better-understood modular polyketide synthases (mPKS) present in bacteria in which each synthesis module is usually responsible for a single chain extension and modification cycle. The synthetic programme of mPKS arises as a result of the number of modules (which controls the chain-length) and the presence or absence of modifying domains in each individual module (which dictate the chemical functionality at each backbone carbon).^3^ The consequence is that the chemical products of modular PKS can often be predicted from the PKS peptide sequence. In contrast, fungal PKS consist of single modules which are iterative. Since neither the number of cycles of chain extension nor the operation of each individual modifying domain in each successive cycle can be predicted, the programmes of fungal PKS (and indeed the *programming mechanisms* themselves) remain cryptic.

The fungal highly-reducing (HR) class of iPKS consist of β-ketoacyl ACP synthase (KS), acyl transferase (AT), dehydratase (DH), *C*-methyl transferase (*C*-MeT), enoyl reductase (ER), keto-reductase (KR) and acyl carrier protein (ACP) catalytic domains. They are thus very similar in domain-order to single modules of mPKS and also the iterative vertebrate fatty acid synthases (vFAS)^4^ – to which they also show significant sequence homology. However, vFAS enzymes display almost no programming as all domains are active in every cycle, and chain-length is dictated by a specialised C-terminal thiolesterase (TE) which releases the fatty acid when it reaches the predesignated length.^5^ Similarly, single mPKS modules have no intrinsic programmes – they usually use all the domains available to them. Thus the fungal HR-iPKS are uniquely programmed.

A typical example of a HR-iPKS is the squalestatin tetraketide synthase (SQTKS) which catalyses the synthesis of **1** from acetate, malonate, *S*-adenosyl methionine (SAM) and NADPH.^6^ **1** forms the sidechain of squalestatin S1 **2**,^7,8^ a potent inhibitor of squalene synthase and potential anticholesterol compound.^9^ SQTKS performs three rounds of chain extension catalysed by the AT and KS domains. After the first extension the chain is methylated (*C*-MeT), the β-carbonyl is reduced (KR), the chain is dehydrated (DH), and final enoyl reduction (ER) saturates the chain and installs the stereochemistry at the α-methyl position (Scheme 1A). The second round of extension and modification is the same; but after the third round of extension no methylation or enoyl reduction occur and further synthesis ceases.

**Scheme 1:**
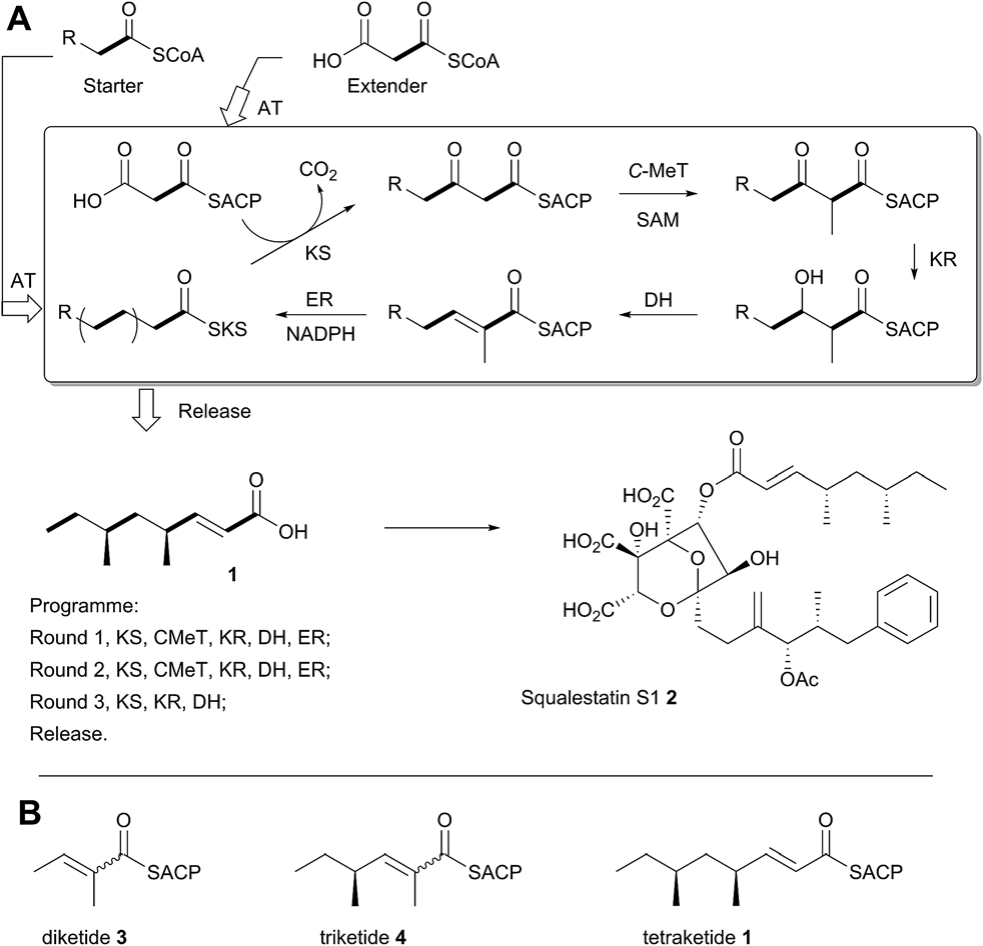
Programmed biosynthesis of squalestatin tetraketide synthase. (A) Iterative reactions catalysed by SQTKS; (B) deduced substrates for the ER domain.

In previous work we have investigated the programming of the tenellin HR-iPKS (known as TENS) through the construction of domain-swap chimeras with donor domains from the desmethylbassianin synthase which makes a longer, but less methylated polyketide.^10^ That work showed that some domains, such as *C*-MeT, may control their own programming, possibly *via* strict substrate selectivity – in other words domains may accept or reject varying substrates presented by the ACP. However exchange of the chain-extending KS domain, and chain off-loading domains did not change the chain-length. Instead, chain-length was strongly affected by exchange of the KR domain. This leads to an alternative hypothesis in which *kinetic competition* between domains could control programming choices.^10^

In order to investigate these hypotheses in more detail, and whether similar factors affect other HR iPKS domains which have not thus-far been investigated, we set out to examine the selectivity of isolated HR-iPKS domains *in vitro*. For this purpose we selected the SQTKS ER domain which is active after the first two chain extensions (*i.e.* diketide **3** and triketide **4** are substrates), but inactive after the third extension (*i.e.* tetraketide **1** is not a substrate, Scheme 1B).

## Results

### Protein production, substrate synthesis and assay procedure

SQTKS is encoded by the *phpks1* gene from Phoma species C2932 and it has previously been heterologously expressed in *Aspergillus oryzae*.^6^ In order to obtain sufficient soluble protein for *in vitro* assays, however, we reconstructed *phpks1* using synthetic DNA optimised for *E. coli* expression by homologous recombination in yeast. The resulting full-length *phpks1* was then used as a template for PCR using a number of possible PCR primers around the putative ER encoding sequence (see ESI for details†). The resulting PCR products were expressed in *E. coli* as his_6_-tagged fusion proteins and the clone which produced the most soluble protein selected for further study. In general protein production and purification of the isolated domain was complicated by low yields, instability and precipitation, but significant improvements to standard procedures included low temperature induction, use of low IPTG concentrations and inclusion of 20% glycerol in all purification and storage buffers. MS analysis of the protein (MALDI and ESMS) confirmed the expected size (38.9 kDa), and calibrated gel-filtration chromatography indicated that the ER domain exists as a dimer in solution (see ESI†).

Initial *in vitro* assays consisted of the isolated ER protein, NADPH (NADH was not turned-over), the diketide tigloyl SNAC **5S** and buffer. The reaction was followed over a period of hours by LCMS which clearly showed the slow conversion of the substrate to 2-methylbutyryl SNAC **6S**. The ER could also be assayed by directly observing NADPH consumption at 340 nm. The mPKS spinosyn (KR–ER)2 didomain has been similarly investigated *in vitro* (*vide infra*). In the case of the spinosyn ER, hydration of a crotonyl substrate was observed,^11^ but addition of water was not observed in our assays (LCMS analysis).

### Substrate selectivity

**Scheme 2:**
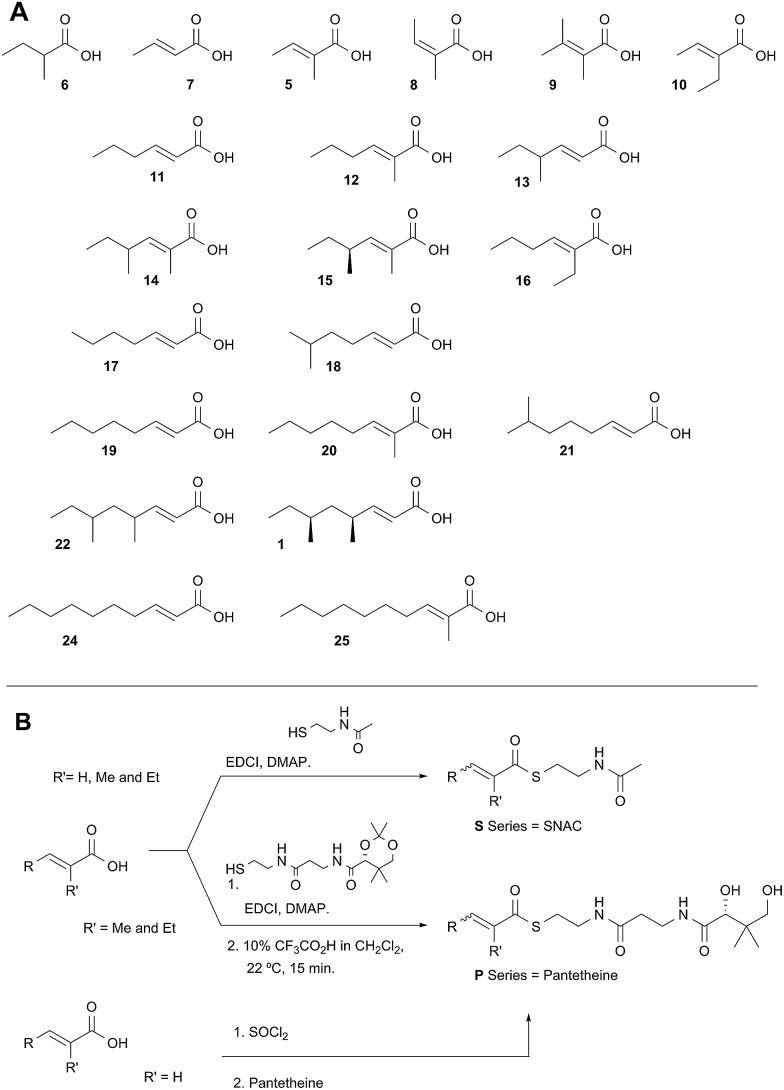
Synthesis of substrate SNACs and pantetheines. (A) Structures assessed; shown as free acids and designated, *e.g.* **5S** or **5P**, for the corresponding SNAC and pantetheine thiolesters; (B) synthetic routes to SNACs and pantetheines.

Diketide and longer substrate acids (**5** and **7–25**) were either commercially available or synthesised by standard methods (see ESI†). The acids were then coupled with SNAC (to give series **S**) by standard procedures. The acyl pantetheines (series **P**) were made either by coupling to the 10′,12′ dimethyl acetonide of pantetheine,^12^ followed by deprotection (for the 2-substituted series) or by conversion to the corresponding acyl chloride and direct thiolesterification with pantetheine itself (for the 2-unsubstituted series, Scheme 2B). All pantetheines were purified by mass-directed HPLC fractionation. All compounds were fully characterised prior to assays (see ESI†).

**Fig. 1:**
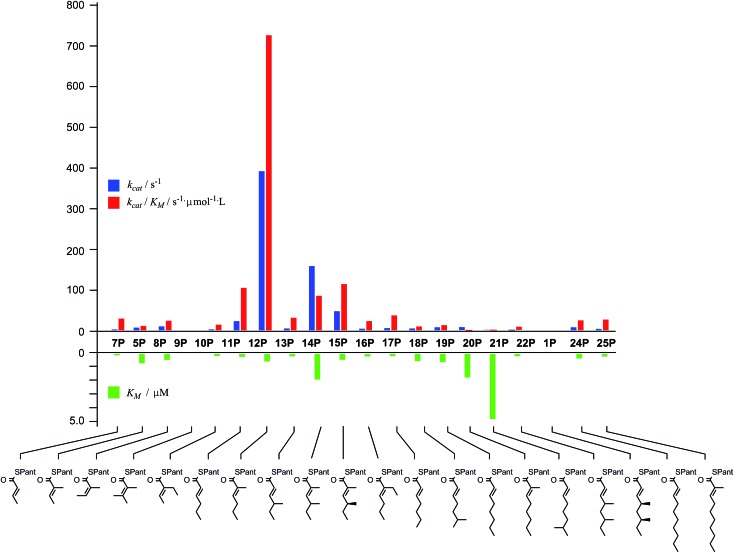
Kinetic parameters obtained for *in vitro* assay of the indicated pantetheine substrates with the isolated ER domain. Compounds **9P** and **1P** showed no measurable activity.

In general SNACS are relatively poor substrates of the isolated ER, and for triketide and longer SNAC substrates (*e.g.* **15S**) low solubility becomes a limiting factor. However, acyl pantetheines are generally better substrates than the corresponding acyl SNACS, *e.g.* tigloyl pantetheine **5P** was reduced approximately 12 times faster by the isolated ER domain under standardised conditions than tigloyl SNAC **5S** (see ESI†). For these reasons the pantetheine series of substrates was used to characterise the substrate selectivity of the isolated ER domain. *K*_M_ and *k*_cat_ values were measured (Fig. 1) using the continuous spectrophotometric assay (see ESI†).

Squalestatin tetraketide **1P** itself is not a substrate for the ER *in vivo* or *in vitro*. However it does act as an inhibitor of the ER *in vitro*, showing observable reduction in turnover of the substrate **5P** (see ESI†).

### Stereoselectivity

**Fig. 2:**
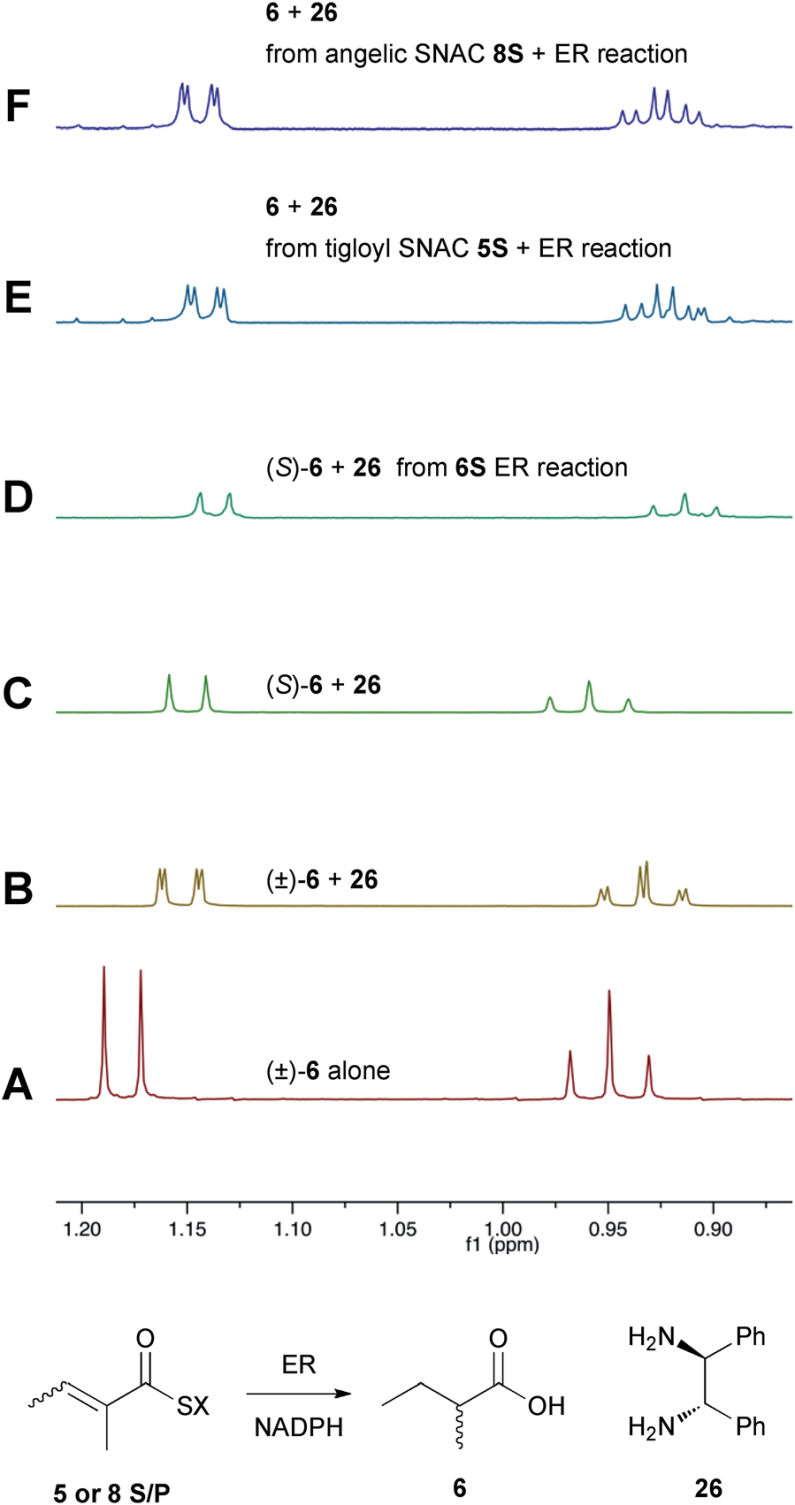
Stereochemical outcome at the 2-position after reduction by the isolated ER domain. NMR spectra show the methyl region of **6** at 500 MHz.

Previous studies of the stereoselectivity of ER proteins have relied on classical methods, relying on the use of auxiliary enzymes with known stereoselectivities to determine the site of label incorporation.^13^ We wished to develop a new, more rapid, and directly observed assay and extend previous work which had shown that NMR is a convenient tool for stereochemical assessment.^14^ In initial work, and in order to study the stereochemistry of the 2-methylbutyrate **6** produced by the isolated SQTKS ER domain, we used Parker's *in situ* NMR assay for determination of chirality at the 2-position of carboxylic acids.^15^ The ^1^H NMR spectrum of racemic 2-methylbutyric acid (±)-**6** was measured first (Fig. 2A) at 500 MHz in CDCl_3_. In the presence of 1*R*,2*R*-1,2-diphenylethylenediamine **26** the methyl resonances of **6** are shifted to higher field and the *R* and *S* enantiomers are resolved (Fig. 2B). When the same conditions are applied to enantiopure 2*S*-2-methylbutyric acid a single set of resonances is observed as expected (Fig. 2C).

In an initial control assay *S*-2-methylbutyryl SNAC **6S** was incubated with the ER and all other assay components for 24 h. At the end of the reaction the SNAC was hydrolysed and the reaction mixture was acidified to pH 3, extracted directly into CDCl_3_ and two equivalents of 1*R*,2*R*-1,2-diphenyl-ethylenediamine **26** was added before examination by ^1^H NMR. This showed the material remained enantiopure, indicating that no racemisation of the reaction product occurred during the assay, hydrolysis and isolation procedures (Fig. 2D).

Next, the isolated ER was incubated with tigloyl SNAC **5S** and an excess of NADPH for 24 h. The reaction was treated as described above and the extracted 2-methylbutyric acid **6** examined by ^1^H NMR. Identical conditions were applied to angelic SNAC **8S**. In both cases the ^1^H NMR indicated that racemic product was produced (Fig. 2E and F). The same results were obtained for the corresponding pantetheine thiolesters **5P** and **8P**.

**Scheme 3:**
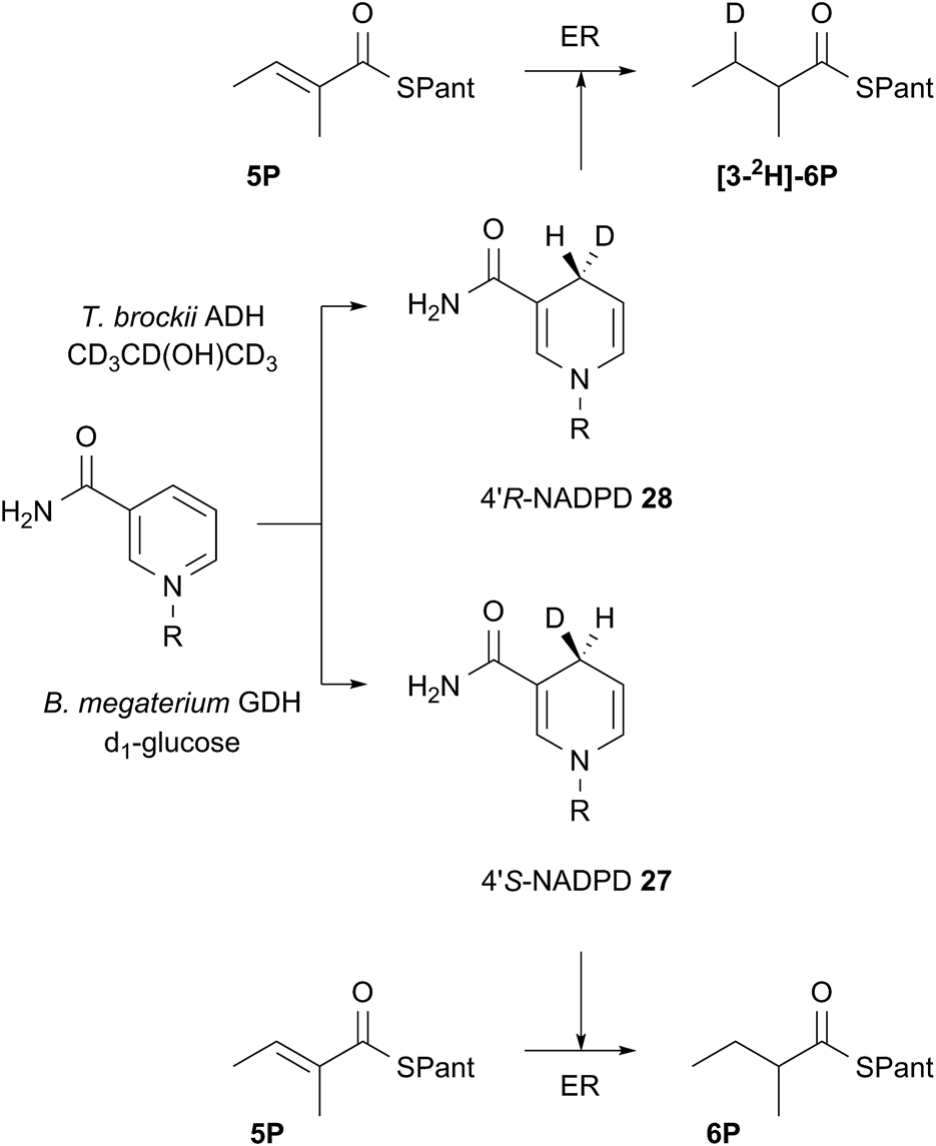
Stereoselectivity at the cofactor. Preparation and use of NADPD stereoisomers. ADH = alcohol dehydrogenase; GDH = glucose-1-dehydrogenase. R = adenine dinucleotide phosphate.

Both 4′-^2^H diastereomers of NADPH were prepared by literature procedures^16^ and shown to incorporate >98% ^2^H by MS analysis (Scheme 3, see ESI†). The 4′-*S* **27** and 4′-*R* **28** labelled cofactors were individually incubated with tigloyl-pantetheine **5P** and ER for 24 h. Reactions were slow because of kinetic isotope effects and as much ER protein was used as possible to compensate. At the end of reaction the 2-methylbutyryl pantetheine **6P** product was isolated and examined by MS. In the case of 4′-*S* ^2^H NADPH **27** no ^2^H was incorporated, but >95% ^2^H incorporation was observed for the 4′-*R* ^2^H diastereomer **28** (Scheme 3).

**Scheme 4:**
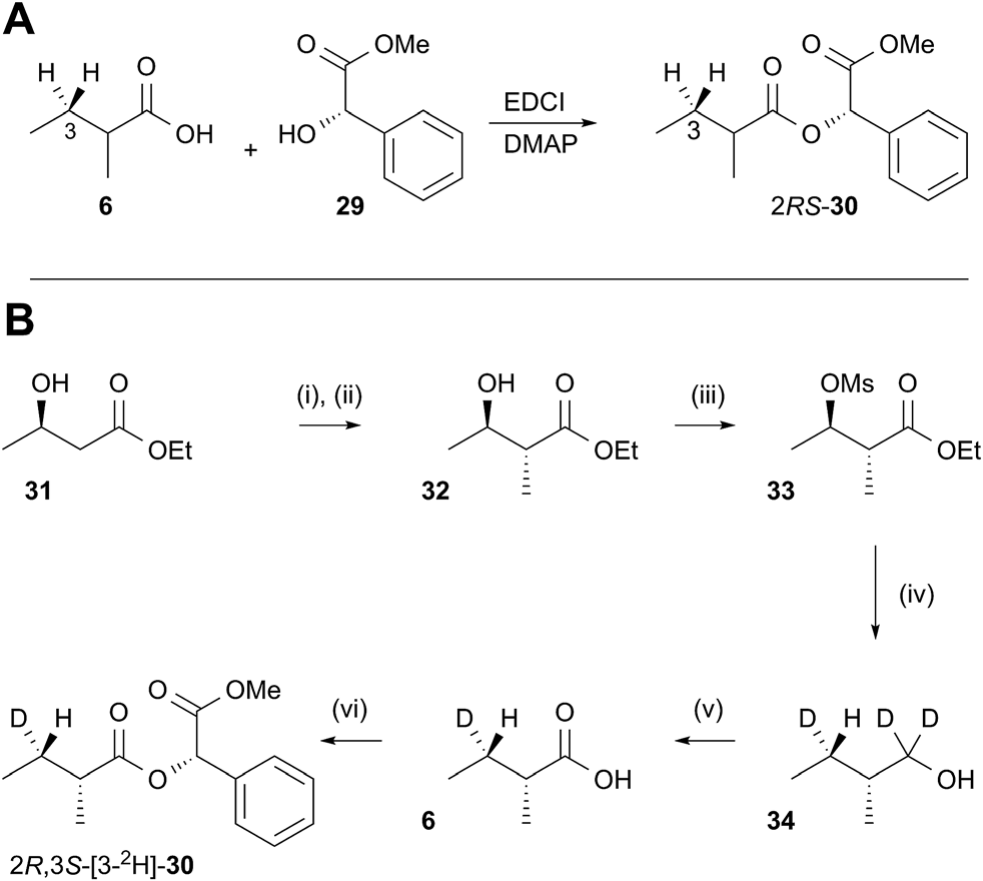
Synthesis of [3-^2^H]-2*R*,3*S*-2-methylbutanoic acid **6** and its methylmandelate ester **30**: (i) LDA; (ii) MeI, 69%; (iii) MsCl, pyr, 76%; (iv) LiAlD_4_; (v) Jones; (vi) methylmandelic acid, EDCI, DMAP, 14% (three steps).

**Fig. 3:**
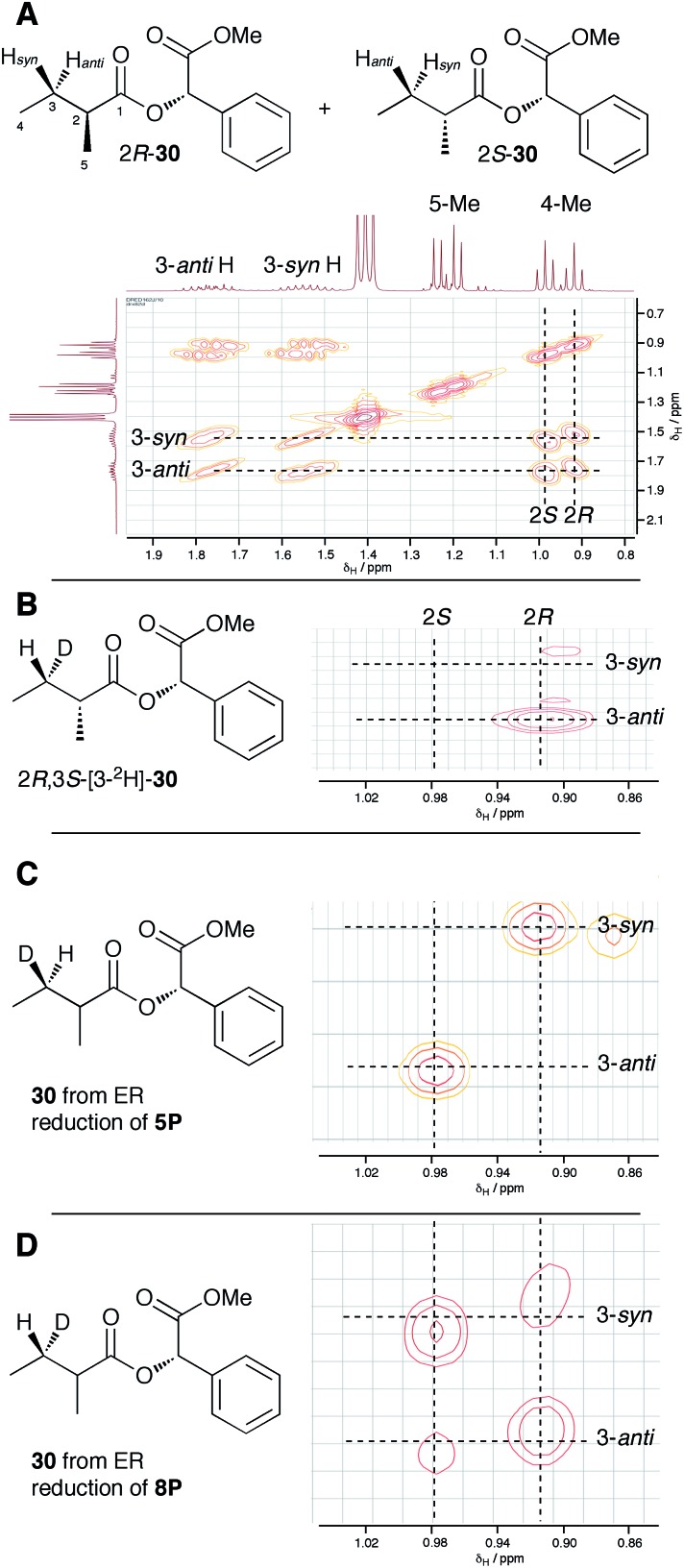
Stereochemical outcome of ER reduction at the 3-position. (A) COSY NMR of 2*RS*-2-methylbutyric methylmandelate ester **30**. (B) Assignment of 2*R*-methyl and 3-*anti*-H from synthetic material, expansion of 4-methyl region; (C) results from incubations with tigloyl pantetheine **5P**, expansion of 4-methyl region; (D) results from incubations with angelic pantetheine **8P**, expansion of 4-methyl region.

In order to examine the stereoselectivity of the reduction at the substrate 3-position, mandelate esters were examined for their ability to resolve the diastereotopic 3-hydrogens of **6** using NMR spectroscopy. Unlabelled material was synthesised by coupling 2*RS*-2-methylbutyric acid (±)-**6** with 2*S*-methylmandelate **29** to give the methylmandelate ester 2*RS*-**30** (Scheme 4A). The total of four X – 3 protons from both diastereomers were not resolved in the 1D ^1^H NMR spectrum (500 MHz), but were clearly resolved by correlation spectroscopy (COSY, Fig. 3A).

In order to assign the diastereotopic C-3 protons in the COSY spectrum, [3-^2^H]-2*R*,3*S*-2-methylbutyrate **6** was synthesised and coupled to form the corresponding 2*S*-mandelate ester [3-^2^H]-2*R*,3*S*-**30**. The route started from ethyl 3*R*-3-hydroxybutyrate **31** which was treated with 2 equivalents of LDA and one equivalent of MeI to form the 2*R*,3*R*-2-methyl-3-hydroxy butyrate **32**. The stereoselectivity of this Frater–Seebach methylation is well established to give the *anti* product.^17^ Activation of the alcohol as the mesylate **33** was followed by reduction with LiAlD_4_ to form [1,1,3-^2^H_3_]-2*R*,3*S*-2-methylbutanol **34**. The alcohol was oxidised to [3-^2^H]-2*R*,3*S*-2-methylbutanoic acid **6** using Jones reagent and the resulting acid was esterified with 2*S*-methyl mandelate to give the ester **30** (Scheme 4B). Examination of the ^1^H NMR spectra of [3-^2^H]-2*R*,3*S*-**30** showed that the 4-methyl group of the 2*R* diastereomer resonates at 0.92 ppm (Fig. 3B). Comparison with racemic material **30** then showed that the 4-methyl of the 2*S* diastereomer resonates at 0.98 ppm. The C-3 proton *anti* to the 5-methyl resonates at lower field (1.75 ppm) than the 3-*syn* proton (1.52 ppm, Fig. 3B, see ESI† for full details of assignment).

The 3-deuterated pantetheine product from the ER reaction, [3-^2^H]-**6P**, was hydrolysed and also coupled to 2*S*-methylmandelate to form the mandelate ester [3-^2^H]-**30**. This showed the presence of methyl doublets at 0.92 (*R*) and 0.98 (*S*) indicating that the compound was racemic at the 2-position as expected (Fig. 3C). The 2*R* product showed exclusive coupling between the 4-methyl resonance and a 3-*syn* proton, while the 2*S* product showed exclusive coupling between its 4-methyl resonance and a 3-*anti* proton (Fig. 3C). This shows that the 3-position possesses *R* configuration for both 2-epimers, indicating exclusive 3-*Re* hydride attack during the reduction.

The ER reaction was repeated using angelic pantetheine **8P** as the substrate. The same NMR analysis once again showed (Fig. 3D) the formation of a racemic product (methyl doublets at 0.92 and 0.98 ppm). In this case, however, the 4-methyl of the 2*R* diastereomer coupled to a 3-*anti* proton, while the 4-methyl of the 2*S* diastereomer coupled to a 3-*syn* proton, indicating formation of the 3*S* configuration for both 2-epimers from 3-*Si* hydride attack. The angelic pantetheine used contained some tigloyl pantetheine which arises from spontaneous and unavoidable isomerisation. The COSY NMR showed the formation of products corresponding to reaction of tigloyl pantetheine and integration of the COSY peaks showed this to account for 32% of the product (see ESI†).

### Model structure

Extensive efforts were made to obtain structural information on the ER domain. Protein crystals could be obtained, but these were not suitable for structural solution. As an alternative, a model was built using the Swiss-Model homology modeling server^18^ with the crystal structure of vertebrate fatty acid synthase (2vz9)^19^ serving as the template.

**Fig. 4:**
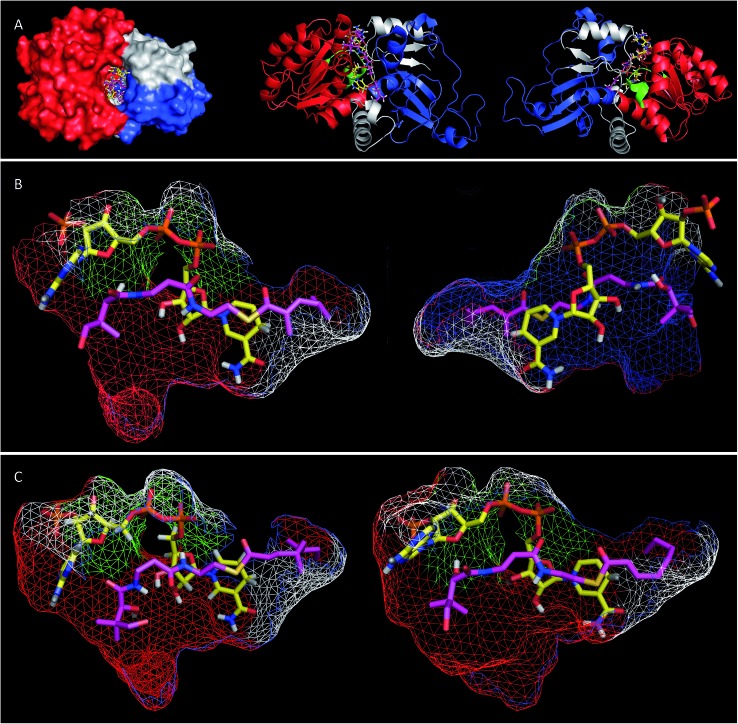
(A) View of the overall structure of the SQTKS ER domain with NADPH and the triketide substrate **15P** docked in the active site; (B) view of the active site of SQTKS ER model with **15P** docked. Left, view from substrate binding domain; right, view from cofactor binding domain; (C) tetraketides docked in the ER active site, left, octenoyl pantetheine **19P**; right, squalestatin tetraketide pantetheine **1P**. Both viewed from substrate binding domain. Colours: red, cofactor binding domain; blue, substrate binding domain; green, Rossmann fold; grey, C-terminal sequence; yellow, NADPH, magenta docked pantetheine substrate.

The model consists of three main structural features (Fig. 4A). The N-terminus (G1886–I2001, blue in Fig. 4) forms a globular domain which appears to be involved in contacting the acyl-pantetheine substrate. The central sequence V2002–V2144 (red/green in Fig. 4) forms a globular cofactor-binding domain and includes a canonical Rossmann fold (green in Fig. 4). Finally the C-terminal sequence (grey in Fig. 4) forms a link between the cofactor and substrate binding domains as well as part of a capping region above the active site. The model shows that the active site of the ER consists of an extended tunnel between the cofactor and substrate-binding domains, into which the NADPH cofactor extends, with the nicotinamide located deep (16 Å) inside the protein. The NADPH contacts one side of the tunnel made up from the N-terminal domain of the ER, making specific contacts with residues S2072 and K2055 (nucleotide 2′ phosphate), G2029 (diphosphate), I2119 and V2144 (nicotinamide amide). All these residues are conserved in other PKS and vFAS ER domains (see ESI†). Of the two nicotinamide 4′ hydrogens, the *pro-S* hydrogen faces the surface of the NADPH-binding domain and is unavailable for reaction. However, the nicotinamide 4′-*pro-R* hydrogen is exposed in agreement with the *in vitro* assay data which shows that this is transferred as hydride to the substrate. The active site tunnel broadens around the nicotinamide and extends past it, making an extended chamber formed by residues from the cofactor- and substrate-binding domains and the C-terminal sequence.

Pantetheine substrates were docked into the active site by manually placing them in approximate positions using PyMol.^20^ These initial poses were then minimised using the YASARA algorithm.^21^ In the case of the triketide pantetheine **15P** this docking procedure resulted in a model in which the pantetheine extends parallel to the adenine diphosphate, locating the thiolester and the β-carbon adjacent to the nicotinamide. The αβ-unsaturated carbonyl of the substrate adopts an s-*cis* conformation which places the reactive β-carbon 3.6 Å away from the cofactor's reactive 4′-*pro-R* hydrogen (Fig. 4B). The geminal dimethyl group of the pantetheine makes contacts with a hydrophobic surface created by C2097 and L2098 at the entrance to the pocket. The pantetheine chain then extends past a largely hydrophobic surface created by N1922, F1923 and I2001 towards a pocket which contains the substrate (*vide infra*). The terminal hydroxyl of the pantetheine is located at the entrance to the substrate tunnel suggesting that in the functional PKS the substrate and pantetheine adopt an extended conformation with the ACP in contact with the outer surface of the ER active site, allowing delivery of the αβ-unsaturated thiolester to the nicotinamide. The model suggests the formation of two hydrogen bonds between pantetheine and NADPH: the pantoate 2-hydroxyl hydrogen lies 2.5 Å from the adenine ribose furan oxygen; and the pantothenic acid NH lies 2.4 Å from nicotinamide ribonucleotide 3′ oxygen.

The stereochemical assays of the *in vitro* ER showed that it transfers hydride from NADPH to the *Re* face at C-3 of tigloyl pantetheine **5P**, and the model is consistent with this geometric constraint. After transfer of hydride a transient enol(ate) must be reprotonated at the 2-carbon. In the native protein this must happen stereoselectively on the *Re*-face of C-2 to provide the observed 2-*S*-stereochemistry. However the model does not show a likely proton source (*e.g.* tyrosine hydroxyl *etc.*) within 5 Å of the substrate 2-carbon.

Tetraketide pantetheine-bound substrates (such as **19P** and **1P**) were also docked using the same procedure. In the case of the non-methylated tetraketide **19P** the pantetheine, thiol and acyl groups were located in approximately the same positions as the triketide pantetheine **15P**, forming the same hydrogen bonds to NADPH itself, but the extended tail of the polyketide stretches more deeply into the core of the ER into a hydrophobic pocket formed by residues from the substrate binding domain and the C-terminal sequence (F1941, L1969, I2001, V2004, I2008, D2145, L2146, I2147, I2149 and F2157). The YASARA algorithm minimises both protein and substrate conformations and our results suggest that the active site of the ER can expand in this region to accommodate the longer substrates. However, for the longer compounds the αβ unsaturated moiety does not form the same s-*ci*s conformation as the fast substrate **15P**. Here the conformation is s-*trans* in the case of **19P**, or twisted out of conjugation for the dimethylated tetraketide **1P**. The dimethylated tetraketide is also pushed further towards the top of the active site pocket, and conformational changes occur in the pantetheine moiety. Thus the model suggests that additional bulk towards the tail of the polyketide, particularly branching, may prevent the substrate from reaching a productive conformation for rapid reduction.

## Discussion

*In vitro* studies of fungal HR-iPKS are beginning to elucidate their selectivities. For example Vederas and coworkers have recently described investigations of the selectivity of *C*-MeT and KR domains of the intact lovastatin nonaketide synthase (LNKS).^22^ LNKS has an inactive *cis*-ER domain, but biosynthesis of the lovastatin nonaketide requires the activity of a *trans*-acting ER known as LovC which is structurally different from *cis* ERs.^23^ Our work shows that the active *cis*-ER domain of SQTKS can be reconstituted *in vitro* as a stand-alone catalytic domain. The fungal HR-iPKS show end-to-end homology with vFAS,^10^ for which a crystal structure has been obtained.^19^ vFAS dimerizes *via* significant contacts between the ER and DH domains from the two separate monomers. It was therefore unsurprising to find that the isolated SQTKS ER exists as a dimer in solution.

Although we were unable to obtain a crystal structure of the isolated SQTKS ER domain, a model built computationally was consistent with numerous experimental observations. For example the structural domain organisation is consistent with that observed for other PKS and FAS ER proteins and docking of NADPH showed interactions with known cofactor binding residues and the correct 4′-hydrogen exposed for reaction. Likewise, docking of substrate pantetheines gave structures consistent with the observed stereochemistry of reduction at the substrate 3-carbon.

The ER was shown to be catalytically active, reducing the SNAC diketide **5S** in the presence of NADPH, but enoyl SNACs were generally poor substrates, and not amenable to the collection of meaningful kinetic data. Much faster reaction was demonstrated for the corresponding acyl pantetheine **5P**, and all the acyl pantetheine substrates were turned over more quickly than their SNAC homologues. Kinetic analysis of a range of different acyl pantetheine substrates showed that the ER is, in fact, tolerant of a wide range of different substrates, including compounds likely to be true intermediates such as **5** and **15**, but also compounds: with unnatural methylation patterns (*e.g.* **12**, **13** and **18**); unsubstituted at C-2 (*e.g.* **11**, **17**, **18**, **19**, **21** and **24**); with odd-carbon main chains (**17** and **18**); with longer main chains (*e.g.* **24** and **25**); and even *Z*-alkenes (**8**) and 2-ethyl substrates (*e.g.* **10** and **16**). Only the tetrasubstituted olefin **9** showed no turnover among the di-and triketides tested. Triketides are generally better substrates (higher *k*_cat_/*K*_M_ values) than diketides or tetraketides, and in fact the unnatural monomethylated triketide **12** is the best substrate tested by a significant factor. The 4*S*,6*S*-dimethyltetraketide **1** was not turned over at all, and compounds with similar structures such as **21** were also very poor substrates. Interestingly a dimethylated tetraketide substrate **22** which is racemic at the 4- and 6-positions showed some detectable substrate activity, indicating that the ER can slowly reduce stereoisomers of **1**, but not **1** itself. Linear pentaketides are also substrates and this suggests that the ER is uniquely sensitive to the 4*S*,6*S*-dimethyltetraketide **1**. Inhibition studies showed that the 4*S*,6*S*-dimethyltetraketide **1P** acts as an inhibitor of the ER, so it can clearly enter the active site of the enzyme like its close structural analogues, but it cannot be reduced. It thus appears that the SQTKS ER acts as a rather general catalyst, able to accommodate and reduce many enoyl species which are passed to it, but it exerts its ‘programming’ effect by its inability to reduce the final tetraketide substrate.

Our results show that pantetheine substrates are processed more effectively than simple SNACs. The model reveals that specific interactions between pantetheine and the enzyme and cofactor are present which favour pantetheine binding over SNAC. The model also explains how compounds such as *Z*, and 2-ethyl alkenes can fit into the active site as the substrate-binding pocket broadens beyond the reaction chamber. The substrate binding pocket eventually narrows, making the accommodation of methylated substrates more difficult. However non-methylated linear pentaketides are substrates.

**Scheme 5:**
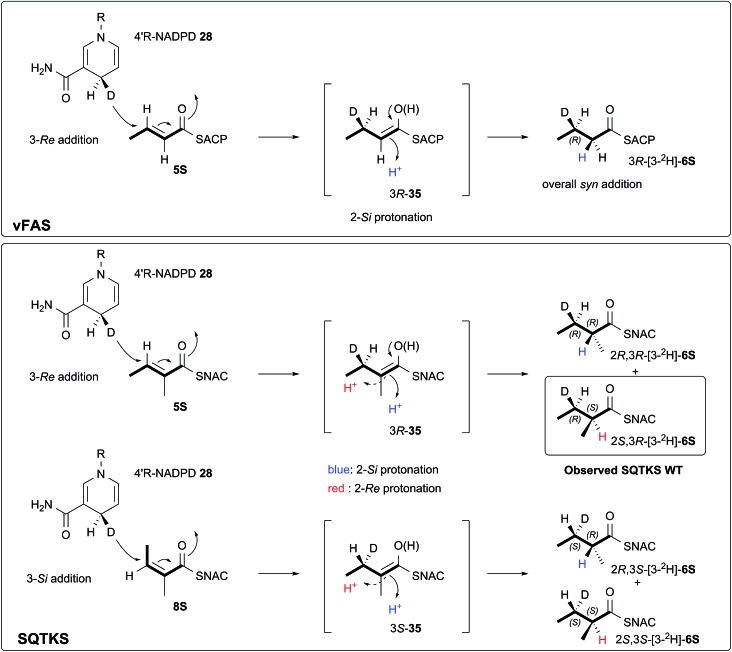
Stereochemical course of the reduction catalysed by the SQTKS isolated ER domain and its comparison with ER reduction by vFAS.

We also investigated the stereoselectivity of the isolated ER through the development of a new NMR-based assay. In intact SQTKS the ER domain sets the stereochemistry of the two methylated positions, presumably *via* a stereoselective reprotonation at the 2-position of an enol(ate) intermediate **35**, itself created by transfer of hydride to the 3-carbon of the enoyl substrate (Scheme 5). Surprisingly the results of two different assays showed that the isolated ER cannot control the stereoselectivity of reprotonation at the 2-carbon. In all assays, with both SNAC and pantetheine substrates, the product was always racemic at the 2-carbon, and control reactions showed that this was not caused by post-reduction racemisation or by the extraction and analysis procedures. We reasoned that this could be explained in two ways: either the substrate is unable to locate in the active site in a single conformation allowing uncontrolled addition of hydride and reprotonation; or the substrate does locate in a single conformation allowing stereoselective addition of hydride but the reprotonation step is uncontrolled. Highly stereoselective transfer of the 4′-*pro-R* hydrogen of NADPH was demonstrated, indicating that the cofactor must be rigidly located in the active site. Furthermore we showed that transfer of hydride to the 3-carbon of the diketide substrate is also highly stereoselective, indicating that the substrate must take a single conformation relative to NADPH. Surprisingly the ER is also able to reduce *Z*-configured alkenes. For the diketide angelic pantetheine **8P** the stereochemical assay again showed that hydride transfer to the 3-carbon is highly stereoselective. Analysis of the sense of hydride addition shows that the *Z*-alkene must bind in the active site in the same orientation as the *E*-alkene (Scheme 5).

Thus we can rule out a situation in which the substrate binds in alternative conformations in the active site. This leads to the conclusion that, for the isolated ER, reprotonation at the 2-carbon has become unselective. A model of the SQTKS ER with the cofactor and substrate docked is fully consistent with the experimental results. The model shows that NADPH binds into the active site in a conformation which presents its 4′-*pro-R* hydrogen towards the substrate such that reduction would occur at the *Re* face of the tigloyl pantetheine **5P** 3-carbon.

The stereochemistry of the ER reaction of FAS systems has been determined previously, and for different FAS systems all four possible modes of reduction have been observed. For vFAS the 4′-*pro-R* hydrogen of NADPH^24^ is transferred by *Re* addition at the substrate 3-carbon,^25^ and both features match our observation for SQTKS ER, reinforcing the similarity between the vFAS and HR-iPKS systems. vFAS then reprotonates stereoselectively at the *Si* face of the 2-position^26^ so that overall delivery of hydrogen occurs *syn* (Scheme 5). For native SQTKS the reprotonation at the 2-position must be opposite, giving the observed *anti* addition of hydrogen, presumably arising by alternative positioning of the proton donor. Vederas also showed that fungal FAS (fFAS) and the PKS responsible for cladosporin biosynthesis in *Cladosporium cladosporioides* have opposite protonation selectivities at the 2-position using *in vivo* isotopic labelling assays: fFAS reprotonates on the *Si* face; while the PKS reprotonates on the *Re* face.^27^ Fungal PKS domains have thus diverged from their FAS counterparts in this respect. However, it appears that the stereochemistry of 2-reprotonation is easily disturbed in the SQTKS ER, raising questions regarding the identity of the proton donor.

Examination of the ER model does not show a likely protein residue which could be responsible for reprotonation of the enol(ate) intermediate within 5 Å of the substrate 2-carbon. Ban and coworkers suggested that K1771 and/or D1797 of vFAS could be the residues responsible for the reprotonation step. In the SQTKS ER, K1771 is conserved (see ESI†), but the amino group is 9.7 Å distant from the reacting 2-carbon. D1797 is not conserved in the SQTKS sequence, although an aspartate is present as D1795 (vFAS numbering): the closest oxygen is 6.5 Å distant from the 2-carbon, and almost coplanar with the C1–C2 enol(ate) of the substrate and it may therefore be unable to protonate the 2-carbon.

Leadlay and coworkers have studied the reprotonation question in erythromycin (ery) ER4 and rapamycin (rap) ER13 domains.^28^ There was strong sequence-based evidence for residue 1584 (vFAS numbering) being involved in stereoselectivity at the 2-position. When this residue is Y in some modular PKS ER domains then 2*S*-configured products are formed (*Si* protonation), otherwise 2*R*-configured products are produced (*Re* protonation). This suggested that Y1584 might be responsible for protonation. However, vFAS ER has L instead of Y at this position, and it protonates the enolate *Si*. The SQTKS ER also has L at this position and it reprotonates *Re* in the WT PKS. Thus the picture is unclear. Furthermore, mutation V1584Y in rap ER13 did not cleanly change the selectivity from *R* to *S*. Other possible residues including N1573, D1576 and Y1657 as well as K1771 (vFAS numbering) were also proposed.^29^ However mutagenesis did not strongly support the role of any of these residues in controlling the stereoselectivity of the protonation step.

In the SQTKS ER these residues are also conserved, and the model shows that while N1573 is only 5.7 Å from the reacting 2-carbon, D1576, L1584 and Y1657 are further away (7.9, 8.8 and 11.7 Å respectively, vFAS numbering). However N1573 and D1576 are correctly placed to give protonation at the required *Re* face, while if position 1584 were Y it could protonate the *Si* face. Thus neither our model, or the suggestions of previous workers can adequately explain the source of the stereoselective reprotonation. Thus it may be that the proton is supplied by a judiciously placed water molecule, itself held in place by diverse residues. In the isolated SQTKS ER domain it is clear that the reprotonation has become unselective and we propose that removal of the other surrounding catalytic domains induces additional flexibility into the ER active site allowing ingress of excess water to both faces of the intermediate enol(ate) **35**, although further experiments will be required to verify this hypothesis.

## Conclusions

Overall our results show that the SQTKS ER is a broadly substrate-tolerant domain, with low intrinsic selectivity for diketides and triketides. It does not strictly reject tetraketides, allowing them into its active site and appearing to control the reduction by subtle conformational effects probably induced by the methylation pattern distil from the reactive carbons. The results differ from those of Vederas and coworkers^22^ who showed that the *C*-MeT domain of LNKS shows high substrate selectivity, but more closely match the conclusions for the LNKS KR domain which shows a lower level of selectivity.

These results reinforce a growing body of evidence which supports a programming mechanism for HR iPKS based upon *kinetic competition* by catalytic domains for individual substrates. In other words the programme decision at any point is not made by a single catalytic domain, but by the *relative rates* of two (or more) competing domains. Any given ACP-bound intermediate can be a substrate for two or more of the PKS catalytic domains. For example triketide olefin **15** could be a substrate for the ER or could be passed back to the KS for chain-extension by the AT. In this case the ER must react faster than the AT, producing a fully saturated product which can then only be a substrate for further chain extension. At the tetraketide stage **1** cannot be reduced by the ER and chain-release (by an as-yet undetermined mechanism) must be faster than AT/KS. Our results also suggest a role for sequestration of substrates by domains which are not catalytically active, for example **1** can enter the ER even though it is not reduced, and this may prevent the AT passing the substrate to the KS for further extension. The time substrates spend in non-reacting domains may also influence competition and thus programming. Current kinetic assays cannot yet probe these non-catalytic interactions.

This model of competition for substrates by different enzymes also offers an explanation for the observation that HR iPKS such as TENS and LNKS display reduced programming fidelity in different circumstances – the fact that domains such as ER and KR can posses broad substrate selectivity allows them to respond to unusual substrates without jamming the entire PKS. Such a mechanism could also allow the rapid evolution of new polyketides by a PKS through the accumulation of subtle selectivity changes in individual domains. The results offer possibilities for future engineering of HR iPKS systems – for example expansion of the active site of SQTKS ER may allow longer chains to be synthesised. Our work in this area currently focuses on obtaining more kinetic evidence for other isolated HR iPKS domains and the development of new methods for studying domain selectivity and competition.

## References

[cit1] Cox R. J. (2007). Org. Biomol. Chem..

[cit2] Chooi Y.-H., Tang Y. (2012). J. Org. Chem..

[cit3] Fischbach M. A., Walsh C. T. (2006). Chem. Rev..

[cit4] Maier T., Leibundgut M., Ban N., Leibundgut M., Maier T., Jenni S., Ban N. (2008). Science.

[cit5] Lin C. Y., Smith S. (1978). J. Biol. Chem..

[cit6] Cox R. J., Simpson T. J., Glod F., Hurley D., Nicholson T. P., Rudd B. A. M., Wilkinson B., Zhang Y., Skellam E. J., Hurley D., Davison J., Lazarus C. M., Simpson T. J., Cox R. J. (2004). Chem. Commun..

[cit7] Sidebottom P. J., Highcock R. M., Lane S. J., Procopiou P. A., Watson N. S. (1992). J. Antibiot..

[cit8] Bergstrom J. D., Kurtz M. M., Rew D. J., Amend A. M., Karkas J. D., Bostedor R. G., Bansal V. S., Dufresne C., VanMiddlesworth F. L., Hensens O. D. (1993). Proc. Natl. Acad. Sci. U. S. A..

[cit9] Bonsch B., Belt V., Bartel C., Duensing N., Koziol M., Lazarus C. M., Bailey A. M., Simpson T. J., Cox R. J. (2016). Chem. Commun..

[cit10] Fisch K. M., Bailey A. M., Bakeer W., Yakasai A. A., Song Z., Pedrick J., Wasil Z., Lazarus C. M., Simpson T. J., Cox R. J. (2011). J. Am. Chem. Soc..

[cit11] Zheng J., Gay D. C., Demeler B., White M. A., Keatinge-Clay A. T. (2012). Nat. Chem. Biol..

[cit12] Gaudelli N. M., Townsend C. A. (2013). J. Org. Chem..

[cit13] Sedgwick B., Morris C., Seyama Y., Kasama T., Yamakawa T., Kawaguchi A., Okuda S. (1980). J. Chem. Soc., Chem. Commun..

[cit14] Parker D. (1991). Chem. Rev..

[cit15] Fulwood R., Parker D. (1994). J. Chem. Soc., Perkin Trans. 2.

[cit16] Jeong S. S., Gready J. E., Ottolina G., Riva S., Carrea G., Danieli B., Buckmann A. F. (1994). Anal. Biochem..

[cit17] Frater G., Müller U., Günther W. (1984). Tetrahedron.

[cit18] Biasini M., Bienert S., Waterhouse A., Arnold K., Studer G., Schmidt T., Kiefer F., Cassarino T. G., Bertoni M., Bordoli L., Schwede T., Arnold K., Bordoli L., Kopp J., Schwede T., Kiefer F., Arnold K., Künzli M., Bordoli L., Schwede T., Guex N., Peitsch M. C., Schwede T. (2014). Nucleic Acids Res..

[cit19] Maier T., Leibundgut M., Ban N. (2008). Science.

[cit20] The PyMOL Molecular Graphics System, Version 1.7.4, Schrödinger, LLC.

[cit21] Krieger E., Joo K., Lee J., Lee J., Raman S., Thompson J., Tyka M., Baker D., Karplus K. (2009). Proteins.

[cit22] Cacho R. A., Thuss J., Xu W., Sanichar R., Gao Z., Nguyen A., Vederas J. C., Tang Y. (2015). J. Am. Chem. Soc..

[cit23] Ames B. D., Nguyen C., Bruegger J., Smith P., Xu W., Ma S., Wong E., Wong S., Xie X., Li J. W. H., Vederas J. C., Tang Y., Tsai S.-C. (2012). Proc. Natl. Acad. Sci. U. S. A..

[cit24] Dugan R. E., Slakey L. L., Porter J. W. (1970). J. Biol. Chem..

[cit25] Anderson V. E., Hammes G. G. (1984). Biochemistry.

[cit26] Saito K., Kawaguchi A., Seyama Y., Yamakawa T., Okuda S. (1981). J. Biochem..

[cit27] Rawlings B. J., Reese P. B., Ramer S. E., Vederas J. C. (1989). J. Am. Chem. Soc..

[cit28] Kwan D. H., Sun Y., Schulz F., Hong H., Popovic B., Sim-Stark J. C. C., Haydock S. F., Leadlay P. F. (2008). Chem. Biol..

[cit29] Kwan D. H., Leadlay P. F. (2010). ACS Chem. Biol..

