# Supplementary material for: Substrate selectivity of an isolated enoyl reductase catalytic domain from an iterative highly reducing fungal polyketide synthase reveals key components of programming
Source: Chem Sci. 2016 Sep 26;8(2):1116–26. doi: 10.1039/c6sc03496a (PMC5369529; doi:10.1039/c6sc03496a)
Supplement: Supplementary file 1 [file SC-008-C6SC03496A-s001.pdf]

Substrate Selectivity of an Isolated Enoyl Reductase Catalytic Domain from an Iterative Highly Reducing Fungal Polyketide Synthase Reveals Key Components of Programming

Douglas M. Roberts,[1,2] Christoph Bartel,[2] Alan Scott,[1] David Ivison,[1] Thomas J. Simpson[1] and Russell J. Cox*[1,2]

1. School of Chemistry, University of Bristol, Cantock's Close, Bristol, BS8 1TS, UK.

2. Institute for Organic Chemistry, Leibniz Universität Hannover, Schneiderberg 1B, 30167, Hannover, Germany.

* Corresponding Author: russell.cox@oci.uni-hannover.de

Electronic Supplementary Information

**1.0**	**Synthesis and characterisation**	**2**
**2.0**	**Production and purification of ER protein**	**55**
**3.0**	**Kinetic and Inhibition Assay procedures**	**58**
**4.0**	**Stereochemical assay procedures**	**68**
**5.0**	**Stereochemical Assignment of Proton Chemical Shifts of 2-Methyl Butyric Acid.**	**70**
**6.0**	**LCMS conditions**	**71**
**7.0**	**Multiple alignment of ER sequences**	**72**
**8.0**	**Modelling and Docking Procedures**	**73**
**9.0**	**Integration of Angelic Mandelate Spectrum**	**74**

1.0 Synthesis of substrates

All solvents were used as purchased unless otherwise stated apart from hexane, which was distilled before use. BuLi was titrated with diphenylacetic acid before use to confirm the concentration.

E-2-Ethylbut-2-enoic acid 10[1]

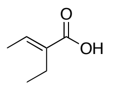

Chemical Formula: $C_6H_{10}O_2$
Exact Mass: 114.07

Potassium *tert*-butoxide (1.68 g, 15.0 mmol)was dissolved in Tetrahydrofuran (THF). The solution was cooled to 0 °C and 1 eq. triethyl 2-phosphonobutyrate was added under nitrogen atmosphere. After the solution was stirred for 10 min at 0 °C, acetaldehyde (0.9 ml, 15.00 mmol) was added and the reaction mixture was then stirred for 3 h at 22 °C. The mixture was added to H_2O, the organic phase was extracted (3 ×) with ethyl acetate, washed (1 ×) with a saturated NaCl solution and dried over $MgSO_4$. The solvent was removed under reduced pressure and the product was purified by column chromatography (eluent: ethyl acetate/hexane 1:10).

The ester was dissolved in a H_2O/EtOH mixture (1:5) and 10 eq. of KOH were added. The reaction mixture was stirred for 4 h at 80 °C. The solution was cooled, diethyl ether was added and the organic phase was washed (1 ×) with a saturated $NaHCO_3$ solution. The aqueous phase was acidified with aq. HCl (2 M) and the product was extracted (3 ×) with ethyl acetate. The solvent was removed *in vacuo*. The product **10** was a yellow oil (0.58 g, 5.08 mmol, 34 %). ^{1}H NMR (400 MHz, $CDCl_3$): δ_H = 1.02 (3 H, t, *J*HH = 7.5 Hz, 20-CH_3), 1.83 (3 H, d, *J*HH = 7.1 Hz, 18-CH_3), 2.32 (2 H, q, *J*HH = 7.5 Hz, 19-CH_2), 6.96 (1 H, q, *J*HH = 7.1 Hz, 17-CH) ppm. ^{13}C NMR (100 MHz, $CDCl_3$): δ_C = 13.6 (20-CH_3), 13.9 (18-CH_3), 19.6 (19-$CH_{2)}$, 134.3 (16-C), 139.6 (17-CH), 171.3 (15-CO), ppm. IR: νM_{ax} = 2969 (w), 2937 (w), 2877 (w), 2656 (w), 1680 (vs), 1640 (s), 1417 (w), 1375 (w), 1290 (m), 1255 (s), 1163 (w), 1097 (w), 1072 (w), 1037 (w), 940 (w), 861 (w), 777 (w), 751 (w), 638 (w) cm^{-1}.

E-Ethyl 2-methyl hex-2-enoate 12a[2]

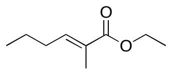

To a stirred solution of butryaldheyde (418 ml, 5.8 mmol) in dry CH_2Cl_2 (6 ml) was added (Carbethoxycarbonylethylidene)triphenylphosphorane (2.1 g, 5.8 mmol). This was refluxed overnight then allowed to cool to room temperature. Hexane (7 ml) was added and this was stirred for 10 mins and then filtered. The filtrate was concentrate in *vacuo* to approximately 5 ml and then hexane (7 ml) was added and stirred for a further 10 mins. This was filtered and then concentrated in *vacuo*. The resulting oil was purified by column chromatography (hexane:ethyl acetate 95:5) to yield the desired product (yellow oil, 690 mg, 4.4 mmol, 76%, Rf 0.21, hexane:ethyl acetate 95:5); δ_H (400 MHz, $CDCl_3$) 6.76 (1H, tq *J* 7.6 1.5, H-3), 4.18 (2H, q *J* 7.1, H2-8), 2.18-2.11 (2H, m, H2-4), 1.85 (3H, dt *J* 1.4 0.9, H3-7), 1.52-1.42 (2H, m, H2-5), 1.29 (3H, t *J* 7.1, H3-9), 0.93 (3H, t *J* 7.4, H3-6); δ_C (100 MHz, CDCl3) 168.5 (C-1), 142.3 (C-3), 128.0 (C-2), 60.5 (C-8), 30.8 (C- 4), 22.0 (C-5), 14.4 (C-7), 14.0 (C-9), 12.5 (C-6); MS (ES+): *m/z* (%) 179 (4, [M]Na+), 157 (100, [M]H+), 129 (78, [M + H - CH_2CH_3]+).

***E*-2-methyl hex-2-enoic acid 12**[2]

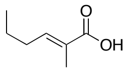

Chemical Formula: $C_7H_{12}O_2$
Exact Mass: 128.08

To a stirred solution of *E*-ethyl-2-methyl hex-2-enoate (**12a**, 125 mg, 0.8 mmol) in THF (2 ml) was added an aqueous NaOH (2M, 4 ml). The reaction was refluxed overnight and on completion this was acidified with aqueous HCl (2M) to pH 4 and then extracted with diethyl ether (3 × 7 ml). The organic fractions were combined, dried ($MgSO_4$) and concentrate in *vacuo* to yield the desired product (yellow oil, 75 mg, 0.6 mmol, 78%); δ_H (400 MHz, $CDCl_3$) 6.92 (1H, dq *J* 7.5 1.5, H-3), 2.21-2.16 (2H, m, H_2-4), 1.85-1.82 (3H, m, H_3-7), 1.49 (2H, tq *J* 7.4 7.3, H_2-5), 0.94 (3H, t *J* 7.4, H_3-6).

***E*-4*RS*-4-methyl hex-2-enoic acid 13**[3]

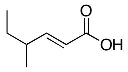

Chemical Formula: $C_7H_{12}O_2$
Exact Mass: 128.08

2-methyl butyraldehyde (1.6 g, 19.0 mmol), malonic acid (1.2 g, 12.0 mmol), pyridine (3.8 ml) and morpholine (18.8 μl) were stirred at room temperature for 24 h. The temperature was then raised to 115 °C and the reactions was stirred for a further 17 h. On completion the reaction was poured into aqueous NaOH (1M, 10 ml) and extracted with diethyl ether (3 × 10 ml) the aqueous layer was then acidified with aqueous HCl (2M) and extracted with diethyl ether (5 × 10 ml). The organics from the acidified extractions were combined, washed with water (25 ml), dried ($MgSO_4$) and concentrated in *vacuo* to yield the desired product (yellow oil, 738 mg, 5.8 mmol, 48%); δ_H (400 MHz, $CDCl_3$) 10.95 (1H, br s, H-8), 6.97 (1H, dd *J* 15.7 7.8, H-3), 5.79 (1H, dd *J* 15.7 1.2, H-2), 2.28-2.21 (1H, m, H-4), 1.45-1.38 (2H, m, H_2-5), 1.05 (3H, d *J* 6.6, H_3- 7), 0.88 (3H, t *J* 7.7, H_3-6); δ_C (100 MHz, $CDCl_3$) 172.1 (C-1), 157.1 (C-3), 119.4 (C-2), 38.3 (C-4), 28.8 (C-5), 18.9 (C-7), 11.7 (C-6); υM_{ax} (oil)/cm^{-1} 2965, 2931, 2871.

***E*-Ethyl 2,4 dimethylhex-2-enoate 14a**[4]

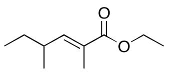

To a stirred solution of 2-methylbutryaldehyde (500 mg, 5.8 mmol) in dry CH_2Cl_2 (6ml) was added (Carbethoxyethylidene)triphenylphosphorane (2.1g, 5.8mmol). This reaction was refluxed overnight and then allowed to cool to room temperature. Hexane (7 ml) was added and this was stirred for 10 mins and then filtered to remove the precipitate that had formed. This was concentrate in *vacuo* to approximately 5 ml and then hexane (7 ml) was added and stirred for a further 10 mins and then filtered and concentrated in *vacuo*. The resulting oil was purified by column chromatography (hexane:ethyl acetate 95:5) to yield the desired product (yellow oil, 441 mg, 2.6 mmol, 45%, Rf 0.24, hexane:ethyl acetate 95:5); δ_H (300 MHz, $CDCl_3$) 6.52 (1H, dq *J* 7.2 1.4, H-3), 4.18 (2H, q *J* 7.1, H2-9), 2.47-2.32 (1H, m, H-4), 1.83 (3H, d *J* 1.4, H3-8), 1.47-1.33 (2H, m, H2-5), 1.29 (3H, t *J* 7.1, H3-10), 0.99 (3H, d *J* 6.7, H3-7) 0.85 (3H, t *J* 7.4, H3-6); δ_C (75 MHz, $CDCl_3$) 168.6 (C-1), 148.0 (C-3), 126.6 (C-2), 60.5 (C-9), 35.0 (C-4), 29.7 (C-5)), 19.8 (C-7), 14.4 (C-10), 12.6 (C-8), 12.0 (C-6); MS (ES+): *m/z* (%) 193 (4, [M]Na+), 171 (100, [M]H+), 143 (84, [M + H - CH_2CH_3]+).

E-4*RS*-2,4-Dimethylhex-2-enoic acid 14[4]

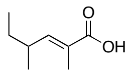

Chemical Formula: $C_8H_{14}O_2$
Exact Mass: 142.10

To a solution of ethyl *E*-4*RS*-2,4-dimethylhex-2-enoate (**14a**, 544 mg, 3.2 mmol) in THF (4 ml) was added aqueous NaOH (2M, 5 ml) and the reaction was refluxed for 4 h. The aqueous layer acidified to pH 4 with aqueous HCl (2M) and extracted with diethyl ether (3 × 10 ml). The organics were combined and washed with water (10 ml) and brine solution (10 ml). The organics were then dried ($MgSO_4$) and concentrated in *vacuo* to yield the desired product (yellow oil, 338 mg, 2.4 mmol, 74%); δ_H (300 MHz, $CDCl_3$) 7.49 (1H,br s, H-9), 6.69 (1H, dq *J* 7.2 1.4, H-3), 2.50-2.34 (1H, m, H-4), 1.84 (3H, d *J* 1.5, H_3-7), 1.37-1.15 (2H, m H_2-5), 1.01 (3H, d *J* 6.6, H_3-8), 0.86 (3H, t *J* 7.5, H_3-6); δ_C (75 MHz, $CDCl_3$) 173.8 (C-1), 150.9 (C-3), 125.9 (C-2), 35.2 (C-4), 29.7 (C-5), 19.7 (C-7) 12.3 (C-8), 12.0 (C-6); MS (ES +): *m*/*z* (%) 165 (6, [M]Na+), 143 (100, [M]H+).

E-4*S*-*2,4*-Dimethylhex-2-enoic acid 15[5]

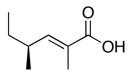

Chemical Formula: $C_8H_{14}O_2$
Exact Mass: 142.10

2*S*-2-methylbutanol (1.00 ml, 10 mmol), pyridinium chlorochromate (2.2 g, 10 mmol) and celite (1.31 g) were dissolved in THF (30 ml). The reaction was followed by TLC and after full consumption of the alcohol (carbethoxyethylidene)triphenylphosphorane (4.20 g, 12.00 mmol) was added and the reaction heated under reflux for 7h. Diethyl ether was added and the mixture was filtered through Florisil. The filtered solution was concentrated *in vacuo* and the residue purified by column chromatography (hexane/ ethyl acetate, 4:1) to give (2E,*4*S)-methyl-*2,4*-dimethylhex-2-enoate (0.40 g, 3.40 mmol) which was directly hydrolysed. To the ester in ethanol/water 5:1 (5 ml/ 1 ml) was added potassium hydroxide (1.22 g, 22.00 mmol). After stirring under reflux for 3 h diethyl ether was added. The mixture was washed with $NaHCO_3$ (3 × 10 ml). Then the aqueous layer was acidified with 2M HCl until pH 1 and extracted with ethyl acetate (3 × 10 ml). The organic layer was dried over $MgSO_4$ and concentrated i*n vacuo*. The obtained product was a red oil (0.57 g, 3.30 mmol, 98 %). ^{1}H-NMR ($CDCl_3$, 400 MHz): δ_H 0.88 (t, *J* = 7.4 Hz, 6H, CH_3); 1.17 (d, *J* = 6.9 Hz, 3H, CH_3); 1.40-1.60 (m, 2H, CH_2); 1.85 (d, *J* = 1.5 Hz, 3H, 6-CH_3); 2.32-2.49 (m, 1H, 3-CH), 6.69 (dd, *J* = 1.5 Hz, *J* = 10.0Hz, 1H, 4-CH). ^{13}C-NMR ($CDCl_3$, 100 MHz): δ_C 11.9 (1-CH_3), 12.6 (5-CH_3), 19.7 (6-CH_3), 29.7 (2-CH_2), 34.9 (3-CH), 126.6 (7-C), 147.9 (4-CH), 168.4 (8-CO); ESMS, *m/z* (%): 143 $[M]H^+$(100 %); $[a]_D$= + 44.0 (CH_2Cl_2) [lit[6] +44.4 CH_2Cl_2]; ν_{max}/ cm^{-1} 2966 (-C-H), 2935 (-C-H), 2877 (-C-H), 1697 (C=O), 1463 (CO), 1382 (C-O), 1278 (C-O).

E-2-ethyl-2-hex-2-enoic acid 16[7]

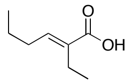

Chemical Formula: $C_8H_{14}O_2$
Exact Mass: 142.10

Potassium tert-butoxide (1.63 g, 14.5 mmol) was added to dry THF (20 ml) and cooled to 0 °C. Triethylphosphonobutyrate (3.5 ml, 14.5 mmol) was added and the solution stirred for 10 min. Butyraldehyde (14 mmol) was added and the mixture warmed up to room temperature. The solution was stirred for 3 h and then water (20 ml) was added and extracted with EtOAc (3 × 20 ml). The combined organic layers were washed with brine and dried

over $MgSO_4$. The crude product was purified by column chromatography (ethyl acetate/ hexane 1:10). The mixture of the *E*- and *Z*-isomers was a colourless oil (1.50 g, 8.80 mmol, 63 %). R_f: 0.69 (ethylacetate/ hexane 1:10). ^{1}H-NMR ($CDCl_3$, 400 MHz): δ_H 0.90-1.05 (m, 6H, 1-10-CH_3); 1.28-1.32 (m, 3H, 8-CH_3); 1.38-1.52 (m, 3H, 2-CH_2); 2.16 (q, *J* = 6.9 Hz, 2H, 3-CH_2); 2.24-2.40 (m, 2H, 9-CH_2); 4.16-4.23 (m, 2H, 7-CH_2); 5.83 (t, *J* = 7.9 Hz, 0.3 H, 4-CH); 6.71 (t, *J* = 7.4 Hz, 0.7 H, 4-CH). ^{13}C-NMR ($CDCl_3$, 100 MHz): δ_C 13.6 (1-CH_3), 13.9 (10-CH_3), 14.3 (8-CH_3), 20.0 (9-CH_{2E}), 22.1 (4-CH_{2E}), 22.7 (9-CH_{2Z}), 27.3 (2-CH_{2Z}), 30.4 (3-CH_{2E}), 31.5 (3-CH_{2Z}), 59.8 (7-CH_{2Z}), 60.3 (7-CH_{2E}), 133.9 (5-C_Z), 134.1 (5-C_e), 140.0 (4-CH_Z), 141.4 (4-CH_E), 168.0 (6-C_Z), 168.4 (6-C_E); ESMS, *m/z* (%): 184 [M - CH_2CH_3 + CH_3CN]H^+(18 %), 151 [M]H^+(8 %), 143 [M - CH_2CH_3]H^+(100 %); ν_{max} = 2963 (-C-H), 2935 (-C-H), 2874 (-C-H), 1709 (C=O), 1646 (-C=C), 1462 (CO), 1379 cm^{-1}.

To a solution of the ester in ethanol/water 5:1 (5 ml/ 1 ml) was added potassium hydroxide. After stirring under reflux for 3 h diethyl ether was added. The mixture was washed with $NaHCO_3$ (3 × 10 ml). Then the aqueous layer was acidified with 2M HCl until pH 1 and extracted with ethyl acetate (3 x 10 ml). The organic layer was dried over $MgSO_4$ and concentrated *in vacuo*. The obtained product was a red oil (1.2 g, 8.40 mmol, 96 %). ^{1}H-NMR ($CDCl_3$, 400 MHz): δ_H 0.91-1.08 (m, 6H, 1-8-CH_3); 1.40-1.54 (m, 2H, 2-CH_2); 2.20 (q, *J* = 7.7 Hz, 2H, 3-CH_2); 2.26-2.34 (m, 2H, 7-CH_2); 6.03 (t, *J* = 7.6 Hz, 1H, 4-CH_Z); 6.88 (t, *J* = 7.6 Hz, 1H, 4-CH_E). ^{13}C-NMR ($CDCl_3$, 100 MHz): δ_C 13.8 (1-CH_3), 13.8 (8-CH_3), 19.7 (7-CH_{2Z}), 22.0 (7-CH_{2E}), 27.4 (2-CH_2), 30.5 (3-CH_{2Z}), 31.7 (3-CH_{2E}), 132.6 (5-C_Z), 133.3 (5-C_E), 144.8 (4-CH_Z), 144.9 (4-CH_E), 173.3 (6-CO). ESMS *m/z* (%) 184 [M + CH_3CN]H^+(18 %), 143 [M]H^+(100 %); IR ν_{max} 2963 (-CH), 2934 (-CH), 2874 (-CH), 1681 (C=O), 1637 (-C=C), 1458 (CO), 1380 (C-O), 1291 (C-O) cm^{-1}.

E-Hept-2-enoic acid 17[8]

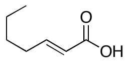

Chemical Formula: $C_7H_{12}O_2$
Exact Mass: 128.08

Valeraldhyde (1.6 g, 19.0 mmol), malonic acid (1.2 g, 12.0 mmol), pyridine (3.8 ml) and morpholine (18.8µl) were stirred at room temperature for 24 h. The temperature was raised to 115 °C and refluxed for 17 h. On completion the reaction was poured into aqueous NaOH (1M, 20 ml) and extracted with diethyl ether (5 × 20 ml). The aqueous phase was then acidified with aqueous HCl solution (2M, 15 ml) and extracted with diethyl ether (3 × 20 ml). The organic fractions from the extraction of the acidified aqueous phase were combined, washed with water (25 ml), dried ($MgSO_4$) and concentrated in *vacuo*. This crude product was purified to by Kugelrohr distillation to give the desired product (pale yellow oil, 685 mg, 5.3 mmol, 44%, 140- 150 °C, 11 mmHg); δ_H (400 MHz, $CDCl_3$) 7.09 (1H, dt *J* 15.6 7.0, H-3), 5.82 (1H, dt *J* 15.6 1.6, H-2), 2.33 (2H, tdd *J* 7.0 7.0 1.6, H_2-4), 1.49-1.41 (2H, m, H_2-5), 1.38-1.31 (2H, m, H_2-6), 0.91 (3H, t *J* 7.3, H_3-7); δ_C (100 MHz, $CDCl_3$) 172.3 (C-1), 152.7 (C-3), 120.7 (C-2), 32.2 (C-4), 30.1 (C-5), 22.4 (C-6), 13.9 (C-7); MS (ES-): *m/z* (%) 127 (100, [M - H]-), 83 (15, [M - COOH]-).

E-6-Methylhept-2-enoic acid 18[9]

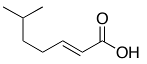

Chemical Formula: $C_8H_{14}O_2$
Exact Mass: 142.10

Pyridinium chlorochromate (1.1 g, 5.0 mmol) and Celite (1.3 g) were stirred in THF (30 ml). 4-Methyl pentanol (535 µl, 3.6 mmol) was added to the reaction, which was followed by TLC. After full consumption of the alcohol (1-2 h) methyl(triphenylphosphoranylidene)acetate (2.0g 6.1 mmol) was added and the reaction was refluxed for 7 h. On completion the reaction was cooled to RT, diethyl ether (20 ml) was added and the suspension was filtered through Florisil. The filtrate was then concentrated in *vacuo* and the resulting oil was purified by column chromatography (hexane:ethyl acetate 8:2) to yield the methyl ester (yellow oil, Rf 0.54, hexane: ethyl acetate 8:2): δ_H (400 MHz, $CDCl_3$); 6.97 (1H, dt *J* 15.7 7.0, H-3), 5.87 (1H, dt *J* 15.6 1.6, H-2), 3.72 (3H, s, H_3-OMe), 2.24-2.17 (2H, m, H_2-4),

1.62-1.54 (1H, m, H-6), 1.36-1.31 (2H, m, H_2-5), 0.89 (6H, d *J* 6.6, H6-7). The resulting oil was dissolved in aqueous NaOH (6M, 3.0 ml) and THF (3.5 ml) and the solution was refluxed for 5 h. On completion the reaction mixture was acidified with HCl solution (2M) to pH 3 and extracted with diethyl ether (3 × 30 ml). The organics were dried ($MgSO_4$) and concentrated in *vacuo,* which gave the desired product (a pale yellow oil, 243 mg, 1.7 mmol, 46%, Rf 0.58, ethyl acetate:hexane 1:1); δ_H (200 MHz, $CDCl_3$); 7.08 (1H, dt *J* 15.6 6.9, H-3), 5.83 (1H, dt *J* 15.6 1.5, H-2), 2.30-2.17 (2H, m, H_2-4), 1.68-1.48 (1H, m, H-6), 1.44-1.31 (2H, m, H_2-5), 0.90 (6H, d *J* 6.5, H6-7); δ_C (100 MHz, $CDCl_3$); 171.3 (C-1), 152.8 (C-3), 120.4 (C-2), 37.0 (C-5), 30.4 (C-4), 27.7 (C-5), 22.5 (C-7); MS (ES-): *m/z* (%) 283 (73, [M_2 - H]-), 141 (100, [M - H]-).

_E_-Ethyl 2-methyloct-2-enoate 20a[10]

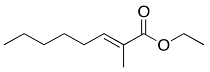

To a stirred solution of hexanal (800 mg, 8.0 mmol) in dry CH_2Cl_2 (9 ml) was added (Carbethoxyethylidene)triphenylphosphorane (2.1 g, 8.0 mmol). The reaction mixture was refluxed overnight and then allowed to cool to RT. Then pentane (15 ml) was added and this was stirred for 15 mins. The formed suspension was filtered and concentrated in *vacuo*, which formed an orange brown solid and pentane (20 ml) was added. The suspension was stirred vigorously for 20 mins and then this was then filtered and concentrated in *vacuo*. The resulting oil was purified by column chromatography (hexane:ethyl acetate, 1:1) to yield the desired product. (yellow oil, 696 mg, 3.8 mmol, 48%, Rf 0.65, hexane:ethyl actetate 1:1); δ_H (300 MHz, $CDCl_3$) 6.76 (1H, tq *J* 7.5 1.5, H-3), 4.18 (2H, q *J* 7.1, H2-10), 2.19-2.12 (2H, m, H2-4), 1.82 (3H, dt *J* 1.5 1.0, H3-9), 1.47-1.40 (2H, m, H2-5), 1.32-1.25 (7H, m, H2-6 H2-7 H3-11), 0.89 (3H, t *J* 7.1, H3-8); δ_C (100 MHz, $CDCl_3$), 168.5 (C-1), 142.6 (C-3), 127.8 (C-2), 60.5 (C-10), 31.7 (C-6), 28.8 (C-4), 28.4 (C-5), 22.7 (C-7), 14.4 (C-11), 14.1 (C-9), 12.5 (C-8); MS (ES+): *m/z*(%) 207 (4, [M]Na+), 185 (44, [M]H+), 157 (100), 139 (6, [M + H - $CO_2HCH_2CH_3$]+) 87 (49).

_E_-2-methyloct-2-enoic acid 20[11]

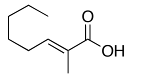

Chemical Formula: $C_9H_{16}O_2$
Exact Mass: 156.12

A solution of *E*-ethyl-2-methyloct-2-enoate (**20a**, 500 mg, 2.7 mmol) in a solution of THF (7.5 ml) and aqueous NaOH (6M, 6 ml) was refluxed for 4 h. On completion this was acidified with aqueous HCl (2M) to pH 4 and then extracted with diethyl ether (3 × 30 ml). The organic fractions were dried ($MgSO_4$) and concentrated in *vacuo*. The resulting oil was purified by bulb-to-bulb distillation to yield the desired product. (colourless oil, 160 mg, 1.0 mmol, 35%, bp 130-140 °C 7 mmHg); δ_H (400 MHz, $CDCl_3$) 6.92 (1H, tq *J* 7.5 1.5, H-3), 2.23-2.16 (2H, m, H_2-4), 1.83 (3H, dt *J* 1.4 0.9, H_3- 9), 1.49-1.42 (2H, m, H_2-5), 1.34-1.27 (4H, m, H_2-6 and H_2-7), 0.89 (3H, t *J* 6.9, H_3-8); δ_C (100 MHz, $CDCl_3$), 173.9 (C-1), 145.6 (C-3), 127.0 (C-2), 31.9 (C-6), 29.1 (C-4) 27.9 (C-5), 22.7 (C-7) 14.5 (C-9) 12.1 (C-8); MS (ES-): *m/z* (%) 155 (100, [M - H]-).

_E_-7-Methyloct-2-enoic acid 21[12]

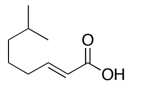

Chemical Formula: $C_9H_{16}O_2$
Exact Mass: 156.12

Pyridinium chlorochromate (1.3 g, 6.4 mmol) and Celite (1.4 g) were stirred in THF (40 ml). To this was added 5-methyl hexanol (608 μl, 4.3 mmol) and the reaction was followed by TLC. After full consumption of the alcohol (1-2 h)

methyl(triphenylphosphoranylidene)acetate (2.4 g, 7.3mmol) was added and the reaction was refluxed for 7 h. On completion the reaction was cooled to RT, diethyl ether (20 ml) was added and the resulting suspension was filtered through Florisil. The filtrate was then concentrated in *vacuo* and further purified by column chromatography (hexane:ethyl acetate 8:2) to yield the methyl ester (yellow oil, Rf 0.57, hexane: ethyl acetate 8:2); δ_H (400 MHz, $CDCl_3$); 6.97 (1H, dt *J* 15.6 7.0, H-3), 5.82 (1H, *J* 15.6 1.6, H-2), 3.72 (3H, s, H_3-OMe), 2.18 (2H, dtd *J* 7.2 7.2 1.6, H_2-4) 1.56-1.41 (3H, m, H_2-5 H-7), 1.23-1.16 (2H, m, H_2-6), 0.87 (6H, d *J* 6.6, H6-8). The resulting oil was dissolved in aqueous NaOH (6M, 3.0 ml) and THF (3.5 ml) and was refuxed for 5 h. On completion the reaction was acidified to pH 2 with aqueous HCl (2M) and then extracted with diethyl ether (3×30 ml). The organics were dried ($MgSO_4$) and concentrated in *vacuo,* this gave the desired product (yellow oil, 490 mg, 3.2 mmol, 73%, Rf 0.65, ethyl acetate:hexane, 1:1): δ_H (400 MHz, $CDCl_3$); 7.09 (1H, dt *J* 15.6 6.9, H-3), 5.82 (1H, dt *J* 15.6 1.6, H-2), 2.22 (2H, dtd *J* 7.2 7.2 1.6, H_2-4), 1.58-1.44 (3H, m, H_2-5 H-7), 1.24-1.16 (2H, m, H_2-6), 0.88 (6H, d *J* 6.6, H6-8); δ_C (100 MHz, $CDCl_3$); 171.7 (C-1), 152.6 (C-3), 120.6 (C-2), 38.5 (C-6), 32.7 (C-4), 30.0 (C-7), 25.9 (C-5), 22.7 (C-8); MS (ES-): *m/z* (%) 311 (100, [M_2 - H]-), 155 (82, [M - H]-).

2*RS*,4*RS*-2,4-dimethyl hexanal 22a[13]

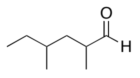

2-Butene (3.4 g, 60 mmol) was bubbled through dry THF (10 ml) at -78 °C for 5 min. Borane.THF (1M, 20 ml, 20 mmol) was added dropwise over 10 min to the reaction and after the addition was complete the reaction was warmed to -10 °C and stirred for 4 h. Methacrolein (2.1 g, 30 mmol) was then added in a dropwise manner and the reaction mixture was stirred for 30 min at RT. The reaction was quenched with water (1.5 ml) and then stirred overnight under N2 to remove excess of butene. Volatiles were removed in *vacuo* and the resultant yellow oil was purified by bulb-to-bulb distillation to yield the desired product an inseparable mixture of diastereomers (A and B 3:2, colourless oil, 1.5 g, 12 mmol, 62%, bp 45 °C/ 8 mmHg lit169 50 °C/ 13 mmHg); δ_H (400 MHz, $CDCl_3$); 9.61 (0.4H, d *J* 2.0, H-1A), 9.58 (0.6H, d *J* 2.6, H-1B), 2.48-2.36 (1H, m, H-2), 1.72 (1H, m, H-3), 1.58-1.48 (1H, m, H-4), 1.45-1.33 (2.H, m, H_2-5), 1.18-1.10 (1H, m, H-3), 1.09-1.06 (3H, m, H_3-8), 0.90-0.86 (6H, m, H_3-6 H_3-7); δ_C (100 MHz, $CDCl_3$); 205.7 (C-1A), 205.6 (C-1B), 44.4 (C-2B), 44.3 (C-2A), 38.0 (C-3A), 37.4 (C- 3B), 32.1 (C-4A), 31.9 (C-4B), 30.0 (C-5B), 29.3 (C-5A), 19.5 (C-7a), 18.9 (C-7B), 14.3 (C-8A), 13.5 (C-8B), 11.4 (C-6B), 11.3 (C-6A); υM_{ax} (oil)/cm^{-1} 2961 (C-H), 2929 (C-H), 2876 (C-H), 2740 (C-H aldehyde), 1726 (C=O), 1461, 1378.

***E*-4*RS*,6*RS*-4,6-dimethyl oct-2-enoic acid 22**[14]

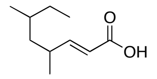

Chemical Formula: $C_{10}H_{18}O_2$
Exact Mass: 170.13

2*RS*,4*RS*-2,4-dimethyl hexanal (**22a**, 260 mg, 2.0 mmol), malonic acid (789 mg, 1.3 mmol), pyridine (2.4 ml) and morpholine (12 µl) were stirred at room temperature for 17 h. The reaction was refluxed at 115 °C for 17 h. On completion the solution was slowly poured into cooled aqueous NaOH (1M, 5 ml) and then extracted with diethyl ether (5 × 10 ml). After the extractions were complete the aqueous layer was acidified with aqueous HCl (2M) to pH 4, and a white suspension was formed. To this was added diethyl ether (10 ml) and the aqueous was extracted (2 × 10 ml). The organic extracts of the acidified aqueous extraction were combined, dried ($MgSO_4$) and concentrated in *vacuo*. This yielded the desired product as an inseperable mixture of diastereomers (A:B 3:2, colourless oil, 188 mg, 1.1 mmol, 84%); δ_H (400 MHz, $CDCl_3$); 6.98 (0.6H, dd *J* 15.6 7.8, H-3A), 6.92 (0.4H, *J* 15.7 8.4, H-3B), 6.80-6.74 (1H, m, H-2), 2.47-2.38 (1H, m, H-4), 1.42-1.09 (7H, m, H_2-5 H-6 H_2-7), 1.02 (3H, t *J* 6.9, H_3-10), 0.87-0.81 (6H, m, H_3-8 H_3-9); δ_C (100 MHz, $CDCl_3$); 172.4 (C-1A), 172.4 (C-1B), 158.3 (C-3A), 157.9 (C-3B), 119.1 (C-2B), 118.7 (C-2A), 43.4 (C-5B), 43.1 (C-5A), 34.5 (C-4B), 34.3 (C- 4A), 32.1 (C-6B), 31.9 (C-6A), 29.9 (C-7B), 29.4 (C-7A), 20.3 (C-10B), 19.3 (C-9A), 19.2 (C-9B), 19.0 (C-10A), 11.3 (C-8B), 11.2 (C-8A); MS (ES-): *m/z* (%) 339 (14, [M_2 - H]-), 169 (100, [M - H]-).

***4S,6S-E*-4,6-dimethyloct-2-enoic acid 1**[15]

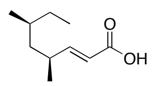

Chemical Formula: $C_{10}H_{18}O_2$
Exact Mass: 170.13

To a solution of squalestatin S1 (1.00 g, 1.40 mmol) in ethanol/water 5:1 (5 ml/ 1 ml) potassium hydroxide (1.22 g, 22.0 mmol) was added. After stirring under reflux for 3 h, diethyl ether was added to the solution and the mixture was washed (3 × $NaHCO_3$, 10 ml). The aqueous layer with the (*4*S,*6*S)-*2,4*-dimethyloct-2-enoic acid was acidified with 2M HCl until pH 1 and extracted with ethyl acetate (3 × 10 ml). The organic layer was dried over $MgSO_4$ and concentrated *in vacuo*. The obtained product was not purified further and was isolated was an oil (0.21 g, 0.12 mmol, 86 %).

***E*-Dec-2-enoic acid 24**[8]

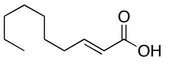

Chemical Formula: $C_{10}H_{18}O_2$
Exact Mass: 170.13

Octanal (1.2g, 9.5mmol), malonic acid (0.8g, 6.0mmol), pyridine (1.9 ml) and morpholine (9.4 µl) were stirred at room temperature for 24 h. The temperature was raised to 115 °C and refluxed for 17 h. On completion the reaction mixture was poured into aqueous NaOH (1M, 20 ml) and extracted with diethyl ether (5 × 20 ml). The aqueous phase was then acidified with aqueous HCl (2M, 15 ml) and extracted with diethyl ether (3 × 20 ml). The organic fractions from the extraction of the acidified aqueous phase were combined, washed with water (25 ml), dried ($MgSO_4$) and concentrated in *vacuo* to give the desired product (pale yellow oil, 652 mg, 3.8 mmol, 64%); δ_H (400 MHz, $CDCl_3$); 7.08 (1H, dt *J* 15.6 7.0, H-3), 5.81 (1H, dt *J* 15.6 1.6, H- 2), 2.26-2.18 (2H, m, H_2-4), 1.50-1.41 (2H, m, H_2-5), 1.35-1.22 (8H, m, H_2-6 H_2-7 H_2-8 H_2-9), 0.87 (3H, t *J* 6.9, H_3-10); δ_C (100 MHz, $CDCl_3$); 172.2 (C-1), 152.7 (C-3), 120.7 (C-2), 32.5 (C-4), 31.9 (C-5), 29.2 (C-6), 29.2 (C-7), 28.0 (C-9), 14.2 (C-10); MS (ES-): *m/z* (%) 169 (100, [M - H]-).

***E*-2-methyldec-2-enoic acid 25**[16]

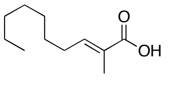

Chemical Formula: $C_{11}H_{20}O_2$
Exact Mass: 184.15

To a solution of ethyl *E*-2-methyldec-2-enoate (synthesised by Wittig reaction of octanal with (Carbethoxyethylidene)triphenylphosphorane, Aldrich) in ethanol/water 5:1 (5 ml/ 1 ml) was added potassium hydroxide. After stirring under reflux for 3 h diethyl ether was added. The mixture was washed with $NaHCO_3$ (3 × 10 ml). Then the aqueous layer was acidified with 2M HCl until pH 1 and extracted with ethyl acetate (3 × 10 ml). The organic layer was dried over $MgSO_4$ and concentrated *in vacuo*. The obtained product was a red oil (0.11 g, 0.60 mmol, 86 %).^{1}H-NMR ($CDCl_3$, 400 MHz): δ_H 0.87-0.90 (m, 3H, 1-CH_3); 1.26-1.30 (m, 10H, 2-3-4-5-6-CH_2); 1.83 (d, *J* = 0.93 Hz, 3H, 10-CH_3); 2.19 (q, *J* = 7.3 Hz, 2H, 7-CH_2); 6.90 (td, *J* = 1.5 Hz, *J* = 7.6 Hz, 1H, 8-CH). ^{13}C-NMR ($CDCl_3$, 100 MHz): δ_C 12.3 (10-CH_3), 14.1 (1-CH_3), 22.6 (2-CH_2), 28.4 (4-CH_2), 28.9 (5-CH_2), 29.1 (6-CH_2), 29.3 (7-CH_2), 31.8 (3-CH_2), 126.8 (9-C), 145.6 (8-CH), 172.8 (11-C); ESMS *m/z* (%) 369 $[M_2]H^+$ (10 %), 226 $[M + CH_3CN]H^+$ (100 %), 167 $[M - H_2O]\ H^+$ (52 %); ν_{max} (oil) / cm^{-1} 2925 (-C-H), 2363 (-O-H), 1712 (C=O), 1650 (-C=C), 1466 (CO), 1367(C-O), 1268 (C-O).

N,*S*-diacetyl cysteamine[17]

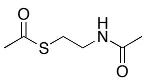

To a solution of cysteamine hydrochloride (3.5 g, 30.8 mmol) in water (20 ml) at 0 °C was added aqueous KOH (6M) until pH 8 was reached. Acetic anhydride (8.7 ml, 92.4 mmol) was added dropwise and the reaction was maintained at a constant pH of 8 using aqueous KOH (6M). After all the acetic anhydride was added the pH was adjusted to 7 with aqueous HCl (2M) and the reaction was stirred at 0 °C for 1.5 h. NaCl was added to the reaction mixture until saturated and the mixture was extracted with CH_2Cl_2 (5 × 25 ml). The combined organics were dried ($MgSO_4$) and concentrated in *vacuo* to give the desired product (colourless oil, 4.8 g, 29.6 mmol, 96%, Rf 0.18, ethyl acetate/hexane, 1:1); δ_H (400 MHz, $CDCl_3$) 6.10 (1H, br s, H-3), 3.42 (2H, m, H_2-2), 3.02 (2H, t *J* 6.5, H_2-1), 2.36 (3H, s, H_3-7), 1.98 (3H, s, H_3-5); δ_C (75 MHz, $CDCl_3$) 196.2 (C-6), 171.3 (C-4, 39.6 (C-2), 30.6 (C-1), 28.6 (C-7), 22.9 (C-5); ν_{max} (oil)/cm^{-1} 3297 (NH), 3085 (CH), 2935 (CH), 1691 (CONH), 954 (COS), 734 (CS).

N-Acetyl cysteamine[17]

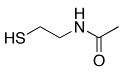

KOH (2.8 g, 49.4 mmol) was added to a solution of *N*, *S* diacetyl cysteamine (2.5 g, 15.4 mmol) in water (10 ml) at 0 °C. The reaction was warmed to RT and stirred for 50 min. The reaction was then cooled to 0 °C and the pH was adjusted to 5 with aqueous HCl (2M). NaCl was added until saturation and extracted with CH_2Cl_2 (10 × 25 ml). The combined organics were dried ($MgSO_4$) and concentrated in *vacuo* to give the desired product (colourless oil, 1.8 g, 14.9 mmol, 97%, Rf 0.07, ethyl acetate/hexane, 1:1); δ_H (400 MHz, $CDCl_3$) 6.23 (1H, br s, H-4), 3.41 (2H, m, H_2-3), 2.66 (2H, dt *J* 8.5 6.5, H_2-2), 2.00 (3H, s, H_3-6), 1.37 (1H, t *J* 8.4, H-1); δ_C (75 MHz, $CDCl_3$) 175.5 (C-5), 42.4 (C-3), 24.7 (C-2), 23.3 (C-6); νM_{ax} (oil)/cm^{-1} 3291 (NH), 3087 (CH), 2934 (CH), 2564 (SH), 1623 (CONH), 734 (CS).

General Procedure for Synthesis of SNAC Thiolesters

N-acetylcysteamine (172 mg, 1.4 mmol) and carboxylic acid (1.3 mmol) were dissolved in CH_2Cl_2 (2 ml) and cooled to 0 °C. DMAP (35 mg, 0.3 mmol) was added followed by EDCI (275 mg, 1.4 mmol) and the reaction was warmed to RT and followed by TLC. On completion the reaction was quenched with aqueous HCl (2M, 5 ml) and extracted with CH_2Cl_2 (3 × 10 ml). The combined organics were washed with saturated $NaHCO_3$ (10 ml), saturated NaCl (10 ml), dried ($MgSO_4$) and concentrated in *vacuo*. The resulting oil was purified by silica column chromatography to yield the desired product.

Tiglic-SNAC 5S[18]

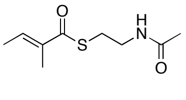

Chemical Formula: $C_9H_{15}NO_2S$
Exact Mass: 201.08

(yellow oil, 75%, 1.0 mmol, Rf 0.16, ethyl acetate:petroleum ether, 4:1); δ_H (400 MHz, $CDCl_3$) 6.82 (1H, qq *J* 6.9 1.3, H-3), 6.28 (1H, br s, H-8), 3.38 (2H, q *J* 6.3, H_2-7), 3.02 (2H, t, *J* 6.5, H_2-6), 1.93 (3H, s, H_3-10), 1.83 (3H, p *J* 1.3, H_3-5) 1.80 (3H, dq *J* 7.0 1.1, H_3-4); δ_C (100 MHz, $CDCl_3$) 193.6 (C-1), 170.3 (C-9), 136.7 (C-3), 136.6 (C-2), 39.6 (C-7), 28.2 (C-6), 23.0 (C-10), 14.3 (C-5), 12.0 (C-4); MS (ES +): *m/z* (%): 425 (16, [M_2 + Na]+), 403 (5, [M_2 + H]+), 224 (100, [M]Na+), 202 (78, [M]H+); ν_{max} (oil)/cm^{-1} 3283 (N-H), 3078 (C-H), 2930 (C-H), 2859 (C-H), 1650 (C=O), 1621 (C=O), 1547 (N- H); elemental analysis; found: C, 53.8; H, 7.6; N, 6.8; S, 15.8; calculated: C, 53.7; H, 7.5; N, 7.0; S, 15.9.

2*RS*-2-Methylbutryic-SNAC 6S[19]

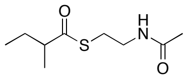

Chemical Formula: $C_9H_{17}NO_2S$
Exact Mass: 203.10

(yellow oil, 71%, 0.9 mmol, Rf 0.21, ethyl acetate:petroleum ether, 4:1), δ_H 400 MHz, $CDCl_3$) 5.86 (1H, br s, H-8), 3.44 (2H, q *J* 6.2, H_2-7), 3.02 (2H, t *J* 6.8, H_2-6), 2.60 (1H, dt *J* 6.9 6.8, H-2), 1.96 (3H, s, H_3-10), 1.79-1.67 (1H, m, H-3), 1.54-1.43 (1H, m, H-3), 1.17 (3H, d *J* 6.8, H_3-5), 0.90 (3H, t *J* 7.4, H_3-4); δ_C (100 MHz, $CDCl_3$) 204.8 (C-1), 170.2 (C-9), 50.2 (C-2), 39.9 (C-7), 28.1 (C-6), 27.1 (C-3), 23.1 (C-10), 17.1 (C-5), 11.5 (C-4); MS (ES +): *m/z* (%): 429 ([M_2 + Na]+), 407 ([M_2 + H]+), 226 ([M]Na+), 204 ([M]H+); νM_{ax} (oil)/cm^{-1} 3284 (N-H), 3078 (C-H), 2967 (C-H), 2933 (C-H), 2877 (C- H), 1684 (O=C), 1651 (O=C), 1548 (N-H); elemental analysis; found: C, 53.9; H, 8.7; N, 6.7; S, 15.1; calculated: C, 53.5, H, 8.4; N, 6.9; S, 15.8.

Crotonyl-SNAC 7S[20]

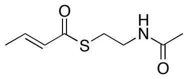

Chemical Formula: $C_8H_{13}NO_2S$
Exact Mass: 187.07

(colourless oil, 68%, 0.9 mmol, Rf 0.23, ethyl acetate:hexane, 4:1); δ_H (400 MHz, $CDCl_3$) 6.95 (1H, dq *J* 15.4 6.78, H-3), 6.17 (1H, dq *J* 15.4 1.7, H-2), 5.90 (1H, br, H- 7), 3.47 (2H, q *J* 6.8, H_2-6), 3.10 (2H, t *J* 6.6, H_2-5), 1.97 (3H, s, H_3-9), 1.91 (3H, dd J 7.0 1.6, H_3-4); δ_C (100 MHz, $CDCl_3$) 190.1 (C-1), 170.4 (C-8), 141.7 (C-3), 129.8 (C- 2), 39.7 (C-6), 28.1 (C-5), 20.0 (C-9), 17.9 (C-4); MS (ES +): *m/z* (%): 397 (6, [M_2 + Na]+), 375 (54, [M_2 + H]+), 188 (100, [M]H+); J max (oil)/cm-1 3250 (N-H), 3070 (C=C-H), 2943 (C-H), 2852 (C-H), 2784 (C-H), 1660 (C=O), 1635 (C=O), 1563 (N-H); HRMS (ESI +): calcd for [M]Na+, $C_8H_{13}NO_2SNa$: 210.0565: found 210.0565.

Angelic-SNAC 8S[21]

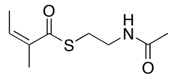

Chemical Formula: $C_9H_{15}NO_2S$
Exact Mass: 201.08

(yellow oil, 53%, 0.7 mmol, Rf 0.16, ethyl acetate:petroleum ether, 4:1); δ_H (400 MHz, $CDCl_3$) 5.93-5.82 (2H, m, H-3 H-8), 3.48 (2H, q *J* 5.9, H_2-7), 3.09 (2H, t *J* 6.6, H_2-6), 2.00 (3H, p *J* 1.3, H_3-5), 1.98 (3H, s, H_3-10), 1.95 (3H, dq *J* 7.2 1.5, H_3-4); δ_C (100 MHz, $CDCl_3$) 202.1 (C-1), 170.2 (C-9), 134.7 (C-3), 134.2 (C-2, 39.8 (C-7), 28.3 (C-6), 23.2 (C-10), 20.5 (C-4), 15.8 (C-5); MS (ES +): *m/z* (%): 425 (20, [M_2 + Na]+), 224 (100, [M]Na+), 202 (70, [M]H+); ν_{max} (oil)/cm^{-1} 3284 (N-H), 3079 (C-H), 2979 (C-H), 2934 (C-H), 1655 (C=O), 1628 (C=O), 1547 (N-H); elemental analysis; found: C, 53.5; H, 7.3; N, 6.9; S, 15.8; predicted: C, 53.7; H, 7.5; N, 7.0; S, 15.9.

***E*-Hex-2-enoic-SNAC 11S**[20]

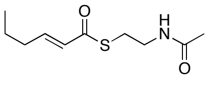

Chemical Formula: $C_{10}H_{17}NO_2S$
Exact Mass: 215.10

(clear oil, 47%, 0.6 mmol, Rf 0.37, ethyl acetate); δ_H (400 MHz, $CDCl_3$) 6.96 (1H, dt, *J* 15.6 6.8, H-3), 6.17 (1H, dt, *J* 15.4 1.5, H-2), 5.91 (1H, br s, H-9), 3.51-3.47 (2H, m, H_2-8), 3.12 (2H, t, *J* 6.6, H_2-7), 2.22 (2H, m, H_2-4), 1.99 (3H, s, H_3-11), 1.58-1.49 (2H, m, H_2-5), 0.97 (3H, t, *J* 7.4, H_3-6); δ_C (100 MHz, $CDCl_3$) 190.6 (C-1), 170.8 (C-10), 146.6 (C-3), 128.4 (C-2), 39.8 (C-8), 34.2 (C-9), 28.2 (C-4), 23.2 (C-11), 21.1 (C-5), 13.7 (C-6); MS (ESI +) *m/z* (%) 238 (29, [M]Na+), 216 (44, [M]H+); υ_{max} (oil)/cm^{-1} 3317 (N-H), 3042 (C=C-H), 2930 (C-H), 2872 (C-H), 1678 (C=O), 1625 (C=O), 1540 (NH); HRMS (CI +) calcd for [M]H + , $C_{10}H_{18}NO_2S$: 216.1058; found 216.1062.

***E*-2-Methylhex-2-enoic SNAC 12S**

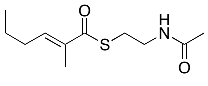

Chemical Formula: $C_{11}H_{19}NO_2S$
Exact Mass: 229.11

(yellow oil, 18%, 0.3 mmol, Rf 0.40, ethyl acetate); δ_H (300 MHz, $CDCl_3$) 6.78 (1H, tq *J* 7.4 1.3, H-3), 5.86 (1H, br s, H-10), 3.49-3.43 (2H, m, H_2-9), 3.07 (2H, t *J* 6.52, H_2- 8), 2.23-2.16 (2H, m, H_2-4), 1.92 (3H, s, H_3-12), 1.88-1.86 (3H, m, H_3-7), 1.54-1.47 (2H, m, H_2-5), 0.95 (3H, t *J* 7.4, H_3-6); δ_C (100 MHz, $CDCl_3$) 194.2 (C-1), 170.4 (C- 11), 142.2 (C-3), 136.0 (C-2), 40.0 (C-9), 30.9 (C-4), 28.5 (C-8), 23.4 (C-12), 21.9 (C- 7), 14.0 (C-5), 12.6 (C-6); MS (ES +): *m/z* (%) 252 (14, [M]Na+), 230 (8, [M]H+); υ_{max} (oil)/cm^{-1} 3285 (NH), 3078 (HC=C), 2960 (CH), 2931 (CH), 2872 (CH), 1651 (CO), 1547 (NH); elemental analysis, observed (%) C, 57.7; H, 8.0; N, 6.1; S, 13.6; predicted C, 57.6; H, 8.4; N 6.1, S, 14.0; HRMS (CI +): calcd for [M]H+ , $C_{11}H_{20}NO_2S$: 230.1215: found 230.1213.

***E*-4*RS*-4-Methylhex-2-enoic-SNAC 13S**[22]

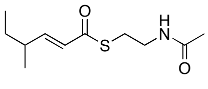

Chemical Formula: $C_{11}H_{19}NO_2S$
Exact Mass: 229.11

(yellow oil, 54%, 0.7 mmol, Rf 0.28, ethyl acetate:hexane, 4:1); δ_H (400 MHz, $CDCl_3$) 6.83 (1H, dd *J* 15.6 7.7, H-3), 6.09 (1H, dd *J* 15.6 1.1, H-2), 5.88 (1H, br s, H-10), 3.49- 3.45 (2H, m, H_2-9), 3.09 (2H, t *J* 6.1, H_2-8), 2.26-2.19 (1H, m, H-4), 1.97 (3H, s, H_3- 12), 1.46-1.39 (2H, m, H_2-5), 1.06 (3H, d *J* 6.7, H_3-7), 0.89 (3H, t *J* 7.4, H_3-6); δ_C (100 MHz, $CDCl_3$) 190.6 (C-1), 170.2 (C-11), 151.7 (C-3), 127.8 (C-2), 39.8 (C-9), 38.2 (C- 4), 28.7 (C-5), 28.3 (C-8), 23.2 (C-12), 18.8 (C-7), 11.6 (C-6); MS (ES +): *m/z* (%) 252 (100, [M]Na+), 230 (13, [M]H+); υ_{max} (oil)/cm^{-1} 3283 (N-H), 3065 (C=C-H), 2963 (C-H), 2930 (C-H), 2870 (C-H), 1651 (C=O), 1629 (C=O), 1547 (N-H); elemental analysis, observed (%) C, 57.5; H, 8.3; N, 6.2; S, 14.1; predicted C, 57.6; H, 8.4; N 6.1, S, 13.8; HRMS (CI +): calcd for [M]H+ , $C_{11}H_{20}NO_2S$: 230.1215; found: 230.1210.

***E*-4*RS*-2,4-Dimethylhex-2-enoic-SNAC 14S**

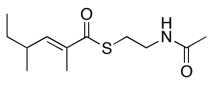

Chemical Formula: $C_{12}H_{21}NO_2S$
Exact Mass: 243.13

(yellow oil, 51%, 0.7 mmol, Rf 0.31 ethyl acetate:hexane, 4:1); δ_H (400 MHz, $CDCl_3$) 6.53-6.49 (1H, m, H-3), 6.10 (1H, br s, H-11), 3.42 (2H, q *J* 6.1, H_2-10), 3.04 (2H, t *J* 6.4, H_2-9), 2.47-2.38 (1H, m, H-4) 1.95 (3H, s, H_3-13), 1.87-1.84 (3H, m, H_3-8), 1.47- 1.38 (1H, m, H-5), 1.38-1.28 (1H, m, H-5), 1.00 (3H, d *J* 6.8, H_3-7), 0.84 (3H, t *J* 7.4, H_3-6); δ_C (100 MHz, $CDCl_3$) 194.3 (C-1), 170.5 (C-12), 147.6 (C-3), 134.7 (C-2), 39.9 (C-10), 35.2 (C-4), 29.7 (C-5), 28.5 (C-9), 23.3 (C-13), 19.6 (C-8), 12.7 (C-7), 12.0 (C- 6); MS (ES +): *m/z* (%) 266 (44, [M]Na+), 244 (4, [M]H+); υ_{max} (oil)/cm^{-1} 3285 (N-H), 3091 (C=C-H), 2961 (C-H), 2929 (C- H), 2870 (C-H), 1670 (C=O), 1653 (C=C), 1548 (N-H); elemental analysis, observed (%) C, 59.0; H, 8.6; N, 5.7; S, 13.0; predicted C, 59.2; H, 8.7; N 5.7, S, 13.2.

***E*-Hept-2-enoic-SNAC 17S**

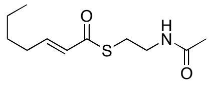

Chemical Formula: $C_{11}H_{19}NO_2S$
Exact Mass: 229.11

(pale yellow oil, 32%, 0.4 mmol, Rf 0.45, ethyl acetate); δ_H (400 MHz, $CDCl_3$) 6.89 (1H, dt *J* 15.5 6.9, H-3), 6.21 (1H, br s, H-10), 6.08 (1H, dt *J* 15.4, 1.6, H-2), 3.42 (2H, q *J* 6.1, H_2-9), 3.05 (2H, t *J* 6.4, H_2-8), 2.21-2.14 (2H, m, H_2-4), 1.93 (3H, s, H-12), 1.46-1.37 (2H, m, H_2-5), 1.34-1.27 (2H, m, H_2-6), 0.87 (3H, t *J* 7.3, H_3-7); δ_C (100 MHz, $CDCl_3$) 190.4 (C-1), 170.5 (C-11), 146.8 (C-3), 128.4 (C-2), 39.8 (C-9), 31.9 (C- 4), 30.5 (C-5), 28.3 (C-8), 23.2 (C-12), 22.3 (C-6), 13.9 (C-7); MS (ES +): *m/z* (%) 252 (100, [M]Na+), 230 (7, [M]H+); υ_{max} (oil)/cm^{-1} 3284 (br N-H), 3081 (C=C-H), 2958 (C-H0, 2930 (C-H), 2872 (C-H), 1653 (C=O), 1631 (C=O), 1548 (N-H); elemental analysis, observed (%) C, 58.0; H, 8.5; N, 5.7; S, 13.6; predicted C, 57.7; H, 8.4; N 6.1; S, 14.0.

***E*-6-Methylhept-2-enoic-SNAC 18S**

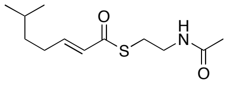

Chemical Formula: $C_{12}H_{21}NO_2S$
Exact Mass: 243.13

(colourless oil, 72% (0.9 mmol), Rf 0.34, ethyl acetate): δ_H (400 MHz, $CDCl_3$); 6.94 (1H, dt *J* 15.5 6.9, H-3), 6.14 (1H, dt *J* 15.5 1.5, H-2), 5.97-5.77 (1H, br s, H-10), 3.46 (2H, td *J* 6.1, H_2-9), 3.09 (2H, t *J* 6.6, H_2-8), 2.21 (2H, tdd *J* 7.5 7.5 1.6, H_2-4), 1.96 (3H, s, H_3-12), 1.61-1.52 (1H, m, H-6), 1.38-1.32 (2H, m, H_2-5), 0.90 (6H, d *J* 6.6, H6-7); δ_C (100 MHz, $CDCl_3$); 190.6 (C-1), 170.4 (C-11), 147.2 (C-3), 128.3 (C-2), 40.0 (C-9), 37.1 (C-5), 30.3 (C-4), 28.4 (C-8), 27.8 (C-6), 23.4 (C-12), 22.5 (C-7); MS (ES +): *m/z* (%) 487 (100, [M_2 + H]+), 244 (79, [M]H+); υ_{max} (oil)/cm^{-1} 3282 (br, N-H), 3079 (C=C-H), 2956 (C-H), 2930 (C-H), 2870 (C-H), 1654 (C=O), 1631 (C=O), 1548 (N-H); HRMS (ESI +): calcd for [M]Na+ , $C_{12}H_{21}NO_2SNa$: 266.1191: found 266.1192.

***E*-Oct-2-enoic-SNAC 19S**[23]

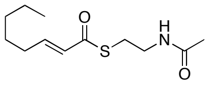

Chemical Formula: $C_{12}H_{21}NO_2S$
Exact Mass: 243.13

(pale yellow oil, 73%, 0.8 mmol, Rf 0.37, ethyl acetate); δ_H (300 MHz, $CDCl_3$) 6.93 (1H, dt *J* 15.4 7.2, H-3), 6.12 (1H, dt *J* 15.5 1.5, H-2), 5.89 (1H, br s, H-11), 3.46 (2H, dt *J* 6.6 5.7, H_2-10), 3.09 (2H, t *J* 6.5, H_2-9), 2.25-2.15 (2H, m, H_2-4), 1.96 (3H, s, H_3-13), 1.52-1.42 (2H, m, H_2-5), 1.35-1.27 (4H, m, H_2-6, H_2-7), 0.89 (3H, t, *J* 6.3, H_3-8); δ_C (100 MHz, $CDCl_3$) 190.6 (C-1), 170.5 (C-12), 147.0 (C-3), 128.4 (C-2), 40.0 (C-10), 32.3 (C-4), 31.6 (C-9), 28.4 (C-5), 27.7 (C-6), 23.3 (C-13), 22.5 (C-7), 14.1 (C- 8); MS (ES +): *m/z* (%) 266 (15, [M]Na+), 244 (100, [M]H+); υ_{max} (oil)/cm^{-1} 3286 (br, N-H), 3078 (H-C=C), 2957 (C-H), 2929 (C-H), 2858 (C-H), 1654 (C=O), 1631 (C=O), 1548 (NH); elemental analysis, observed (%) C, 59.6; H, 8.5; N, 5.8; S, 13.2; predicted C, 59.2; H, 8.7; N 5.8, S, 13.1.

***E*-2-Methyloct-2-enoic-SNAC 20S**

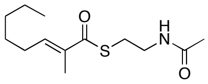

Chemical Formula: $C_{13}H_{23}NO_2S$
Exact Mass: 257.14

(colourless oil, 22%, 0.3 mmol, Rf 0.53, ethyl acetate); δ_H (400 MHz, $CDCl_3$); 6.74 (1H, tq *J* 7.3 1.3, H-3), 6.12 (1H, br s, H-12), 3.41 (2H, dt *J* 6.6 5.0, H_2-11), 3.03 (2H, t *J* 6.4, H_2-10), 2.17 (2H, dtq *J* 7.4 7.3 0.8, H_2-4), 1.94 (3H, s, H_3-14), 1.84 (3H, dt *J* 1.1 0.9, H_3-9), 1.47-1.40 (2H, m, H_2-5), 1.35-1.24 (4H, m, H_2-6 H_2-7), 0.87 (3H, t *J* 7.1, H_3- 8); δ_C (100 MHz, $CDCl_3$); 190.4 (C-1), 170.4 (C-13), 142.4 (C-3), 135.8 (C-2), 39.9 (C- 11), 31.4 (C-6), 28.9 (C-4), 28.5 (C-5), 28.2 (C-10), 23.3 (C-14), 22.3 (C-7), 13.9 (C-8); MS (ES +): *m/z* (%) 280 (21, [M]Na+), 258 (2, [M]H+); υ_{max} (oil)/cm^{-1} 3285 (br N-H), 3079 (C=C-H), 2960 (C-H), 2926 (C-H), 2858 (C-H), 1652 (C=O), 1548 (N-H); elemental analysis, observed (%) C, 60.9; H, 9.0; N, 5.4; S, 12.1; predicted C, 60.7; H, 9.0; N, 5.4; S, 12.5.

***E*-7-Methyl oct-2-enoic SNAC 21S**[24]

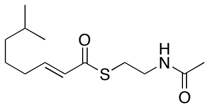

Chemical Formula: $C_{13}H_{23}NO_2S$
Exact Mass: 257.14

(colourless oil, 20%, 0.3 mmol, Rf 0.36, ethyl acetate); δ_H (400 MHz, $CDCl_3$); 6.94 (1H, dt *J* 15.5 6.9, H-3), 6.13 (1H, dt *J* 15.5 1.5, H-2), 5.89 (1H, br s, H-11), 3.47 (2H, dt *J* 6.0 6.0, H_2-10), 3.09 (2H, t *J* 6.4, H_2-9), 2.25 (2H, tdd *J* 7.0 7.0 1.5, H_2-4), 1.96 (3H, s, H_3-13), 1.57-1.42 (3H, m, H_2-5 H-7), 1.20 (2H, dt *J* 6.9 6.9, H_2-6), 0.88 (6H, d *J* 6.6, H6-8); δ_C (100 MHz, $CDCl_3$); 190.6 (C-1), 170.1 (C-12) 147.0 (C-3), 128.4 (C-2), 40.0 (C-10), 38.6 (C-6), 32.7 (C-4), 29.9 (C-5), 28.4 (C-9), 28.0 (C-7), 23.4 (C-13), 22.7 (C-8); MS (ES +): *m/z* (%) 515 (43, $[M_2 + H]+$), 258 (100, [M]H+); υ_{max} (oil)/cm^{-1} 3278 (br N-H), 3079 (C=C-H), 2954 (C-H), 2930 (C-H), 2869 (C-H), 1653 (C=O), 1632 (C=O), 1547 (N-H); HRMS (ESI +): calcd for [M]Na+ , $C_{13}H_{23}NO_2SNa$: 280.1347: found 280.1347.

E-4*RS*,6*RS*-4,6-Dimethyl-oct-2-enoic-SNAC 22S

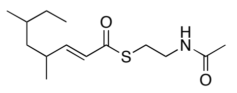

Chemical Formula: $C_{14}H_{25}NO_2S$
Exact Mass: 271.16

An inseparable mixture of diastereomers A and B 3:2 (yellow oil, 32%, 0.4 mmol, Rf 0.36, ethyl acetate); δ_H (300 MHz, $CDCl_3$); 6.84 (0.5H, dd *J* 15.5 7.7, H-3A), 6.79 (0.5H, dd *J* 15.7 8.3, H-3B), 6.11-6.06 (1H, m, H-2), 5.87 (1H, br s, H-13), 3.47 (2H, q *J* 6.5. H_2-12 A), 3.09 (2H, t *J* 6.3, H_2-11), 2.45-2.34 (1H, m, H-4), 1.97 (3H, s, H_3-15), 1.42-1.23 (3H, m, H_2-5 H-6), 1.20-1.10 (2H, m, H_2-7), 1.04 (3H, dd *J* 6.7 6.7, H_3-10), 0.88-0.81 (6H, m, H_3-8 H_3-9); δ_C (100 MHz, $CDCl_3$); 190 (C-1), 170.4 (C-14), 152.6 (C-3A), 152.1 (C-3B), 126.7 (C-2A), 126.4 (C-2B), 43.4 (C-5A), 43.1 (C-5B), 40.0 (C-12), 34.5 (C-4A). 34.3 (C-4B), 32.1 (C-6A), 31.9 (C-6B), 29.9 (C-7A), 29.5 (C-7B), 28.4 (C-11), 23.4 (C-15), 20.3 (C-10), 19.3 (C-9A), 19.2 (C- 9B), 11.3 (C-8A), 11.2 (C-8B); MS (ES +): *m/z* (%) 294 (27, [M]Na+), 272 (100, [M]H+); υ_{max} (oil)/cm^{-1} 3287 (br, NH), 3075 (HC=C), 2961 (C-H), 2926 (C-H), 2874 (C-H), 1654 (CO), 1629 (CO), 1548 (NH); elemental analysis, observed (%) C, 62.4; H, 9.0; N, 5.1; S, 12.2; predicted C, 62.0; H, 9.3; N, 5.2; S, 11.8.

E-Dec-2-enoic SNAC 24S[25]

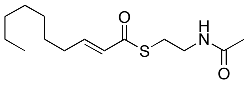

Chemical Formula: $C_{14}H_{25}NO_2S$
Exact Mass: 271.16

(white solid, 27%**,** 0.4 mmol, Rf 0.33, ethyl acetate); δ_H (400 MHz, $CDCl_3$); 6.94 (1H, dt *J* 15.5 7.1, H-3), 6.13 (1H, dt *J* 15.5 1.5, H-2), 5.86 (1H, br s, H-13), 3.46 (2H, td *J* 6.2 6.2, H_2-12), 3.09 (2H, t *J* 6.3, H_2-11), 2.21 (2H, dtd *J* 7.2 7.2 1.5, H_2-4), 1.96 (3H, s, H_3-15), 1.47 (2H, tt *J* 7.3 7.2, H_2-5), 1.34-1.29 (8H, m, H_2-6 H_2-7 H_2-8 H_2-9), 0.88 (3H, t *J* 7.1, H_3-10); δ_C (100 MHz, $CDCl_3$); 190.8 (C-1), 170.4 (C-14), 147.1 (C-3), 128.4 (C- 2), 40.0 (C-12), 32.4 (C-4), 31.9 (C-8), 29.3 (C-6), 29.2 (C-7), 28.4 (C-11), 28.1 (C-5), 23.4 (C-15), 22.8 (C-9), 14.3 (C-10); MS (ES-): *m/z* (%) 543 (25, [M_2 + H]+), 272 (100, [M]H+); υ_{max} (oil)/cm^{-1} 3284 (N-H), 3078 (C=C-H), 2954 (C-H), 2930 (C-H), 2869 (C.H), 1653 (C=O), 1632 (C=O), 1547 (N-H); HRMS (ESI +): calcd for [M]Na+ , $C_{14}H_{25}NO_2SNa$: 294.1504: found 294.1501.

Pantetheine dimethyl ketal[26]

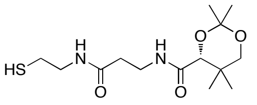

D-pantothenic acid hemi-calcium salt (2.50 g, 10.50 mmol), p-toluensulfonic acid (2.30 g, 13.00 mmol) and molecular sieves (5 g) were suspended in dry acetone (125 ml) and stirred at 25 °C for 12 h under a nitrogen atmosphere. The suspension was filtered with celite and washed with 200 ml acetone. The filtrate was concentrated to a colourless oil, redissolved in ethyl acetate (200 ml) and washed with brine (2 × 25 ml) and dried over $MgSO_4$. After that the ethyl acetate was removed under vacuum and hexane was added to the flask to give a white solid that was dried under high vacuum. The corresponding D-pantothenic dimethyl ketal (1.90 g, 7.00 mmol was dissolved in 40 ml dry THF with CDI (1.70 g, 11.00 mmol) and stirred for one hour at 25 °C. Then cysteamine (1.30 g, 11.00 mmol) was added to the solution and stirred for 12 h. The solution was concentrated under vacuum and CH_2Cl_2 was added. The organic layer were washed with NH_4Cl (1 × 25 ml) and brine (1 × 25 ml), dried over $MgSO_4$ and concentrated *in vacuo*. The colourless oil was purified by column chromatography (ethyl acetate) to give a white solid (1.90 g, 6.00 mmol, 86 %). R_f: 0.1 (ethylacetate). ^{1}H-NMR ($CDCl_3$, 400 MHz): δ_H 0.98 (s, 3H, 11-CH_3); 1.05 (s, 3H, 12-CH_3); 1.39 (t, *J* = 8.6 Hz, 1H, SH); 1.43 (s, 3H, 15-CH_3); 1.47 (s, 3H, 16-CH_3); 2.40 (t, *J* = 5.8 Hz, 2H, 5-CH_2); 2.64-2.70 (m, 2H, 1-CH_2); 3,29 (d, *J* = 12.0 Hz, 1H, 13a-CH_2); 3.37-3.63 (m, 4H, 2-6-CH_2); 3,69 (d, *J* = 12.0 Hz, 1H, 13b-CH_2); 4.09 (s,1H, 9-CH); 6.37

(bt, J = 5.2 Hz, 1H, 3-NH); 7.03 (bt, J = 5.9 Hz, 1H, 7-NH). ^{13}C-NMR ($CDCl_3$, 100 MHz): δ_C 18.7 (12-CH_3); 18.9 (11-CH_2); 22.1 (15-CH_3); 24.6 (1-CH_2); 29.5 (15-CH_3); 33.0 (10-C); 34.9 (6-CH_2); 36.2 (5-CH_2); 42.4 (2-CH_2); 71.4 (13-CH_2); 77.2 (9-CH); 99.1 (14-C); 170.3 (4-CO); 171.1 (8-CO). ESMS: m/z (%): 319 $[M]H^+$ (65%), 261 $[M-(CH_3)_2CO]H^+$ (100%). IR (ν_{max}/cm^{-1}): 3419 (N-H), 3324 (N-H), 2980 (CH), 2944 (CH), 2872 (CH), 2556 (C-O), 1659 (-C=O).

tigloyl pantetheine dimethyl ketal 5Pa

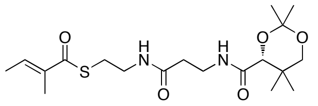

Chemical Formula: $C_{19}H_{32}N_2O_5S$
Exact Mass: 400.20

Tiglic acid (**5**, 0.10 g, 1.00 mmol) and pantetheine dimethyl ketal (0.32 g, 1.00 mmol,) were dissolved in CH_2Cl_2 (8 ml). The mixture was cooled to 0 °C. Then *N*,*N*-dimethylaminopyridine (0.10 g, 0.80 mmol,) and *N*-(3-Diethylamino-propyl)-*N*-ethylcarbodiimide (0.38 g, 2.00 mmol) were added. The mixture was warmed to 25 °C and stirred for 4 h. After that the mixture was quenched with 2M HCl (10 ml) and extracted with CH_2Cl_2 (3 × 25 ml). The organic layer was washed with saturated $NaHCO_3$ (20 ml) and brine (20 ml). The product was dried over $MgSO_4$ and concentrated *in vacuo*. The crude product was purified by column chromatography (ethyl acetate). The obtained product was a colourless oil (0.32 g, 0.80 mmol, 80 %). R_f: 0.46 (ethyl acetate). ^{1}H-NMR ($CDCl_3$, 400 MHz): δ_H 0.99 (s, 3H, 18-CH_3); 1.04 (s, 3H, 19-CH_3); 1.44 (s, 3H, 16-CH_3); 1.48 (s, 3H, 17-CH_3); 1.65 (s, 3H, 4-CH_3); 1.85-1.87 (m, 6H, 1-4-CH_3); 2.44 (t, J = 6.5 Hz, 2H, 9-CH_2); 3.08 (t, J = 6.3 Hz, 2H, 6-CH_2); 3.28 (d, J = 11.7 Hz, 1H, 14a-CH_2); 3.41-3.62 (m, 4H, 7-10-CH_2); 3.68 (d, J = 11.6 Hz, 1H, 14b-CH_2); 4.08 (s,1H, 12-CH); 6.13 (bt, J = 5.8 Hz, 1H, 8-NH); 6.93 (qq, J = 1.4, 6.8 Hz, 1H, 2-CH); 7.03 (bt, J = 5.9 Hz, 1H, 11-NH). ^{13}C-NMR ($CDCl_3$, 100 MHz): δ_C 12.2 (4-CH_3); 14.4 (1-CH_3); 18.7 (18-CH_3); 18.9 (16-CH_3); 22.1 (19-CH_3); 28.3 (6-CH_2); 29.5 (17-CH_3); 32.9 (13-C); 34.8 (9-CH_2); 35.9 (10-CH_2); 39.7 (7-CH_2); 71.5 (14-CH_2); 77.2 (12-CH); 99.1 (15-C); 136.8 (3-CH); 136.9 (2-CH); 170.1 (8-CO); 171.1 (11-CO); 190.2 (5-CO). ESMS: m/z (%) 423.3 [M]Na$^+$ (100 %). IR (ν_{max} / cm^{-1}): 3306 (NH), 2940 (CH_2), 1648 (CO), 1523 (C-O), 1438 (C=C). HRMS: m/z (%): calculated 423.1930, found 423.1929 for [M]Na+.

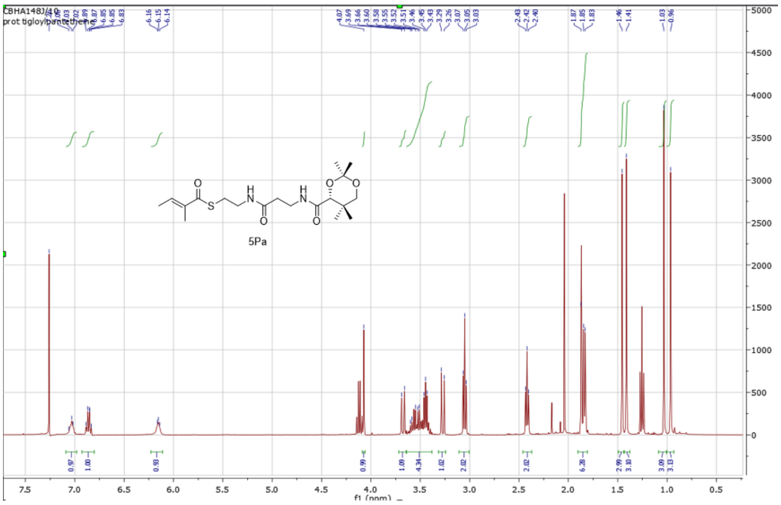

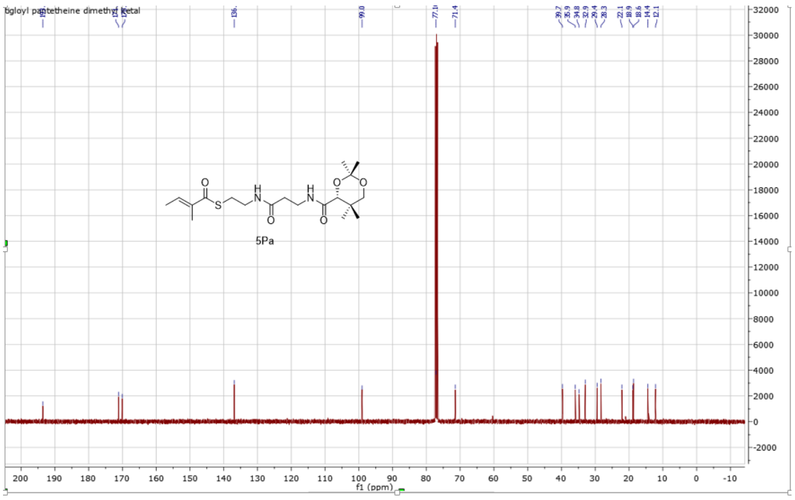

Preparation of angeloyl pantetheine dimethyl ketal 8Pa

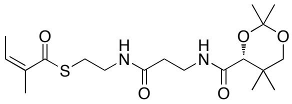

Chemical Formula: $C_{19}H_{32}N_2O_5S$
Exact Mass: 400.20

Angelic acid (**8**, 0.11 g, 1.00 mmol) and pantetheine dimethyl ketal (0.32 g, 1.00 mmol) were dissolved in CH_2Cl_2 (8 ml). Then the mixture was cooled to 0 °C. Then *N*,*N*-dimethylaminopyridine (0.1 g, 0.80 mmol) and *N*-(3-Diethyl-aminopropyl)-*N*-ethylcarbodiimide (0.38 g, 2.00 mmol) were added to the mixture. The mixture was warmed to 25 °C and then stirred for 4 h. After that the mixture was quenched with 2M HCl (10 ml) and extracted with CH_2Cl_2 (3 × 20 ml). The organic layer was washed with saturated $NaHCO_3$ (25 ml) and brine (25 ml). The product was dried over $MgSO_4$ and concentrated *in vacuo*. The crude product was purified by column chromatography (ethyl acetate). The obtained product was a yellow oil (0.29 g, 0.70 mmol, 70 %). R_f: 0.49 (ethyl acetate). ^{1}H-NMR ($CDCl_3$, 400 MHz): δ_H 0.97 (s, 3H, 19-CH_3); 1.04 (s, 3H, 18-CH_3); 1.41 (s, 3H, 16-CH_3); 1.46 (s, 3H, 17-CH_3); 1.92-1.97 (m, 3H, 1-CH_3); 1.99 (t, *J* = 1.5 Hz, 1H, 4-CH_3); 2.19 (dg, *J* = 1.5, 7.3 Hz, 2H, 3-CH_2); 2.42 (t, *J* = 6.0 Hz, 2H, 9-CH_2); 3.07 (t, *J* = 6.7 Hz, 2H, 6-CH_2); 3.27 (d, *J* = 11.6 Hz, 1H, 14a-CH_2); 3.40-3.62 (m, 4H, 7-10-CH_2); 3.68 (d, *J* = 11.6 Hz, 1H, 14b-CH_2); 4.07 (s,1H, 12-CH); 5.89 (dt, *J* = 1.3, 7.3 Hz, 1H, 2-CH); 6.11 (bt, *J* = 5.1 Hz, 1H, 8-NH); 7.03 (bt, *J* = 5.9 Hz, 1H, 11-NH). ^{13}C-NMR ($CDCl_3$, 100 MHz): δ_C 15.9 (1-CH_3); 18.7 (19-CH_3); 18.9 (16-CH_3); 20.5 (4-CH_3); 22.1 (18-CH_3); 28.4 (6-CH_2); 29.5 (17-CH_3); 32.9 (13-C); 34.8 (9-CH_2); 35.9 (10-CH_2); 39.8 (7-CH_2); 71.5 (14-CH_2); 77.3 (12-CH); 99.1 (15-C); 134.1 (3-C); 134.9 (2-CH); 170.0 (8-CO); 171.2 (11-CO); 193.9 (5-CO). ESMS: *m/z* (%) 423.4 $[M]Na^+$ (25 %), 401.4 $[M]H^+$ (28 %), 343.4 $[M - C_3H_6O]H^+$ (100 %). IR (ν_{max} = cm^{-1}): 3260 (NH), 3078 (CH_2), 2924 (CH_2), 1651 (CO), 1538 (C-O), 1446 (C=C). HRMS: *m/z* (%): calculated 423.1930, found 423.1932 for [M]Na+.

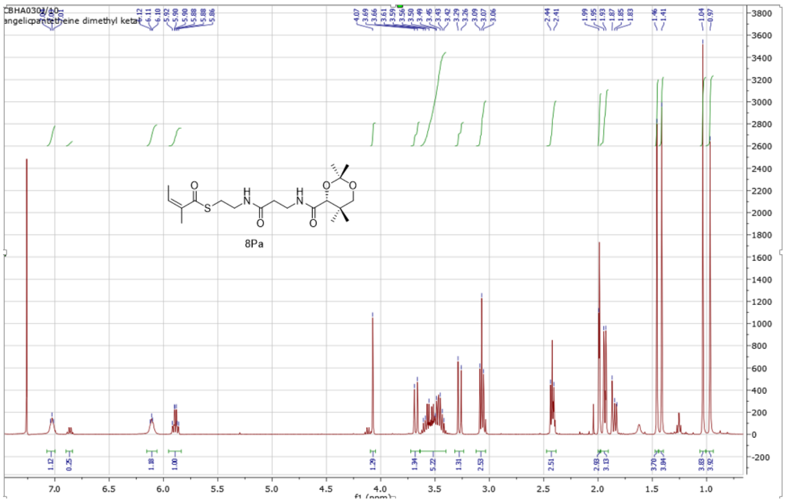

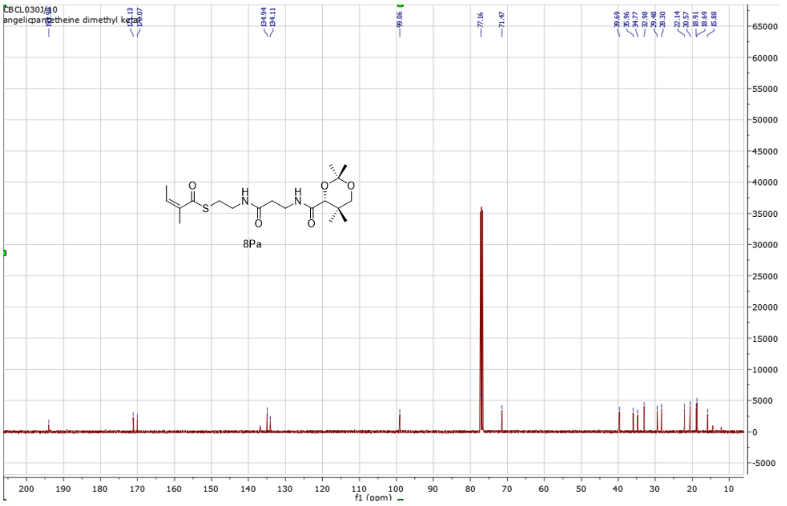

2,3-Dimethylbut-2-enoyl panetheine dimethyl acetal 9Pa

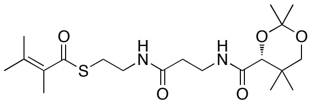

Chemical Formula: $C_{20}H_{34}N_2O_5S$
Exact Mass: 414.22

The reaction was carried out with 0.17 g (1.50 mmol) of 2,3-dimethylbut-2-enoic acid,[27] 0.48 g (1.50 mmol) of pantetheine dimethyl ketal, 0.29 g (1.50 mmol) of EDCI and a catalytic amount of DMAP in 10 ml CH_2Cl_2. The solution was added to aq. HCl (10 ml, 2 M) and the product was extracted with 3 × 20 ml CH_2Cl_2. The organic phase was washed with 1 × 50 ml of a saturated $NaHCO_3$ solution and with 1 × 50 ml of a saturated NaCl solution. The product was a colorless oil (0.55 g, 1.32 mmol, 88 %). ^{1}H NMR (400 MHz, $CDCl_3$): δ_H = 0.96 (3 H, s, 10/11-CH_3), 1.03 (3 H, s, 10/11-CH_3), 1.41 (3 H, s, 13/14-CH_3), 1.45 (3 H, s, 13/14-CH_3), 1.78 (3 H, s, 19-CH_3), 1.92 (3 H, s, 18-CH_3 and 20-CH_3), 2.41 (2 H, t, *J*HH = 6.1 Hz, 6-CH_2), 3.04 (2 H, t, *J*HH = 6.5 Hz, 9-CH_2), 3.27 (1 H, d, 3*J*HH = 11.7 Hz, 1a/b-CH), 3.31-3.64 (4 H, overlapping peaks, 5-CH_2 and 8-CH_2), 3.67 (1 H, d, 3*J*HH = 11.7 Hz, 1a/b-CH), 4.07 (1 H, s, 3-CH), 6.18 (1 H, br. t, NH), 7.03 (1 H, br. t, NH) ppm. ^{13}C NMR (100 MHz, $CDCl_3$): δ_C = 16.2 (18-CH_3 and 20-CH_3), 18.8 (10/11-CH_3), 19.1 (13/14-CH_3), 22.3 (10/11-CH_3), 23.0 (19-CH_3), 28.7 (9-CH_2), 29.6 (13/14-CH_3), 33.1 (2-C), 34.9 (5-CH_2), 36.1 (6- CH_2), 39.9 (8-CH_2), 71.6 (1-CH_2), 77.3 (3-CH), 99.2 (12-C), 129.4 (17-C), 140.6 (16-C), 170.2 (7-CO), 171.3 (4-CO), 196.2 (15-CO) ppm. IR: ν_{max} = 3307 (w), 2937 (w), 2871 (w), 1737 (w), 1652 (vs), 1521 (s), 1438 (w), 1376 (m), 1288 (w), 1254 (w), 1222 (w), 1196 (m), 1159 (w), 1096 (s), 1048 (w), 1018 (w), 1000 (w), 931 (m), 900 (w), 873 (w), 784 (w), 690 (w), 642 (w) cm^{-1}. ESMS: *m/z* (%) = 437 (15) [M]Na+ , 415 (65) [M]H+ , 357 (100) [M + H - C_3H_6O]+.

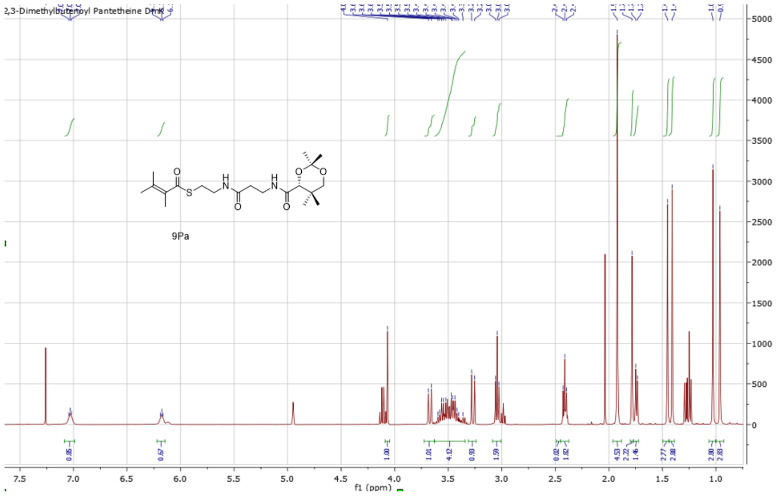

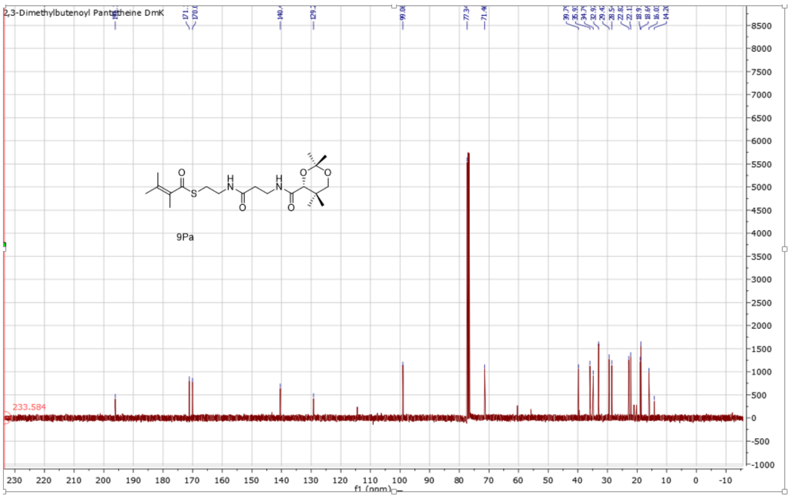

_E_-2-Ethylbut-2-enoyl pantetheine dimethyl acetal 10Pa

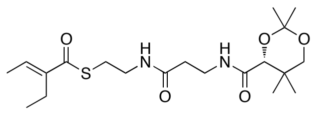

Chemical Formula: $C_{20}H_{34}N_2O_5S$
Exact Mass: 414.22

The reaction was carried out with 0.58 g (5.09 mmol) of **10**, 1.62 g (5.09 mmol) of pantetheine dimethyl ketal, 0.97 g (5.09 mmol) of EDCI and a catalytic amount of DMAP in 10 ml CH_2Cl_2. The solution was dumped in 10 ml HCl (2 M) and the product was extracted with 3 × 20 ml CH_2Cl_2. The organic phase was washed with 1 × 50 ml of a saturated NaHCO3 solution and with 1 × 50 ml of a saturated NaCl solution. The product was a colorless oil (0.99 g, 2.38 mmol, 47 %). ^{1}H NMR (400 MHz, $CDCl_3$): δ_H = 0.96 (3 H, s, 10/11-CH_3), 0.99 (3 H, t, *J*HH = 7.5 Hz, 20-CH_3), 1.03 (3 H, s, 10/11-CH_3), 1.41 (3 H, s, 13/14-CH_3), 1.45 (3 H, s, 13/14-CH_3), 1.82-1.90 (3 H, m, 18-CH_3), 2.41 (2 H, t, *J*HH = 6.1 Hz, 6-CH_2), 2.28-2.45 (2 H, overlapped peak, 19-CH_2), 3.04 (2 H, t, *J*HH = 6.3 Hz, 9-CH_2), 3.27 (1 H, d, 3*J*HH = 11.5 Hz, 1a/b-CH), 3.38-3.64 (4 H, overlapping peaks, 5-CH_2 and 8-CH_2), 3.67 (1 H, d, 3*J*HH = 11.5 Hz, 1a/b-CH), 4.07 (1 H, s, 3-CH), 6.16 (1 H, br. t, NH), 6.81 (1 H, q, *J*HH = 7.0 Hz, 17-CH); 7.03 (1 H, br. t, NH) ppm. ^{13}C NMR (100 MHz, $CDCl_{3)}$: δ_C = 13.5 (20-CH_3), 14.3 (18CH$_3$), 18.8 (10/11-CH_3), 19.1 (13/14-CH_3), 20.2 (19-CH_2), 22.3 (10/11-CH_3), 28.5 (9-CH_2), 29.6 (13/14-CH_3), 33.1 (2-C), 35.0 (5-CH_2), 36.1 (6-CH_2), 39.9 (8-CH_2), 71.6 (1-CH_2), 77.3 (3-CH), 99.2 (12-C), 136.7 (17-CH), 143.3 (16-CH), 170.2 (7-CO), 171.3 (4-CO), 193.5 (15-CO) ppm. IR: ν_{max} = 3306 (w), 3080 (w), 2937 (w), 2873 (w), 1738 (w), 1650 (vs), 1523 (s), 1461 (w), 1377 (m), 1288 (w), 1254 (w), 1196 (m), 1159 (w), 1096 (s), 1049 (w), 1017 (w), 946 (w), 932 (w), 900 (w), 872 (w), 844 (w), 787 (w), 755 (w), 691 (w), 653 (w) cm^{-1}. ESMS (70 eV): *m*/*z* (%) = 437 (12) [M]Na+ , 415 (30) [M]H+ , 357 (100) [M + H - C_3H_6O]+.

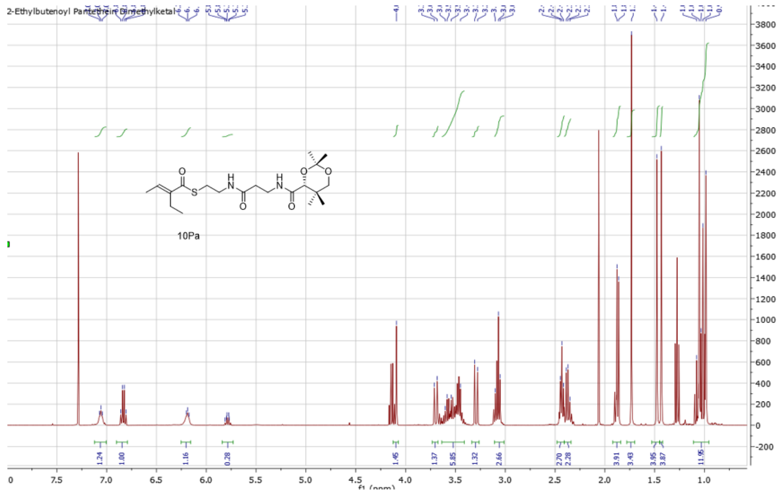

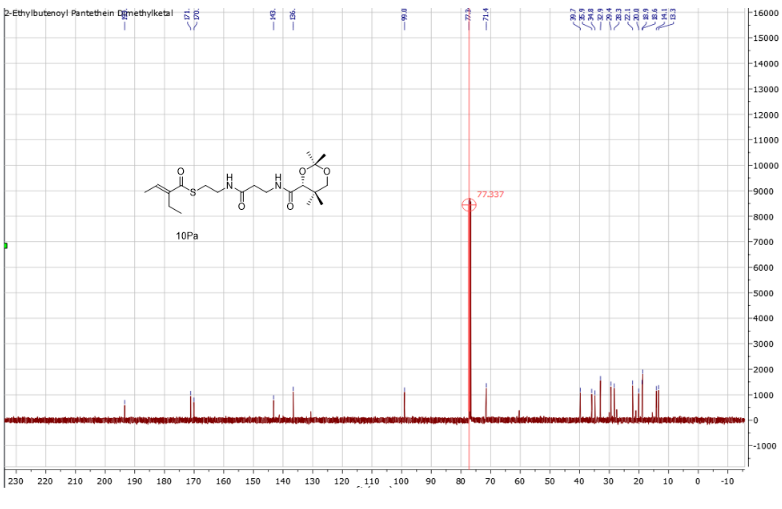

***E*-hex-2-enoic pantetheine dimethyl ketal 11Pa**

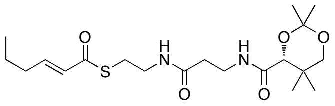

Chemical Formula: $C_{20}H_{34}N_2O_5S$
Exact Mass: 414.22

E-Hex-2-enoic acid (**11**, 0.11 g, 1.00 mmol) and pantetheine dimethyl ketal (0.32 g, 1.00 mmol) were dissolved in CH_2Cl_2 (8 ml). Then the mixture was cooled to 0 °C. Then *N*,*N*-dimethylaminopyridine (0.1 g, 0.80 mmol) and *N*-(3-Diethyl-aminopropyl)-*N*-ethyl-carbodiimide (0.38 g, 2.00 mmol) were added to the mixture. The mixture was warmed to 25 °C and then stirred for 4 h. After that the mixture was quenched with 2M HCl (10 ml) and extracted with CH_2Cl_2 (3x 20 ml). The organic layer was washed with saturated $NaHCO_3$ (25 ml) and brine (25 ml). The product was dried over $MgSO_4$ and concentrated in vacuo. The crude product was purified by column chromatography (ethyl acetate). The obtained product was a yellow oil (0.32 g, 0.70 mmol, 70 %). R_f: 0.49 (ethyl acetate). ^{1}H-NMR ($CDCl_3$, 400 MHz): δ_H: 0.92-0.96 (m, 3H, 1-CH_3); 0.97 (s, 3H, 19-CH_3); 1.03 (s, 3H, 18-CH_3); 1.41 (s, 3H, 22-CH_3); 1.46 (s, 3H, 21-CH_3); 1.50 (m, 2H, 2-CH_2); 2.19 (dq, *J* = 1.5, 7.3 Hz, 2H, 3-CH_2); 2.42 (t, *J* = 6.3 Hz, 2H, 11-CH_2); 3.08 (t, *J* = 6.3 Hz, 2H, 7-CH_2); 3.27 (d, *J* = 11.6 Hz, 1H, 17a-CH_2); 3.40-3.62 (m, 4H, 8-12-CH_2); 3.68 (d, *J* = 11.6 Hz, 1H, 17b-CH_2); 4.07 (s,1H, 15-CH); 6.10 (bt, *J* = 1.6 Hz, 1H, 5-CH); 6.14 (bt, *J* = 1.5 Hz, 1H, 9-NH); 6.92 (dt, *J* = 6.9, 15.6 Hz, 1H, 4-CH); 7.03 (bt, *J* = 5.9 Hz, 1H, 13-NH). ^{13}C-NMR ($CDCl_3$, 100 MHz): δ_C 13.7 (1-CH_3); 18.7 (19-CH_3); 18.9 (21-CH_3); 21.2 (2-CH_2); 22.1 (18-CH_3); 28.2 (7-CH_2); 29.5 (22-CH_3); 32.9 (16-C); 34.2 (3-CH_2); 34.8 (11-CH_2); 35.9 (12-CH_2); 39.7 (8-CH_2); 71.5 (17-CH_2); 77.3 (15-CH); 99.1 (20-C); 128.4 (5-CH); 146.6 (4-CH); 170.0 (10-CO); 171.2 (14-CO); 190.2 (6-CO). ESMS: *m*/*z* (%): 415.4 [M]H$^+$ (100 %). IR (ν_{max} / cm^{-1}): 3306 (NH), 2960 (CH_2), 2872 (CH_2), 1655 (CO), 1523 (C-O), 1461 (C=C). **HRMS:** *m*/*z* (%): calculated 437.2086, found 437.2088 for [M]Na+.

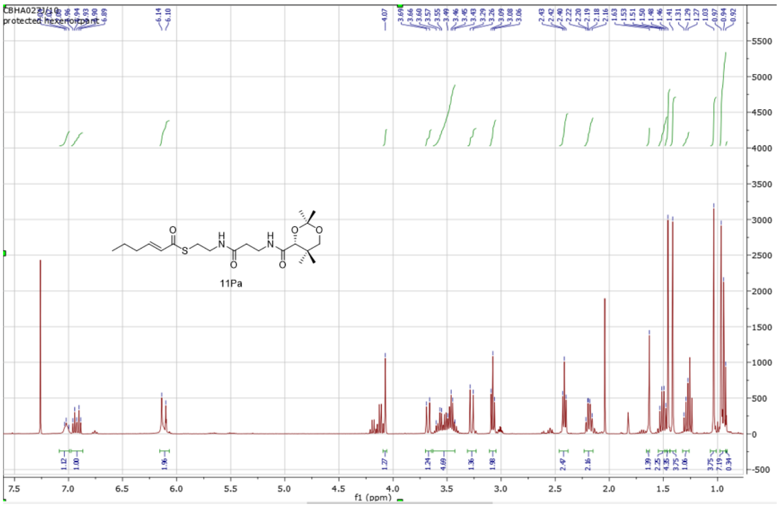

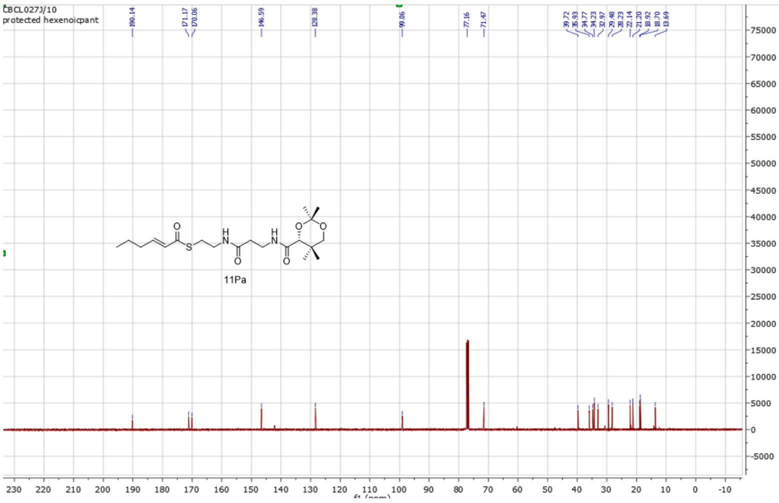

_E_-2-methyl hexenoic pantetheine dimethyl ketal 12Pa

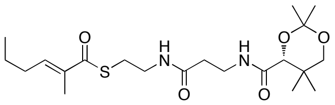

Chemical Formula: $C_{21}H_{36}N_2O_5S$
Exact Mass: 428.23

E-2-Methylhex-2-enoic acid (**12**, 0.13 g, 1 mmol) and pantetheine dimethyl ketal (0.32 g, 1.00 mmol) were dissolved in CH_2Cl_2 (8 ml). The mixture was cooled to 0 °C. Then *N*,*N*-dimethylaminopyridine (0.10 g, 0.80 mmol) and *N*-(3-Diethylaminopropyl)-*N*-ethylcarbodiimide (2.00 mmol, 0.38 g) were added. The mixture was warmed to 25 °C and stirred for 8 h. After that the mixture was quenched with 2M HCl (10 ml) and extracted with CH_2Cl_2 (3 × 25 ml). The organic layer was washed with saturated $NaHCO_3$ (25 ml) and brine (25 ml). The product was dried over $MgSO_4$ and concentrated in vacuo. The crude product was purified by column chromatography (ethyl acetate). The obtained product was a yellow oil (0.38 g, 0.88 mmol, 88 %). R_f: 0.45 (ethyl acetate). ^{1}H-NMR ($CDCl_3$, 400 MHz): δ H 0.96 (t, *J* = 7.6 Hz, 3H, 1-CH_3); 0.97 (s, 3H, 17-CH_3); 1.04 (s, 3H, 18-CH_3); 1.44-1.55 (m, 2H, 2-CH_2); 1.41 (s, 3H, 20-CH_3); 1.46 (s, 3H, 21-CH_3); 1.87 (s, 3H, 6-CH_3); 2.20 (q, *J* = 7.4 Hz, 2H, 3-CH_2); 2.42 (t, *J* = 6.1 Hz, 2H, 10-CH_2); 3.05 (t, *J* = 6.5 Hz, 2H, 8-CH_2); 3.27 (d, *J* = 11.2 Hz, 1H, 16a-CH_2); 3.39-3.62 (m, 4H, 9-12-CH_2); 3.68 (d, *J* = 11.7 Hz, 1H, 16b-CH_2); 4.07 (s,1H, 14-CH); 6.10 (bt, *J* = 5.7 Hz, 1H, 10-NH); 6.77 (dt, *J* = 1.3, 6.9 Hz, 1H, 4-CH); 7.03 (bt, *J* = 5.7 Hz, 1H, 13-NH). ^{13}C-NMR ($CDCl_3$, 100 MHz): δ_C 12.5 (6-CH_3); 13.9 (1-CH_3); 18.7 (17-CH_3); 18.9 (18-CH_3); 21.8 (2-CH_2); 22.1 (20-CH_3); 28.4 (3-CH_2); 28.8 (8-CH_2); 29.5 (21-CH_3); 30.9 (7-CH_2); 32.9 (15-C); 34.8 (11-CH_2); 35.9 (12-CH_2); 39.7 (9-CH_2); 71.5 (16-CH_2); 77.2 (14-CH); 99.1 (19-C); 135.9 (5-C); 142.1 (4-CH); 170.0 (10-CO); 171.2 (13-CO); 193.7 (7-CO). ESMS: *m*/*z* (%) 429.7 [M]H$^+$ (100 %). IR (ν_{max} / cm^{-1}): 3293 (NH), 2959 (CH_2), 2870 (CH_2), 1648 (CO), 1538 (C-O), 1449 (C=C). HRMS: *m*/*z* (%): calculated 451.2243, found 451.2244 for [M]Na+.

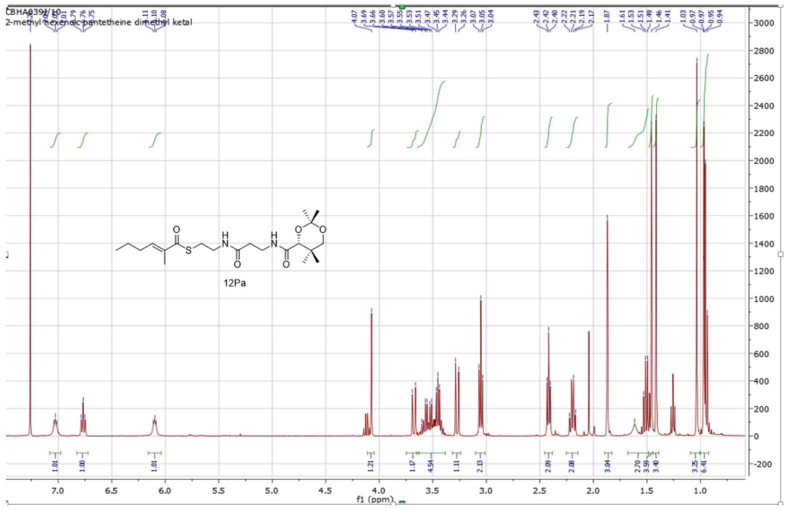

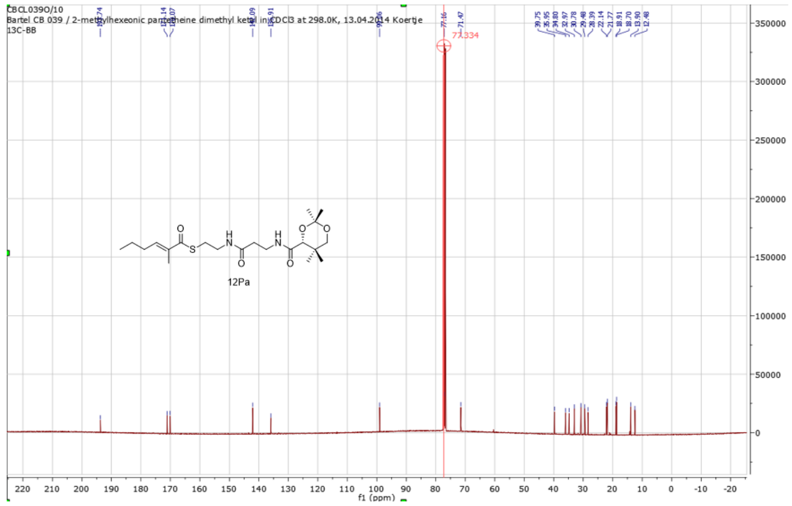

***E*-4*RS*-4-methylhex-2-enoic pantetheine dimethyl ketal 13Pa**

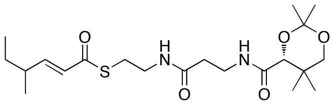

Chemical Formula: $C_{21}H_{36}N_2O_5S$
Exact Mass: 428.23

4*RS*-4-Methylhex-2-enoic acid (**13**, 0.13 g, 1.00 mmol) and pantetheine dimethyl ketal (0.33 g, 1.00 mmol) were dissolved in CH_2Cl_2 (8 ml). The mixture was cooled to 0 °C. Then *N*,*N*-dimethylaminopyridine (0.10 g, 0.80 mmol) and *N*-(3-Diethylaminopropyl)-*N*-ethylcarbodiimide (0.38 g, 2.00 mmol) were added. The mixture was warmed to 25 °C and stirred for 8 h. After that the mixture was quenched with 2M HCl (10 ml) and extracted with CH_2Cl_2 (3x 20 ml). The organic layer was washed with saturated $NaHCO_3$ (20 ml) and brine (20 ml). The product was dried over $MgSO_4$ and concentrated *in vacuo*. The crude product was purified by column chromatography (ethyl acetate). The obtained product was a colourless oil (0.35 g, 0.82 mmol, 82 %). R_f: 0.45 (ethyl acetate). ^{1}H-NMR ($CDCl_3$, 400 MHz): δ_H 0.88 (t, *J* = 7.2 Hz, 3H, 1-CH_3); 0.97 (s, 3H, 17-CH_3); 1.04 (s, 3H, 2-CH_3); 1.06 (d, *J* = 6.7 Hz, 1H, 4-CH_3), 1.40-1.45 (m, 2H, 2-CH_2); 1.41 (s, 3H, 20-CH_3); 1.46 (s, 3H, 21-CH_3); 2.17-2.26 (m, 1H, 3-CH); 2.42 (t, *J* = 6.3 Hz, 2H, 11-CH_2); 3.05 (t, *J* = 6.3 Hz, 2H, 8-CH_2); 3.27 (d, *J* = 11.9 Hz, 1H, 16a-CH_2); 3.40-3.62 (m, 4H, 9-12-CH_2); 3.68 (d, *J* = 11.7 Hz, 1H, 16b-CH_2); 4.07 (s,1H, 14-CH); 6.08 (dd, *J* = 1.2, 15.6 Hz, 1H, 6-CH); 6.12 (bt, *J* = 5.1 Hz, 1H, 10-NH); 6.77 (dd, *J* = 7.5, 15.5 Hz, 1H, 5-CH); 7.03 (bt, *J* = 6.0 Hz, 1H, 13-NH). ^{13}C-NMR ($CDCl_3$, 100 MHz): δ_C 11.6 (1-CH_3); 18.7 (18-CH_3); 18.9 (4-CH_3); 19.1 (21-CH_3); 22.1 (17-CH_3); 28.3 (2-CH_2); 28.4 (8-CH_2); 28.7 (2-CH_2); 29.5 (21-CH_3); 32.9 (15-C); 34.8 (11-CH_2); 35.9 (12-CH_2); 38.1 (3-CH); 39.7 (9-CH_2); 71.5 (16-CH_2); 77.2 (14-CH); 99.1 (19-C); 126.7 (6-CH); 151.7 (5-CH); 170.0 (10-CO); 171.2 (13-CO); 190.3 (7-CO). ESMS: *m*/*z* (%): 429.7 [M]H$^+$ (100 %). IR (ν_{max} / cm^{-1}): 3305 (NH), 2961 (CH_2), 2873 (CH_2), 1655 (CO), 1522 (C-O), 1460 (C=C). HRMS: *m*/*z* (%): calculated 451.2243, found 451.2243 for [M]Na+.

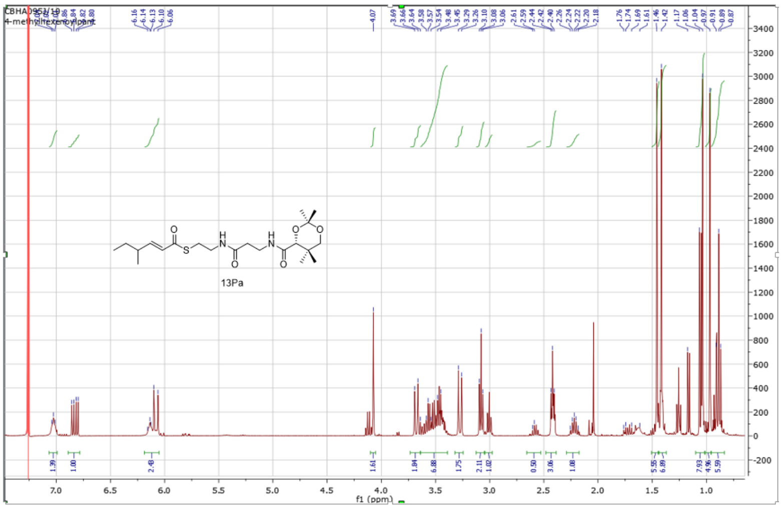

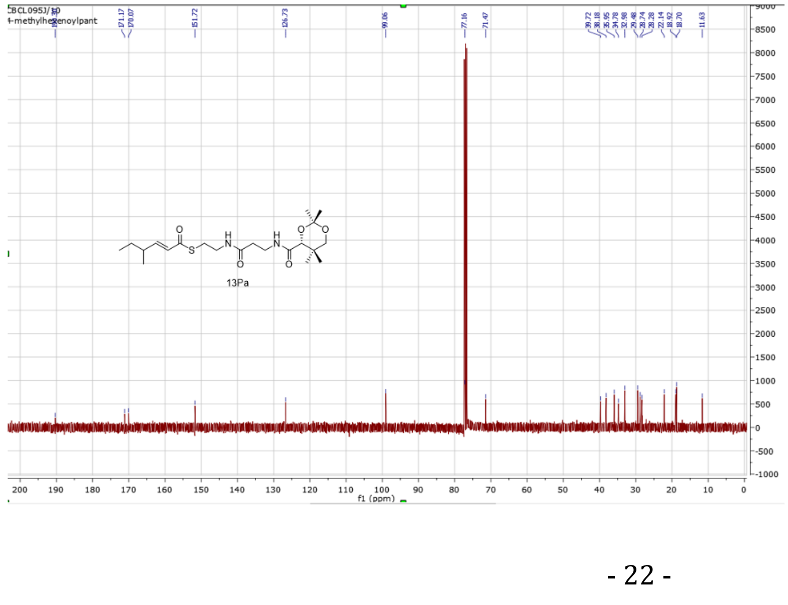

_E_-2_R_S-2,4-dimethylhex-2-enoic pantetheine dimethyl ketal 14Pa

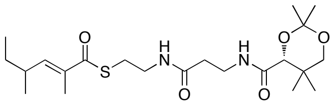

Chemical Formula: $C_{22}H_{38}N_2O_5S$
Exact Mass: 442.25

2*RS*-2,4-dimethylhex-2-enoic acid (**14**, 0.14 g, 1.00 mmol) and pantetheine dimethyl ketal (0.32 g, 1.00 mmol) were dissolved in CH_2Cl_2 (8 ml). The mixture was cooled to 0 °C. Then *N*,*N*-dimethylaminopyridine (0.10 g, 0.80 mmol) and *N*-(3-Diethylamino-propyl)-*N*-ethylcarbodiimide (0.38 g, 2.00 mmol) were added. The mixture was warmed to 25 °C and stirred for 8 h. After that the mixture was quenched with 2M HCl (10 ml) and extracted with CH_2Cl_2 (3x 25 ml). The organic layer was washed with saturated $NaHCO_3$ (20 ml) and brine (20 ml). The product was dried over $MgSO_4$ and concentrated *in vacuo*. The crude product was purified by column chromatography (ethyl acetate). The obtained product was a colourless oil (0.38 g, 0.89 mmol, 89 %). R_f 0.45 (ethyl acetate). ^{1}H-NMR ($CDCl_3$, 400 MHz): δ_H 0.87 (t, *J* = 7.9 Hz, 3H, 1-CH_3); 0.97 (s, 3H, 17-CH_3); 1.04 (s, 3H, 18-CH_3); 1.03 (d, *J* = 7.5 Hz, 1H, 4-CH_3), 1.24-1.49 (m, 2H, 2-CH_2); 1.41 (s, 3H, 21-CH_3); 1.46 (s, 3H, 22-CH_3); 1.88 (s, 3H, 7-CH_3);2.42 (t, *J* = 5.9 Hz, 2H,12-CH_2); 3.05 (t, *J* = 6.4 Hz, 2H, 9-CH_2); 3.27 (d, *J* = 11.4 Hz, 1H, 19a-CH_2); 3.39-3.64 (m, 4H, 10-13-CH_2); 3.68 (d, *J* = 11.7 Hz, 1H, 19b-CH_2); 4.07 (s,1H, 15-CH); 6.09 (bt, *J* = 5.4 Hz, 1H, 11-NH); 6.53 (dd, *J* = 1.4, 9.8 Hz, 1H, 5-CH); 7.03 (bt, *J* = 5.8 Hz, 1H, 14-NH). ^{13}C-NMR ($CDCl_3$, 100 MHz): δ_C 11.9 (1-CH_3); 12.2 (4-CH_3); 18.7 (18-CH_3); 18.9 (21-CH_3); 22.1 (17-CH_3); 28.5 (2-CH_2); 29.5 (22-CH_3); 29.6 (9-CH_2); 32.9 (16-C); 34.4 (12-CH_2); 35.0 (3-CH); 35.9 (13-CH_2); 39.7 (10-CH_2); 71.5 (19-CH_2); 77.2 (15-CH); 99.1 (20-C); 134.5 (6-C); 147.6 (5-CH); 170.0 (11-CO); 171.2 (14-CO); 193.9 (8-CO). ESMS: *m*/*z* (%): 443.7 [M]H$^+$(100 %). IR (ν_{max} / cm^{-1}): 3291 (NH), 2923 (CH_2), 2872 (CH_2), 1651 (CO), 1544 (C-O), 1444 (C=C). HRMS: *m*/*z* (%): calculated 465.2399, found 465.2396 for [M]Na+.

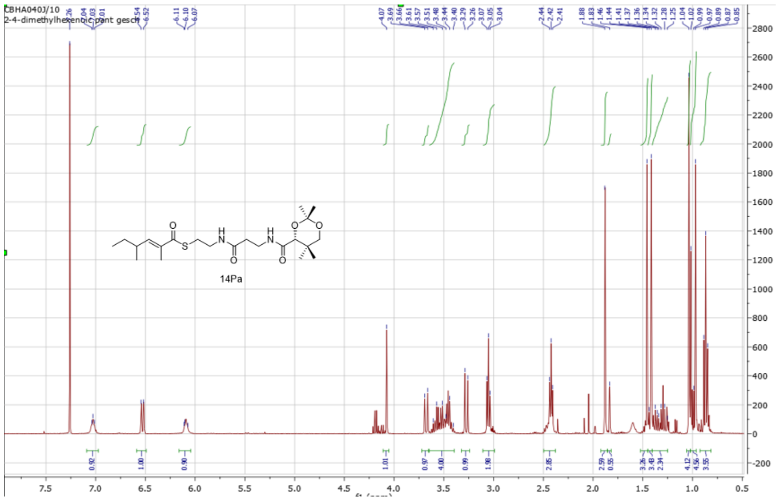

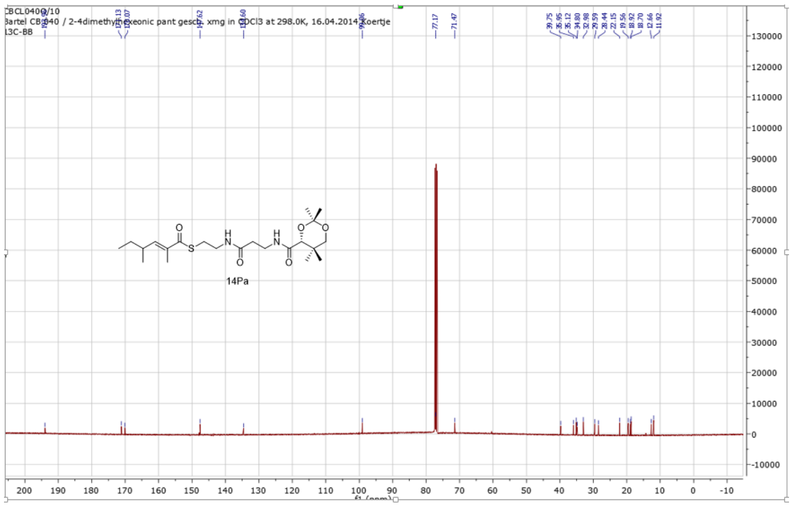

E-4S-2,4-dimethylhex-2-enoic pantetheine dimethyl ketal 15Pa

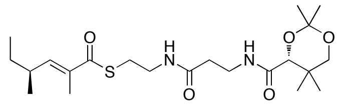

Chemical Formula: $C_{22}H_{38}N_2O_5S$
Exact Mass: 442.25

E-4*S*-2,4-dimethylhex-2-enoic acid (**15**, 0.14 g, 1.00 mmol) and pantetheine dimethyl ketal (0.32 g, 1.00 mmol) were dissolved in CH_2Cl_2 (8 ml). The mixture was cooled to 0 °C. Then *N*,*N*-dimethylaminopyridine (0.10 g, 0.80 mmol) and *N*-(3-Diethylamino-propyl)-*N*-ethylcarbodiimide (0.38 g, 2.00 mmol) were added. The mixture was warmed to 25 °C and stirred for 8 h. After that the mixture was quenched with 2M HCl (10 ml) and extracted with CH_2Cl_2 (3x 25 ml). The organic layer was washed with saturated $NaHCO_3$ (20 ml) and brine (20 ml). The product was dried over $MgSO_4$ and concentrated in vacuo. The crude product was purified by column chromatography (ethyl acetate). The obtained product was a colourless oil (0.30 g, 0.68 mmol, 68 %). R_f 0.45 (ethyl acetate). ^{1}H-NMR ($CDCl_3$, 400 MHz): δ_H 0.91 (t, J = 7.9 Hz, 3H, 1-CH_3); 0.97 (s, 3H, 17-CH_3); 1.04 (s, 3H, 18-CH_3); 1.17 (d, J = 7.5 Hz, 1H, 4-CH_3), 1.24-1.49 (m, 2H, 2-CH_2); 1.42 (s, 3H, 21-CH_3); 1.46 (s, 3H, 22-CH_3); 1.88 (s, 3H, 7-CH_3); 2.42 (t, J = 6.1 Hz, 2H,12-CH_2); 2.54-2.62 (m, 1H, 3-CH); 3.05 (t, J = 6.5 Hz, 2H, 9-CH_2); 3.27 (d, J = 11.7 Hz, 1H, 19a-CH_2); 3.38-3.62 (m, 4H, 10-13-CH_2); 3.68 (d, J = 12.1 Hz, 1H, 19b-CH_2); 4.08 (s,1H, 15-CH); 6.09 (bt, J = 5.4 Hz, 1H, 11-NH); 6.53 (dd, J = 1.5, 10.2 Hz, 1H, 5-CH); 7.03 (bt, J = 5.8 Hz, 1H, 14-NH). ^{13}C-NMR ($CDCl_3$, 100 MHz): δ_C 11.6(1-CH_3); 17.2 (4-CH_3); 18.7 (18-CH_3); 18.9 (21-CH_3); 22.1 (17-CH_3); 28.5 (2-CH_2); 29.5 (22-CH_3); 29.6 (9-CH_2); 32.9 (16-C); 34.8 (12-CH_2); 35.1 (3-CH); 35.9 (13-CH_2); 39.7 (10-CH_2); 71.5 (19-CH_2); 77.2 (15-CH); 99.1 (20-C); 135.6 (6-C); 147.6 (5-CH); 170.1 (11-CO); 171.1 (14-CO); 204.1 (8-CO). ESMS: *m/z* (%): 443.7 $[M]H^+$ (100 %). IR (ν_{max} / cm^{-1}): 3302 (NH), 2960 (CH_2), 2873 (CH_2), 1649 (CO), 1521 (C-O), 1460 (C=C). HRMS: *m/z* (%): calculated 443.2580, found 443.2853 for [M]H+.

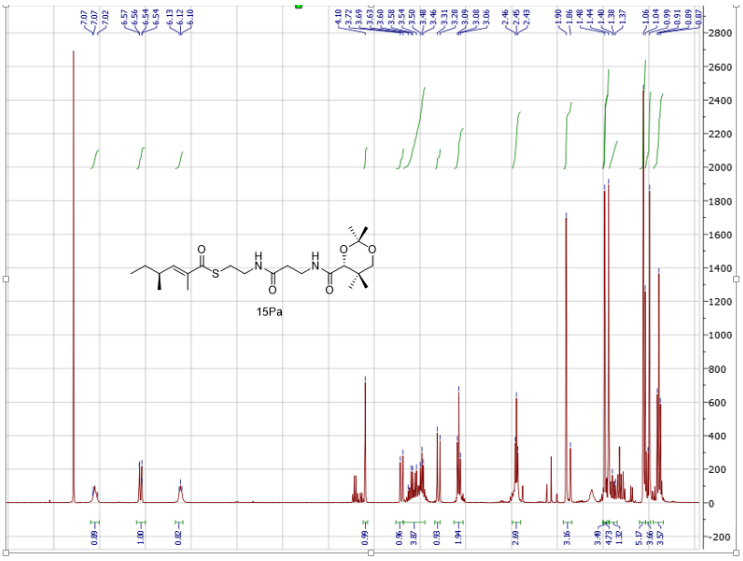

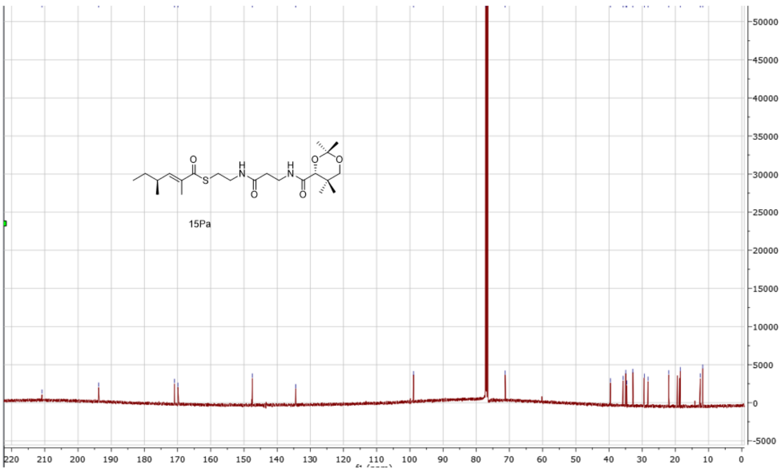

E-2-ethylhexenoic pantetheine dimethyl ketal 16Pa

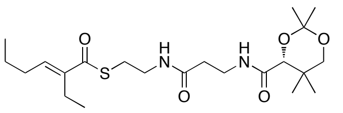

E-2-Ethylhex-2-enoic acid (**16**, 0.13 g, 1 mmol) and pantetheine dimethyl ketal (0.32 g, 1.00 mmol) were dissolved in CH_2Cl_2 (8 ml). The mixture was cooled to 0 °C. Then *N*,*N*-dimethylaminopyridine (0.10 g, 0.80 mmol) and *N*-(3-Diethylaminopropyl)-*N*-ethylcarbodiimide (2.00 mmol, 0.38 g) were added. The mixture was warmed to 25 °C and stirred for 8 h. After that the mixture was quenched with 2M HCl (10 ml) and extracted with CH_2Cl_2 (3x 25 ml). The organic layer was washed with saturated $NaHCO_3$ (25 ml) and brine (25 ml). The product was dried over $MgSO_4$ and concentrated in vacuo. The crude product was purified by column chromatography (ethyl acetate). The obtained product was a yellow oil (0.38 g, 0.88 mmol, 88 %). R_f: 0.45 (ethyl acetate). ^{1}H-NMR ($CDCl_3$, 400 MHz): δ_H 0.86-1.08 (m, 1-7-18-19-CH_3); 1.40-1.53 (m, 2H, 2-CH_2); 1.41 (s, 3H, 21-CH_3); 1.46 (s, 3H, 22-CH_3); 2.20 (q, *J* = 7.5 Hz, 2H, 3-CH_2); 2.31-2.38 (m, 2H, 11-CH_{2E}); 2.40-2.44 (m, 2H, 6-CH_{2Z}); 3.03-3.09 (m, 2H, 9-CH_2); 3.27 (d, *J* = 11.2 Hz, 1H, 17a-CH_2); 3.39-3.64 (m, 4H, 10-13-CH_2); 3.68 (d, *J* = 11.7 Hz, 1H, 17b-CH_2); 4.07 (s,1H, 15-CH); 5.62 (t, *J* = 7.7 Hz, 1H, 4-CH_Z); 6.12 (bt, *J* = 5.7 Hz, 1H, 11-NH); 6.71 (t, *J* = 7.5 Hz, 1H, 4-CH_E); 7.04 (bt, *J* = 5.7 Hz, 1H, 14-NH). ^{13}C-NMR ($CDCl_3$, 100 MHz): δ_C 13.9 (1-CH_3); 14.1 (22-CH_3); 18.7 (18-CH_3); 18.9 (22-CH_3); 20.5 (6-CH_2); 22.2 (2-CH_2); 22.1 (19-CH_3); 28.4 (9-CH_2); 28.8 (3-CH_2); 29.5 (21-CH_3); 30.9 (3-CH_2); 32.9 (15-C); 34.8 (12-CH_2); 35.9 (13-CH_2); 39.7 (10-CH_2); 71.5 (17-CH_2); 77.2 (15-CH); 99.1 (20-C); 135.9 (5-C); 142.1 (4-CH); 170.0 (11-CO); 171.2 (14-CO); 193.7 (8-CO). ESMS: *m/z* (%) 465.4 $[M]Na^+$ (51 %), 443.7 $[M]H^+$ (28 %), 385.4 $[M - C_4H_8]H^+$ (100 %). HRMS: *m/z* (%): calculated 465.2399, found 465.2396 for [M]Na+.

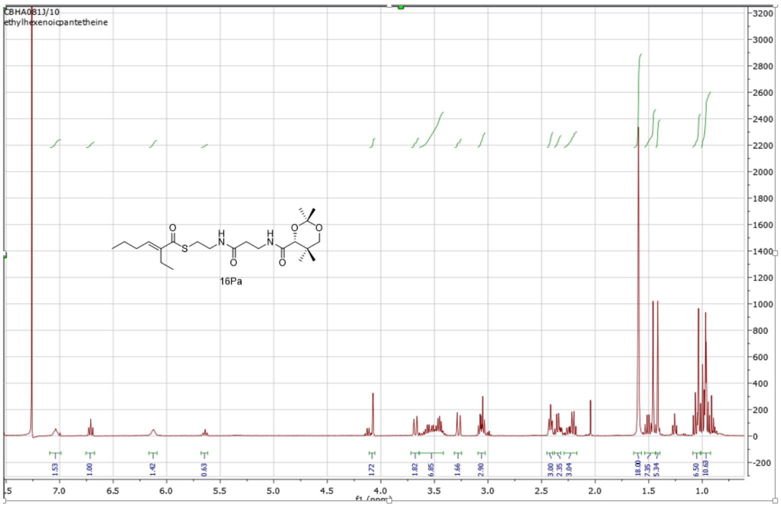

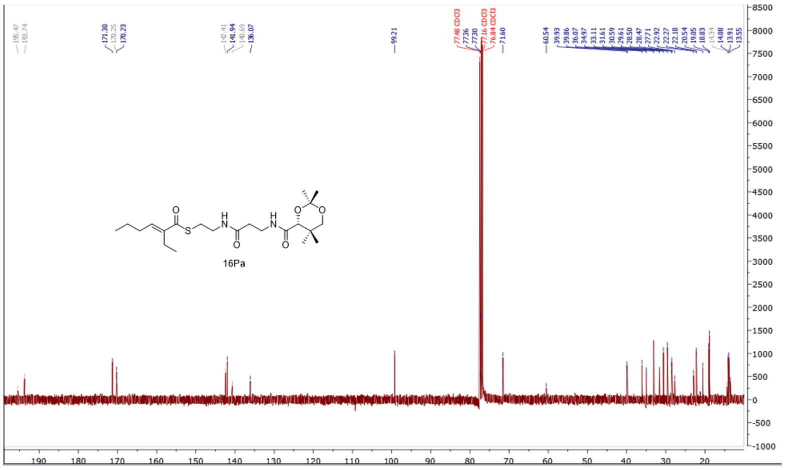

***E*-6*RS*-6-methyl hept-2-enoic pantetheine dimethyl ketal 18Pa**

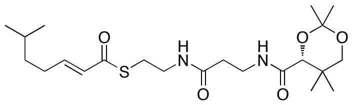

Chemical Formula: $C_{22}H_{38}N_2O_5S$
Exact Mass: 442.25

E-6*RS*-6-Methyl heptenoic acid (**18**, 0.16 g, 1.00 mmol) and pantetheine dimethyl ketal (0.32 g, 1.00 mmol,) were dissolved in CH_2Cl_2 (8 ml). The mixture was cooled to 0 °C. Then *N*,*N*-dimethylaminopyridine (0.10 g, 0.80 mmol,) and *N*-(3-Diethylamino-propyl)-*N*-ethylcarbodiimide (0.38 g, 2.00 mmol) were added. The mixture was warmed to 25 °C and stirred for 4 h. After that the mixture was quenched with 2M HCl (10 ml) and extracted with CH_2Cl_2 (3x 25 ml). The organic layer was washed with saturated $NaHCO_3$ (20 ml) and brine (20 ml). The product was dried over $MgSO_4$ and concentrated in vacuo. The crude product was purified by column chromatography (ethyl acetate). The obtained product was a colourless oil (0.22 g, 0.52 mmol, 52 %). R_f: 0.53 (ethyl acetate). ^{1}H-NMR ($CDCl_3$, 400 MHz): δ_H 0.92 (d, *J* = 6.7 Hz, 6H, 1-3-CH_3); 0.97 (s, 3H, 20-CH_3); 1.04 (s, 3H, 19-CH_3); 1.32-1.38 (m, 2H, 4-CH_2); 1.42 (s, 3H, 22-CH_3); 1.46 (s, 3H, 21-CH_3); 2.22 (dq, *J* = 1.9, 7.1 Hz, 2H, 5-CH_2); 2.42 (t, *J* = 6.1 Hz, 2H,12-CH_2); 3.08 (t, *J* = 6.4 Hz, 2H, 9-CH_2); 3.28 (d, *J* = 11.7 Hz, 1H, 17a-CH_2); 3.40-3.62 (m, 4H, 10-13-CH_2); 3.68 (d, *J* = 11.6 Hz, 1H, 17b-CH_2); 4.07 (s,1H, 15-CH); 6.10 (bt, *J* = 1.6 Hz, 1H, 11-NH); 6.14 (t, *J* = 1.5 Hz, 1H, 7-CH); 6.93 (dt, *J* = 7.1, 15.6 Hz, 1H, 6-CH); 7.02 (bt, *J* = 6.0 Hz, 1H, 14-NH). ^{13}C-NMR ($CDCl_3$, 100 MHz): δ_C 18.7 (19-CH_3); 18.9 (21-CH_3); 22.1 (20-CH_3); 22.4 (1-CH_3); 22.4 (3-CH_3); 27.6 (2-CH); 28.2 (9-CH_2); 29.5 (22-CH_3); 30.1 (5-CH_2); 32.9 (16-C); 34.8 (12-CH_2); 35.9 (13-CH_2); 36.9 (4-CH_2); 39.7 (10-CH_2); 71.5 (17-CH_2); 77.2 (15-CH); 99.1 (18-C); 128.2 (7-CH); 147.1 (6-CH); 170.1 (11-CO); 171.4 (14-CO); 190.2 (8-CO). ESMS: *m*/*z* (%): 465.3 $[M]Na^+$ (28 %), 443.3 $[M]H^+$ (12 %), 385 $[M + H - C_3H_6O]$+ (100 %). IR (ν_{max} / cm^{-1}): 3303 (NH), 2953 (CH_2), 2870 (CH_2), 1655 (CO), 1524 (C-O), 1465 (C=C). HRMS: *m*/*z* (%): calculated 465.2399, found 465.2396 for [M]Na+.

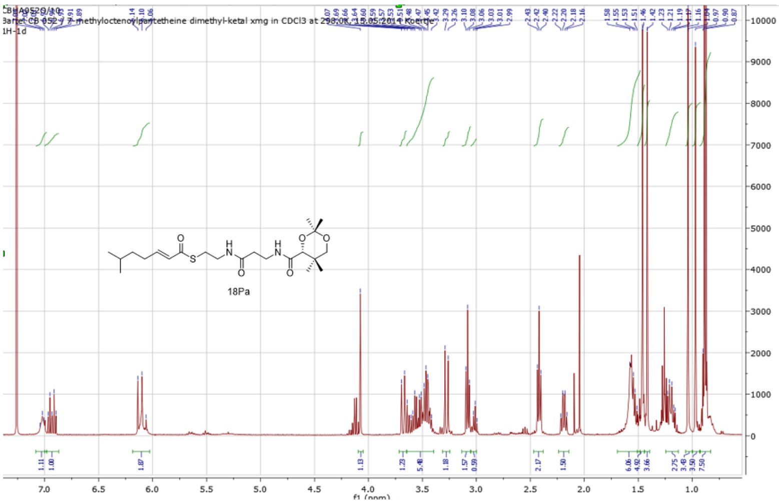

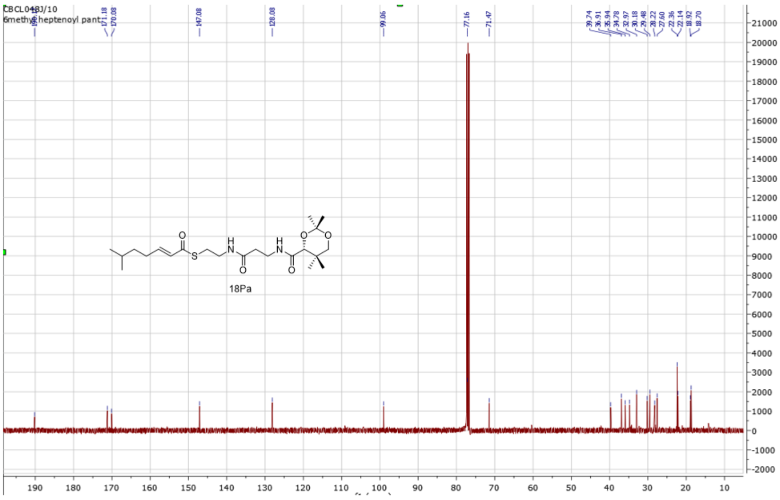

E-2-methyloct-2-enoic pantetheine dimethyl ketal 20Pa

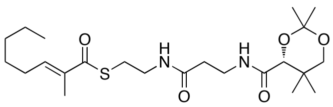

Chemical Formula: $C_{23}H_{40}N_2O_5S$
Exact Mass: 456.27

E-2-Methyloct-2-enoic acid (**20**, 0.16 g, 1.00 mmol) and pantetheine dimethyl ketal (0.32 g, 1.00 mmol) were dissolved in CH_2Cl_2 (8 ml). The mixture was cooled to 0 °C. Then *N*,*N*-dimethylaminopyridine (0.10 g, 0.80 mmol) and *N*-(3-Diethyl-aminopropyl)-*N*-ethylcarbodiimide (0.38 g, 2.00 mmol) were added. The mixture was warmed to 25 °C and stirred for 4 h. After that the mixture was quenched with 2M HCl (10 ml) and extracted with CH_2Cl_2 (3x 25 ml). The organic layer was washed with saturated $NaHCO_3$ (20 ml) and brine (20 ml). The product was dried over $MgSO_4$ and concentrated in vacuo. The crude product was purified by column chromatography (ethyl acetate). The obtained product was a colourless oil (0.27 g, 0.60 mmol, 60 %). R_f: 0.47 (ethyl acetate). ^{1}H-NMR ($CDCl_3$, 400 MHz): δ_H 0.90 (t, J = 7.4 Hz, 3H, 1-CH_3); 0.97 (s, 3H, 21-CH_3); 1.04 (s, 3H, 19-CH_3); 1.31-1.51 (m, 6H, 2-3-4-CH_2); 1.41 (s, 3H, 25-CH_3); 1.46 (s, 3H, 24-CH_3); 1.87 (s, 3H, 8-CH_3); 2.21 (g, J = 7.3 Hz, 2H, 5-CH_2); 2.42 (t, J = 5.8 Hz, 2H, 14-CH_2); 3.05 (t, J = 6.7 Hz, 2H, 10-CH_2); 3.27 (d, J = 11.8 Hz, 1H, 22a-CH_2); 3.39-3.62 (m, 4H, 11-15-CH_2); 3.68 (d, J = 12.2 Hz, 1H, 22b-CH_2); 4.07 (s,1H, 18-CH); 6.08 (bt, J = 5.8 Hz, 1H, 12-NH); 6.92 (dt, J = 1.5, 7.4 Hz, 1H, 6-CH); 7.03 (bt, J = 5.8 Hz, 1H, 16-NH). ^{13}C-NMR ($CDCl_3$, 100 MHz): δ_C 12.5 (8-CH_3); 13.9 (1-CH_3); 18.7 (19-CH_3); 18.9 (21-CH_3); 22.1 (24-CH_3); 22.5 (2-CH_3); 28.2 (4-CH_2); 28.4 (3-CH_2); 28.8 (10-CH_2); 29.5 (25-CH_3); 31.6 (5-CH_2); 32.9 (20-C); 34.8 (14-CH_2); 35.9 (15-CH_2); 39.7 (11-CH_2); 71.5 (22-CH_2); 77.2 (18-CH); 99.1 (23-C); 135.7 (7-C); 142.4 (6-CH); 170.0 (13-CO); 171.1 (17-CO); 193.7 (9-CO). ESMS: m/z (%): 457.7 $[M]H^+$ (100 %). IR (ν_{max} / cm^{-1}): 3306 (NH), 2929 (CH_2), 2860 (CH_2), 1652 (CO), 1523 (C-O), 1461 (C=C). HRMS: m/z (%): calculated 479.2556, found 479.2552 for [M]Na+.

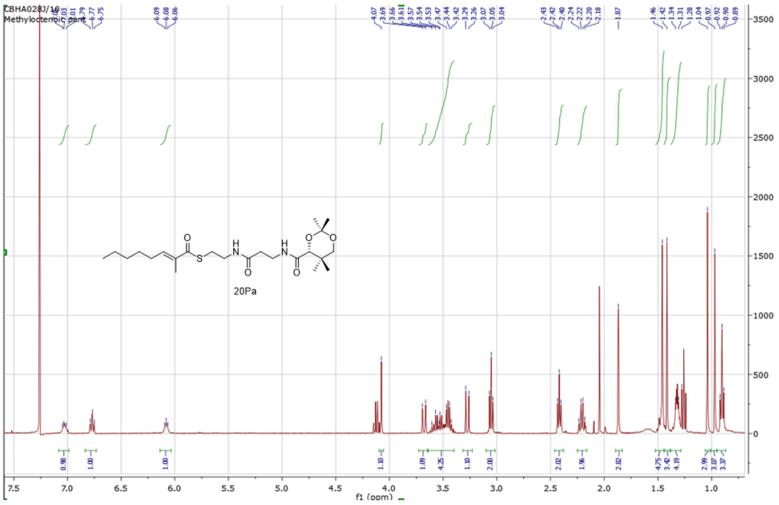

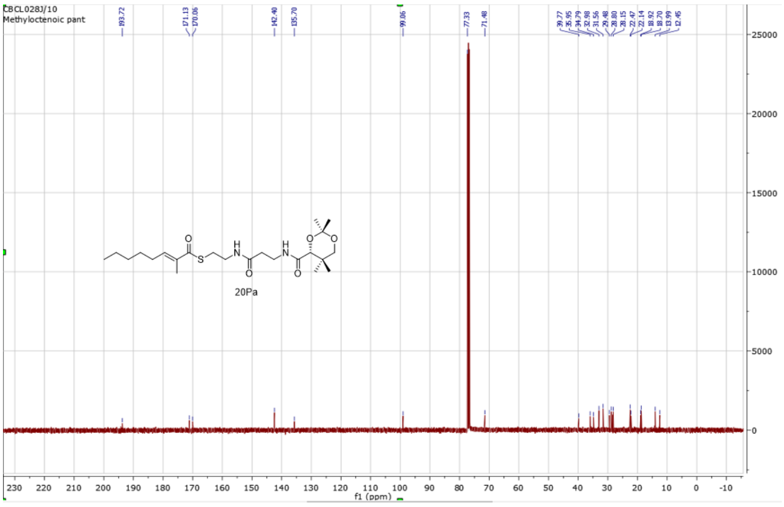

***E*-7-methyloct-2-enoic pantetheine dimethyl ketal 21Pa**

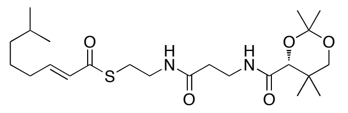

Chemical Formula: $C_{23}H_{40}N_2O_5S$
Exact Mass: 456.27

E-7-Methyloct-2-enoic acid (**21**, 0.16 g, 1.00 mmol) and pantetheine dimethyl ketal (0.32 g, 1.00 mmol,) were dissolved in CH_2Cl_2 (8 ml). The mixture was cooled to 0 °C. Then *N*,*N*-dimethylaminopyridine (0.10 g, 0.80 mmol,) and *N*-(3-Diethylamino-propyl)-*N*-ethylcarbodiimide (0.38 g, 2.00 mmol) were added. The mixture was warmed to 25 °C and stirred for 4 h. After that the mixture was quenched with 2M HCl (10 ml) and extracted with CH_2Cl_2 (3x 25 ml). The organic layer was washed with saturated $NaHCO_3$ (20 ml) and brine (20 ml). The product was dried over $MgSO_4$ and concentrated *in vacuo*. The crude product was purified by column chromatography (ethyl acetate). The obtained product was a colourless oil (0.18 g, 0.40 mmol, 40 %). R_f: 0.43 (ethyl acetate). ^{1}H-NMR ($CDCl_3$, 400 MHz): δ_H 0.89 (d, J = 6.7 Hz, 6H, 1-3-CH_3); 0.97 (s, 3H, 20-CH_3); 1.04 (s, 3H, 19-CH_3); 1.42 (s, 3H, 22-CH_3); 1.46 (s, 3H, 21-CH_3); 1.43-1.62 (m, 5H, 2-CH-3-4-CH_2); 2.22 (dq, J = 1.7, 7.3 Hz, 2H, 5-CH_2); 2.42 (t, J = 6.3 Hz, 2H,12-CH_2); 3.08 (t, J = 6.3 Hz, 2H, 9-CH_2); 3.28 (d, J = 11.7 Hz, 1H, 17a-CH_2); 3.42-3.64 (m, 4H, 10-13-CH_2); 3.68 (d, J = 11.6 Hz, 1H, 17b-CH_2); 4.08 (s,1H, 15-CH); 6.10 (bt, J = 1.6 Hz, 1H, 11-NH); 6.17 (t, J = 1.5 Hz, 1H, 7-CH); 6.93 (dt, J = 6.9, 15.4 Hz, 1H, 6-CH); 7.02 (bt, J = 6.2 Hz, 1H, 14-NH). ^{13}C-NMR ($CDCl_3$, 100 MHz): δ_C 18.7 (19-CH_3); 18.9 (21-CH_3); 22.1 (20-CH_3); 22.4 (1-CH_3); 22.4 (23-CH_3); 25.8 (3-CH_2); 27.8 (2-CH); 28.2 (9-CH_2); 29.5 (22-CH_3); 32.5 (5-CH_2); 32.9 (16-C); 34.8 (12-CH_2); 35.9 (13-CH_2); 39.7 (10-CH_2); 71.5 (17-CH_2); 77.2 (15-CH); 99.1 (18-C); 128.4 (7-CH); 147.1 (6-CH); 170.1 (11-CO); 171.4 (14-CO); 190.2 (8-CO). ESMS: *m*/*z* (%): 479.3 [M]Na$^+$ (5 %), 457.4 [M]H$^+$ (18 %), 399.4 [M - C_3H_6O]+ (100 %). IR (ν_{max} / cm^{-1}): 3307 (NH), 2952 (CH_2), 2869 (CH_2), 1655 (CO), 1524 (C-O), 1463 (C=C). HRMS: *m*/*z* (%): calculated 479.2556, found 479.2557 for [M]Na+.

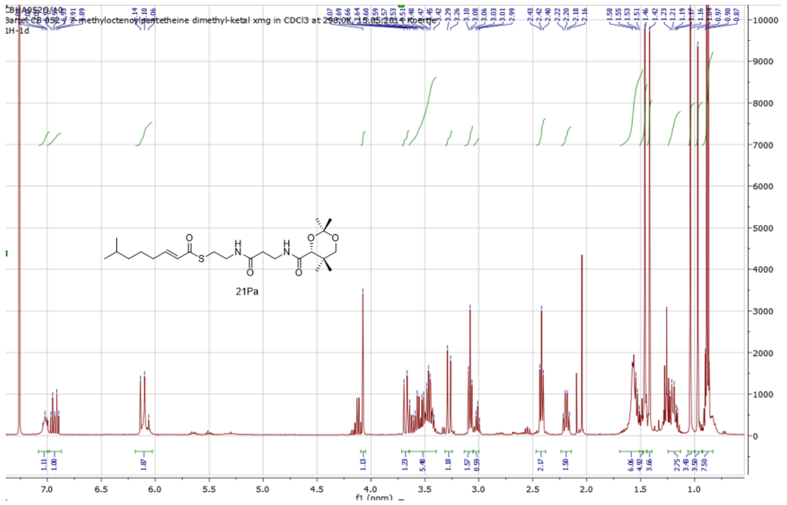

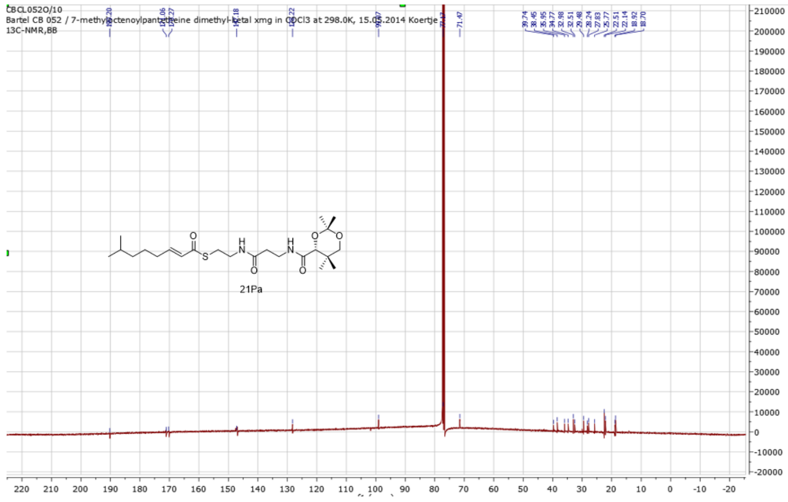

***E*-4*RS*, 6*RS*-4,6-dimethyloct-2-en-oyl-pantetheine 22Pa**

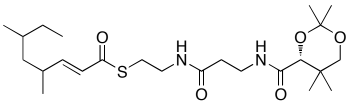

Chemical Formula: $C_{24}H_{42}N_2O_5S$
Exact Mass: 470.28

E-4*RS*,6*RS*-4,6-dimethyloct-2-enoic acid (**22**, 0.17 g, 1.00 mmol) and pantetheine dimethyl ketal (0.32 g, 1.00 mmol,) were dissolved in CH_2Cl_2 (8 ml). The mixture was cooled to 0 °C. Then *N*,*N*-dimethylaminopyridine (0.10 g, 0.80 mmol,) and *N*-(3-Diethylamino-propyl)-*N*-ethylcarbodiimide (0.38 g, 2.00 mmol) were added. The mixture was warmed to 25 °C and stirred for 4 h. After that the mixture was quenched with 2M HCl (10 ml) and extracted with CH_2Cl_2 (3 x 25 ml). The organic layer was washed with saturated $NaHCO_3$ (20 ml) and brine (20 ml). The product was dried over $MgSO_4$ and concentrated in vacuo. The crude product was purified by column chromatography (ethyl acetate). The obtained product was a colourless oil (0.32 g, 0.65 mmol, 65 %). R_f: 0.46 (ethyl acetate). ^{1}H-NMR ($CDCl_3$, 400 MHz): δ_H 0.83 (m, 6H, 1-3-CH_3); 0.97 (s, 3H, 20-CH_3); 1.04 (s, 3H, 21-CH_3); 1.14 (m, , 3H, 7-CH_3), 1.24-1.49 (m, 5H, 2-4-5-CH_2); 1.42 (s, 3H, 23-CH_3); 1.46 (s, 3H, 24-CH_3); 1.71-1.78 (m, 1H, 6-CH); 2.42 (t, *J* = 6.1 Hz, 2H,14-CH_2); 2.54-2.62 (m, 1H, 6-CH); 3.05 (t, *J* = 6.5 Hz, 2H, 11-CH_2); 3.27 (d, *J* = 11.7 Hz, 1H, 19a-CH_2); 3.38-3.62 (m, 4H, 12-15-CH_2); 3.68 (d, *J* = 12.1 Hz, 1H, 19b-CH_2); 4.07 (s,1H, 17-CH); 6.05 (m, 1H, 9-CH); 6.12 (bt, *J* = 5.4 Hz, 1H, 13-NH); 6.74-6.88 (m, 1H, 8-CH); 7.03 (bt, *J* = 5.8 Hz, 1H, 16-NH). ^{13}C-NMR ($CDCl_3$, 100 MHz): δ_C 11.1 (1-CH_3); 11.2 (3-CH_3); 17.6 (7-CH_3); 18.7 (20-CH_3); 18.9 (23-CH_3); 22.1 (21-CH_3); 28.2 (2-CH_2); 29.5 (24-CH_3); 29.6 (11-CH_2); 32.1 (4-CH); 32.9 (18-C); 34.7 (15-CH_2) 35.9 (14-CH_2); 39.7 (12-CH_2); 40.8 (5-CH), 46-5 (6-C) 71.5 (19-CH_2); 77.2 (17-CH); 99.1 (22-C); 126.9 (9-CH); 147.6 (8-CH); 170.1 (13-CO); 171.1 (16-CO); 204.1 (10-CO). ESMS: *m*/*z* (%): 471 [M]H$^+$ (100%) IR (ν_{max} / cm^{-1}): 2958 (CH), 2927 (CH_2), 2872 (CH_2), 1654 (CO), 1629 (CO), 1523 (C-O), 1460 (C=C). HRMS: *m*/*z* (%): calculated 471.2893, found 471.2891 for [M]H+.

E-4S,6S-4,6-dimethyloct-2-enoyl-pantetheine 1Pa

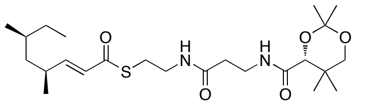

Chemical Formula: $C_{24}H_{42}N_2O_5S$
Exact Mass: 470.28

E-4*S*,6*S*-4,6-dimethyloct-2-enoic acid (**1**, 0.17 g, 1.00 mmol) and pantetheine dimethyl ketal (0.32 g, 1.00 mmol,) were dissolved in CH_2Cl_2 (8 ml). The mixture was cooled to 0 °C. Then *N*,*N*-dimethylaminopyridine (0.10 g, 0.80 mmol,) and *N*-(3-Diethylamino-propyl)-*N*-ethylcarbodiimide (0.38 g, 2.00 mmol) were added. The mixture was warmed to 25 °C and stirred for 4 h. After that the mixture was quenched with 2M HCl (10 ml) and extracted with CH_2Cl_2 (3 x 25 ml). The organic layer was washed with saturated $NaHCO_3$ (20 ml) and brine (20 ml). The product was dried over $MgSO_4$ and concentrated *in vacuo*. The crude product was purified by column chromatography (ethyl acetate). The obtained product was a colourless oil (0.34 g, 0.70 mmol, 70 %). R_f: 0.46 (ethyl acetate). ^{1}H-NMR ($CDCl_3$, 400 MHz): δ_H 0.87 (m, 6H, 1-3-CH_3); 0.97 (s, 3H, 20-CH_3); 1.04 (s, 3H, 21-CH_3); 1.13-1.19 (m, 3H, 7-CH_3), 1.26-1.49 (m, 5H, 2-4-5-CH_2); 1.44 (s, 3H, 23-CH_3); 1.48 (s, 3H, 24-CH_3); 2.45 (t, *J* = 5.9 Hz, 1H, 14-CH); 3.10 (t, *J* = 6.5 Hz, 2H, 11-CH_2); 3.27 (d, *J* = 11.7 Hz, 1H, 19a-CH_2); 3.42-3.62 (m, 4H, 12-15-CH_2); 3.70 (d, *J* = 11.6 Hz, 1H, 19b-CH_2); 4.09 (s,1H, 17-CH); 6.12 (dd, *J* = 1.0, 16.0 Hz, 1H, 9-CH); 6.28 (bt, *J* = 6.3 Hz, 1H, 13-NH); 6.80 (dd, *J* = 8.2,15.8 Hz, 1H, 8-CH); 7.06 (bt, *J* = 5.8 Hz, 1H, 16-NH). ^{13}C-NMR ($CDCl_3$, 100 MHz): δ_C 11.1 (1-CH_3); 14.2 (3-CH_3); 18.7 (20-CH_3); 18.9 (23-CH_3); 22.1 (21-CH_3); 28.2 (2-CH_2); 29.5 (24-CH_3); 29.7 (3-CH_2); 29.7 (11-CH_2); 34.4 (4-CH); 32.9 (18-C); 34.7 (15-CH_2) 35.9 (14-CH_2); 39.7 (12-CH_2); 43-5 (6-C) 71.5 (19-CH_2); 77.2 (17-CH); 99.1 (22-C); 126.6 (9-CH); 152.1 (8-CH); 170.1 (13-CO); 171.1 (16-CO); 190.1 (10-CO). ESMS: *m*/*z* (%): 471 [M]H$^+$ (100%), 413 [M - C_3H_6O]H $^+$ (12%). IR (ν_{max} / cm^{-1}): 2958 (CH), 2927 (CH_2), 2872 (CH_2), 1654 (CO), 1629 (CO), 1523 (C-O), 1460 (C=C). HRMS: *m*/*z* (%): calculated 493.2712 found 493.2619 for [M]Na+.

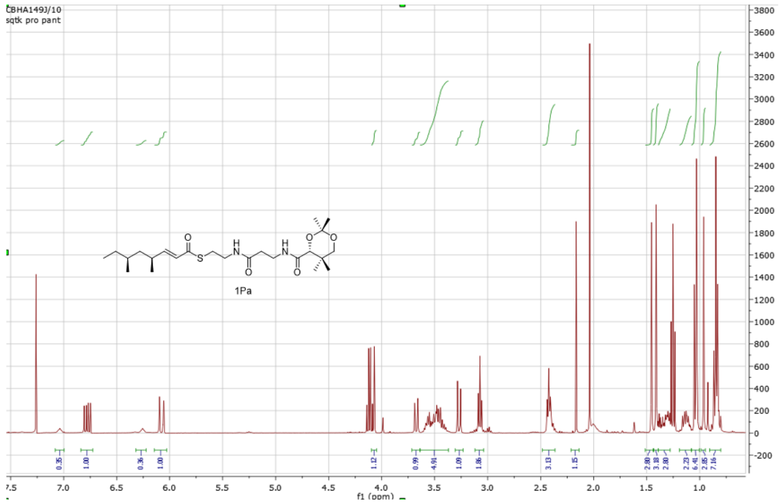

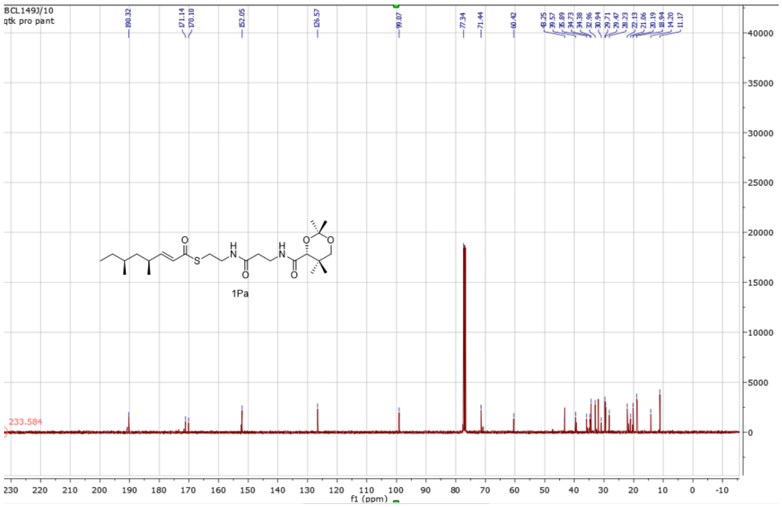

E-2-methyldec-2-enoic pantetheine dimethyl ketal 25Pa

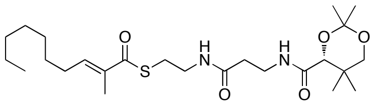

Chemical Formula: $C_{25}H_{44}N_2O_5S$
Exact Mass: 484.30

E-2-Methyldec-2-enoic acid (**25**, 0.184 g, 1.00 mmol) and pantetheine dimethyl ketal (0.32 g, 1.00 mmol) were dissolved in CH_2Cl_2 (8 ml). The mixture was cooled to 0 °C. Then *N*,*N*-dimethylaminopyridine (0.10 g, 0.80 mmol) and *N*-(3-Diethylaminopropyl)-*N*-ethylcarbodiimide (0.38 g, 2.00 mmol) were added. The mixture was warmed to 25 °C and stirred for 4 h. After that the mixture was quenched with 2M HCl (10 ml) and extracted with CH_2Cl_2 (3 x 25 ml). The organic layer was washed with saturated $NaHCO_3$ (20 ml) and brine (20 ml). The product was dried over $MgSO_4$ and concentrated *in vacuo*. The crude product was purified by column chromatography (ethyl acetate). The obtained product was a colourless oil (0.28 g, 0.58 mmol, 58 %). R_f 0.45 (ethyl acetate). ^{1}H-NMR ($CDCl_3$, 400 MHz): δ_H 0.88 (t, J = 7.6 Hz, 3H, 1-CH_3); 0.97 (s, 3H, 23-CH_3); 1.04 (s, 3H, 21-CH_3); 1.30-1.47 (m, 10H, 2-3-4-5-6-CH_2); 1.41 (s, 3H, 27-CH_3); 1.46 (s, 3H, 26-CH_3); 1.87 (s, 3H, 10-CH_3); 2.21 (g, J = 7.1 Hz, 2H, 7-CH_2); 2.42 (t, J = 6.2 Hz, 2H, 13-CH_2); 3.05 (t, J = 6.2 Hz, 2H, 12-CH_2); 3.27 (d, J = 12.2 Hz, 1H, 24a-CH_2); 3.39-3.62 (m, 4H, 13-17-CH_2); 3.68 (d, J = 12.2 Hz, 1H, 24b-CH_2); 4.07 (s,1H, 20-CH); 6.08 (bt, J = 5.0 Hz, 1H, 14-NH); 6.77 (dt, J = 1.9, 6.9 Hz, 1H, 8-CH); 7.03 (bt, J = 5.5 Hz, 1H, 18-NH). ^{13}C-NMR ($CDCl_3$, 100 MHz): δ_C 12.5 (10-CH_3); 14.0 (1-CH_3); 18.7 (23-CH_3); 18.9 (21-CH_3); 22.1 (26-CH_3); 22.6 (2-CH_2); 28.4 (4-CH_2); 28.5 (5-CH_2); 28.8 (12-CH_2); 29.1 (3-CH_2); 29.5 (27-CH_3); 31.8 (6-CH_2); 32.9 (22-C); 34.8 (16-CH_2); 35.9 (17-CH_2); 39.7 (13-CH_2); 71.5 (24-CH_2); 77.2 (20-CH); 99.1 (25-C); 135.7 (9-C); 142.4 (8-CH); 170.0 (15-CO); 171.2 (19-CO); 193.7 (11-CO). ESMS: *m*/*z* (%): 485.7 [M]H$^+$(100 %). IR (ν_{max} / cm^{-1}): 3307 (NH), 2926 (CH_2), 2856 (CH_2), 1652 (CO), 1523 (C-O), 1461 (C=C). HRMS: *m*/*z* (%): calculated 507.2869, found 507.2870 for [M]Na+.

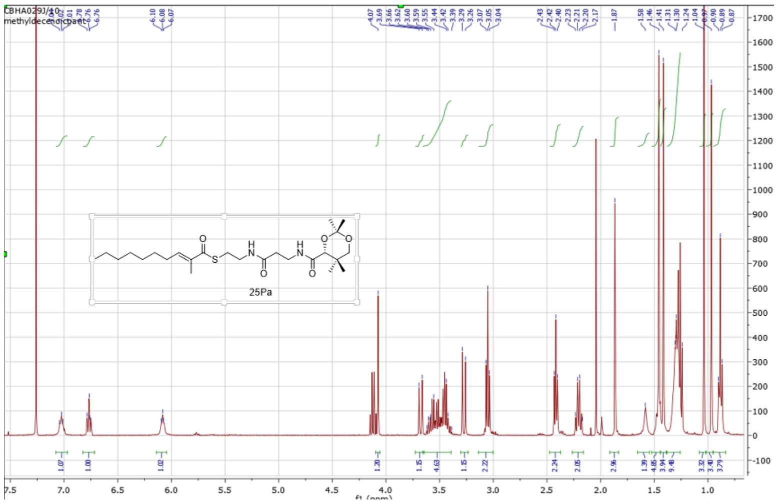

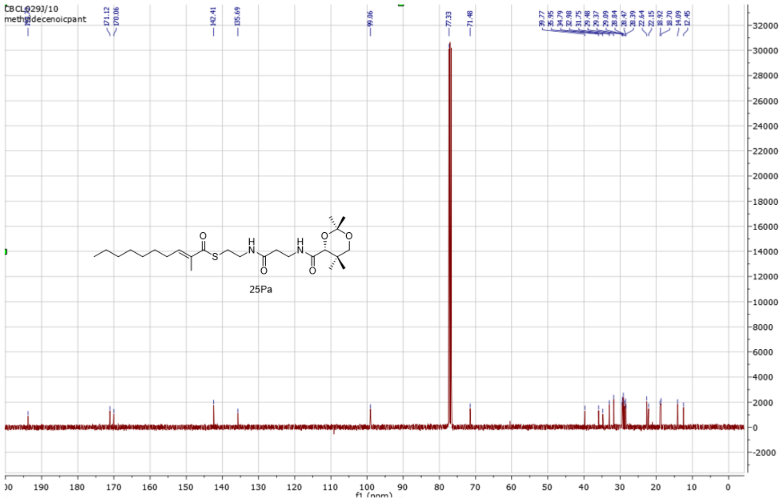

tigloyl-pantetheine 5P

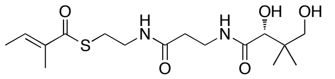

Chemical Formula: $C_{16}H_{28}N_2O_5S$
Exact Mass: 360.17

A solution of pantetheine (0.45 g, 0.80 mmol) and DTT (0.12 g, 0.80 mmol) in THF (4 ml) was stirred in a nitrogen atmosphäre at 30 °C for 3 h. After that triethylamine (0.3 ml) and DMAP (0.09 mmol, 12 mg) were added to the reaction and stirred for 15 min. Then the reaction was cooled down to 0 °C and tigloyl chloride (0.19 g, 1.60 mmol) was added to the reaction. The mixture was stirred for one hour at this temperature. The solvents were removed under a nitrogen flow. The product was purified by HPLC (acetonitrile) and concentrated under a nitrogen flow. The obtained product was a colourless oil (0.14 g, 0.40 mmol, 50 %). ^{1}H-NMR ($CDCl_3$, 400 MHz) δ_H 0.92 (s, 3H, 13-CH_3); 0.92 (s, 3H, 12-CH_3); 1.82-1.87 (m, 6H, 1-CH_3, 4-CH_3); 2.41 (t, J = 5.9 Hz, 3H, 8-CH_2); 3.01-3.10 (m, 2H, 5-CH_2); 3.32-3.60 (m, 6H, 6-9-14-CH_2); 3.99 (s, ^{1}H, 11-CH); 6.33 (bt, J = 5.8 Hz, ^{1}H, 7-NH); 6.86 (qq, J = 1.3, 6.8 Hz, ^{1}H, 2-CH); 7.40 (bt, J = 5.6 Hz,^{1}H, 10-NH). ^{13}C-NMR ($CDCl_3$, 100 MHz): δ_C 12.2 (4-CH_3); 14.5 (1-CH_3), 20.4 (13-CH_3); 21.5 (12-CH_3); 28.2 (5-CH_2); 35.2 (9-CH_2); 35.6 (8-CH_2); 39.3 (C); 39.9 (6-CH_2); 70.9 (14-CH_2); 77.9 (11-CH); 136.8 (3-C); 137.3 (2-CH); 171.8 (CO); 173.5 (CO); 194.1 (COS). ESMS m/z (%): 383 [M]Na^{+}(8 %), 361 [M]H^{+}(100%), 343 [M - H_2O]H^{+}(2 %), 231 [M - $C_6H_{11}O_3$]H^{+}(5 %). HRMS m/z (%): calculated 383.1617, found 383.1621 for [M]Na+.

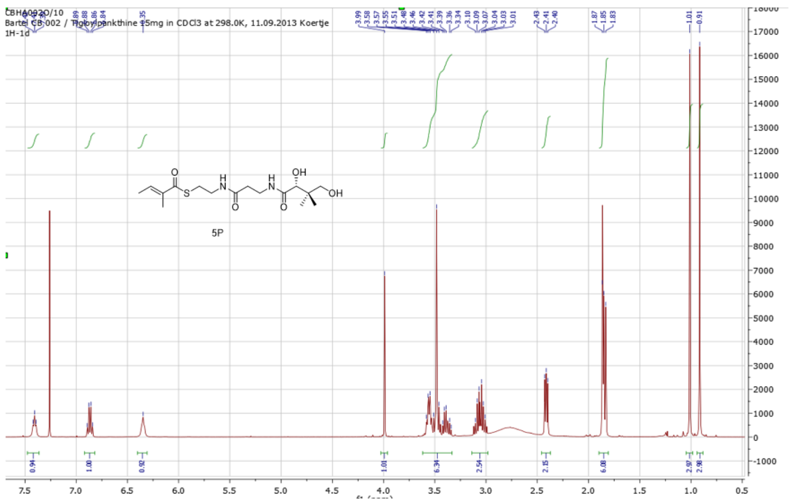

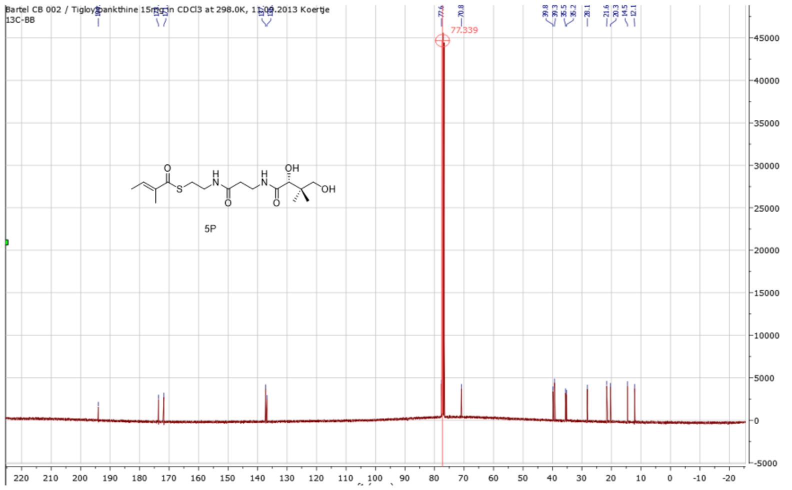

2*RS*-2-methylbutyryl pantetheine 6P

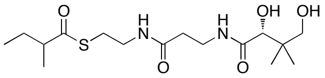

Chemical Formula: $C_{16}H_{30}N_2O_5S$
Exact Mass: 362.19

A solution of pantetheine (0.15 g, 27 µmol) and DTT (0.04 g, 27 µmol) in THF (1 ml) was stirred in a nitrogen atmosphäre at 30 °C for 3 h. After that pyridine (0.1 ml) and DMAP (4 mg, 0.03 mmol) were added to the reaction and stirred for 15 min. Then the reaction was cooled down to 0 °C and (±) 2-methylbutyryl chloride (0.06 g, 54 µmol) was added to the reaction. The mixture was stirred for one hour at this temperature. The solvents were removed under a nitrogen flow. The product was purified by HPLC (acetonitrile) and concentrated under a nitrogen flow. The obtained product was a colourless oil (0.035 g, 0.10 mmol, 36 %) ^{1}H-NMR ($CDCl_3$, 400 MHz) δ_H 0.90 (t, *J* = 7.6 Hz, 3H, 1-CH_3); 0.92 (s, 3H, 13-CH_3); 1.01 (s, 3H, 12-CH_3); 1.15 (d, *J* = 7.5 Hz, 3H, 4-CH_3); 1.36-1.78 (m, 2H, 2-CH_2); 2.40 (t, *J* = 6.1 Hz, 2H, 8-CH_2); 2.53-2.63 (m, ^{1}H, 3-CH); 2.96-3.05 (m, 2H, 5-CH_2); 3.37-3.60 (m, 6H, 6-9-14-CH_2); 3.99 (s,^{1}H, 15-CH_2); 6.32 (t, *J* = 5.4 Hz, ^{1}H, 7-NH); 7.44 (bt, *J* = 5.9 Hz, ^{1}H, 10-NH). ^{13}C-NMR ($CDCl_3$, 100 MHz) δ_C 17.1 (4-CH_3), 20.4 (13-CH_3); 21.5 (12-CH_3); 27.1 (2-CH_2); 27.4 (1-CH_2); 27.9 (5-CH_2); 35.1 (9-CH_2); 35.6 (8-CH_2); 39.3 (C); 39.7 (6-CH_2); 50.2 (14-CH_2); 70.8 (11-CH); 172.5 (CO); 174.5 (CO); 205.1 (COS). ESMS *m/z* (%): 385 [M]Na^+ (3 %), 363 [M]H^+ (100 %), 345 [M - H_2O]H^+ (3 %), 233 [M - $C_6H_{11}O_3$]H^+ (8 %), 100 [$C_5H_{11}O_2$] (35 %) HRMS *m/z* (%): calculated 361.1797, found 361.1796 for [M - H]-.

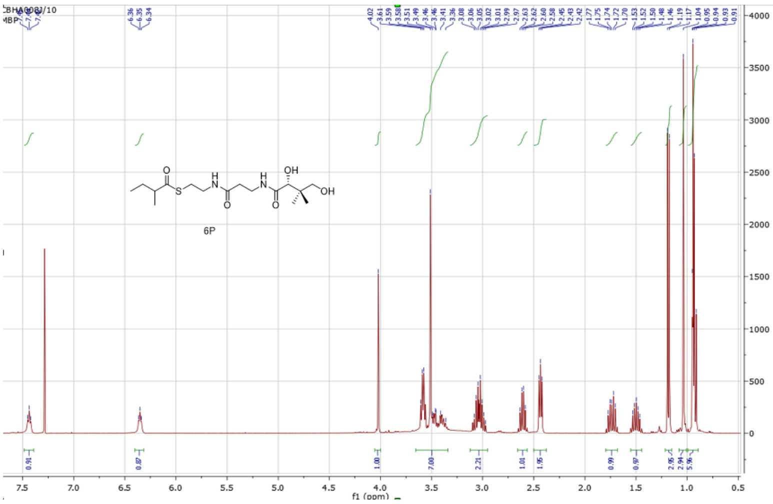

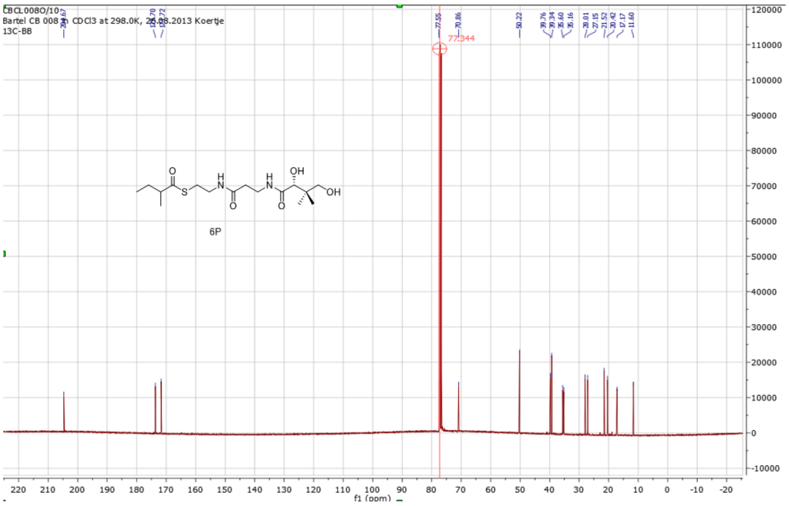

crotonyl-pantetheine 7P[26]

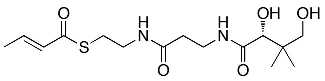

Chemical Formula: $C_{15}H_{26}N_2O_5S$
Exact Mass: 346.16

A solution of pantetheine (0.45 g, 0.80 mmol) and DTT (0.13 g, 0.80 mmol) in THF (4 ml) was stirred in a nitrogen atmosphäre at 30 °C for 3 h. After that triethylamine (0.3 ml) and DMAP (0.01 g, 0.09 mmol) were added to the reaction and stirred for 15 min. Then the reaction was cooled down to 0 °C and crotonyl chloride (0.16 g, 1.66 mmol) was added to the reaction. The mixture was stirred for one hour at this temperature. The solvents were removed under a nitrogen flow. The product was purified by HPLC (acetonitrile) and concentrated under a nitrogen flow. The obtained product was a colourless oil (0.18 g, 0.50 mmol, 34%) ^{1}H-NMR ($CDCl_3$, 400 MHz) δ_H 0.92 (s, 3H, 13-CH_3); 1.03 (s, 3H, 12-CH_3); 1.90 (dd, J = 1.3, 6.8 Hz, 3H, 1-CH_3); 2.41 (t, J = 5.9 Hz, 3H, 8-CH_2); 3.02-3.16 (m, 2H, 5-CH_2); 3.36-3.60 (m, 6H, 6-9-14-CH_2); 3.99 (s, ^{1}H, 11-CH); 6.13 (dd, J = 5.8 Hz, ^{1}H, 3-CH); 6.23 (bt, J = 5.8 Hz, ^{1}H, 7-NH); 6.95 (dq, J = 1.3, 6.8 Hz, ^{1}H, 2-CH); 7.36 (bt, J = 5.6 Hz,^{1}H, 10-NH). ^{13}C-NMR ($CDCl_3$, 100 MHz) δ_C 18.1 (1-CH_3), 20.4 (13-CH_3); 21.7 (12-CH_3); 28.1 (5-CH_2); 35.2 (9-CH_2); 35.6 (8-CH_2); 39.3 (C); 39.9 (6-CH_2); 70.9 (14-CH_2); 77.9 (11-CH); 129.7 (3-CH); 142.3 (2-CH); 171.8 (CO); 173.5 (CO); 190.5 (COS). ESMS *m/z* (%): 715.9 $[M_2]Na^+$(5 %), 693 $[M_2]H^+$(48 %), 369 $[M]Na^+$(2 %), 347 $[M]H^+$(100 %), 329 $[M - H_2O]H^+$(2 %). HRMS *m/z* (%): calculated 369.1460, found 369.1461 for [M]Na+.

angeloyl-pantetheine 8P

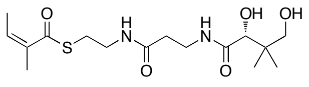

Chemical Formula: $C_{16}H_{28}N_2O_5S$
Exact Mass: 360.17

Angeloyl pantetheine dimethyl ketal (**8Pa**, 0.02 g, 0.002 mmol) stirred in a mixture of acetonitrile and water (1:1) and 10 % TFA for 20 min. The reaction was followed by TLC and LCMS. After that the solvents were liphophilized. 0.03 g of the product were purified by HPLC. ^{1}H-NMR ($CDCl_3$, 400 MHz) δ_H 0.92 (s, 3H, 13-CH_3); 1.01 (s, 3H, 12-CH_3); 1.83-1.90 (m, 6H, 1-CH_3, 4-CH_3); 2.40-2.44 (m, 2H, 8-CH_2); 2.99-3.14 (m, 2H, 5-CH_2); 3.34-3.59 (m, 6H, 6-9-14-CH_2); 3.99 (s, ^{1}H, 11-CH); 5.90 (dq, J = 1.2, 7.3 Hz, ^{1}H, 2-CH); 6.35 (bt, J = 5.5 Hz, ^{1}H, 7-NH); 7.40 (bt, J = 6.1 Hz,^{1}H, 10-NH). ^{13}C-NMR ($CDCl_3$, 100 MHz) δ_C 15.9 (1-CH_3), 20.4 (13-CH_3); 20.6 (4-CH_3); 21.5 (12-CH_3); 28.2 (5-CH_2); 35.2 (9-CH_2); 35.6 (8-CH_2); 39.4 (C); 39.8 (6-CH_2); 70.9 (14-CH_2); 77.9 (11-CH); 134.0 (3-C); 135.3 (2-CH); 171.8 (CO); 173.5 (CO); 194.1 (COS). ESMS m/z (%): 383 $[M]Na^{+}$ (8 %), 361 $[M]H^{+}$ (58 %), 343 $[M - H_2O]H^{+}$ (88 %), 231 $[M - C_6H_{11}O_3]H^{+}$ (100 %). HRMS m/z (%): calculated 383.1617, found 383.1616 for [M]Na+.

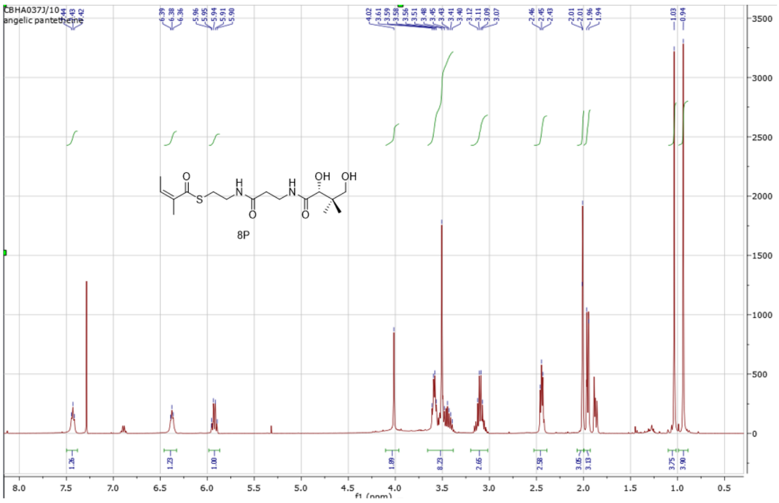

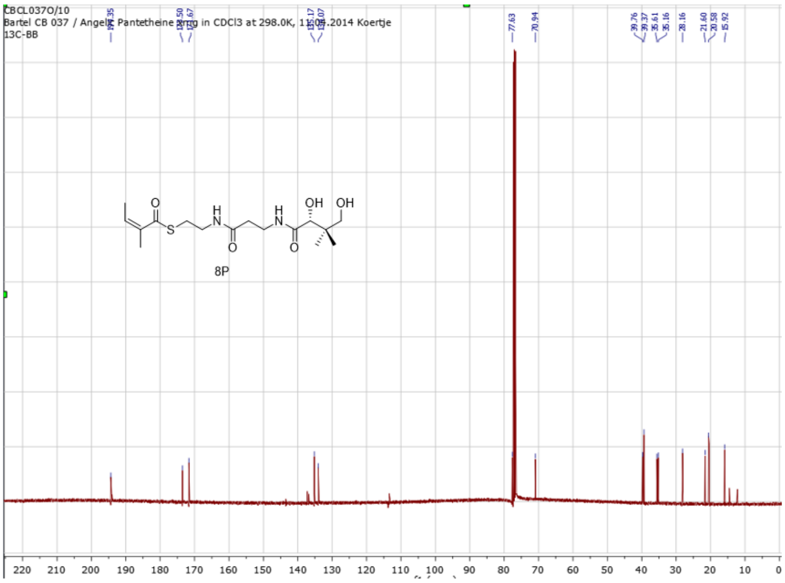

E-2,3-Dimethylbut-2-enoyl pantetheine 9P

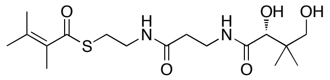

Chemical Formula: $C_{17}H_{30}N_2O_5S$
Exact Mass: 374.19

The acyl pantetheine dimethyl acetal (**9Pa**, 0.55 g ,1.32 mmol) was dissolved in CH_2Cl_2 (2 ml). TFA (0.2 ml) was then added and the solution was stirred for 15 min at 22 °C. Thereafter, H_2O was added and the solution was stirred for an additional 10 min at 22 °C. The solvent was then removed by blowing of N_2 flow and the product was purified by HPLC. After purification, 202.0 mg (0.54 mmol) of the colorless oily product 3c were collected. ^{1}H NMR (400 MHz, $CDCl_3$): δ_H = 0.91 (3 H, s, 10/11-CH_3), 1.01 (3 H, s, 10/11-CH_3), 1.79 (3 H, s, 19-CH_3), 1.92 (6 H, s, 18-CH_3 and 20-CH_3), 2.38- 2.45 (2 H, m, 6-CH_2), 2.93-3.14 (2 H, m, 9-CH_2), 3.29-3.61 (7 H, overlapping peaks, OH, 1-CH_2, 5-CH_2 and 8-CH_2), 3.98 (1 H, s, 3-CH), 6.32 (1 H, br. t, NH), 7.40 (1 H, br. t, NH) ppm. ^{13}C NMR (100 MHz, $CDCl_3$): δ_C = 16.2 (18-CH_3 and 20-CH_3), 20.5 (10/11-CH_3), 21.8 (10/11-CH_3), 23.0 (19-CH_3), 28.6 (9-CH_2), 35.3 (5-CH_2), 35.7 (6-CH_2), 39.5 (2-C), 40.1 (8-CH_2), 71.1 (1- CH_2), 77.8 (3-CH), 129.3 (17-C), 141.0 (16-C), 171.8 (7-CO), 173.6 (4-CO), 196.7 (15-CO) ppm. IR: ν_{max} = 3291 (w), 2933 (w), 2873 (w), 1642 (vs), 1526 (s), 1438 (w), 1374 (m), 1292 (w), 1262 (w), 1198 (w), 1167 (w), 1136 (w), 1077 (w), 1041 (m), 950 (m), 784 (w), 689 (w), 643 (w) cm^{-1}. ESMS: *m/z* (%) = 375 (82) [M]H+ , 357 (76) [M + H - H_2O]+ , 261 (43) [M + H - C_6H_9O - H_2O]+ , 245 (100) [M + H - $C_6H_{11}O_3$]+ .

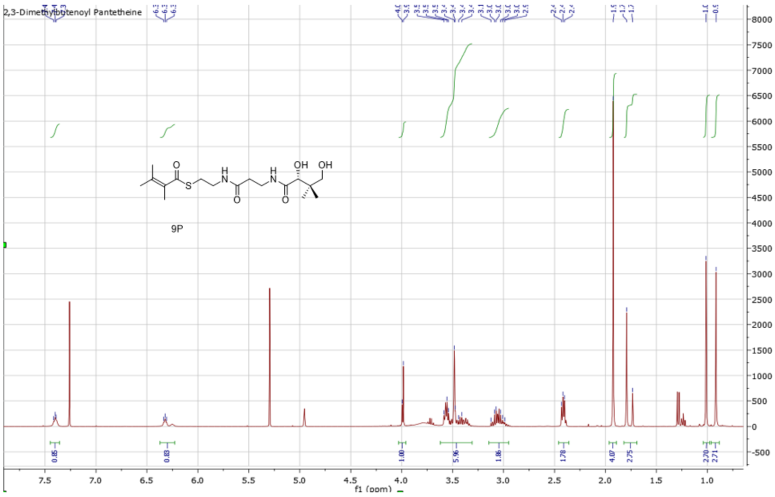

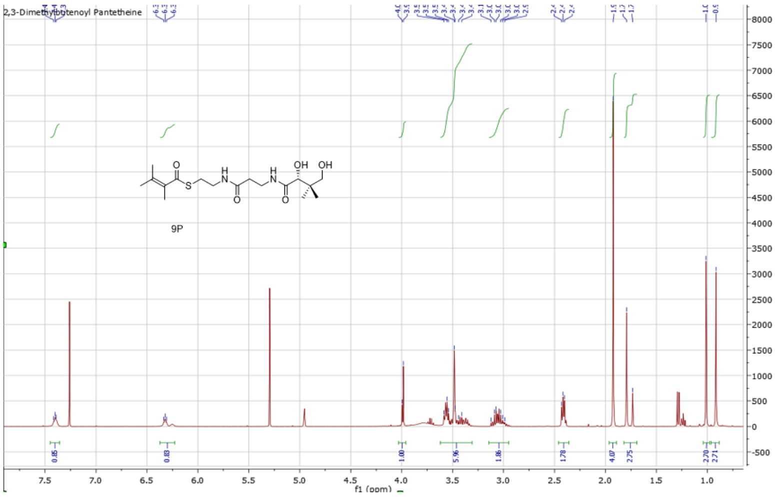

E-2-Ethylbut-2-enoyl pantetheine 10P

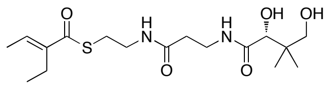

Chemical Formula: $C_{17}H_{30}N_2O_5S$
Exact Mass: 374.19

The acyl pantetheine dimethyl acetal (**10Pa,** 0.99 g, 2.38 mmol) of was dissolved in CH_2Cl_2 (2 ml). TFA (0.2 ml) was then added and the solution was stirred for 15 min at 22 °C. Thereafter, H_2O was added and the solution was stirred for an additional 10 min at 22 °C. The solvent was then removed by blowing of N_2 flow and the product was purified by HPLC. After purification, 261.0 mg (0.70 mmol) of the colorless oily product were collected. ^{1}H NMR (400 MHz, $CDCl_3$): δ_H = 0.91 (3 H, s, 10/11-CH_3), 0.99 (3 H, t, *J*HH = 7.4 Hz, 20-CH_3), 1.01 (3 H, s, 10/11-CH_3), 1.85 (3 H, d, *J*HH = 7.0 Hz, 18-CH_3), 2.35 (2H, q, *J*HH = 7.5 Hz, 19-CH_2), 2.40 (2 H, t, *J*HH = 5.8 Hz, 6-CH_2), 2.98-3.16 (2 H, m, 9-CH_2), 3.33-3.61 (7 H, overlapping peaks, OH, 1-CH_2, 5-CH_2 and 8-CH_2), 3.99 (1 H, s, 3-CH), 6.33 (1 H, br. t, NH), 6.82 (1 H, q, *J*HH = 7.1 Hz, 17-CH), 7.40 (1 H, br. t, NH) ppm. ^{13}C NMR (100 MHz, $CDCl_3$): δ_C = 13.5 (20-CH_3), 14.3 (18-CH_3), 20.2 (19-CH_3), 20.5 (10/11-CH_3), 21.8 (10/11-CH_3), 28.3 (9-CH_2), 35.3 (5-CH_2), 35.7 (6-CH_2), 39.5 (2-C), 40.0 (8-CH_2), 71.1 (1-CH_2), 77.8 (3-CH), 137.0 (17-CH), 143.2 (16-C), 171.8 (7-CO), 173.6 (4-CO), 193.9 (15-CO) ppm. IR: νM_{ax} = 3294 (w), 2965 (w), 2934 (w), 2874 (w), 1641 (vs), 1526 (s), 1438 (w), 1380 (m), 1292 (w), 1206 (w), 1073 (w), 1042 (m), 1011 (w), 958 (w), 844 (w), 735 (w), 690 (w), 652 (w) cm^{-1}. ESMS: *m/z* (%) = 397 (51) [M]Na+ , 375 (67) [M]H+ , 357 (88) [M + H - H_2O]+ , 261 (73) [M + H - C_6H_9O - H_2O]+ , 245 (100) [M + H - $C_6H_{11}O_3$]+ .

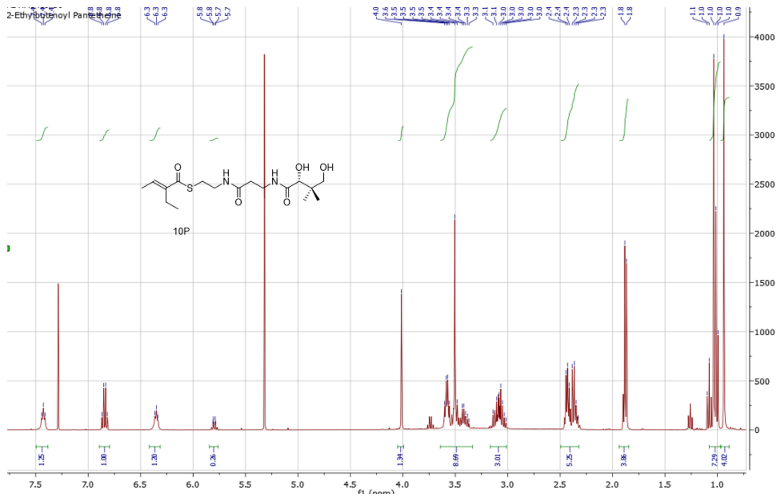

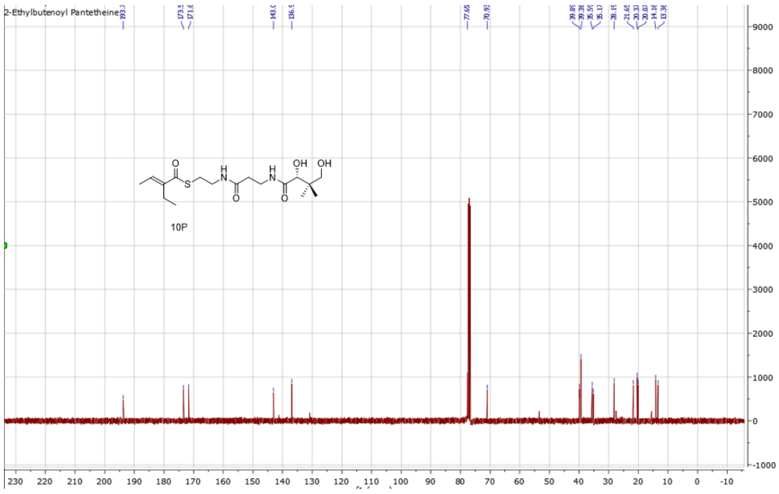

E-hex-2-enoyl pantetheine 11P[28]

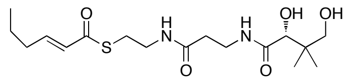

Chemical Formula: $C_{17}H_{30}N_2O_5S$
Exact Mass: 374.19

E-Hex-2-enoyl pantetheine dimethyl ketal (**11Pa**, 0.02 g, 0.002 mmol) stirred in a mixture of acetonitrile and water (1:1) and 10 % TFA for 20 min. The reaction was followed by TLC and LCMS. After that the solvents were liphophilized. 0.003 g of the product were purified by HPLC. ^{1}H-NMR ($CDCl_3$, 400 MHz) δ_H 0.93 (s, 3H, 21-CH_3); 0.95 (t, *J* = 7.8 Hz, 3H, 1-CH_3); 1.04 (s, 3H, 20-CH_3); 1.46-1.56 (m, 2H, 2-CH_2); 2.20 (dq, *J* = 1.3 Hz, *J* = 6.7 Hz, 2H, 5-CH_2); 2.41 (t, *J* = 6.1 Hz, 2H, 11-CH_2); 3.03-3.18 (m, 2H, 7-CH_2); 3.36-3.65 (m, 6H, 8-12-17-CH_2); 3.99 (s,^{1}H, 15-CH); 6.13 (m, ^{1}H, 5-CH); 6.17 (dt, *J* = 1.8 Hz, *J* = 15.7 Hz, ^{1}H, 9-NH); 6.93 (dt, *J* = 6,7 Hz, *J* = 15.3 Hz, ^{1}H, 4-CH); 7.32 (bt, *J* = 6.^{1}Hz, ^{1}H, 13-NH). ^{13}C-NMR ($CDCl_3$, 100 MHz) δ_C 13.7 (1-CH_3), 20.4 (19-CH_3); 21.1 (18-CH_3); 21.7 (2-CH_2); 28.1 (7-CH_2); 34.3 (3-CH_2); 35.1 (12-CH_2); 35.5 (11-CH_2); 39.4 (16-C); 39.8 (8-CH_2); 70.9 (17-CH_2); 77.8 (15-CH); 128.3 (5-CH); 147.0 (4-CH); 171.6 (10-CO); 173.3 (14-CO); 190.6 (6-COS). ESMS *m/z* (%): 375 $[M]H^+$ (100 %), 357 $[M - H_2O]H^+$ (32 %). HRMS *m/z* (%): calculated 397.1773, found 397.1774 for [M]Na+.

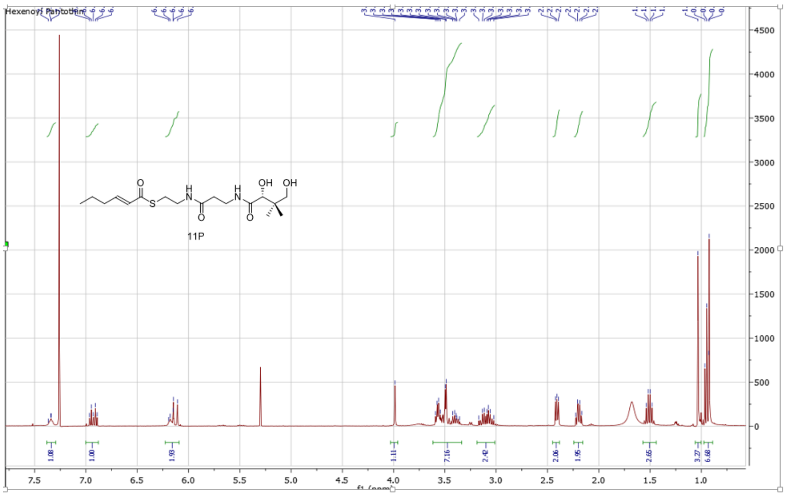

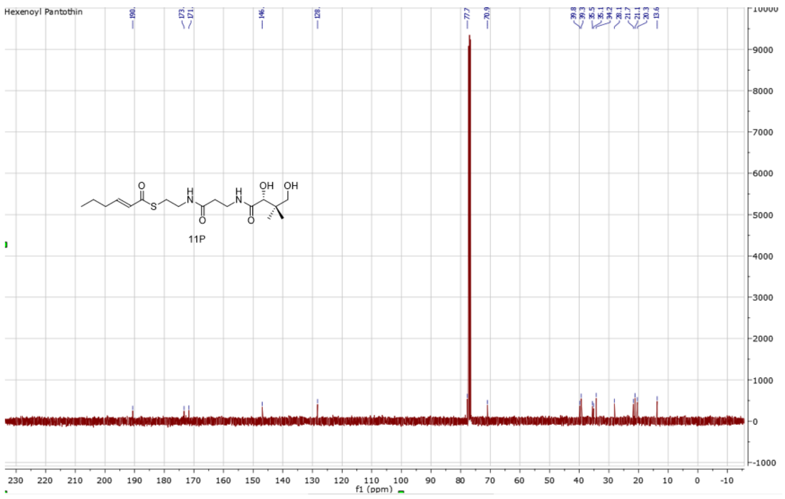

E-2-methylhex-2-enoyl-pantetheine 12P

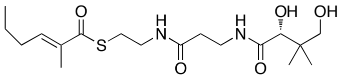

Chemical Formula: $C_{18}H_{32}N_2O_5S$
Exact Mass: 388.20

E-Methylhex-2-enoyl pantetheine dimethyl ketal (**12Pa**, 0.06 g, 0.003 mmol) stirred in a mixture of CH_2Cl_2 and 10 % TFA for 20 min. The reaction was followed by TLC and LCMS. After that the solvents were evaporated. 0.02 g of the product were purified by HPLC. ^{1}H-NMR ($CDCl_3$, 400 MHz) δ_H 0.93 (s, 3H, 18-CH_3); 0.95 (t, J = 7.4 Hz, 3H, 1-CH_3); 1.02 (s, 3H, 19-CH_3); 1.46-1.55 (m, 2H, 2-CH_2); 1.88 (d, J = 1.4 Hz, 5-CH_3) 2.20 (dq, J = 1.3 Hz, J = 7.0 Hz, 2H, 3-CH_2); 2.41 (t, J = 5.9 Hz, 2H, 11-CH_2); 3.00-3.13 (m, 2H, 8-CH_2); 3.35-3.59 (m, 6H, 9-12-17-CH_2); 3.99 (s,^{1}H, 15-CH); 6.23 (bt, J = 5.7 Hz, ^{1}H, 10-NH); 6.77 (tq, J = 1,4 Hz, J = 7.4 Hz, ^{1}H, 4-CH); 7.38 (bt, J = 5.9 Hz, ^{1}H, 13-NH). ^{13}C-NMR ($CDCl_3$, 100 MHz) δ_C 12.5 (5-CH_3), 13.9 (1-CH_3), 20.4 (19-CH_3); 21.7 (18-CH_3); 21.8 (2-CH_2); 28.3 (8-CH_2); 30.8 (3-CH_2); 35.1 (12-CH_2); 35.5 (11-CH_2); 39.4 (16-C); 39.8 (9-CH_2); 70.9 (17-CH_2); 77.7 (15-CH); 135.7 (5-C); 142.5 (4-CH); 171.6 (11-CO); 173.4 (14-CO); 194.2 (7-COS). ESMS *m/z* (%): 411 [M]Na^+ (28 %), 389 [M]H^+ (31 %), 371 [M - H_2O]H^+ (88 %), 259 [M - $C_6H_{11}O_3$]H^+ (100 %). HRMS *m/z* (%): calculated 411.1930, found 411.1931 for [M]Na+.

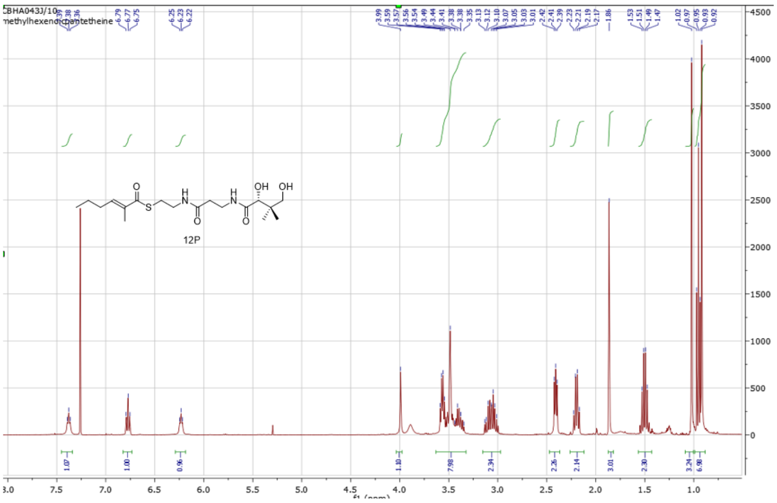

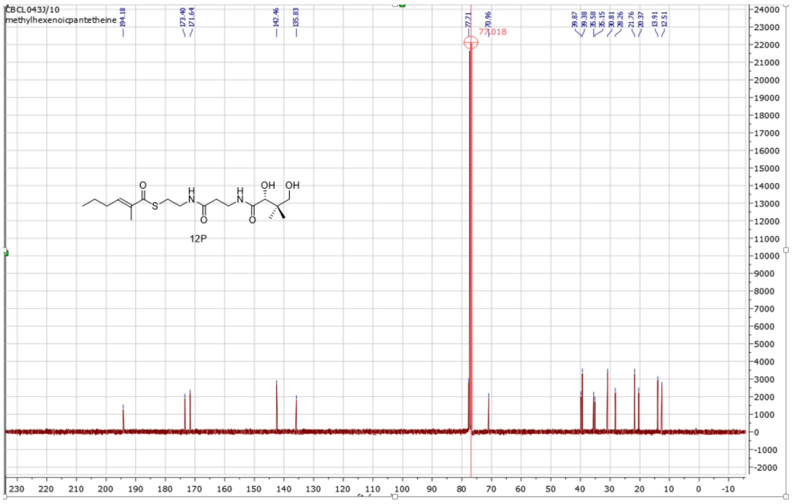

_E_-4_RS_-4-methyl-hex-2-enoyl-pantetheine 13P

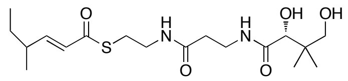

Chemical Formula: $C_{18}H_{32}N_2O_5S$
Exact Mass: 388.20

_E_-4_RS_-4-Methylhex-2-enoyl pantetheine dimethyl ketal (**13Pa**, 0.06 g, 0.003 mmol) stirred in a mixture of CH_2Cl_2 and 10 % TFA for 20 min. The reaction was followed by TLC and LCMS. After that the solvents were evaporated. 0.02 g of the product were purified by HPLC. ^{1}H-NMR ($CDCl_3$, 400 MHz) δ_H 0.88 (t, J = 7.5 Hz, 3H, 1-CH_3); 0.98 (s, 3H, 18-CH_3); 1.01 (s, 3H, 19-CH_3); 1.05 (d, J = 6.9 Hz, 4-CH_3); 1.38-1.47 (m, 2H, 2-CH_2); 2.19-2.27 (m, ^{1}H, 3-CH); 2.41 (t, J = 5.8 Hz, 2H, 11-CH_2); 2.94-3.15 (m, 2H, 8-CH_2); 3.33-3.58 (m, 6H, 9-12-17-CH_2); 4.00 (s,^{1}H, 15-CH); 6.08 (dd, J = 1.4 Hz, J = 15.4 Hz, ^{1}H, 6-CH); 6.40 (bt, J = 5.4 Hz, ^{1}H, 10-NH); 6.83 (dd, J = 7.4 Hz, J = 15.4 Hz, ^{1}H, 5-CH); 7.40 (bt, J = 5.4 Hz, ^{1}H, 13-NH). ^{13}C-NMR ($CDCl_3$, 100 MHz) δ_C 11.6 (1-CH_3), 18.7 (4-CH_3), 20.4 (19-CH_3); 21.7 (18-CH_3); 28.2 (2-CH_2); 28.8 (8-CH_2); 30.7 (3-CH_2); 35.1 (12-CH_2); 35.2 (11-CH_2); 39.4 (16-C); 39.7 (9-CH_2); 70.8 (17-CH_2); 77.6 (15-CH); 126.7 (6-CH); 152.1 (5-CH); 171.7 (10-CO); 173.6 (14-CO); 190.8 (7-COS). ESMS m/z (%): 411 $[M]Na^+$ (8 %), 389 $[M]H^+$ (65 %), 371 $[M - H_2O]H^+$ (88 %), 259 $[M - C_6H_{11}O_3]H^+$ (100 %). HRMS m/z (%): calculated 411.1930, found 411.1930 for [M]Na+.

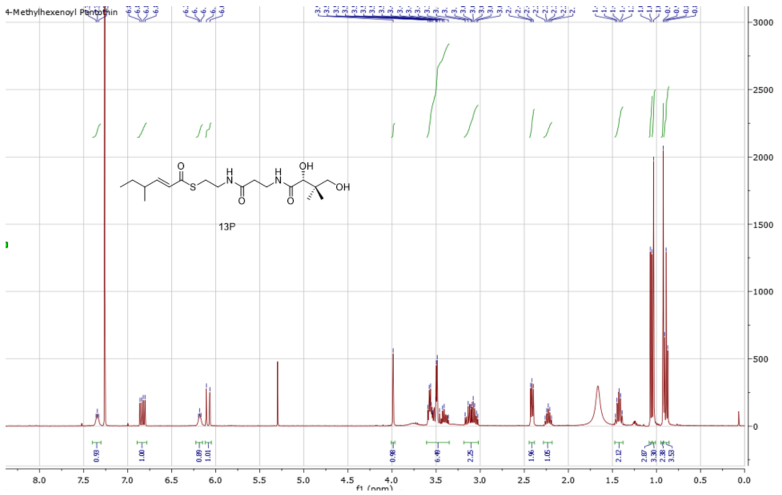

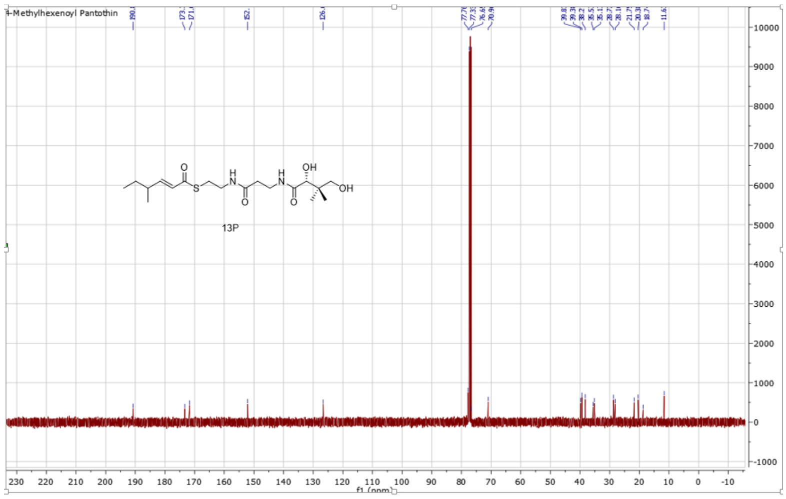

E-4*RS*-2,4-dimethylhex-2-enoyl-pantetheine 14P

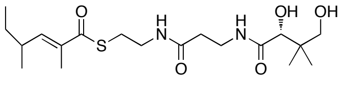

Chemical Formula: $C_{19}H_{34}N_2O_5S$
Exact Mass: 402.22

E-4*RS*-2,4-Dimethylhex-2-enoyl pantetheine dimethyl ketal (**14Pa**, 0.06 g, 0.003 mmol) was stirred in a mixture of CH_2Cl_2 and 10 % TFA for 20 min. The reaction was followed by TLC and LCMS. After that the solvents were evaporated. 0.01 g of the product were purified by HPLC (acetonitrile). ^{1}H-NMR ($CDCl_3$, 400 MHz) δ_H 0.86 (t, J = 7.4 Hz, 3H, 1-CH_3); 0.92 (s, 3H, 18-CH_3); 1.03 (s, 3H, 19-CH_3); 1.03 (d, J = 2.3 Hz, 3H, 4-CH_3); 1.30-1.51 (m, 4H, 2-CH_2); 1.88 (d, J = 1.1 Hz, 7-CH_3), 2.41 (t, J = 5.6 Hz, 2H, 12-CH_2); 2.40-2.49 (m, ^{1}H, 3-CH); 2.99-3.14 (m, 2H, 9-CH_2); 3.35-3.59 (m, 6H, 10-13-17-CH_2); 3.99 (s,^{1}H, 15-CH); 6.19 (bt, J = 5.7 Hz, ^{1}H, 11-NH); 6.54 (dq, J = 1,3 Hz, J = 9.8 Hz, ^{1}H, 5-CH); 7.36 (bt, J = 5.9 Hz, ^{1}H, 14-NH). ^{13}C-NMR ($CDCl_3$, 100 MHz) δ_C 11.9 (1-CH_3), 12.7 (4-CH_3), 19.5 (7-CH_3), 20.4 (19-CH_3); 21.7 (18-CH_3); 28.3 (9-CH_2); 29.6 (3-CH); 35.1 (13-CH_2); 35.6 (12-CH_2); 39.4 (16-C); 39.8 (10-CH_2); 71.0 (17-CH_2); 77.7 (15-CH); 134.4 (6-C); 147.8 (5-CH); 171.4 (11-CO); 173.3 (14-CO); 194.5 (8-COS). ESMS *m/z* (%): 425 $[M]Na^+$ (12%), 403 $[M]H^+$ (28%), 385 $[M - H_2O]H^+$ (88%), 273 $[M - C_6H_{11}O_3]H^+$ (100%). HRMS *m/z* (%): calculated 425.2086, found 425.2085 for [M]H+.

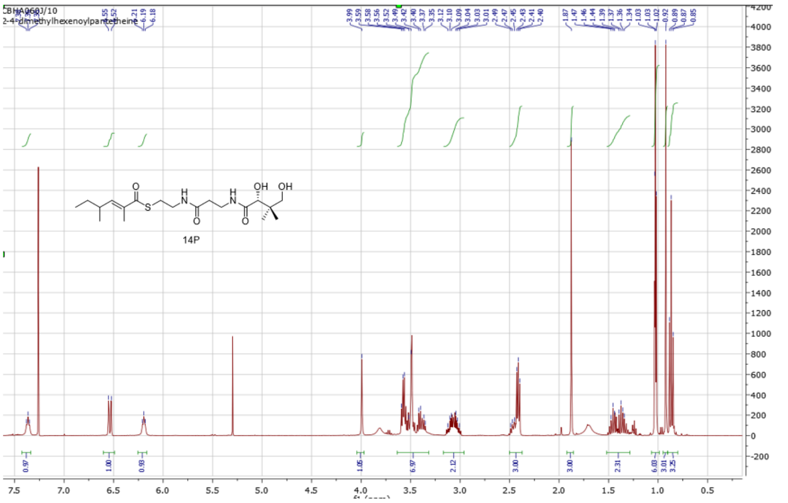

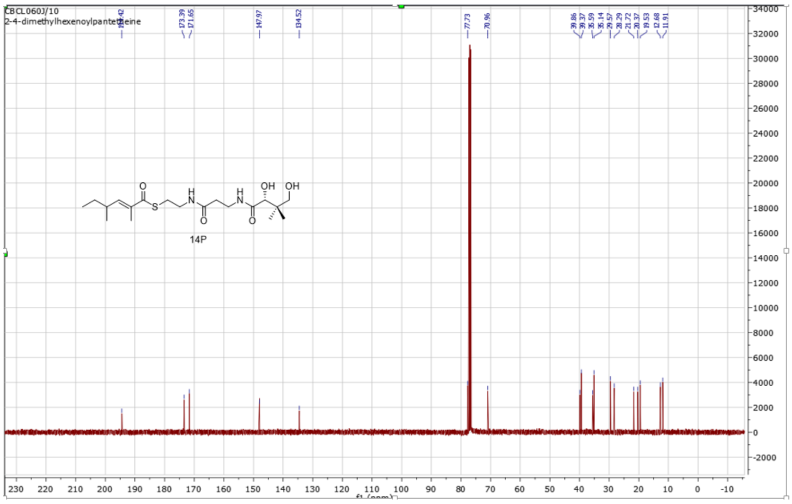

_E_-4_S_-2,4-dimethylhex-2-enoyl-pantetheine 15P

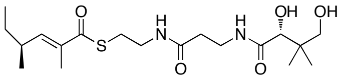

Chemical Formula: $C_{19}H_{34}N_2O_5S$
Exact Mass: 402.22

E-4S-2,4-Dimethylhex-2-enoyl pantetheine dimethyl ketal (**15Pa**, 0.06 g, 0.003 mmol) was stirred in a mixture of CH_2Cl_2 and 10 % TFA for 20 min. The reaction was followed by TLC and LCMS. After that the solvents were evaporated. 0.01 g of the product were purified by HPLC. ^{1}H-NMR ($CDCl_3$, 400 MHz) δ_H 0.86 (t, J = 7.4 Hz, 3H, 1-CH_3); 0.92 (s, 3H, 18-CH_3); 1.03 (s, 3H, 19-CH_3); 1.03 (d, J = 2.3 Hz, 3H, 4-CH_3); 1.30-1.51 (m, 4H, 2-CH_2); 1.88 (d, J = 1.1 Hz, 7-CH_3), 2.41 (t, J = 5.6 Hz, 2H, 12-CH_2); 2.40-2.49 (m, ^{1}H, 3-CH); 2.99-3.14 (m, 2H, 9-CH_2); 3.35-3.59 (m, 6H, 10-13-17-CH_2); 3.99 (s,^{1}H, 15-CH); 6.19 (bt, J = 5.7 Hz, ^{1}H, 11-NH); 6.54 (dq, J = 1,3 Hz, J = 9.8 Hz, ^{1}H, 5-CH); 7.36 (bt, J = 5.9 Hz, ^{1}H, 14-NH). ^{13}C-NMR ($CDCl_3$, 100 MHz) δ_C 11.9 (1-CH_3), 12.7 (4-CH_3), 19.5 (7-CH_3), 20.4 (19-CH_3); 21.7 (18-CH_3); 28.3 (9-CH_2); 29.6 (3-CH); 35.1 (13-CH_2); 35.6 (12-CH_2); 39.4 (16-C); 39.8 (10-CH_2); 71.0 (17-CH_2); 77.7 (15-CH); 134.4 (6-C); 147.8 (5-CH); 171.4 (11-CO); 173.3 (14-CO); 194.5 (8-COS). ESMS *m*/*z* (%): 425 $[M]Na^+$ (11%), 403 $[M]H^+$(26%), 385 $[M - H_2O]H^+$(88%), 273 $[M - C_6H_{11}O_3]H^+$(100%). HRMS *m*/*z* (%): calculated 425.2086, found 425.2085 for [M]Na+.

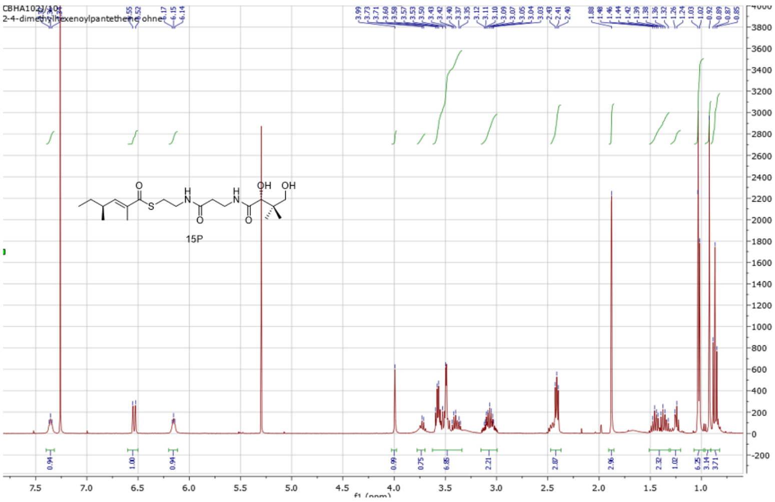

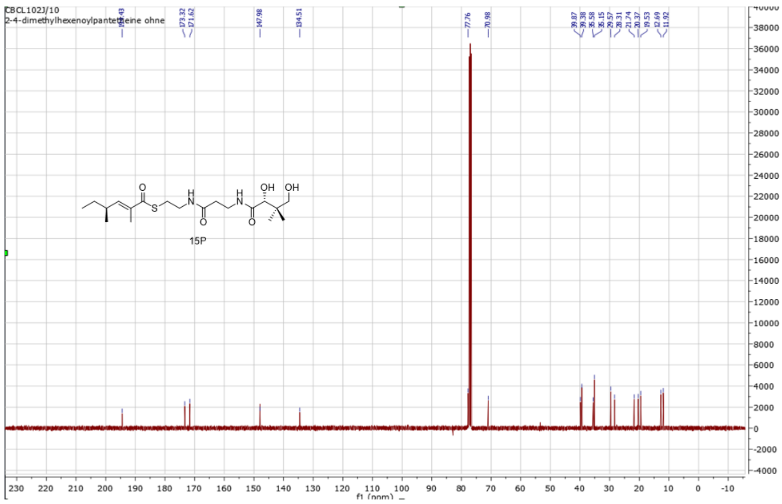

_E_-2-ethylhex-2-enoyl-pantetheine 16P

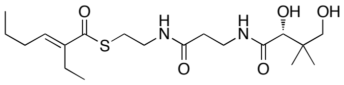

Chemical Formula: $C_{19}H_{34}N_2O_5S$
Exact Mass: 402.22

E-2-Ethylhex-2-enoyl pantetheine dimethyl ketal (**16Pa**, 300 mg) was stirred in a mixture of CH_2Cl_2 and 10 % TFA for 20 min. The reaction was followed by TLC and LCMS. After that the solvents were evaporated. 0.027 g of the product were purified by HPLC. ^{1}H-NMR ($CDCl_3$, 400 MHz) δ_H 0.85-0.99 (m, 12H, 1-7-18-19-CH_3); 1.38-1.53 (m, 2H, 2-CH_2); 2.16-2.35 (m, 4H, 3-6-CH_2); 2.41 (t, *J* = 6.4 Hz, 2H, 12-CH_2); 2.97-3.06 (m, 2H, 9-CH_2); 3.33-3.56 (m, 6H, 10-13-17-CH_2); 3.98 (s,^{1}H, 15-CH); 5.63 (t, *J* = 7.6 Hz, ^{1}H, 4-CH_Z); 6.56 (bt, *J* = 5.7 Hz, ^{1}H, 11-NH); 6.70 (t, *J* = 7.6 Hz, ^{1}H, 4-CH_E); 7.46 (bt, *J* = 5.9 Hz, ^{1}H, 14-NH). ^{13}C-NMR ($CDCl_3$, 100 MHz) δ_C 13.4 (1-CH_3), 13.9 (7-CH_3), 20.4 (19-CH_3); 21.5 (18-CH_3); 22.0 (6-CH_2); 22.8 (2-CH_2); 28.2 (9-CH_2); 30.4 (3-CH_2); 35.1 (13-CH_2); 35.5 (12-CH_2); 39.4 (16-C); 39.8 (10-CH_2); 70.9 (17-CH_2); 77.7 (15-CH); 136.1 (4-CH_Z); 140.5 (5-C); 142.2 (4-CH_E); 171.6 (11-CO); 173.4 (14-CO); 193.9 (8-COS). ESMS *m/z* (%): 425 [M]Na^+ (78 %), 403 [M]H^+(13 %), 385 [M - H_2O]H^+(56 %), 273 [M - $C_6H_{11}O_3$]H^+(100 %). HRMS *m/z* (%): calculated 425.2086, found 425.2086 for [M]Na+.

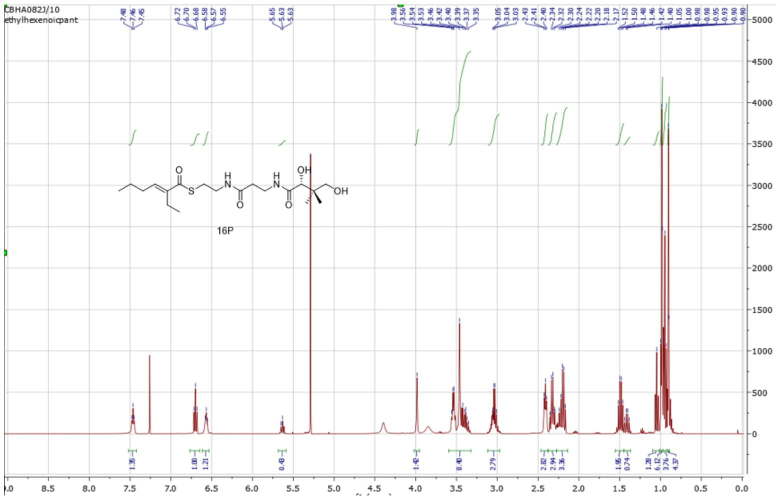

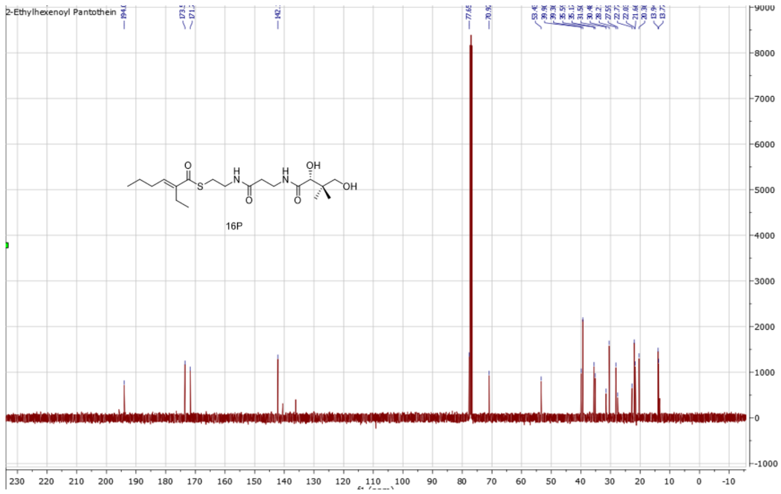

***E*-hept-2-enoyl-pantetheine 17P**

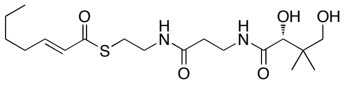

Chemical Formula: $C_{18}H_{32}N_2O_5S$
Exact Mass: 388.20

E-Hept-2-enoyl chloride was prepared from *E*-hept-2-enoic acid (**17,** 0.11 g, 0.90 mmol) with thionyl chloride (0.11 g, 1.00 mmol) in THF (0.5 ml). This solution was stirred for 24 h under nitrogen, followed by evaporation of solvent.

A solution of pantetheine (0.30 g, 0.54 mmol) and DTT (88 mg, 0.54 mmol) in THF (3 ml) was stirred in a nitrogen atmosphäre at 30 °C for 3 h. Pyridine (0.3 ml) and DMAP (12 mg, 0.09 mmol) were added to the reaction and stirred for 15 min. The reaction was cooled to 0 °C and hept-2-enoyl chloride was added. The mixture was stirred for one hour at this temperature. The solvents were removed under a nitrogen flow. 6 mg of the product were purified by HPLC. ^{1}H-NMR ($CDCl_3$, 400 MHz) δ_H 0.94 (t, J = 7.1 Hz, 3H, 1-CH_3); 0.95 (s, 3H, 18-CH_3); 1.06 (s, 3H, 17-CH_3); 1.28-1.42 (m, 4-H, 2-3-CH_2); 2.24 (dq, J = 1.6 Hz, J = 7.2 Hz, 2H, 4-CH_2); 2.43 (t, J = 7.2 Hz, 2H, 11-CH_2); 3,05-3.25 (m, 2H, 8-CH_2); 3.38-3.62 (m, 6H, 16-12-9-CH_2); 4.01 (s,^{1}H, 14-CH); 6.13 (m, ^{1}H, 6-CH); 6.15 (dt, J = 1.5 Hz, J = 15.4 Hz, ^{1}H, 10-NH); 6.96 (dt, J = 7,0 Hz, J = 15.5 Hz, ^{1}H, 5-CH); 7.34 (bt, J = 6.0 Hz, ^{1}H, 13-NH). ^{13}C-NMR ($CDCl_3$, 100 MHz) δ_C 13.8 (1-CH_3), 20.4 (17-CH_3); 21.7 (18-CH_3); 22.3 (2-CH_2); 28.1 (8-CH_2); 29.9 (3-CH_2); 31.9 (4-CH_2); 35.1 (12-CH_2); 35.6 (11-CH_2); 39.3 (15-C); 39.9 (9-CH_2); 70.9 (16-CH_2); 77.8 (14-CH); 128.1 (6-CH); 147.3 (5-CH); 171.6 (10-CO); 173.2 (13-CO); 190.6 (7-COS). ESMS *m*/*z* (%):777.9 $[M_2]H^+$ (28 %), 389 $[M]H^+$ (100 %). HRMS *m*/*z* (%): calculated 411.1930, found 411.1929 for [M]Na+.

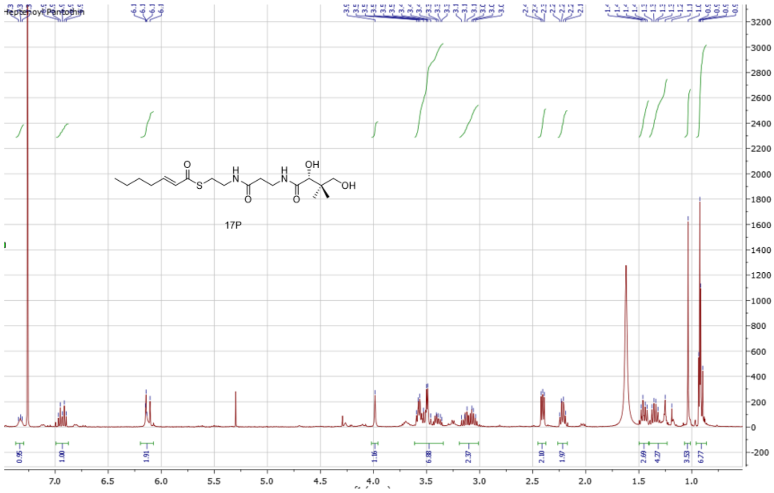

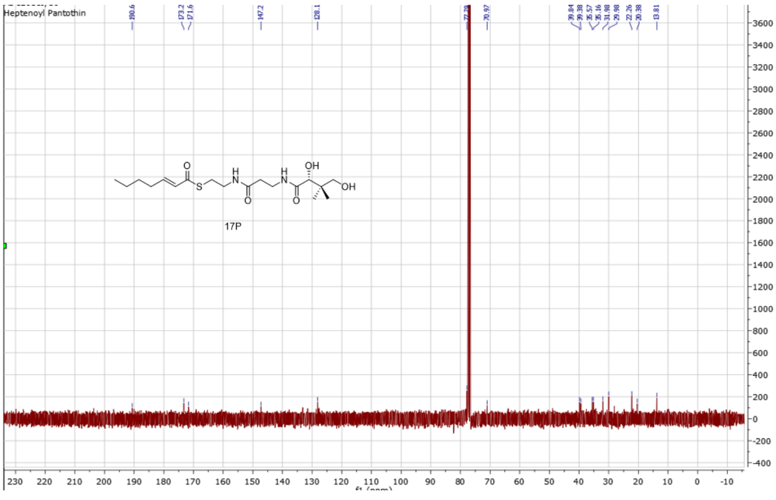

_E_-6-methylhept-2-enoyl pantetheine 18P

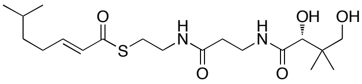

Chemical Formula: $C_{19}H_{34}N_2O_5S$
Exact Mass: 402.22

E-6-Methylhept-2-enoyl pantetheine dimethyl ketal (**18Pa**, 0.06 g, 0.003 mmol) was stirred in a mixture of CH_2Cl_2 and 10 % TFA for 20 min. The reaction was followed by TLC and LCMS. After that the solvents were evaporated. 0.01 g of the product were purified by HPLC. ^{1}H-NMR ($CDCl_3$, 400 MHz) δ_H 0.86-0.92 (m, 9H, 1-3-18-CH_3); 1.01 (s, 3H, 19-CH_3); 1.14-1.38 (m, 2H, 4-CH_2); 1.47-1.62 (m, ^{1}H, 2-CH); 2.22 (q, J = 7.0 Hz, 2H, 5-CH_2); 2.41 (t, J = 6.2 Hz, 2H, 12-CH_2); 3.01-3.15 (m, 2H, 9-CH_2); 3.35-3.58 (m, 6H, 10-13-17-CH_2); 3.99 (s,^{1}H, 15-CH); 6.13 (dt, J = 1.5 Hz, J = 15.2 Hz, ^{1}H, 7-CH); 6.39 (bt, J = 5.6 Hz, ^{1}H, 11-NH); 6.93 (dt, J = 6.5 Hz, J = 15.5 Hz, ^{1}H, 6-CH); 7.39 (bt, J = 5.9 Hz, ^{1}H, 14-NH). ^{13}C-NMR ($CDCl_3$, 100 MHz) δ_C 20.4 (19-CH_3); 21.7 (18-CH_3); 22.4 (1-3-CH_3); 27.6 (2-CH); 28.1 (9-CH_2); 30.2 (4-CH_2); 35.1 (13-CH_2); 35.6 (12-CH_2); 36.9 (5-CH_2); 39.4 (16-C); 39.8 (10-CH_2); 70.9 (17-CH_2); 77.7 (15-CH); 128.0 (7-CH); 147.4 (6-CH); 171.7 (11-CO); 173.5 (14-CO); 190.6 (8-COS). ESMS m/z (%): 425 $[M]Na^+$ (12%), 403 $[M]H^+$ (30%), 385 $[M - H_2O]H^+$ (88%), 273 $[M - C_6H_{11}O_3]H^+$ (100%). HRMS m/z (%): calculated 425.2086, found 425.2089 for [M]Na+.

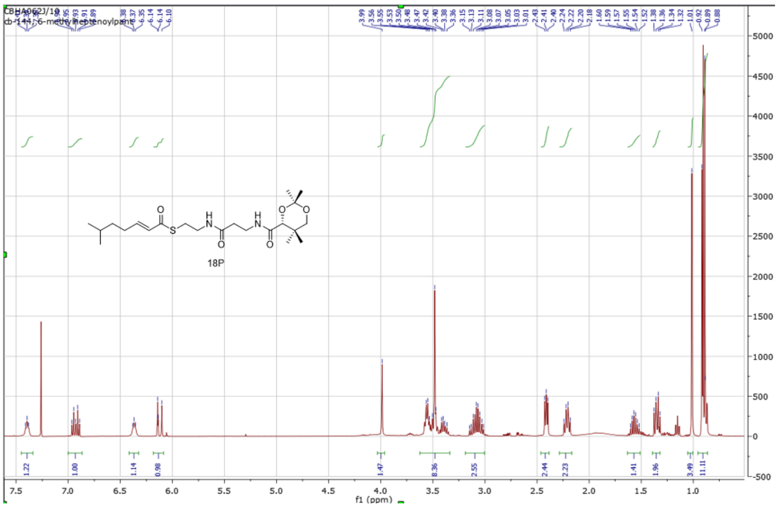

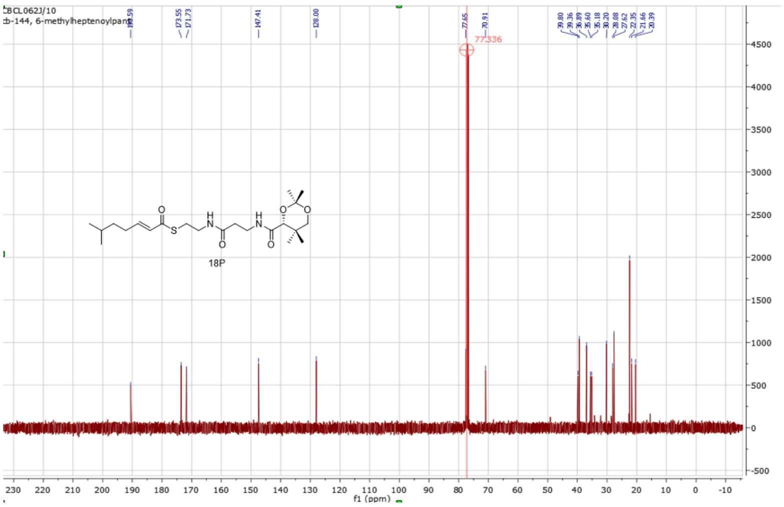

***E*-oct-2-enoyl pantetheine 19P**

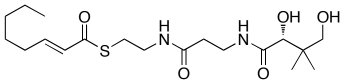

Chemical Formula: $C_{19}H_{34}N_2O_5S$
Exact Mass: 402.22

E-Oct-2-enoyl chloride was prepared from *E*-oct-2-enoic acid (**19**, 0.27g, 2.00 mmol) with thionyl chloride (0.22 g, 3.20 mmol) in THF (0.5 ml). This solution stirred for 24 h under nitrogen and solvent removed *in vacuo*.

A solution of pantetheine (0.45 g, 0.81 mmol) and DTT (0.12 g, 0.81 mmol) in THF (3 ml) was stirred in a nitrogen atmosphere at 30 °C for 3 h. After that pyridine (0.3 ml) and DMAP (12 mg, 0.09 mmol) were added to the reaction and stirred for 15 min. Then the reaction was cooled to 0 °C and oct-2-enoyl chloride was added. The mixture was stirrred for one hour at this temperature. The solvents were removed under a nitrogen flow. 6 mg of the product were purified by HPLC. ^{1}H-NMR ($CDCl_3$, 400 MHz) δ_H 0.91 (t, J = 6.7 Hz, 3H, 1-CH_3); 0.95 (s, 3H, 21-CH_3); 1.06 (s, 3H, 20-CH_3); 1.28-1.61 (m, 6H, 2-3-4-CH_2); 2.21 (dq, J = 1.4 Hz, J = 7.3 Hz, 2H, 5-CH_2); 2.43 (t, J = 6.0 Hz, 2H, 13-CH_2); 3,05-3.20 (m, 2H, 9-CH_2); 3.38-3.62 (m, 6H, 10-14-19-CH_2); 4.01 (s,^{1}H, 17-CH); 6.13 (t, J= 4.07, ^{1}H, 7-CH); 6.17 (dt, J = 1.4 Hz, J= 15.4 Hz,^{1}H, 11-NH); 6.96 (dt, J = 6,9 Hz, J = 15.4 Hz, ^{1}H, 6-CH); 7.34 (bt, J = 5.82 Hz, ^{1}H, 15-NH). ^{13}C-NMR ($CDCl_3$, 100 MHz) δ_C 13.9 (1-CH_3), 20.4 (21-CH_3); 21.7 (20-CH_3); 22.4 (2-CH_2); 27.6 (3-CH_2); 28.1 (9-CH_2); 31.7 (4-CH_2); 32.3 (5-CH_2); 35.1 (14-CH_2); 35.5 (13-CH_2); 39.3 (18-C); 39.9 (10-CH_2); 70.9 (19-CH_2); 77.8 (17-CH); 128.1 (7-CH); 147.3 (6-CH); 171.6 (12-CO); 173.2 (16-CO); 190.6 (8-COS). ESMS m/z (%): 805.9 $[M_2]H^+$ (14 %), 403 $[M]H^+$ (100 %). HRMS m/z (%): calculated 425.2086, found 425.2089 for [M]Na+.

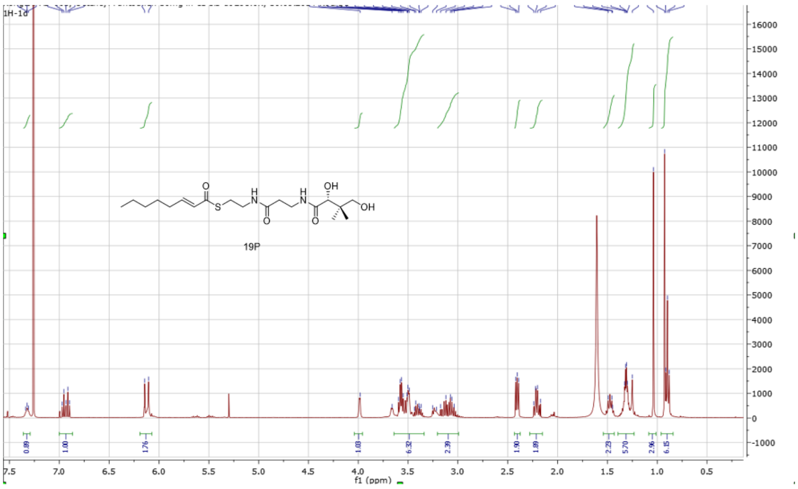

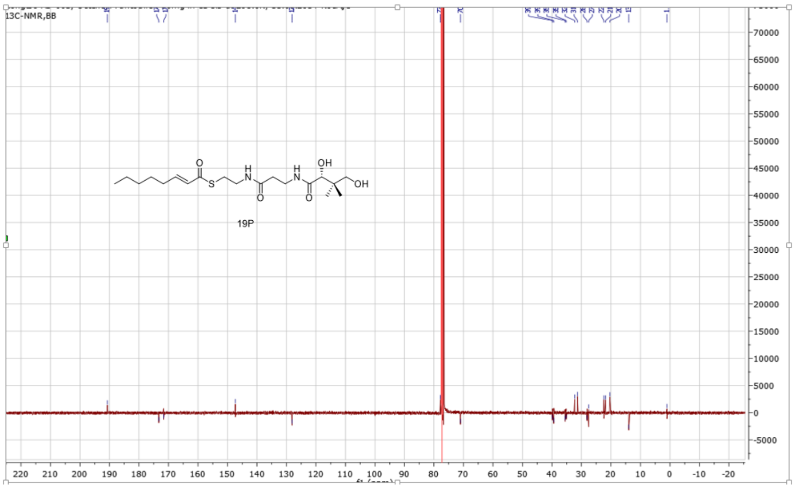

_E_-2-methyloct-2-enoyl-pantetheine 20P

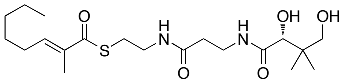

Chemical Formula: $C_{20}H_{36}N_2O_5S$
Exact Mass: 416.23

E-2-Methyloct-2-enoyl pantetheine dimethyl ketal (**20Pa,** 0.06 g, 0.003 mmol) was stirred in a mixture of CH_2Cl_2 and 10 % TFA for 20 min. The reaction was followed by TLC and LCMS. After that the solvents were evaporated. 0.01 g of the product were purified by HPLC. ^{1}H-NMR ($CDCl_3$, 400 MHz) δ_H 0.90 (tr, J = 6.9 Hz, 3H, 1-CH_3); 0.91 (s, 3H, 19-CH_3); 1.01 (s, 3H, 20-CH_3); 1.25-1.36 (m, 4H, 2-4-CH_2); 1.41-1.50 (m, 2H, 3-CH_2); 1.85 (s, 3H, 8-CH_3); 2.20 (q, J = 7.5 Hz, 2H, 5-CH_2); 2.41 (t, J = 6.6 Hz, 2H, 13-CH_2); 2.99-3.12 (m, 2H, 10-CH_2); 3.35-3.58 (m, 6H, 11-14-18-CH_2); 3.99 (s, ^{1}H, 16-CH); 6.37 (bt, J = 5.7 Hz, ^{1}H, 12-NH); 6.77 (tq, J = 1.3 Hz, J = 7.6 Hz, ^{1}H, 6-CH); 7.41 (bt, J = 6.2 Hz, ^{1}H, 15-NH). ^{13}C-NMR ($CDCl_3$, 100 MHz) δ_C 12.5 (8-CH_3); 14.1 (1-CH_3); 20.4 (19-CH_3); 21.6 (20-CH_3); 22.5 (2-CH_2); 28.1 (5-CH_2); 28.2 (4-CH_2); 28.8 (10-CH_2); 31.6 (3-CH_2); 35.1 (14-CH_2); 35.6 (13-CH_2); 39.4 (17-C); 39.8 (11-CH_2); 70.9 (18-CH_2); 77.8 (16-CH); 135.6 (7-C); 142.7 (6-CH); 171.7 (12-CO); 173.2 (15-CO); 194.2 (9-COS). ESMS *m*/*z* (%): 439 $[M]Na^+$ (2%), 417 $[M]H^+$(41%), 399 $[M - H_2O]H^+$(85%), 287 $[M - C_6H_{11}O_3]H^+$(100%). HRMS *m*/*z* (%): calculated 439.2243, found 439.2240 for [M]Na+.

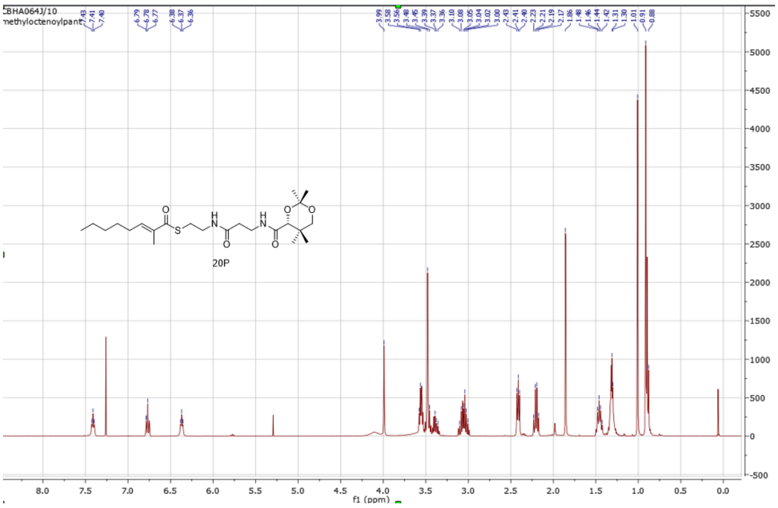

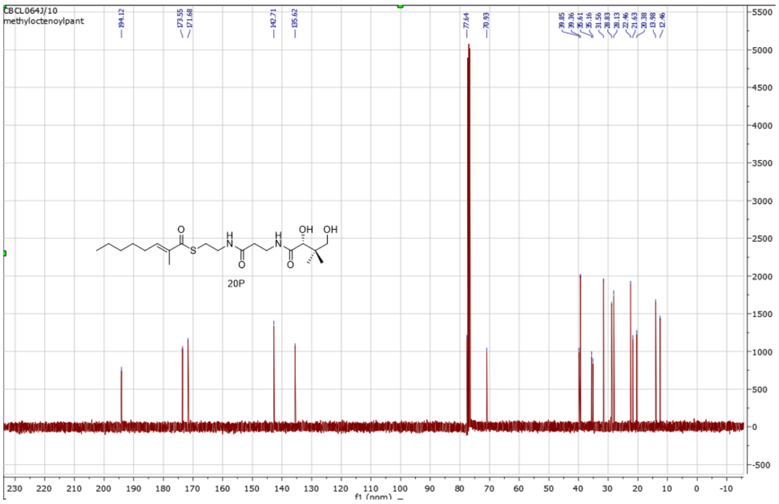

E-7-methyloct-2-enoyl-pantetheine 21P

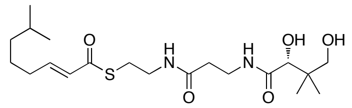

Chemical Formula: $C_{20}H_{36}N_2O_5S$
Exact Mass: 416.23

E-7-Methyloct-2-enoyl pantetheine dimethyl ketal (**21Pa**, 0.06 g, 0.003 mmol) was stirred in a mixture of CH_2Cl_2 and 10 % TFA for 20 min. The reaction was followed by TLC and LCMS. After that the solvents were evaporated. 0.01 g of the product were purified by HPLC. ^{1}H-NMR ($CDCl_3$, 400 MHz) δ_H 0.86 (d, J = 6.6 Hz, 6H, 1-3-CH_3); 0.93 (s, 3H, 18-CH_3); 1.03 (s, 3H, 19-CH_3); 1.14-1.30 (m, 2H, 3-4-CH_2); 1.42-1.60 (m, ^{1}H, 2-CH); 2.22 (q, J = 7.4 Hz, 2H, 5-CH_2); 2.41 (t, J = 5.8 Hz, 2H, 12-CH_2); 3.01-3.18 (m, 2H, 9-CH_2); 3.35-3.59 (m, 6H, 10-13-17-CH_2); 3.99 (s,^{1}H, 15-CH); 6.13 (dt, J = 1.5 Hz, J = 15.5 Hz, ^{1}H, 7-CH); 6.18 (bt, J = 5.8 Hz, ^{1}H, 11-NH); 6.93 (dt, J = 6.9 Hz, J = 15.5 Hz, ^{1}H, 6-CH); 7.34 (bt, J = 5.8 Hz, ^{1}H, 14-NH). ^{13}C-NMR ($CDCl_3$, 100 MHz) δ_C: 20.4 (19-CH_3); 21.7 (18-CH_3); 22.5 (1-1-CH_3); 25.8 (4-CH); 27.8 (2-CH); 28.1 (9-CH_2); 32.6 (3-CH_2); 35.1 (13-CH_2); 35.6 (12-CH_2); 38.5 (5-CH_2); 39.4 (16-C); 39.8 (10-CH_2); 70.9 (17-CH_2); 77.8 (15-CH); 128.1 (7-CH); 147.3 (6-CH); 171.6 (11-CO); 173.3 (14-CO); 190.6 (8-COS). ESMS m/z (%): 417 $[M]H^+$ (15%), 399 $[M - H_2O]H^+$ (60%), 287 $[M - C_6H_{11}O_3]H^+$ (100%). HRMS m/z (%): calculated 439.2243, found 439.2242 for [M]Na+.

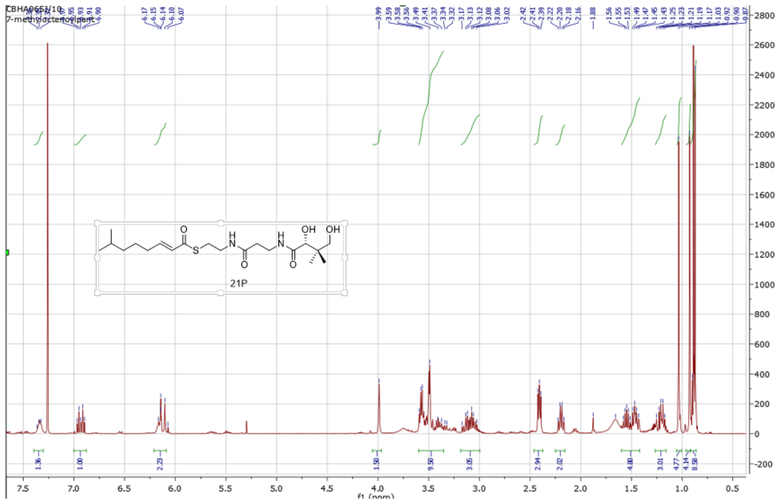

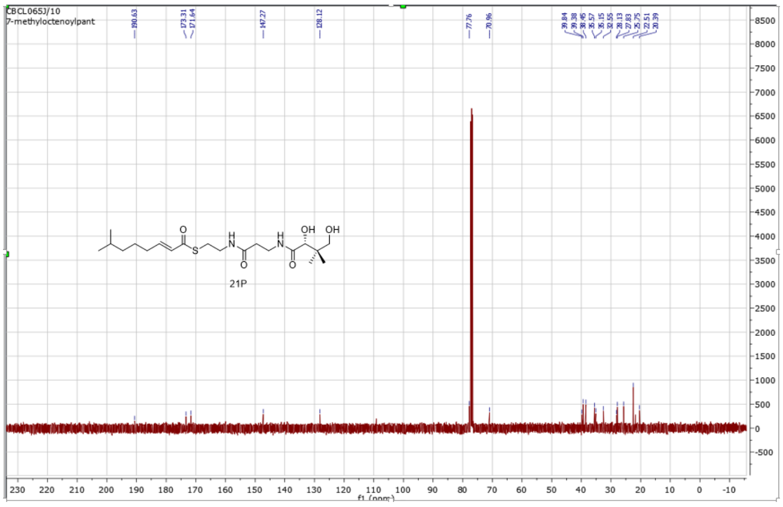

_E_-4_RS_,6_RS_-4,6-dimethyl-oct-2-enoyl-pantetheine 22P

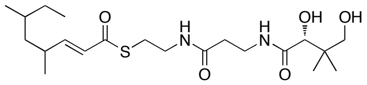

Chemical Formula: $C_{21}H_{38}N_2O_5S$
Exact Mass: 430.25

E-4*RS*,6*RS*-4,6-dimethyl-oct-2-enoyl pantetheine dimethyl ketal (**22Pa**, 300 mg) stirred in a mixture of CH_2Cl_2 and 10 % TFA for 20 min. The reaction was followed by TLC and LCMS. After that the solvents were evaporated. 0.027 g of the product were purified by HPLC. ^{1}H-NMR ($CDCl_3$, 400 MHz) δ_H 0.81-0.90 (m, 6H, 1-3-CH_3), 0.92 (s, 3H, 20-21-CH_3), 1.03 (s, 3H, 20-CH_3), 1.00-1.07 (m, 3H, 7-CH_3), 1.07-1.44 (m, 6H, 2-5-CH_2, 4-CH), 2.41 (t, 2H, *J* = 6.0 Hz, 14-CH_2), 2.34-2.46 (m, ^{1}H, 6-CH), 2.95-3.17 (m, 2H, 11-CH_2), 3.33-3.66 (m, 6H, 12-15-19-CH_2), 3.99 (s, ^{1}H, 17-CH), 6.08 (dt, ^{1}H, *J* = 1.4 Hz, *J* = 15.6 Hz, 8-CH), δ_C 6.23 (bt, *J* = 6.0 Hz, ^{1}H, NH), 6.75-6.88 (m, ^{1}H, 9-CH); 7.36 (bt, *J* = 6.0 Hz, ^{1}H, NH). ^{13}C-NMR ($CDCl_3$, 100 MHz) 11.3 (22-CH_3 and 24-CH_3), 19.2 (23-CH_3), 20.5 (20-CH_3), 21.9 (21-CH_3), 28.3 (11-CH_2), 29.5 (5-CH_2), 29.8 (19-CH_2), 31.9 (4-CH), 34.3 (6-CH), 35.3 (15-CH_2), 35.7 (14-CH_2), 39.5 (18-C), 40.0 (12-CH_2), 71.1 (19-CH_2), 77.9 (17-CH), 126.2 (9-CH), 152.5 (8-CH), 171.8 (13-CO), 173.5 (16-CO), 190.9 (10-CO). ESMS *m/z* (%): 453 $[M]Na^+$ (11 %), 431 $[M]H^+$ (62 %), 413 $[M - H_2O]H^+$ (85 %), 301 $[M - C_6H_{11}O_3]H^+$ (100 %). HRMS *m/z* (%): calculated 453.2396, found 453.2396 for [M]Na+.

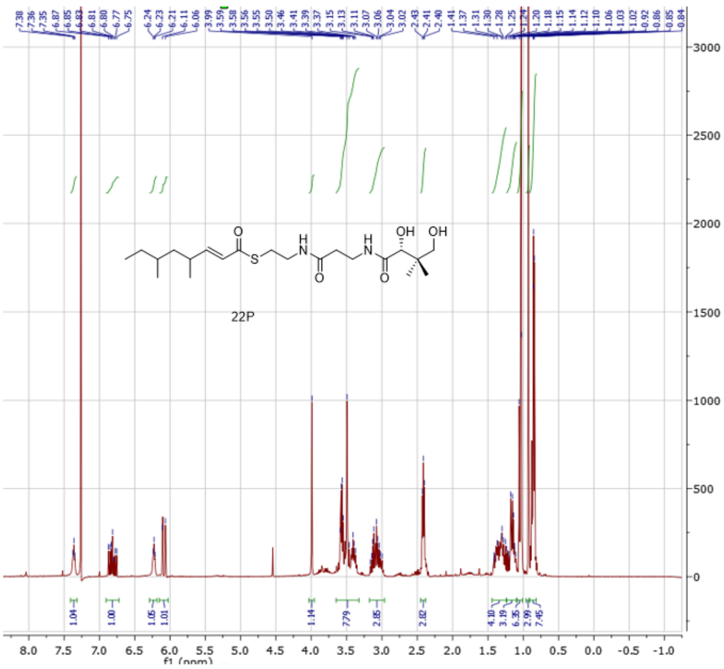

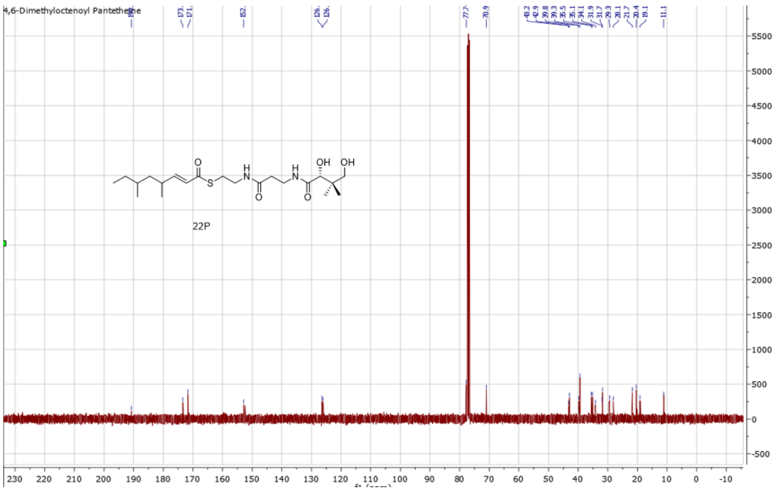

E-4*S*,6*S*-4,6-dimethyl-oct-2-enoyl pantetheine 1P

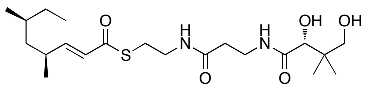

Chemical Formula: $C_{21}H_{38}N_2O_5S$
Exact Mass: 430.25

E-4*S*,6*S*-4,6-dimethyloct-2-enoyl pantetheine dimethyl ketal (**1Pa**, 300 mg) was stirred in a mixture of CH_2Cl_2 and 10 % TFA for 20 min. The reaction was followed by TLC and LCMS. After that the solvents were evaporated. 0.027 g of the product were purified by HPLC (acetonitrile). ^{1}H-NMR ($CDCl_3$, 400 MHz) δ_H 0.81-0.90 (m, 6H, 1-3-CH_3), 0.92 (s, 3H, 21-CH_3), 1.03 (s, 3H, 20-CH_3), 1.05 (m, 3H, 7-CH_3), 1.10-1.44 (m, 6H, 2-5-CH_2, 4-CH), 2.42 (t, 2H, J = 6.0 Hz, 14-CH_2), 2.37-2.41 (m, ^{1}H, 6-CH), 3.03-3.17 (m, 2H, 11-CH_2), 3.36-3.58 (m, 6H, 12-15-19-CH_2), 3.99 (s, ^{1}H, 17-CH), 6.08 (dd, ^{1}H, J = 1.4 Hz, J = 15.6 Hz, 8-CH), 6.23 (bt, J = 6.0 Hz, ^{1}H, NH), 6.79 (dd, J = 7.6 Hz, ^{1}H, 9-CH); 7.37 (bt, J = 6.0 Hz, ^{1}H, NH). ^{13}C-NMR ($CDCl_3$, 100 MHz) δ_C 11.3 (1-CH_3), 18.8 (3-CH_3), 20.2 (20-CH_3), 20.5 (21-CH_3), 21.9 (7-CH_3), 28.3 (2-CH_2), 28.5 (11-CH_2), 29.5 (4-CH), 34.3 (6-CH), 35.1 (15-CH_2), 35.9 (14-CH_2), 39.5 (18-C), 39.8 (12-CH_2), 43.3 (5-CH_2), 71.1 (19-CH_2), 77.9 (17-CH), 126.2 (9-CH), 152.5 (8-CH), 171.8 (13-CO), 173.5 (16-CO), 190.9 (10-CO). ESMS *m*/*z* (%): 453 [M]Na$^+$ (8 %), 431 [M]H$^+$(65 %), 413 [M - H_2O]H$^+$(88 %), 301 [M - $C_6H_{11}O_3$]H$^+$(100 %). HRMS *m*/*z* (%): calculated 453.2399, found 453.2392 for [M]Na+.

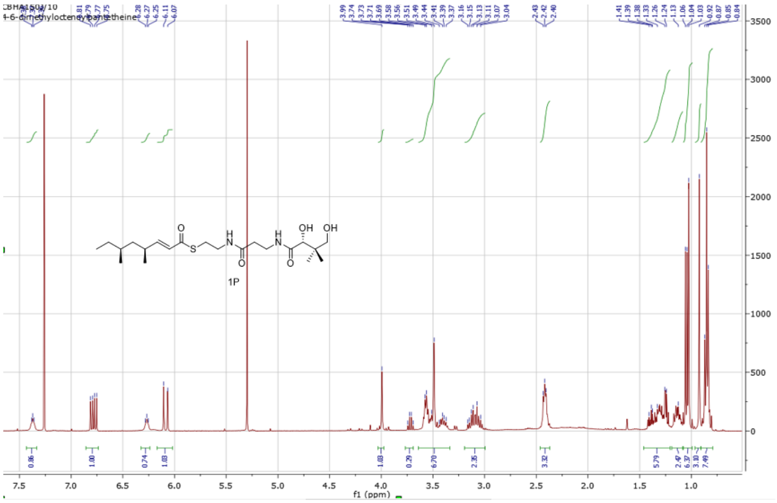

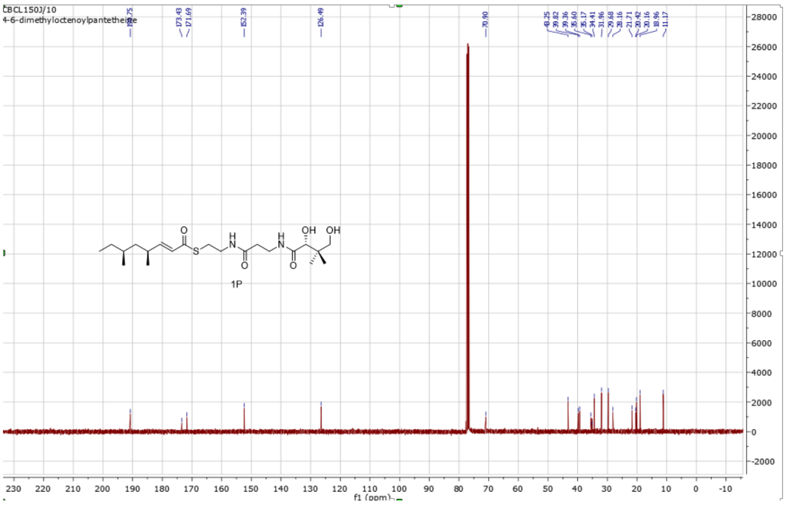

***E*-dec-2-enoyl pantetheine 24P**

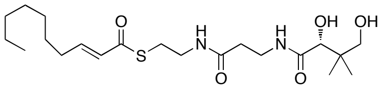

Chemical Formula: $C_{21}H_{38}N_2O_5S$
Exact Mass: 430.25

E-Dec-2-enoyl chloride was prepared from *E*-dec-2-enoic acid (**24**, 0.138 g, 1.00 mmol,) with thionyl chloride (0.110 g, 1.00 mmol) in THF (0.5 ml). This solution stirred for 24 h under nitrogen, and solvent removed *in vacuo*. A solution of pantetheine (0.30 g, 0.54 mmol,) and DTT (0.08 mg, 0.54 mmol) in THF (3 ml) was stirred in a nitrogenatmosphäre at 30 °C for 3 h. After that pyridine (0.3 ml) and DMAP (12 mg, 0.09 mmol,) were added to the reaction and stirred for 15 min. Then the reaction was cooled to 0 °C and *E*-dec-2-enoyl chloride was added to the reaction. The mixture was stirrred for one hour at this temperature. The solvents were removed under a nitrogen flow. 6 mg of the product were purified by HPLC. ^{1}H-NMR ($CDCl_3$, 400 MHz) δ_H 0.91 (t, J = 7.1 Hz, 3H, 1-CH_3); 0.95 (s, 3H, 21-CH_3); 1.06 (s, 3H, 20-CH_3); 1.28-1.33 (m, 8H, 3,4,5,6-CH_2); 1.45-1.53 (m, 2H, 2-CH_2); 2.23 (dq, J = 1.6 Hz, J = 7.2 Hz, 2H, 7-CH_2); 2.43 (t, J = 7.2 Hz, 2H, 15-CH_2); 3,05-3.20 (m, 2H, 11-CH_2); 3.37-3.62 (m, 6H, 12-16-23-CH_2); 4.01 (s,^{1}H, 19-CH); 6.13 (m, ^{1}H, 9-CH); 6.17 (dt, J = 1.5 Hz, J = 15.4 Hz, ^{1}H, 13-NH); 6.96 (dt, J = 7,0 Hz, J = 15.5 Hz, ^{1}H, 8-CH); 7.34 (bt, J = 6.0 Hz, ^{1}H, 17-NH). ^{13}C-NMR ($CDCl_3$, 100 MHz) δ_C 14.1 (1-CH_3), 20.4 (21-CH_3); 21.7 (20-CH_3); 22.6 (2-CH_2); 28.3 (11-CH_2); 29.1 (4-CH_2); 31.7 (6-CH_2); 32.3 (7-CH_2); 35.1 (16-CH_2); 35.6 (15-CH_2); 39.3 (22-C); 39.9 (12-CH_2); 70.9 (23-CH_2); 77.8 (19-CH); 128.1 (9-CH); 147.7 (8-CH); 171.6 (14-CO); 173.3 (18-CO); 190.9 (10-COS). ESMS *m/z* (%):862.9 $[M_2]H^+$(28 %), 431 $[M]H^+$(100 %), 413 $[M - H_2O]H^+$(1 %). HRMS *m/z* (%): calculated 453.2399, found 453.2399 for [M]Na+.

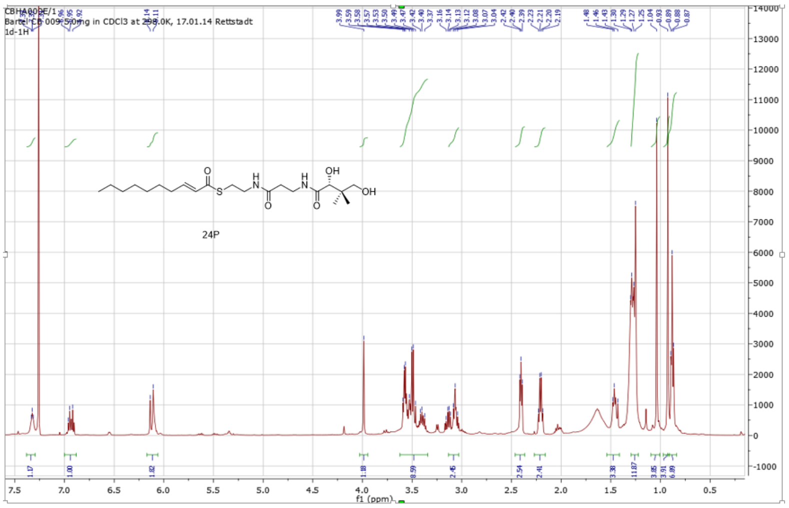

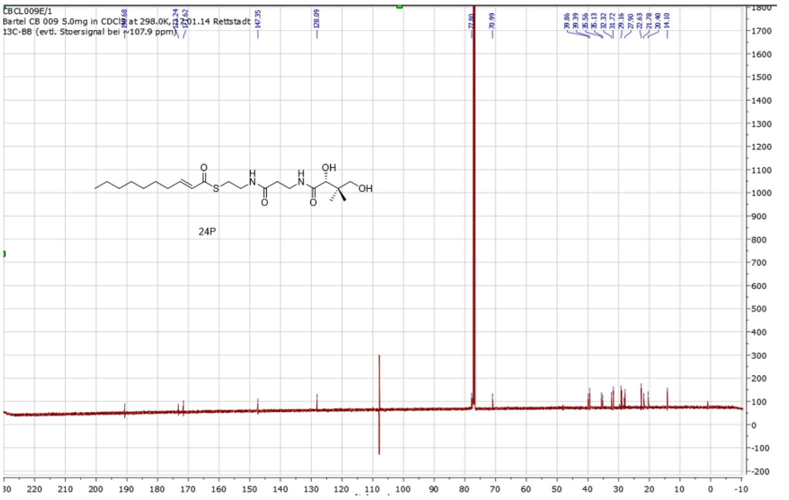

***E*-2-methyldec-2-enoyl pantetheine 25P**

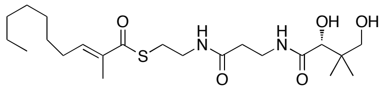

Chemical Formula: $C_{22}H_{40}N_2O_5S$
Exact Mass: 444.27

E-2-Methyldec-2-enoyl pantetheine dimethyl ketal (**25Pa**, 0.06 g, 0.003 mmol) was stirred in a mixture of CH_2Cl_2 and 10 % TFA for 20 min. The reaction was followed by TLC and LCMS. After that the solvents were evaporated. 0.01 g of the product were purified by HPLC. ^{1}H-NMR ($CDCl_3$, 400 MHz) δ_H 0.88 (tr, J = 7.2 Hz, 3H, 1-CH_3); 0.93 (s, 3H, 22-CH_3); 1.04 (s, 3H, 21-CH_3); 1.28-1.31 (m, 8H, 2-3-4-6-CH_2); 1.43-1.48 (m, 2H, 5-CH_2); 1.87 (s, 3H, 10-CH_3); 2.22 (q, J = 7.2 Hz, 2H, 7-CH_2); 2.41 (t, J = 6.2 Hz, 2H, 12-CH_2); 3.00-3.14 (m, 2H, 12-CH_2); 3.35-3.63 (m, 6H, 13-16-20-CH_2); 3.99 (s,^{1}H, 18-CH); 6.09 (bt, J = 5.6 Hz, ^{1}H, 14-NH); 6.78 (tq, J = 1.3 Hz, J = 7.4 Hz, ^{1}H, 8-CH); 7.38 (bt, J = 6.6 Hz, ^{1}H, 17-NH). ^{13}C-NMR ($CDCl_3$, 100 MHz) δ_C 12.5 (10-CH_3); 14.1 (1-CH_3); 20.4 (21-CH_3); 21.7 (22-CH_3); 22.6 (2-CH_2); 28.3 (7-CH_2); 28.5 (12-CH_2); 28.8 (4-CH_2); 29.1 (5-CH_2); 29.4 (6-CH_2); 31.8 (3-CH_2); 35.1 (16-CH_2); 35.6 (15-CH_2); 39.4 (19-C); 39.8 (13-CH_2); 70.9 (20-CH_2); 77.8 (18-CH); 135.6 (9-C); 142.8 (8-CH); 171.7 (14-CO); 173.2 (17-CO); 194.2 (11-COS). ESMS m/z (%): 467 $[M]Na^+$ (12%), 445 $[M]H^+$ (27%), 427 $[M - H_2O]H^+$ (67%), 315 $[M - C_6H_{11}O_3]H^+$ (100%). HRMS m/z (%): calculated 467.2556, found 467.2556 for [M]Na+.

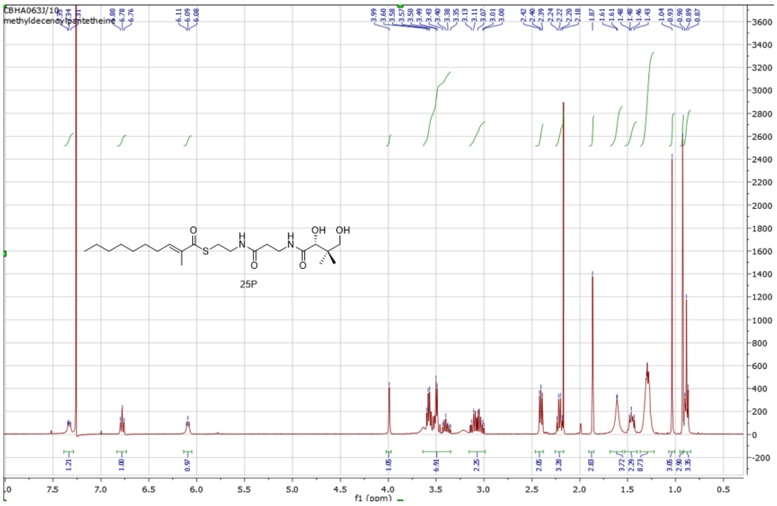

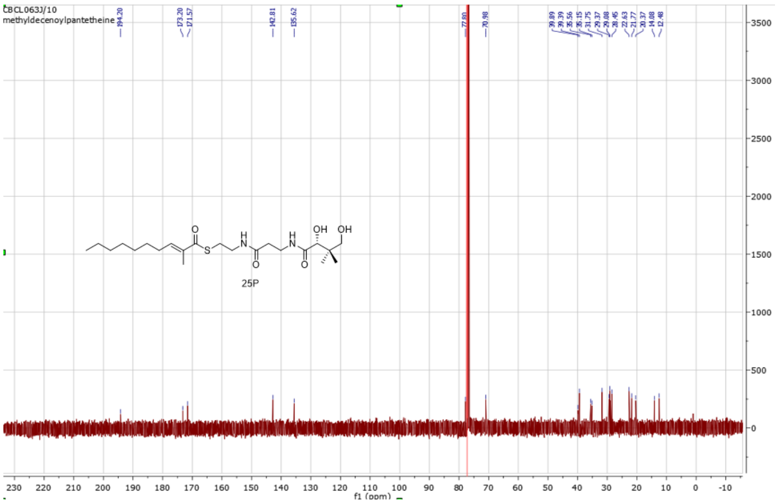

1.1 Synthesis of Compounds for Stereochemical Investigations

1.1.1 Testing the stereochemistry of the α-methyl of the reduction of the diketide substrate

NMR assays were performed with tiglyl-SNAC **5S** (2.0 mg, 10 mmol), NADPH (8 mg, 10 mmol), tris buffer pH 8.5 (50 µl, 1M stock), glycerol (20% v/v) and the isolated ER domain (380 mU, 100 µl of stock) in water (total volume 1 ml). The progress of the reaction was monitored by LCMS. After 24 h further NADPH (8 mg, 10 mmol) was added to the reaction. After a further 17 h the protein was precipitated with CH_2Cl_2 (1 ml) and centrifuged (3000 rpm, 20 min). The organic layer was removed and the aqueous layer was further extracted with CH_2Cl_2 (2 × 1 ml). The organic fractions were combined and the solvent was removed under a flow of dry N_2 and then dried in *vacuo* for a further 2 h. To the dried sample was added water (450 µl) and aqueous NaOH (2M, 50 µl). The sample was left for 90 min at RT and then acidified with aqueous HCl (2M, 100 µl) to pH 3 and extracted with $CDCl_3$ (4 × 200 µl). The organics were combined, dried with $MgSO_4$ and then filtered through $MgSO_4$ directly into an NMR tube. (1*R*,2*R*)-(+)-1,2 diphenylethylenediamine was titrated (100 mM stock) to optimize resolution of the obtained spectra.

1.1.2 Production of NADPD stereoisomers

4'*S*-NADPD 27[29]

A solution of sodium bicarbonate (6.3 mg, 75 µmol), D_7-glucose (8.0 mg, 45 µmol) and NADP + (16.7 mg, 23 µmol) were titrated to a pH 7.5 in water (1.5 ml). Glucose dehydrogenase from *Pseudomonas sp* (35 U) was added and the reaction was incubated at RT and the progress of the reaction was monitored by UV (340 nm). On completion the reaction was confirmed by MS (747 (100, [M]H+) and was used without further purification (final concentration of NADPD 15 mM).

4'*R*-NADPD 28[30]

A stock solution of alcohol dehydrogenase NADP + dependent, from *thermoanerobium brockii* (TbADH) was prepared in tris pH 7.2, 25 mM (0.085 U/µl) and was added (25 µl) to a solution of NADP + disodium trihydrate (17. mg, 20.8 µmol), *iso*propanol (300 µl) and tris buffer pH 9 (25 mM, 7.5 ml), this solution was incubated at 43 °C and the progress of the reaction was monitored by UV (340 nm). When no further change in absorption was observed the acetone and remaining *iso*propanol was removed *in vacuo* and the product was confirmed by MS (747 (100, [M]H+). This was used without and further purification (final concentration of NADPD 3.1 mM).

1.1.3 Synthesis of mandelate ester standards

***RS*-2-methyl butyryl methyl *S*-mandelate 30**[31]

2-Methylbutyric acid (102 mg, 1.0 mmol) and *S*-methyl mandelate (208 mg, 1.3 mmol) were stirred at 0 °C in CH_2Cl_2 (3 ml). To this was added DMAP (4 mg) and EDCI (240 mg, 1.25 mmol) and this was then stirred at RT and followed by TLC. On completion, aqueous HCl (0.5 M, 5 ml) was added and this was extracted with CH_2Cl_2 (3 × 5 ml). The organic fractions were combined and concentrated in *vacuo*. The resulting oil was purified by column chromatography (hexane:ethyl acetate 3:1) to yield the desired product as a inseparable mixture of diastereomer (A:B 1:1, colourless oil, 206 mg, 832 µmol, 83%, Rf 0.46, hexane:ethyl acetate, 3:1): δ_H (400 MHz, $CDCl_3$); 7.50-7.45 (2H, m, H_2-10 A and B), 7.41-7.36 (3H, m, H_2-11 H-12 A and B), 5.93 (0.5H, s, H-6 A), 5.92 (0.5H, s, H-6 B), 3.72 (1.5H, s, H_3-8 A), 3.72 (1.5H, s, H_3-8 B), 2.54 (1H, qt *J* 7.0, H-2), 1.85-1.69 (1H, m, H-3), 1.63-1.47 (1H, m, H-3), 1.24 (1.5H, d *J* 7.0, H_3-5 A), 1.19 (1.5H, d *J* 7.0, H_3-5 B), 0.99 (1.5H, t *J* 7.4, H_3-4 B), 0.92 (1.5H, t *J* 7.4, H_3-4 A); δ_C (100 MHz, $CDCl_3$); 176.1 (C-1B), 176.0 (C-1A), 169.5 (C-7 A and B), 134.0 (C-9 129.1 (C-12), 128.8 (C-11), 127.7 (C-10), 74.2 (C-6B), 74.1 (C-6A), 52.6 (C-8A), 52.5 (C-8B), 40.9 (C-2A), 40.3 (C-2B), 26.8 (C-3B), 26.6 (C-3A), 16.6 (C-5A), 16.3 (C-5B), 11.5 (C-4A), 11.4 (C-4B); MS (ES +): *m/z* (%) 523 (13, [M_2 + Na]+), 501 (10, [M_2 + H]+), 251 (63, [M]H+), 191 (81, [M + H - $COOHCH_3$]+), 149 (100, $CH_3CH_2CHCH_3COOH$]+), 212 (23, [M + H_2 - $CH_3CH_2CHCH_3COOHOCH_3$]+).

Ethyl 3*R*-hydroxyl 2*R*-methylbutyrate 32[32]

BuLi (1.6 M in hexanes, 4.2 ml, 6.7 mmol), was added dropwise to a solution of freshly distilled diisopropylamine (894 µl, 6.4 mmol) in THF (6.5 ml) at -78 °C. The resulting solution was stirred for 1 hour. A solution of ethyl (*R*)-3-hydroxybutyrate (400 mg, 3.0 mmol) and hexamethylphosphoramide (900 µl) in THF (2 ml) was added dropwise to the

reaction over 15 min. The mixture was then warmed to RT for 20 min and then cooled back to -78 °C. Iodomethane (236 µl, 3.79 mmol) was added to the reaction and this was stirred at 0 °C for 2 h. On completion saturated NH_4Cl (8 ml) was added to the reaction and then was acidified with aqueous HCl (2 M) until pH 7 was reached. The solution was then extracted with diethyl ether (3 × 10 ml) and the organics were combined, dried ($MgSO_4$) and concentrated *in vacuo*. The resulting oil was purified by silica chromatography (ethyl acetate:hexane 1:1) to yield the desired product (a pale yellow oil, 306 mg, 2.1 mmol, 69%, Rf 0.47, ethyl acetate:hexane 1:1); δ_H (200 MHz, $CDCl_3$); 4.17 (2H, q *J* 7.1, H_2-7), 3.88 (1H, qdd *J* 6.4 6.4 6.4, H-3), 2.69 (1H, br d *J* 5.8, H-5), 2.44 (1H, p *J* 7.2, H-2), 1.31-1.16 (9H, m, H_3-4 H_3-6 H_3-8); δ_C (100 MHz, $CDCl_3$); 176.1 (C-1), 69.6 (C-3), 60.7 (C-7), 47.1 (C-2), 20.9 (C-4), 14.3 (C-8), 14.3 (C-6); MS (ES +): *m/z* (%) 147 (100, [M]H+), 129 (11, [M + H - H_2O]+), 101 (8, [M + H - CH_3CH_2OH]+).

Ethyl 2*R*-methyl 3*R*-(methylsulfonyl)oxybutanoate 33

Mesyl chloride (325 µl, 4.2 mmol) was slowly added to a solution of ethyl 3-(*R*)- hydroxy-2-(*R*)-methyl butyrate (307 mg, 2.1 mmol) and pyridine (1.7 ml, 10.5 mmol) in CH_2Cl_2 (5 ml) at 0 °C. The reaction was stirred at 0 °C and followed by TLC and on completion water (10 ml) was added to the solution and then this was extracted with CH_2Cl_2 (3 × 10 ml). The organics were combined, dried ($MgSO_4$) and concentrated *in vacuo*. The resultant oil was further purified by silica column chromatography (hexane:ethyl acetate 3:1) to yield the desired product (colourless oil, 360mg, 1.6 mmol, 76%, Rf 0.22 hexane:ethyl acetate 3:1); δ_H (400 MHz, $CDCl_3$); 4.97 (1H, dq *J* 7.8 6.3, H-3), 4.16 (2H, q *J* 7.1, H_2-7), 2.99 (3H, s, H_3-5), 2.77 (1H, qd *J* 7.2 7.2, H-2), 1.44 (3H, d *J* 6.4, H_3-4), 1.27 (3H, t *J* 7.1, H_3-8), 1.20 (3H, d *J* 7.2, H_3-6); δ_C (100 MHz, $CDCl_3$); 173.4 (C-1), 80.3 (C-3), 61.1 (C-7), 45.3 (C-2), 38.5 (C-5), 18.5 (C-4), 14.3 (C- 8), 12.9 (C-4); MS (ES +): *m/z* (%) 471 (9, [M_2]Na+), 449 (25, [M_2]H+), 225 (8, [M]H+), 170 (14), 129 (100, [M + H - MsOH]+); υ_{max} (oil)/cm^{-1} 2986 (C-H), 2953 (C-H), 1729 (C=O), 1458 (C-H), 1351 (S=O), 1333 (S=O), 1205 (S=O), 1169 (S=O), 1073 (C- O), 1027 (C-O); HRMS (ESI +): calcd for [M]H+ , $C_8H_{16}NO_5SNa$: 255.0797: found 255.0799.

2*R*,3*S*-[3-^{2}H]-30

Ethyl 2-methyl-3-(methylsulfonyl)oxybutanoate (273 mg, 1.2 mmol) was dissolved in anhydrous diethyl ether (1 ml) and was added to $LiAlD_4$ (76 mg, 1.8 mmol) under dry N_2. The reaction was warmed to RT and stirred for a further 2 h. On completion saturated sodium sulfate (3 ml) was added and the solution was extracted with diethyl ether (3 × 5 ml), dried ($MgSO_4$) and concentrated carefully in *vacuo*. This yielded 189 mg of the crude material (colourless oil) that was used without further purification or characterization.

Jones reagent was prepared by taking chromium oxide (1.6 g) and adding conc. H_2SO_4 (1.3 ml) and then the solution was diluted to a final volume of 6 ml with water. The freshly prepared Jones reagent was added to the previously produced oil (189 mg) in acetone (4 ml) at 0 °C until a permanent red colour was seen. The reaction was stirred for 1 hour and then excess Jones reagent was quenched by slowly adding *iso*-propanol. The acetone was removed in *vacuo* and the remaining green precipitate was redissolved in water (5 ml) and extracted with chloroform (3 × 5 ml). The organics were dried and concentrated *in vacuo*. This yielded a colourless oil (117 mg, Rf 0.42, hexane:ethyl acetate 8:2) which was used without further purification or characterization.

The oil (117 mg) was then dissolved in CH_2Cl_2 (2 ml) and cooled to 0 °C and to it was added *S*-methyl mandelate (237 mg, 1.4 mmol), DMAP (5 mg) and EDCI (234 mg, 1.4 mmol). The reaction was warmed to RT and followed by TLC. On completion aqueous HCl (2M, 2 ml) was added and the reaction was extracted with CH_2Cl_2 (3 × 3 ml), dried ($MgSO_4$) and concentrated *in vacuo*. The resulting oil was purified by silica flash chromatography (hexane:ethyl acetate 3:1) and yielded the desired product (a pale yellow oil, 42 mg, 167 µmol, 14 % across the three steps, Rf 0.48, hexane:ethyl acetate 3:1): δ_H (400 MHz, $CDCl_3$); 7.49-7.46 (2H, m, H_2-10), 7.40-7.37 (3H, m, H_2-11 H-12), 5.93 (1H, s, H-6), 3.72 (3H, s, H_3-8), 2.53 (1H, p *J* 7.1, H-2), 1.73 (1H, p *J* 7.4 *JHD* 1.8, H-3), 1.24 (3H, d *J* 7.0, H_3-5), 0.91 (3H, d *J* 7.1, H_3-4); δ_C (100 MHz, $CDCl_3$); 176.1 (C-1), 169.5 (C-7), 134.1 (C-9), 129.3 (C-12), 128.9 (C-11), 127.7 (C-10), 74.2 (C-6), 52.7 (C-8), 40.9 (C-2), 26.4 (C-3, *J*CD 19.6), 16.6 (C-5), 11.6 (C-4); MS (ES +): *m/z* (%) 252 (83, [M]H+), 192 (11, [M + H - $COOHCH_3$]+), 149 (100, [M + H- $CH_3CHDCHCH_3COOH$]+); υmax (oil)/cm^{-1} 2963 (C-H), 2925 (C-H), 2851 (C-H), 1755 (C=O), 1737 (C=O), 1498 (C=C-C), 1455 (C=C-C), 1436 (C=C-C), 1215 (C-H aromatic), 1533 (C-H aromatic), 1124 (C-H aromatic), 1084 (C-H aromatic),732 (C-H aromatic), 696 (C-H aromatic); HRMS (ESI +): calcd for [M]Na+, $C_{14}H_{17}O_4DNa$: 274.1166: found 274.1166.

2.0 Production and Purification of ER protein

All media and Gilson tips were autoclaved (steam, 121 °C, 10 min) before use. Media used was LB agar [Agar (3 g), tryptone (2 g), yeast extract (1 g), NaCl (1 g) and water (200 ml)] and 2TY media [Tryptone (16 g), yeast extract (10 g), NaCl (5 g) and water (1 L)] and kanamycin was added to the media (100 µL of stock (30 mg/ ml) per 100 ml of media).

2.1 SQTKS isolated ER domain

The sequence of SQTKS[14] was used to design an *E. coli* optimised sequence which was used as the template for the amplification of the ER domain. Primers for the ER domain with added bases in bold and restriction sites underlined. FW-Nde1: **CAC CAT ATG** GAA CCC TTT CAT CAG CCG GGG AAG C RV-BamH1: **AAA GGA TCC** TTA TGG CGC GGT GAT GAC AAT TTT GC. The PCR product was cloned into pET28a and the plasmid transformed into *E. coli* BL21 DE3 (Novagen), 100 µl of the culture was spread on a LB agar plate with kanamycin and this was incubated overnight at 37 °C. A single colony was taken to form a starter culture (50 ml 2TY media with kanamycin added), which was incubated overnight at 37°C with shaking. The starter culture was then used to inoculate 2TY media (1:100 starter culture:media dilution per flask). The OD was monitored and when it reached ~0.6 A the flasks were cooled to 16 °C and IPTG solution (1 M, 50 µl, 0.5 mM final IPTG concentration) and the flasks were left overnight at 16 °C with shaking (250 rpm).

On completion the media was centrifuged (7000 rpm, 15 min) and the pellet was collected. The cells were resuspended in nickel column wash buffer (50 ml buffer per 1 l of media, [50 mM Tris pH 8, 150 mM NaCl, 10% glycerol (v/v) and 20 mM imidazole]). The suspension was then sonicated (6.5 min, 30 seconds 20% power, 30 seconds rest) on ice and then centrifuged (17000 rpm, 30 min). The supernatant was taken, filtered (0.45 µm filter) and purified on a Ni^{2+} affinity column using a linear gradient with the elution buffer [50 mM Tris pH 8, 150 mM NaCl, 10% glycerol (v/v) and 0.5 M imidazole] to 100%. The fractions were analyzed by SDS page and fractions containing protein of approximately the right mass were combined and concentrated to ~2 ml using centrifugal ultrafiltration (10 kDa cut-off).

The concentrated protein was purified using size exclusion chromatography. The protein was loaded directly onto the column and eluted in the size exclusion elution buffer [50 mM Tris pH 8, 150 mM NaCl, 20% glycerol (v/v)]. The fractions were analyzed by SDS page and fractions containing protein of approximately the right mass and high purity were combined together and concentrated to ~2 ml using centrifugal ultrafiltration (10 kDa cutoff). The concentration of protein was estimated using a Bradford assay and the activity (U) of the enzyme was measure. The protein was then divided into aliquots (0.2 U/µl) and stored long term at -80 °C.

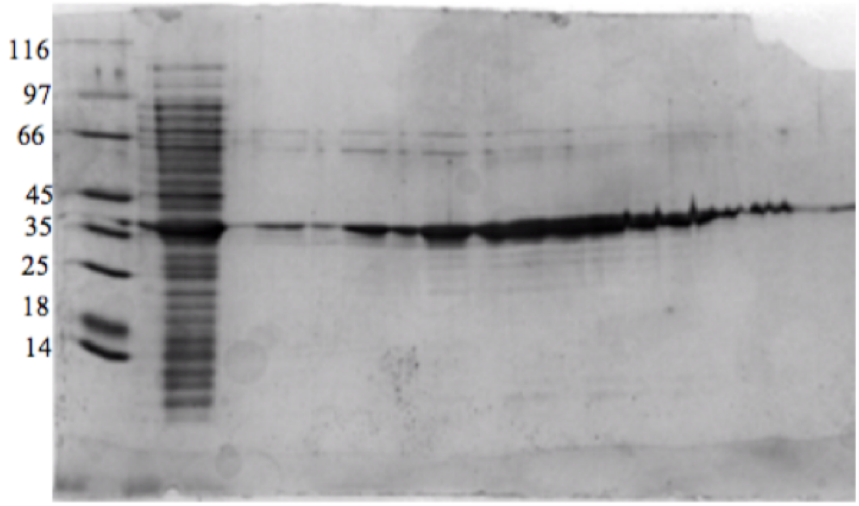

Fig S2.1.1. Nickel affinity purification of SQTKS ER domain: Lane 1, molecular weight markers / kDa; Lane 2, cell-free lysate; lanes 3-9 fractions eluted with imidazole gradient.

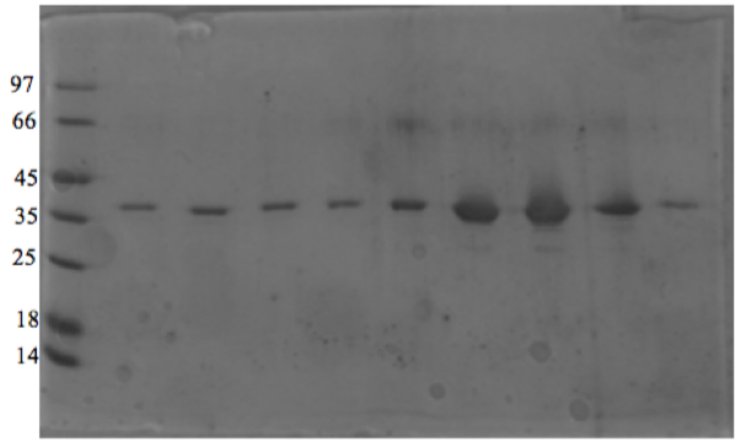

Figure S2.1.2 SDS-PAGE showing the purification of isolated ER domain by size exclusion chromatography.

The identity og the protein was confirmed by MALDI analysis of digested protein fragments obtained directly from the SDS-PAGE gel.

MEPFHQPGKLLQMGIKTPGLIDTLQFSKTDATDNLPNDYIEIEPKAFGLNFRDV
MVAMGQLEESIMGFECAGVVRRVGPSSAGHNIKVGDRVCALLGGQWTNTVR
VHWHSVAPIPQAMDWETAASIPIVFVTAYISLVKIARMQAGETVLIHAASGGVG
QAAIILAKHVGAEIFATVGTDEKRDLLIKEYKIPDDHIFSSRNALFAKSIRQRTNG
KGVDVVLNCLAGGLLQESFDCLADFGRFIEIGKRDIELNHCLNMGMFARSATFT
AVDLIAIGRDRSYMFAEALPKIMTLLQEKAIRPVTPISIYKIGDIETAFRLMQAGK
HMGKIVITAP

Figure 2.1.3. Red text shows peptide sequences detected by MS from MALDI analysis of digested protein.

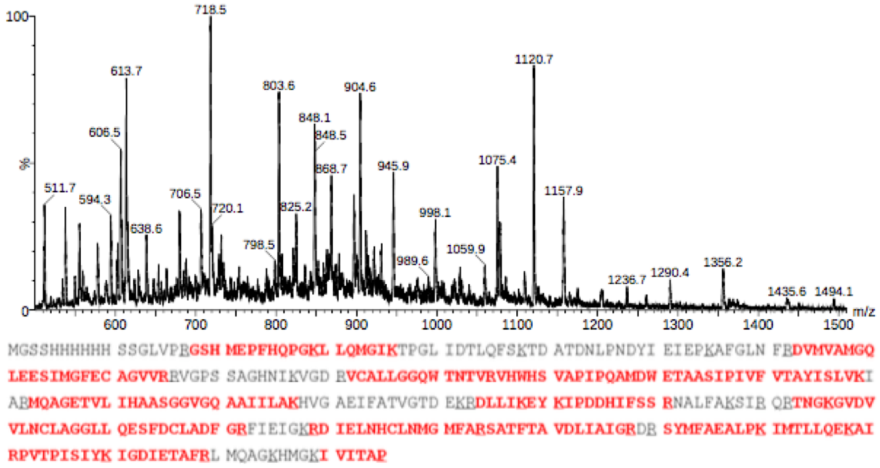

Fig 2.1.4. Combined ES+ spectrum from digestion of ER and matched peptides. Matched Peptides are highlighted in red and cleavage sites are underlined.

2.2 Defining and measuring the activity units for the isolated ER domain

1 unit of activity is defined as 1 µM·min^{-1} tigloyl pantetheine reduction by NADPH at 30 °C. The freshly produced protein was analyzed by a standard UV assay (section 3.2.2) and the produced enzyme was diluted to the appropriate concentration (typically 0.2 mU/µl) with the size exclusion buffer.

2.3 Bradford Assay

Standard solutions of bovine serum albumin (0.1-2 ml/ ml) in size exclusion buffer [50 mM Tris pH 8, 150 mM NaCl, 20% glycerol (v/v)] were prepared by serial dilution. 100 µl of the standards were mixed with Bradford dye reagent (1 ml) and incubated for 15 min at RT. The absorption of each sample was measured at 595 nm against a standard (size exclusion buffer 100 µl, Bradford dye reagent, 1 ml) to construct a standard concentration curve. A sample of the protein to be quantified (20 µl) was diluted in size exclusion buffer (80 µl) and treated with Bradford dye reagent (1 ml). This was incubated at room temperature for 15 min and then the absorption was measured at 595 nm. This was compared to the previously prepared concentration curve to calculate the amount of protein that had been produced.

2.4 Evidence for Dimeric State of ER in Solution

The protein solution was loaded onto the column (GE Healthcare HiLoad 26/600 Superdex 200 pg) and eluted with 50 mM Tris pH 8, 150 mM NaCl, 20% glycerol (*v/v*). In the run no concentration change take place. The flow of the FPLC was constant 1mL/min over 300 min.

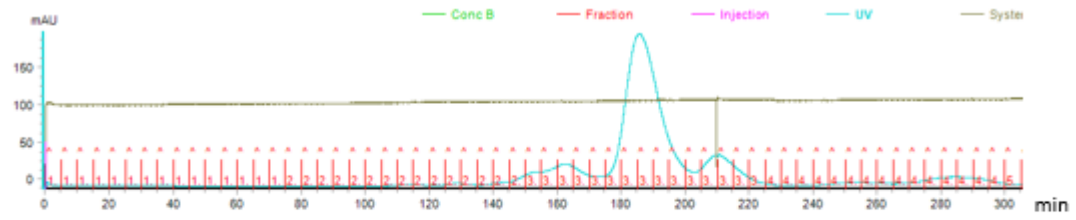

Calibration:
For the calibration a mixture of three different proteins (Carbonic Anhydrase, BSA, Apoferritin) was loaded on the column. The mixture was loaded onto the column (GE Healthcare HiLoad 26/600 Superdex 200 pg) and eluted with 50 mM Tris pH 8, 150 mM NaCl, 20% glycerol (*v/v*). The flow of the FPLC was constant 1mL/min over 300 min.

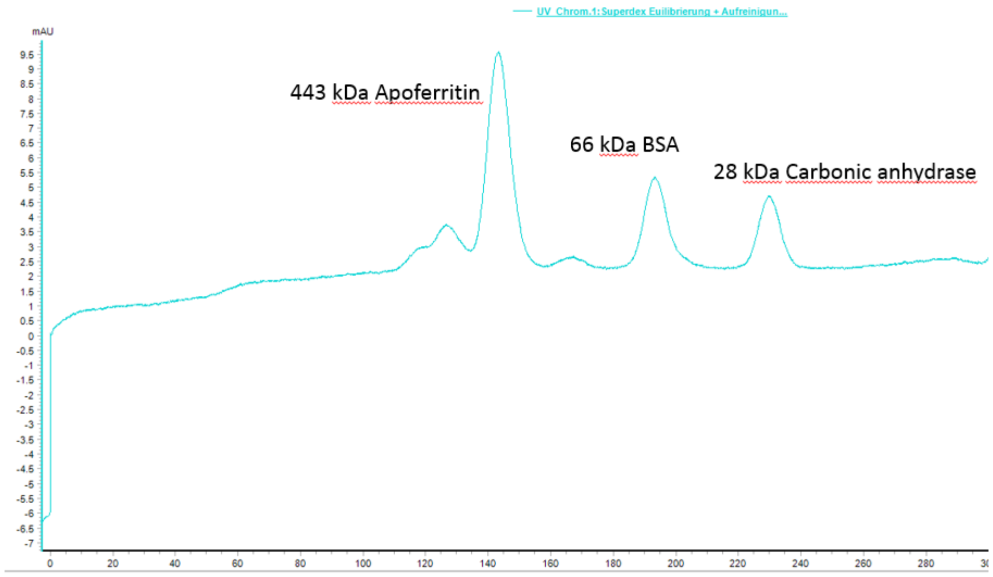

3.0 Kinetic and Inhibition Assay procedures

3.1 LCMS assay of ER

A sample of reaction mixture (20 µl) was mixed with acetonitrile (80 µl) to precipitate the protein and then centrifuged (13,000 rpm, 1 min). The supernatant was analyzed directly by LCMS using a standard LCMS profile (Section 6.1).

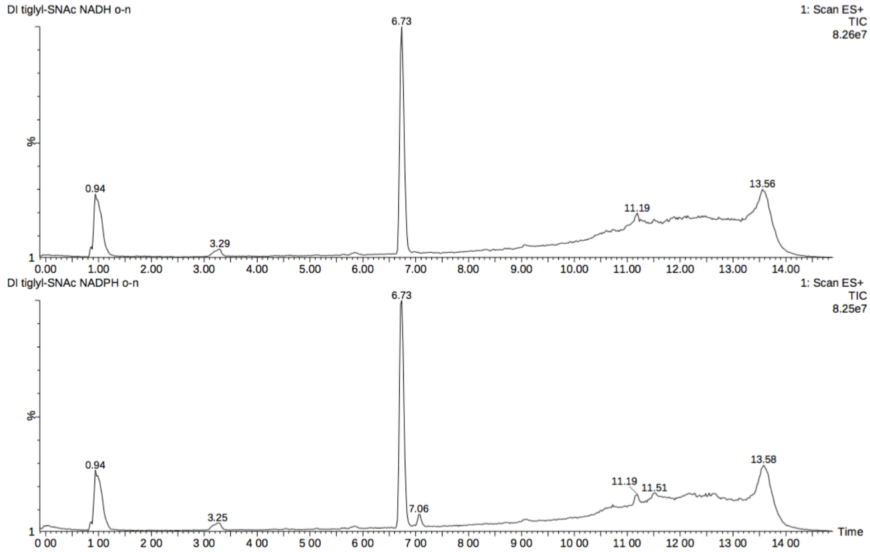

Fig S3.1.1. Enzyme reactions contained 0.5 mM tiglyl-SNAC (6.73 min peak), 0.5 mM NAD(P)H, 0.1 mg SQTKS ER per 200 µL reaction, and 50 mM Tris-HCl pH 7.5. After an overnight incubation at 30 ˚C, protein was precipitated from the reaction mixtures by the addition of 10 µL of assay mixture to 40 µL of acetonitrile and removed by centrifugation. A portion of the supernatant was analysed by LCMS. Use of NADH (top chromatogram) gave no new product. Use of NADPH (bottom chromatogram) resulted in the formation of product (7.06 min).

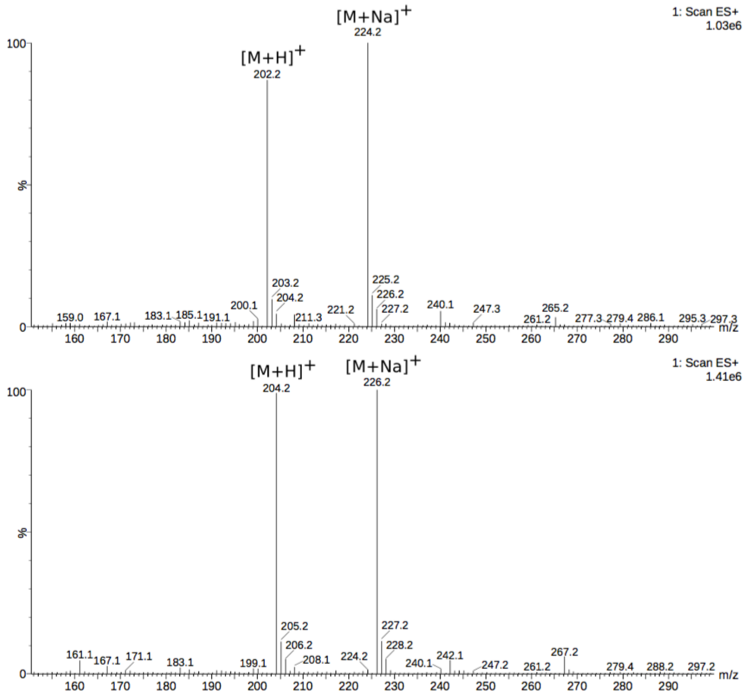

Fig S3.1.2. ESMS spectra for 6.7 min peak (tigloyl SNAC, top) and 7.06 min peak (methylbutyryal SNAC, bottom).

3.2 Kinetic Assays

3.2.1 Comparative Kinetic Assays

Comparative assays of SNAC and Pantetheine activity were conducted using a Spectramax Plus-382 96-well plate reader at 340 nm over several hours at 30 ˚C. Four times more enzyme was used for SNAC assays than for Pantetheine assays in order to observe meaningful conversion rates.

Assays with acyl-SNAC substrates were performed using stocks of acyl-SNAC (10 mM), NADPH (10 mM), Tris pH 8.5 (1 M), glycerol (50% *v/v*) and SQTKS ER (0.2 mU/µl). Assays were conducted at (final concentration, volume of stock): acyl-SNAC (0.5 mM, 10 µl), NADPH (0.75 mM, 15 µl), Tris buffer pH 8.5 (50 mM, 10 µl), glycerol (20% *v/v*, 80 µl), SQTKS ER (4 mU, 20 µl) and water (65 µl), to a total volume of 200 µl.

Assays with acyl-PANT substrates were performed using stocks of acyl-PANT (10 mM), NADPH (10 mM), Tris pH 8.5 (1 M), glycerol (50% v/v) and SQTKS ER (0.2 mU/µl). Assays were conducted at (final concentration, volume of stock): acyl-PANT (0.5 mM, 10 µl), NADPH (0.75 mM, 15 µl), Tris buffer pH 8.5 (50 mM, 10 µl), glycerol (20% *v/v*, 80 µl), SQTKS ER (1 mU, 5 µl) and water (80 µl), to a total volume of 200 µl.

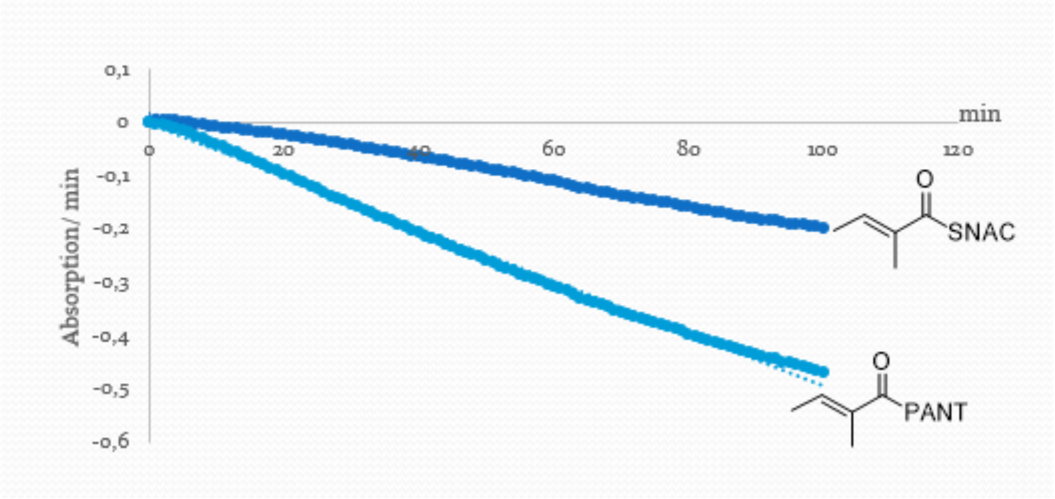

Fig S3.2.1. Kinetic analysis of the substrate activity of 5P vs 5S. Note that the 5S assay contained 4 x as much enzyme as the 5P assay.

3.2.2 Quantitative Kinetic Assays

Assays were run in 500 µl quartz cuvettes with a path length of 1 cm and the absorption was measured at 340 nm against a standard cuvette at 30 ˚C. Assays with acyl-PANT substrates were performed using stocks of acyl-PANT (5 mM), NADPH (10 mM), Tris pH 8.5 (1 M), glycerol (50% *v/v*) and SQTKS ER (0.08 U/µl). Assays were conducted at (final concentration, volume of stock): acyl-PANT (62.5 µM - 500 µM, 5 - 40 µl); NADPH (250 µM, 10 µl); SQTKS ER (20 µL, 4 mU); Buffer (330 - 365 µl) in a total volume 400 µl. Reactions were run in triplicate. Initial rates were plotted *vs* substrate concentration (section 3.4).

3.4 Kinetic Data for Substrate Variants

Initial rate data was collected over a range of substrate concentrations (0-100 μM as appropriate) and kinetic parameters were obtained by direct fit of the data to the Michaelis Menten equation (see below). All data is *apparent* at fixed NADPH concentration.

	Substrate	K_M / μM	k_{cat} / s^{-1}	k_{cat} / K_M s^{-1} μM^{-1}
**7P**	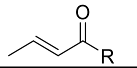	0.12	3.7	30.8
**5P**	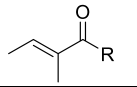	0.69	8.9	12.9
**8P**	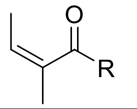	0.44	11.4	25.9
**9P**	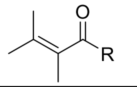	0	0	0
**10P**	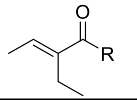	0.16	2.58	16.1
**11P**	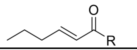	0.23	25.9	112.6
**12P**	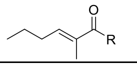	0.54	415	768.5
**13P**	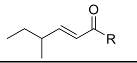	0.18	5.9	32.8
**14P**	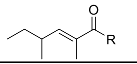	1.85	169	91.4
**15P**	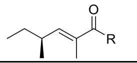	0.41	48.8	119.0
**16P**	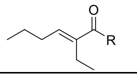	0.19	4.71	24.8
**17P**	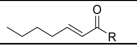	0.18	7.11	39.5
**18P**	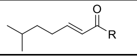	0.52	5.6	10.8
**19P**	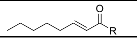	0.6	9	15
**20P**	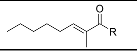	1.70	15.7	9.2
**21P**	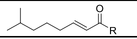	4.70	0.85	0.2
**22P**	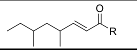	0.18	2	11.1
**1P**	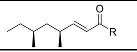	0	0	0
**24P**	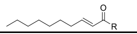	0.36	10	27.8
**25P**	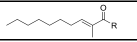	0.2	6	30

Table S3.4: Kinetic data different pantetheine substrates
R = S-Pantetheine residue

Raw Kinetic DATA - direct fits to the MM equation

7P Crotonylpantetheine

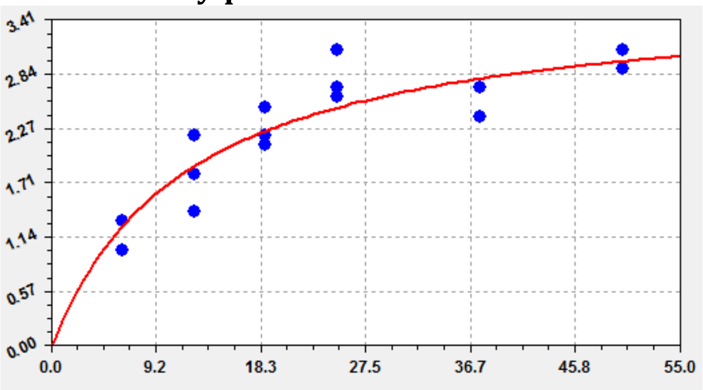

5P Tigloylpantetheine

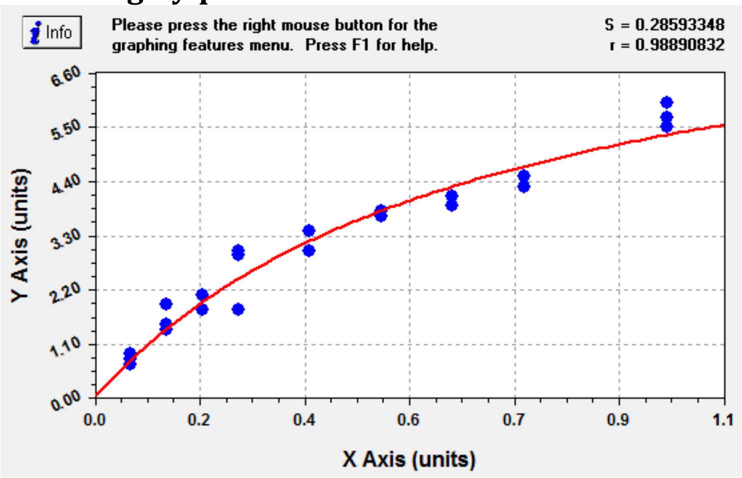

8P Angeloylpantetheine

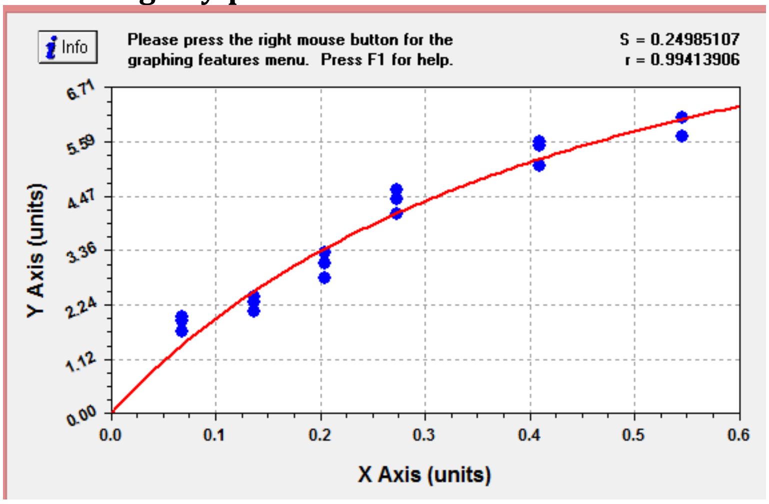

9P 2-3-dimethylbut-2-enoylpantethein

It was not possible to measure any rate. No product observed.

10P *E*-2-Ethylbut-2-enoylpantetheine

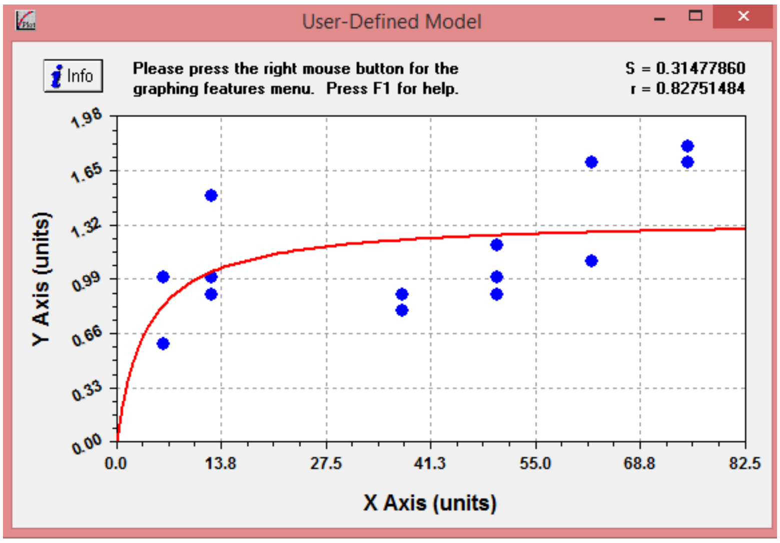

11P *E*-Hex-2-enoylpantetheine

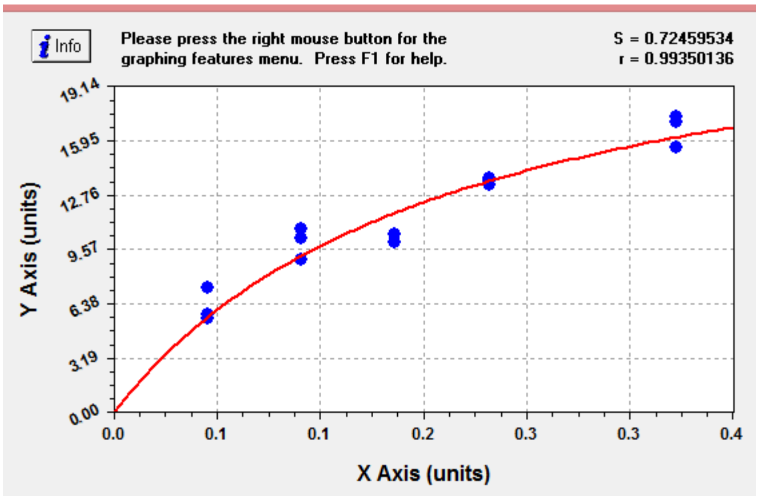

12P *E*-2-methylhex-2-enoylpantetheine

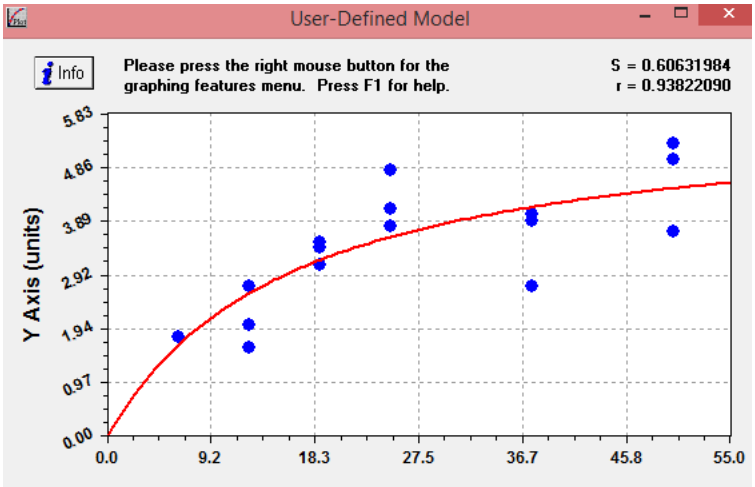

13P ***E*-4-methylhex-2-enoylpantetheine**

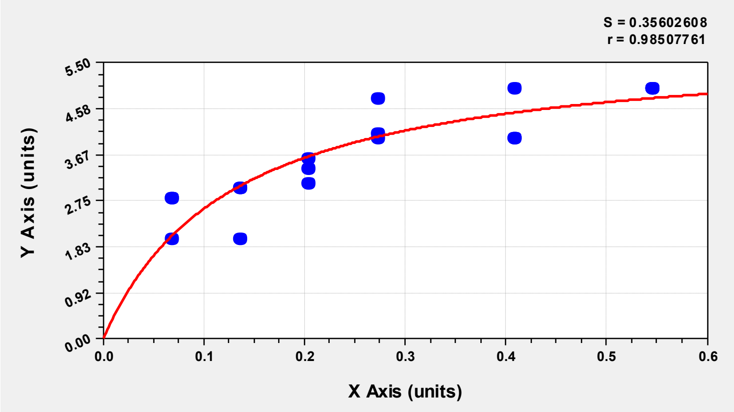

14P ***E*-2,4-dimethylhex-2-enoylpantetheine**

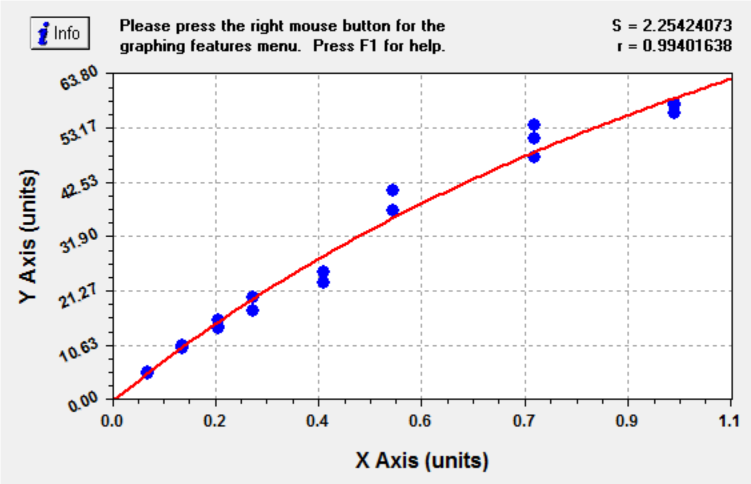

15P ***E*-4*S*-2,4-dimethylhex-2-enoylpantetheine**

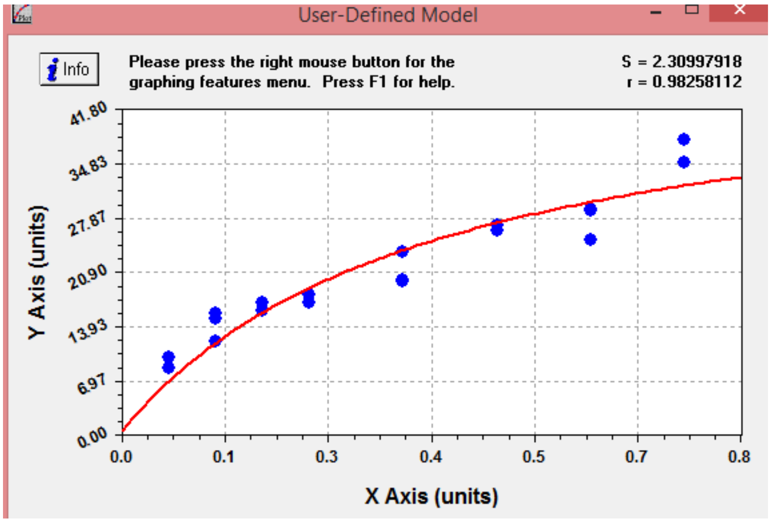

16P *E*-2-Ethylhex-2-enoylpantetheine

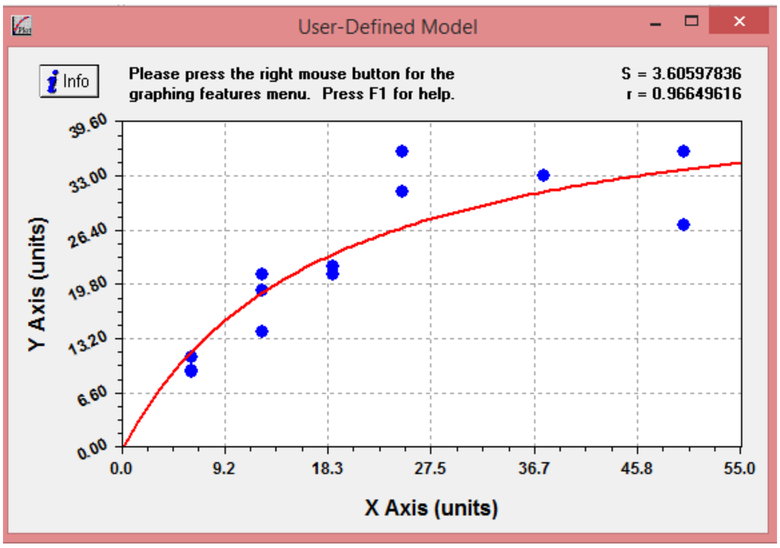

17P *E*-hept-2-enoylpantetheine

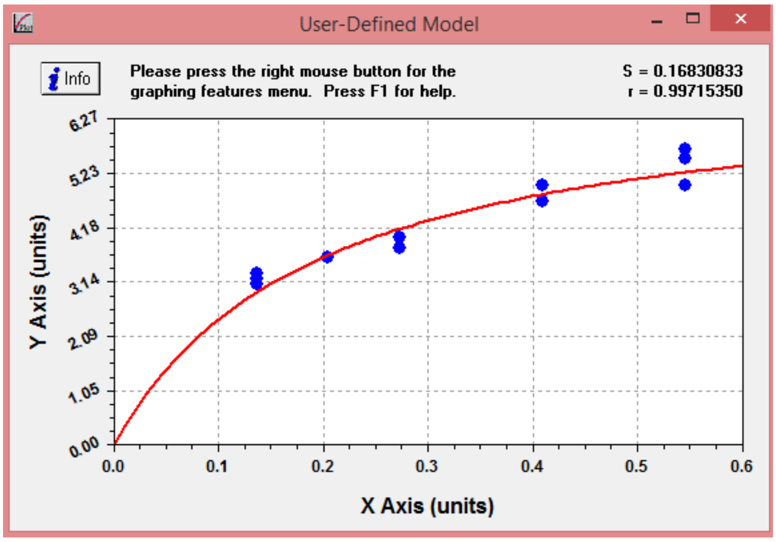

18P *E*-6-methylhept-2-enoylpantetheine

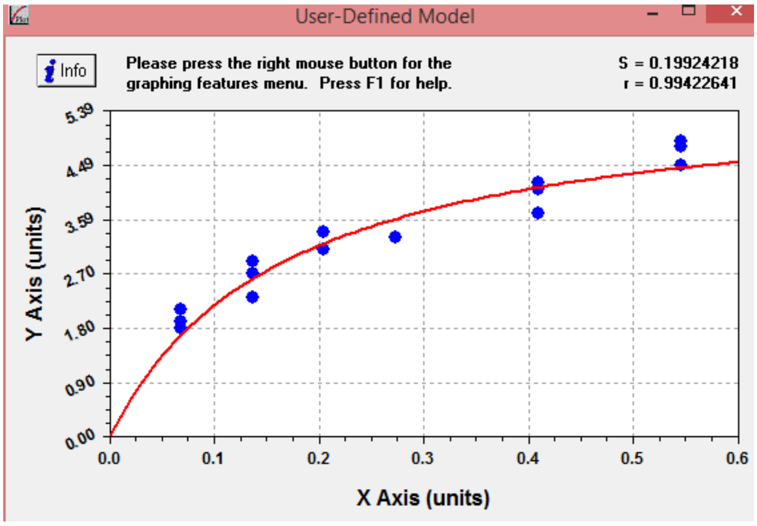

19P *E*-oct-2-enoylpantetheine

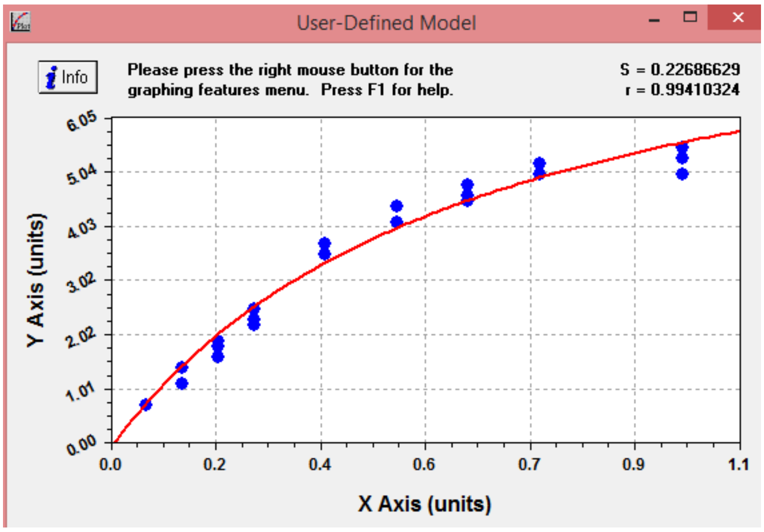

20P *E*-2-methyloct-2-enoylpantetheine

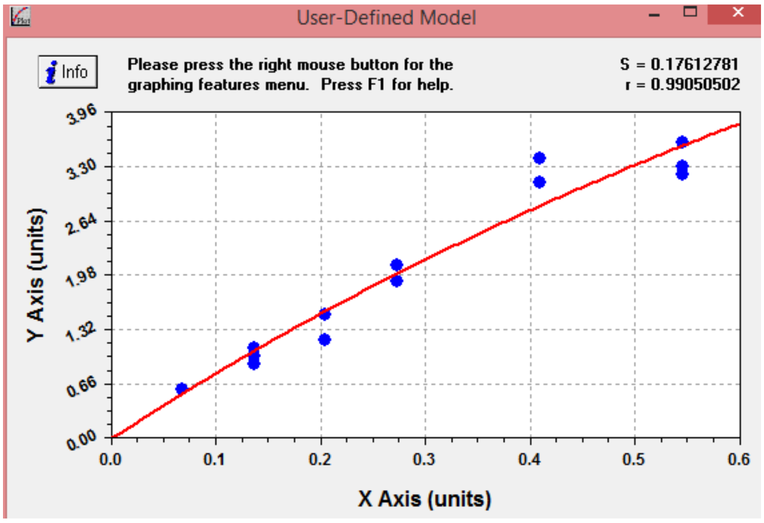

21P *E*-7-methyloct-2-enoylpantetheine

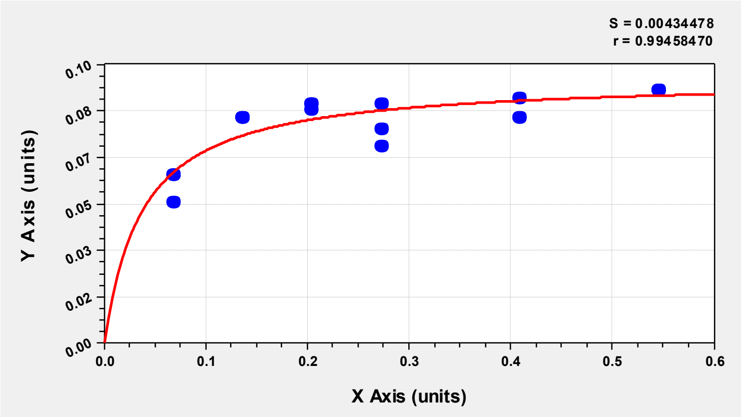

22P 4*RS*,6*RS*-*E*-4,6-dimethyloct-2-enoylpantetheine

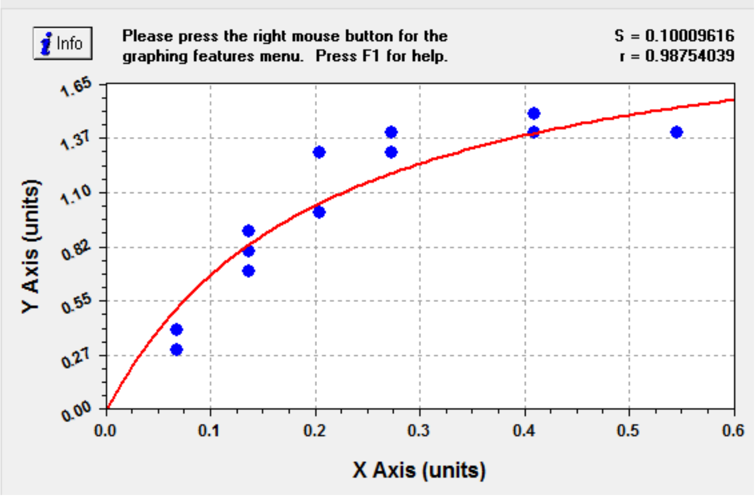

1P *E*-4*S*-6*S*-4,6-dimethyloct-2-enoylpantethein

It was not possible to measure any rate.

24P *E*-dec-2-enoyl pantetheine

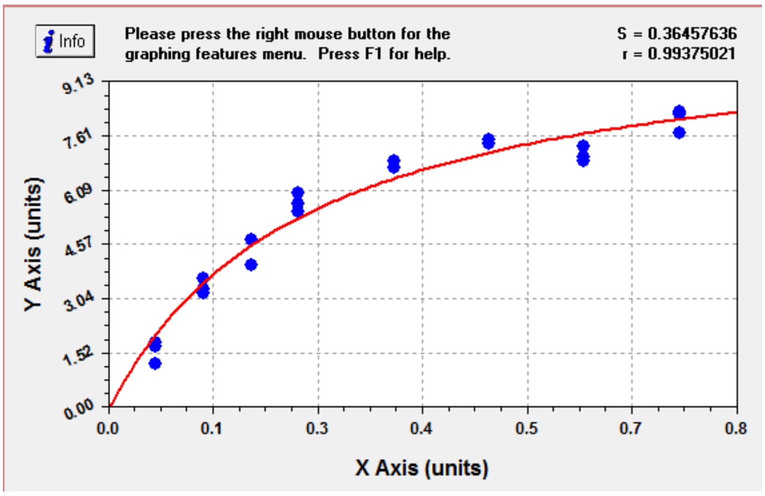

25P *E*-2-methyldec-2-enoyl pantetheine

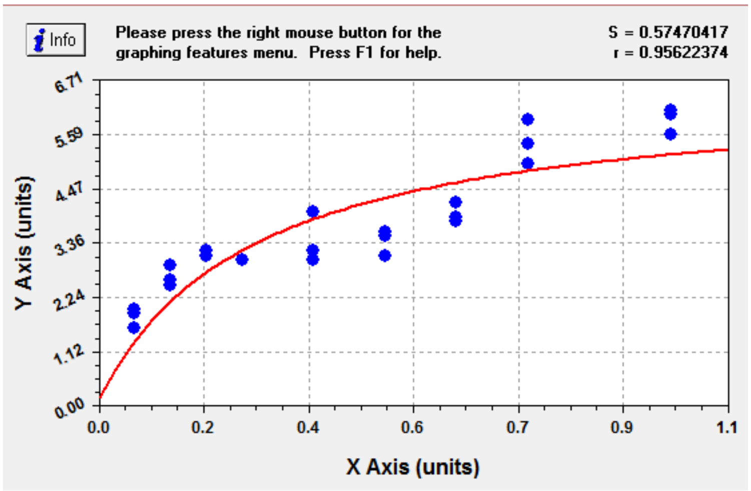

3.3 Inhibition Assay

Procedere as for Section **3.2**. Assays were performed in total volume of 400 µl; including: 5 - 40 µl Substrate (5 mmol); 10 µl NADPH (10 mmol); 20 µl Enzyme (def. 0.02 mU); 320-355 µl Buffer. (PANT); 5-10 µl SQTK (5 mmol).

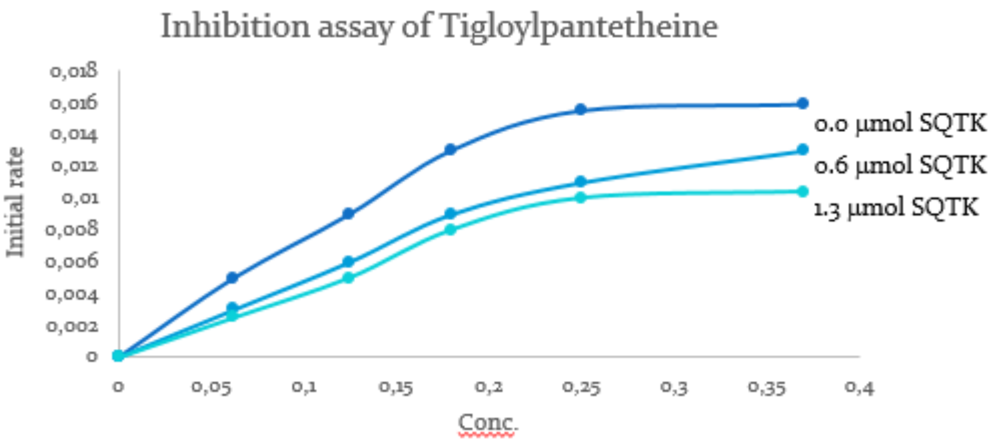

4.0 Stereochemical Assay Procedures

4.1 Analysis using diphenylethylene diamine

NMR assays were performed with tiglyl-SNAC **5S** (2.0 mg, 10 mmol), NADPH (8 mg, 10 mmol), tris buffer pH 8.5 (50 µl, 1M stock), glycerol (20% v/v) and the isolated ER domain (380 µU, 100 µl of stock) in water (total volume 1 ml). The progress of the reaction was monitored by LCMS. After 24 h further NADPH (8 mg, 10 mmol) was added to the reaction. After a further 17 h the protein was precipitated with CH_2Cl_2 (1 ml) and centrifuged (3000 rpm, 20 min). The organic layer was removed and the aqueous layer was further extracted with CH_2Cl_2 (2 × 1 ml). The organic fractions were combined and the solvent was removed under a flow of N_2 and then dried in *vacuo* for a further 2 h. To the dried sample was added water (450 µl) and aqueous NaOH (2M, 50 µl). The sample was left for 90 mins at RT and then acidified with aqueous HCl (2M, 100 µl) to pH 3 and extracted with $CDCl_3$ (4 × 200 µl). The organics were combined, dried with $MgSO_4$ and then filtered through $MgSO_4$ directly into an NMR tube. (1*R*, 2*R*)-(+)-1, 2 diphenylethylenediamine was titrated (100 mM stock) to optimize resolution of the obtained spectra.

4.2 Formation and Analysis of Methyl Mandelates

A solution of tiglyl/angelic-PANT **5P**/**8P** (3.6 mg, 10 mmol), NADPH (856 µl of 12.5 mM stock), tris buffer pH 8.5 (50 µl 1M stock), glycerol (500 µl, 50% v/v) and SQTKS ER (80 mU, 400 µl, 0.2 mU/µl stock) was incubated at 30 °C and the progress of the reaction was monitored by LCMS. After 24 h further SQTKS ER (200 µl) was added and the reaction mixture was incubated at 30 °C for 24 h. On completion CH_2Cl_2 (1 ml) was added and the sample was centrifuged (3000 rpm, 20 min) and the organics removed. The aqueous layer was extracted with CH_2Cl_2 (2 × 2 ml) and the organics were combined and concentrated under a flow of N_2 and then further concentrated *in vacuo* for 2 h.

After concentration, water (450 µl) and aqueous NaOH (2M, 50 µl) were added. This was left for 90 min and then acidified with aqueous HCl (2M, 100 µl) and then aqueous $CuSO_4$ (saturated, 400 µl) was added to remove panthetheine. The copper solution was extracted with CH_2Cl_2 (3 × 1 ml) and the organics were dried ($MgSO_4$) and concentrated to 1 ml under a flow of N_2. To the concentrated solution was added *S*-methyl mandelate (2.1 mg, 12.6 µmol), EDCI (2.4 mg 12.6 µmol) and DMAP (0.1 mg) and the reaction was followed by TLC. On completion aqueous HCl (2M, 0.5 ml) was added and the solution was extracted with CH_2Cl_2 (3 × 0.5 ml). The organics were dried ($MgSO_4$) and concentrated with N_2 and redisolved in $CDCl_3$ and were analyzed directly by NMR.

4.3 Analysis of use of 4'-2H NADPH isomers

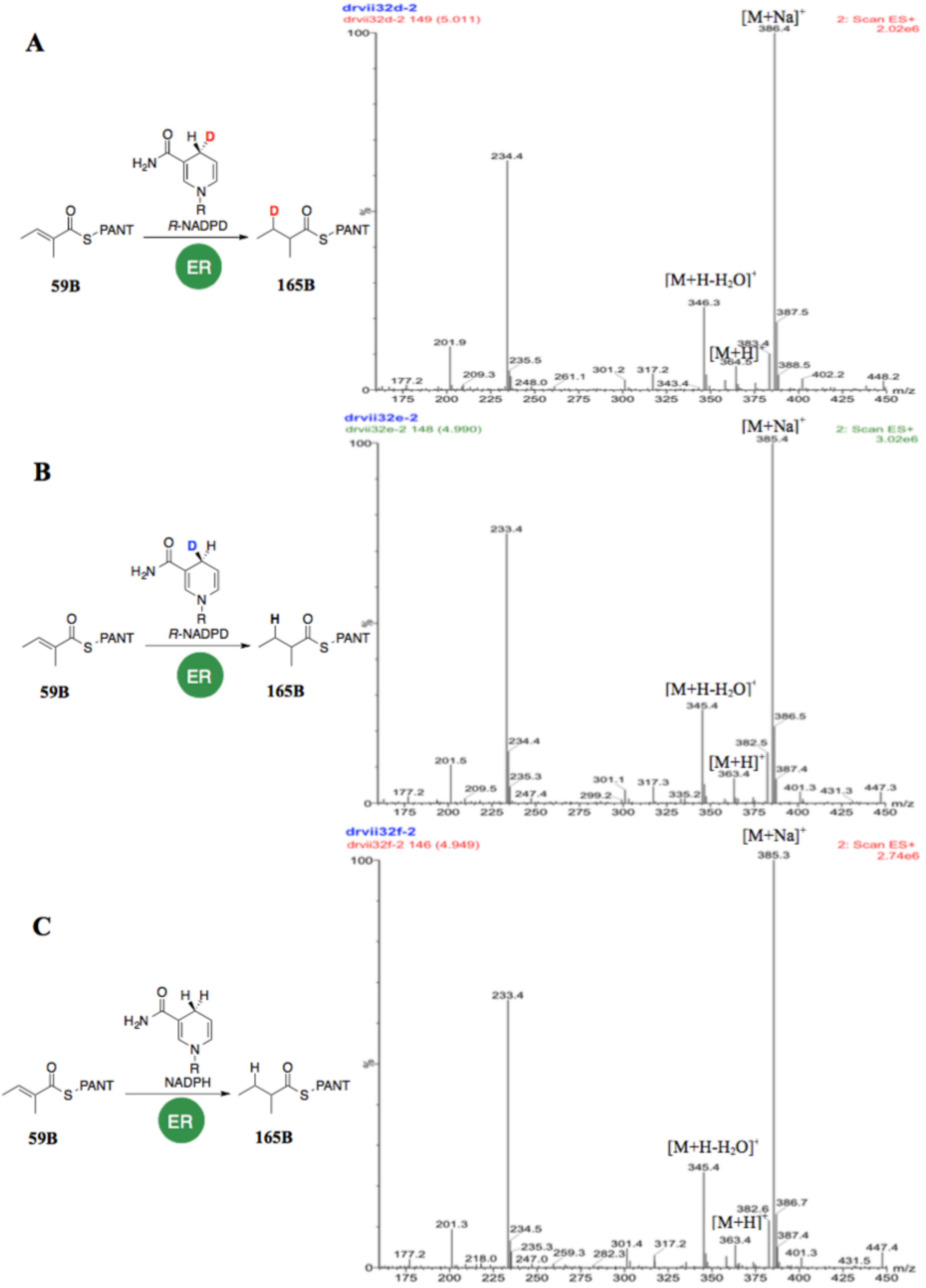

Fig S4.3.1. MS analysis of reduction of tigloyl pantethiene by isotopically labelled ^{2}H-isomers of NADPH.

5. Stereochemical Assignment of Proton Chemical Shifts of 2-Methyl Butyric Acid.

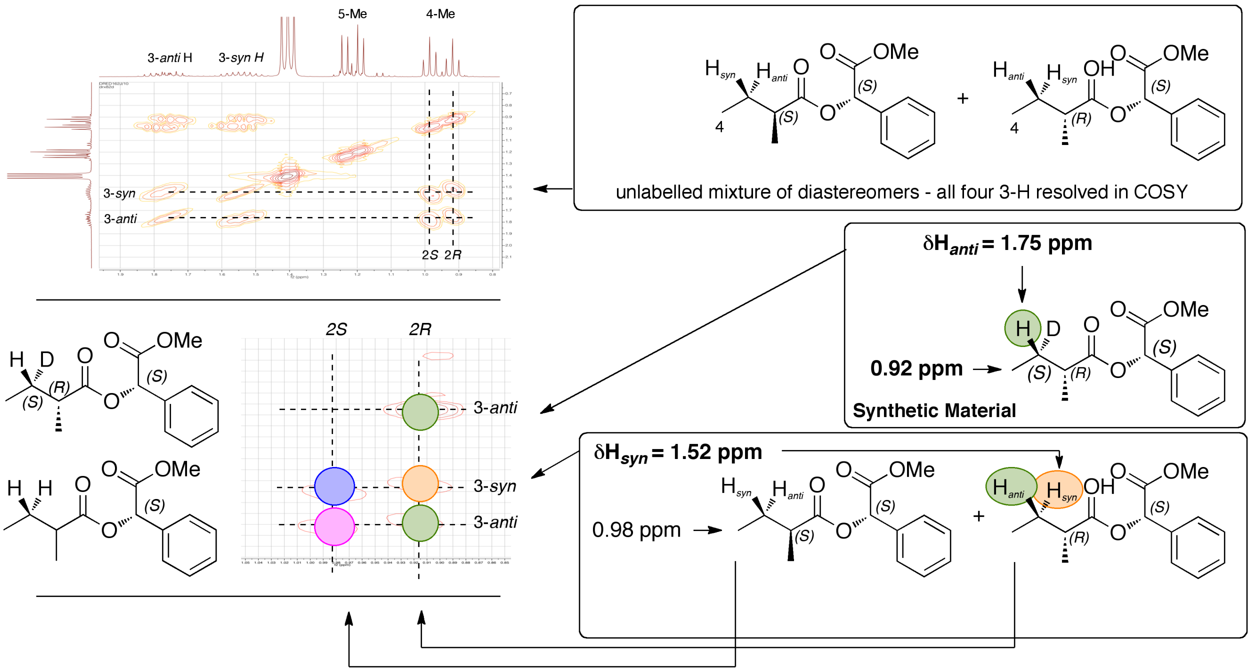

1. Synthetic 2*R* material identifies chemical shift of 4-methyl at 0.92 ppm. Therefore chemical shift of 4-methyl in 2*S* material is 0.98 ppm.
2. Synthetic 2*R* material identifies chemical shift of *anti* 3-H at 1.75 ppm (green); thus *syn* 3-H resonates at 1.52 ppm (orange).
3. Reduction of *E*-2-methyl butyrate by ER gives only two products.* One is the 2*R*,3*R* (orange) diastereomer showing 3-*Re* face attack by D. The other is 2*S*, and H is therefore *anti*, thus 3*R* (purple).

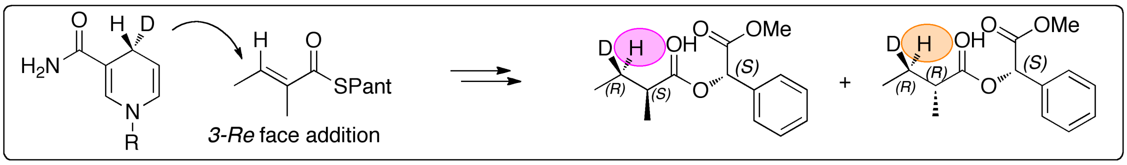

4. The final ^{1}H resonance in the COSY must therefore arise from the 2*S* 3-*syn* proton (blue).

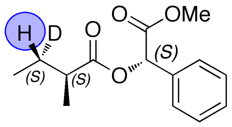

5. Reduction of *Z*-2-methyl butyrate by ER gives two products. These are asigned as the 2*S* 3-*syn* (blue) and 2*R* 3-a*nti* (green) diastereomers and this shows that D addition occurs at the 3-*Si* face, *i.e* with the same absolute sense as reduction of the *E*-substrate.

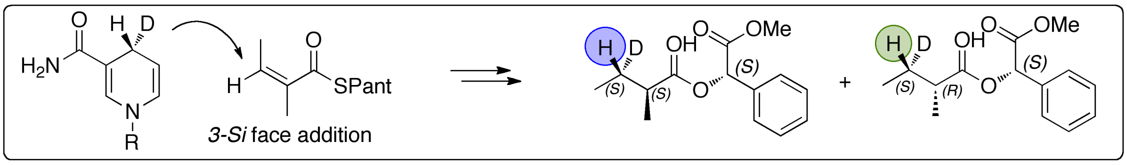

* Since it is known that the reprotonation step gives racemate at C-2, the origin of the 2-diastereomers observed must be due to the two configurations at C-2. Thus the configurations at C-3 must be identical. The substrate must be bound in a fixed orientation for only two products to be observed.

6. LCMS conditions

6.1 Analytical LCMS

LC-MS data were obtained with using a Waters LCMS system comprising of a Waters 2767 autosampler, Waters 2545 pump system, a Phenomenex Kinetex column (2.6 μ, C_{18}, 100 Å, 4.6 × 100 mm) equipped with a Phenomenex Security Guard precolumn (Luna C_5 300 Å) eluted at 1 ml/min. Detection was by Waters 2998 Diode Array detector between 200 and 600 nm; Waters 2424 ELSD and Waters SQD-2 mass detector operating simultaneously in ES + and ES- modes between 100 *m/z* and 650 *m/z*. Solvents were: A, HPLC grade H_2O containing 0.05% formic acid; B, HPLC grade MeOH containing 0.045% formic acid; and C, HPLC grade CH_3CN containing 0.045% formic acid. Gradients were as follows. *Method 1*. Kinetex/CH_3CN: 0 min, 10% C; 10 min, 90% C; 12 min, 90% C; 13 min, 10% C; 15 min, 10% C.

6.2 Semi-Preparative LCMS and Compound Purification.

Purification of all pantetheines was generally achieved using a Waters mass-directed autopurification system comprising of a Waters 2767 autosampler, Waters 2545 pump system, a Phenomenex Kinetex Axia column (5μ, C_{18}, 100 Å, 21.2 × 250 mm) equipped with a Phenomenex Security Guard precolumn (Luna C_5 300 Å) eluted at 20 ml/min at ambient temperature. Solvent A, HPLC grade H_2O + 0.05% formic acid; Solvent B, HPLC grade CH_3CN + 0.045% formic acid. The post-column flow was split (100:1) and the minority flow was made up with HPLC grade MeOH + 0.045% formic acid to 1 ml·min^{-1} for simultaneous analysis by diode array (Waters 2998), evaporative light scattering (Waters 2424) and ESI mass spectrometry in positive and negative modes (Waters SQD-2). Detected peaks were collected into glass test tubes. Combined tubes were evaporated (vacuum centrifuge), weighed, and residues dissolved directly in solvent for use or analysis.

7.0 Multiple alignment of ER sequences

```
SQTKS         --EPFHQ--- ---------- ------PGKL LQMGIKTPGL IDTLQFS-KT DATDN-LPND 1911
Azanigerone   IHCRFRD--- ---------- ------AARP LRMKIGTPGL IDTLVFHDDL DAKSDPLPAD 1860
Spin          QLALRRGGAH APRLAGLGSD DVLPVPDGTG WRLEATRPGS LDGLALVDEP TATAP-LGDG 239
Geldanmycin   QVALRRGLLW VPRLV--RSP QGLAVPAHEH WYLDVSEKGS LENLVLRPDP EATAP-LATG 2519
Borrelidin    ELVPPAG--- ---------- -------ADA WRLEITEPGT FDNLTLGVYP HAEKT-LADN 1429
erythromycin  RLSPLAP--- ---------- -SALTLPAGT QRLVPGDG-A IDSVAFEPAP DVEQP-LRAG 2914
Megalomicin   RLTPLAG--- ---------- -PVPAVADRA YRLVPGNGGS IEAVAFAPVP DADRP-LAPE 2843
vFAS RHFPLE         Q--- ---------- -DRPEKQTEH AFVNVLSRGD LSSIRWVCSP LHYALPASCQ 1561

                              Substrate Binding
SQTKS         YIEIEPKAFG LNFRDVMVAM GQLEE-SIMG FECAGVVRRV GPSSAGHNIK VGDRVCALLG 1970
Azanigerone   WIEFDPTAFG LNFRDVMVAM GQLEANAIMG FECAGTIVRL GATAAAKGFA VGDRVCTLLR 1920
Spin          EVRIAMRAAG VNFRDALIAL GMYPGVASLG SEGAGVVVET G--PGVTGLA PGDRVMGMIP 297
Geldanmycin   QVRIEVRAAG QNFRDVLVAL GGVAGQEGLG GEGAGVVTEV G--PGVEGLA VGDRVMGLFP 2577
Borrelidin    EVRVAVHAGG LNFHDVVAAL GMVEDDLTLG REAAGVVVEV G--DAVPDLT PGDHVMGILS 1487
erythromycin  EVRVDVRATG VNFRDVLLAL GMYPQKADMG TEAAGVVTAV G--PDVDAFA PGDRVLGLFQ 2972
Megalomicin   EVRVAVRATG VNFRDVLLAL GMYPEPAEMG TEASGVVTEV G--SGVRRFT PGQAVTGLFQ 2901
vFAS          DRLCSVYYTS LNFRDVMLAT GKLSPDSIPG KWLT-RDCML G-MEFSGRDA SGRRVMGMVP 1618
                         .* .       .        ^                         ^  

                                                   Cofactor         Ross
SQTKS         GQ-WTNTVRV HWHSVAPIPQ AMDWETAASI PIVFVTAYIS LVKIARMQAG ETVLIHAASG 2029
Azanigerone   GH-WATRPRA PWTSVMRIPQ HLSDQEAASF PTVFATAYIA LHETARLQRG ESILIHAATG 1979
Spin          KA-FGPLAVA DHRMVTRIPA GWSFARAASV PIVFLTAYYA LVDLAGLRPG ESLLVHSAAG 356
Geldanmycin   RS-FGPLAIA DARTVAPIPE GWSYATAAGV PVAYLTALYG LRDLGTVQPG ETVLVHAAAG 2636
Borrelidin    SG-FGPLAVT DHRYLARMPE GWTFAQAASV PAAFLTAYYG LCDLGGIRAG DRVLIHAAAG 1546
erythromycin  GA-FAPIAVT DHRLLARVPD GWSDADAAAV PIAYTTAHYA LHDLAGLRAG QSVLIHAAAG 3031
Megalomicin   GA-FGPVAVA DHRLLTPVPD GWRAVDAAAV PIAFTTAHYA LHDLAGLQAG QSVLVHAAAG 2960
vFAS          AEGLATSVLL LQHATWEVPS TWTLEEAASV PIVYTTAYYS LVVRGRMQPG ESVLIHSGSG 1678
                                             ^ .^    .                    !

              MANN                  Cofactor
SQTKS         GVGQAAIILA KHVGAEIFAT VGTDEKRD-L LIKEYKIPDD HIFSSRNALF AKSIRQRTNG 2088
Azanigerone   GVGQAAIQLA QLIGAEIYAT ASTPAKRQ-L LHETYGIPEN NIFSSRDPSF ATDVHLRTDG 2038
Spin          GVGMAAIQLA RHLGAEVYAT ASE-DK---- -WQAVELSRE HLASSRTCDF EQQFLGATGG 410
Geldanmycin   GVGMAAVQLA RHFGATVYAT AHP-SKHH-- VLTALGVPEG HLASSRDLGF ASAFP----- 2688
Borrelidin    GVGMAAVQIA RHLGAEVFGT ASP RKWG-- ALRALGLDDA HLSSSRTLDF EQEFLDATDG 1603
erythromycin  GVGMAAVALA RRAGAEVLAT AGP AKHG-- TLRALGLDDE HIASSRETGF ARKFRERTGG 3088
Megalomicin   GVGMAAVALA RRAGAEVFAT ASP AKHP-- TLRALGLDDD HIASSRESGF GERFAARTGG 3017
vFAS          GVGQAAIAIA ISRGCRVFTT VGSAEKRAYL QARFPQLDET CFANSRDTSF EQHVLRHTAG 1738
                                         !                 !                  

SQTKS         KGVDVVLNCL AGGLLQESFD CLADFGRFIE IGKRDIELNH CLNMGMFARS ATFTAVDLIA 2148
Azanigerone   RGVDVVLNSL AGRLLQESFN CLAEFGRMVE IGKRDLEQHS GLDMYPFTRN VSFSSVDLLT 2098
Spin          RGVDVVLNSL AGEFADASLR MLPRGGRFLE LGKTDVRDPV EV--ADAHPG VSYQAFDT-- 466
Geldanmycin   -ALDVVLNSL TGEYVDASLG LLGTGGRFVE MGKNDIRDPA SV--AAAHPG VGYQAFDLG- 2744
Borrelidin    RGVDLVLNSL AREFVDASLR LMPGGGRFVD MGKTDIRRPE QV--AEDHGG VAYQAFDL-- 1559
erythromycin  RGVDVVLNSL TGELLDESAD LLAEDGVFVE MGKTDLRD-- ----AGDFRG R-YAPFDL-- 3139
Megalomicin   RGVDVVLNSL TGDLLDESAR LLADGGVFVE MGKTDLRP-- ----AEQFRG R-YVPFDL-- 3068
vFAS          KGVDLVLNSL AEEKLQASVR CLAQHGRFLE IGKFDLSNNH ALGMAVFLKN VTFHGILLDS 1798
                      **           ! .                        !^^.  

                                C-terminal
SQTKS         IGRDRSYMFA EALPKIMTLL QEKAIRPVTP ISIYKIGDIE TAFRLMQAGK HMGKIVITAP 2208
Azanigerone   WQSRRGADIS CVLQSLSKLL GEKKIMPVYP LTLYPITQIE KAFRTMQTGQ HMGKIIISVG 2158
Spin          -VEAGPQRIG EMLHELVELF EGRVLEPLP- VTAWDVRQAP EALRHLSQAR HVGKLVLTMP 524
Geldanmycin   -GDAGPDRIR ELLAELVELF EAGRIEPLP- IRHWDVTQAP TAFRWMSQGR HTGKIVLTLP 2802
Borrelidin    -VEAGPQRTG EMLAEIVRLF QAGAFRPLP- ITQWDVRRAP EAFRHISQAK HIGKIVLTVP 1717
erythromycin  -GEAGDDRLG EILREVVGLL GAGELDRLP- VSAWELGSAP AAIQHMSRGR HVGKLVLTQP 3197
Megalomicin   -AEAGPDRLG EILEEVVGLL AAGALDRLP- VSVWELSAAP AALTHMSRGR HVGKLVLTQP 3126
vFAS          LFEEGGATWQ EVSELLKAGI QEGVVQPLK- CTVFPRTKVE AAFRYMAQGK HIGKVVIQVR 1857
              ^
```

. = residues identified by Leadlay, Ban and others as possibly involved in enol(ate) reprotonation; ! = residues contacting NADPH; * = residues contacting the pantetheine; ^ = residues contacting the substrate.

colour code. A = 8/8 conserved; A = 7/8 conserved; A = 6/8 conserved; A = 5/8 conserved;

Domain colours (blue, red, green, grey) and descriptions correspond to those in Figs 4, 5 and 6.

8.0 Modelling and Docking Procedures

ER Homology Model:
A homology of the ER domain with NADP bound was generated using the SWISS-MODEL protein structure homology-modelling server in the fully automated mode.[33] The PDB entry 2VZ9 was used as the template structure.[34]

Pantetheine Docking:
Ligand structures were generated using Chem3D Pro 13.0 (Perkin Elmer). The ligands were docked manually into the active site using PyMOL (The PyMOL Molecular Graphics System, Version 1.3 Schrodinger, LLC.) and the resulting protein-ligand complex was energy minimised using the YASARA energy minimization server.[35] The docked ligand was removed from the resulting protein model and re-docked using Autdock Vina 1.1.2.[36] The ligand files and ER PDB file were converted into PDBQT format using Autodock Tools 1.5.6[37] to add polar hydrogens and set rotatable bonds. Ligands were docked using a grid of 44 Å (x) x 22 Å (y) x 16 Å (z) centred on the active site at position 45.8 (x), 143 (y), 76.556 (z). Images were processed using PyMol.

pdb files of representative docked structures are appended as supplementary information.

9.0 Integration of Angelic Mandelate Spectrum

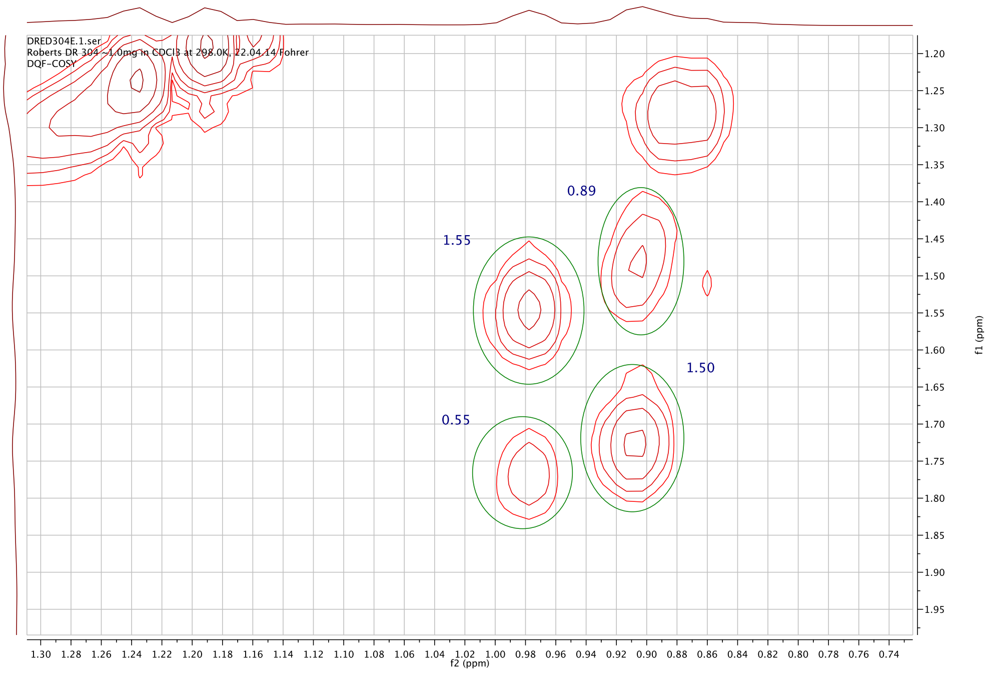

Integration of crosspeaks for **30** from reduction of **8P** was achieved using MestreNova 9.0 Software using standard parameters.

9.0 References

1. Jackson, David A.; Rey, Max; Dreiding, Andre S., *Helv. Chim. Acta*, **1983** , *66*, 2330 - 2341.
2. Ramamoorthy, G.; Acevedo, C. M.; Alvira, E.; Lipton, M. A., *Tetrahedron: Asymmetry* **2008**, *19*, 2546.
3. Hadrami, El, M.; Lavergne, J. P.; Viallefont, P.; Itto, M., *Tetrahedron Letters* **1991**, *32*, 3985.
4. Rossi, R.; Carpita, A.; Cossi, P. *Tetrahedron* **1992**, *48*, 8801.
5. Das, Biswanath; Banerjee, Joydeep; Chowdhury, Nikhil; Majhi, Anjoy; Mahender, Gurram, *Helv. Chimi. Acta*, **2006** , *89*, 876 - 883
6. T. Mimura, Y. Kimura and T. Nakai, *Chem. Lett.*, **1979**, 1361–1364.
7. Blanc,P.-Y. et al., *Helv. Chim. Acta*, **1964** , *47*, 567 - 575
8. Concellón, J. M.; Concellón, C., *J. Org. Chem.* **2006**, *71*, 1728.
9. H. H. Wasserman, A. K. Petersen and M. Xia, *Tetrahedron*, **2003**, *59*, 6771.
10. T. E. Lightburn, O. A. De Paolis, K. H. Cheng, and K. L. Tan, *Org. Lett.*, **2011**, *13*, 2686.
11. F. Bargiggia and O. Piva, *Tetrahedron: Asymmetry*, **2003**, *14*, 6771.
12. Ahad, S. M.; Ange, A. L.; Bates, R. B.; Bell, B. L.;Bodour, A. A.; Bourne, B. R.; Contreras, C. G.; Goldberg, E. L.; Gunatilaka, A. A. L.; King, S.; Lee, A. K.; Low, R. L.; Maier, R. M.; Marlor, K. M.; Marron, M. T.; Scolnik, R. C.; Streeter, M. J.; Strelczuk, M.; Trinh, L. N.; Truong, V. K.; Vissering, S. P.; Weick, M. C.; Williams, M. T., *Tetrahedron,* **2010**, *66*, 9107.
13. M. Morr, V. Wray, J. Fortkamp and R. D. Schmid, *Liebigs Ann. der Chem.*, **1992**, 433.
14. Cox, R. J.; Glod, F.; Hurley, D.; Lazarus, C. M.; Nicholson, T. P.; Rudd, B. A. M.; Simpson, T. J.; Wilkinson, B.; Zhang, Y., *Chem. Commun.*, **2004**, 2260 - 2261.
15. B. Bonsch, V. Belt, C. Bartel, N. Duensing, M. Koziol, C. M. Lazarus, A. M. Bailey, T. J. Simpson and R. J. Cox, *Chem. Commun.*, **2016**, *52*, 6777–6780.
16. L. Raffier and O. Piva, *Beilstein J. Org. Chem.*, **2011**, *7*, 151.
17. Lee, M. S.; Qin, G.; Nakanishi, K., *J. Am. Chem. Soc.*, **1989**, *11*, 6234.
18. Moore, Bradley S.; Seng, Dieter, *Tetrahedron Letters*, **1998** , 39, 3915 - 3918.
19. F. Wang, Y. Wang, J. Ji, Z. Zhou, J. Yu, H. Zhu, Z. Su, L. Zhang and J. Zheng, *ACS Chem. Biol.*, 2015, **10**, 1017–1025.
20. Jensen, Katja; Niederkrueger, Holger; Zimmermann, Katrin; Vagstad, Anna L.; Moldenhauer, Jana; Brendel, Nicole; Frank, Sarah; Poeplau, Petra; Kohlhaas, Christoph; Townsend, Craig A.; Oldiges, Marco; Hertweck, Christian; Piel, Joern, *Chemistry and Biology*, **2012** , *19*, 329 - 339
21. Speir; Barnsley, *Biochem. J.*, **1971**, *125*, 267 -2 69.
22. Winter, Jaclyn M.; Cascio, Duilio; Dietrich, David; Sato, Michio; Watanabe, Kenji; Sawaya, Michael R.; Vederas, John C.; Tang, Yi, *J. Am. Chem. Soc.*, **2015** , *137*, 9885 - 9893.
23. Mizugaki, Michinao; Hoshino, Toshiaka; Ito, Yoko; Sakamoto, Takao; Shiraishi, Takayuki; Yamanaka, Hiroshi, Chem. Pharm. Bull., **1980**, *28*, 2347 - 2350.
24. Singh, Renu; Reynolds, Kevin A., *ChemBioChem*, **2015**, *16*, 631 - 640.
25. Schwab, John M.; Klassen, John B., *J. Am. Chem. Soc.*, **1984**, *106*, 7217 - 7227.
26. Agarwal, Vinayak; Diethelm, Stefan; Ray, Lauren; Garg, Neha; Awakawa, Takayoshi; Dorrestein, Pieter C.; Moore, Bradley S., *Org. Lett.*, **2015**, *17*, 4452 - 4455.
27. M. Koller, M. Karpf and A. S. Dreiding, *Helv. Chim. Acta.*, **1986**, *69*, 560 - 579.
28. Gay, Darren C.; Spear, Philip J.; Keatinge-Clay, Adrian T., *ACS Chem. Biol.*, **2014**, *9*, 2374 - 2381.
29. Ottolina, G.; Riva, S.; Carrea, G.; Danieli, B.; Buckmann, A. F. Biochimica et Biophysica Acta (BBA) - Protein Structure and Molecular Enzymology 1989, 998, 173.; Podschun, B.; Jahnke, K.; Schnackerz, K. D.; Cook, P. F. J. Biol. Chem., **1993**, *268*, 3407.
30. Jeong, S. S.; Gready, J. E., *Anal. Biochem.*, **1994**, *221*, 273.
31. Kashima; Fukuchi; Hosomi, *J. Org. Chem.*, **1994**, *59*, 7821 - 7824.
32. Micoine, K.; Fürstner, A., *J. Am. Chem. Soc.,* **2010**, *132*, 14064.

33. M. Biasini, S. Bienert, A. Waterhouse, K. Arnold, G. Studer, T. Schmidt, F. Kiefer, T. G. Cassarino, M. Bertoni, L. Bordoli, T. Schwede, *Nucleic Acids Res.*, **2014**, *42*, W252 - W258; Arnold K., Bordoli L., Kopp J., and Schwede T., *Bioinformatics*, **2006**, *22*, 195 - 201; Kiefer F, Arnold K, Künzli M, Bordoli L, Schwede T, *Nucleic Acids Res.*, **2009**, *37*, D387 - D392; Guex, N., Peitsch, M.C., Schwede, T., *Electrophoresis*, **2009**, *30*, S162 - S173.

34. T. Maier, M. Leibundgut and N. Ban, *Science*, **2008**, *321*, 1315-1322.

35. E. Krieger, K. Joo, J. Lee, J. Lee, S. Raman, J. Thompson, M. Tyka, D. Baker and K. Karplus, *Proteins*, **2009**, *77*, 114 – 122.

36. O. Trott and A. J. Olson, *J. Comp. Chem.*, **2010**, *31*, 455 - 461.

37. Morris, G. M., Huey, R., Lindstrom, W., Sanner, M. F., Belew, R. K., Goodsell, D. S. and Olson, A. J., *J. Comp. Chem.*, **2009**, *16*, 2785 - 2791; M. F. Sanner, *J. Mol. Graphics Mod.*, **1999**, *17*, 57 - 61.
